# A Tool Set for the Genome-Wide Analysis of *Neurospora crassa* by RT-PCR

**DOI:** 10.1534/g3.115.019141

**Published:** 2015-08-06

**Authors:** Jennifer M. Hurley, Arko Dasgupta, Peter Andrews, Alexander M. Crowell, Carol Ringelberg, Jennifer J. Loros, Jay C. Dunlap

**Affiliations:** * Department of Genetics, Geisel School of Medicine, Hanover, New Hampshire 03755; † Institute for Biomedical Informatics, Perelman School of Medicine, Philadelphia, Pennsylvania 19104; ‡ Department of Biochemistry, Geisel School of Medicine, Hanover, New Hampshire 03755

**Keywords:** *Neurospora crassa*, reference genes, circadian, light induction, quinic acid induction

## Abstract

*Neurospora crassa* is an important model organism for filamentous fungi as well as for circadian biology and photobiology. Although the community-accumulated tool set for the molecular analysis of Neurospora is extensive, two components are missing: (1) dependable reference genes whose level of expression are relatively constant across light/dark cycles and as a function of time of day and (2) a catalog of primers specifically designed for real-time PCR (RT-PCR). To address the first of these we have identified genes that are optimal for use as reference genes in RT-PCR across a wide range of expression levels; the mRNA/transcripts from these genes have potential for use as reference noncycling transcripts outside of Neurospora. In addition, we have generated a genome-wide set of RT-PCR primers, thereby streamlining the analysis of gene expression. In validation studies these primers successfully identified target mRNAs arising from 70% (34 of 49) of all tested genes and from all (28) of the moderately to highly expressed tested genes.

Profiling gene expression at the mRNA level via real-time PCR (RT-PCR) is a rapid means of assessing an organism’s response to changing environmental conditions. In comparison to other techniques that also track the steady-state levels of mRNA such as Northern blot analysis, microarrays, and RNA-Seq, RT-PCR is cheaper, faster, requires less mRNA, and produces large amounts of data with limited effort. Two key pieces contributing to the success of this technique are (1) the efficient design of optimal primers that are used in the reaction and (2) reference genes to serve as internal controls for template inputs. RT-PCR primer design must take into account sequence position, exon position, product size, melting temperature (T_m_), secondary structure, and GC content, as well as the terminal nucleotides to create an optimal primer. To achieve this, programs to aid in primer design have been developed (Rozen and Skaletsky 2000; Untergasser *et al.* 2012) as well as Internet-based primer design resources (*e.g.*, Perfect Primer; Invitrogen, Waltham, MA). However, existing Internet-based primer design web sites are not high-throughput, leaving the user to provide individually not only the gene but also the genomic sequence of interest. Designing individual primers for target genes is a time-consuming process, and very often multiple primers for a single gene are required as part of the validation process; designing multiple RT-PCR primers per gene for multiple genes can quickly become tedious, and a genome-scale data set is optimal for large-scale efforts (Cui *et al.* 2007).

Neurospora has long been a salient model for nonyeast fungal species, including important plant and animal pathogens, and has been defined as a model organism by the NIH (http://www.nih.gov/science/models/). Its 43-Mb genome contains approximately 11,000 predicted genes (Galagan *et al.* 2003; Stajich *et al.* 2012) (http://fungidb.org/fungidb/). Haploid progeny, regulatable promoters (Hurley *et al.* 2012; Lamb *et al.* 2013; Giles *et al.* 1985), efficient and simple transformations (Chakraborty *et al.* 1991; Margolin *et al.* 1997), as well as an extensive culture collection (Fungal Genetics Stock Center) (McCluskey *et al.* 2010) and knockout library (Colot *et al.* 2006) (Dunlap *et al.* 2007) contribute to the utility of Neurospora for the study of a wide variety of biological processes, including many that are not fungal-specific. For example, in addition to being an established model organism for the study of circadian clock, Neurospora is commonly used to study fungal biomass deconstruction, epigenetics, fungal infection model, cell morphology, sexual development, gene silencing, and photobiology (Aramayo and Selker 2013; Riquelme *et al.* 2011; Znameroski and Glass 2013; Baker *et al.* 2012; Chen *et al.* 2009; Kuo *et al.* 2014; Lehr *et al.* 2014; Wang *et al.* 2014b; Fulci and Macino 2007).

Recently, extensive analyses of mRNA output via deep sequencing of mRNA levels as well as microarray analyses in Neurospora have thoroughly described circadian gene expression, as well as the organism’s response to light and to addition of quinic acid (Hurley *et al.* 2014; Logan *et al.* 2007; Wu *et al.* 2014; Sancar *et al.* 2015). By probing these data sets, we have identified genes that can potentially serve as stably expressed reference genes when tracking changes in mRNA levels under various experimental conditions. The identified genes have a broad range of expression levels, providing a variety of comparable and appropriate normalizing genes for any target gene of interest. In addition, we report the development of a genome-wide data set of RT-PCR primers to streamline RT-PCR analyses. To create this resource, we designed and implemented a program to generate a list of five optimal RT-PCR primers for each gene in the Neurospora genome. We used the industry-standard program Primer3 (Untergasser *et al.* 2012; Rozen and Skaletsky 2000) to identify multiple primer pairs that are statistically likely to generate reliable gene detection via RT-PCR studies and validated a selection of the primer pairs (0.5%) by testing their utility on target genes selected to span the range of gene expression levels.

## Materials and Methods

### Primer design

A software application was written in Python to retrieve regions from each ORF (designated from Assembly 12 of the Neurospora genome; www.broadinstitute.org/annotation/genome/neurospora/GenomeDescriptions.html#NC12; http://fungidb.org/fungidb/; http://www.ncbi.nlm.nih.gov/genome/?term=txid367110) (Stajich *et al.* 2012) (Supporting Information, File S1) and pass them to Primer3 (version 2.3.6; http://frodo.wi.mit.edu/cgi-bin/primer3/primer3_www.cgi), which automatically generated a list of candidate primers with designated parameters (Table S1). Three to five primer pairs were selected and primers were ordered for 49 genes and supplied in 96-well plates (Illumina, San Diego, CA), diluted, and mixed by a Biomek NX robot (Beckman, Danvers, MA). The 49 genes were selected because they were potentially circadianly regulated genes based on previous work (Hurley *et al.* 2014).

### RT-PCR protocols

One-thousand five-hundred ng of RNA [a mix of the 10- and 30-hr time points from the third RNA-Seq time course published in the work by Hurley *et al.* (2014) chosen to encompass maximal expression of genes at two different clock phases] was used to prepare cDNA using the SuperScript III First-Strand synthesis kit (Invitrogen, Waltham, MA). This was followed by RT-PCR using the Fast SYBR green master mix kit in an ABI 7500 real-time cycler (Applied Biosystems, Waltham, MA). The primer combinations used are listed in Table S1 and the final concentration of primers in the reaction mix was 0.5 μM. The following cycling parameters were used: step 1: 95° for 5 min and step 2: 95° for 10 sec and 60° for 30 sec for 40 cycles. C_t_ values were calculated using software provided by the instrument manufacturer. The relative mRNA levels for each time point were calculated using at least two out of three biological replicates in the case where one of the replicates differed from either of the other two by more than three-fold. Only a single biological replicate and primer concentration was tested.

### Data analysis

The functionality of each of the 49 selected primer pairs was tested. Each unique primer pair was assigned a C_t_ value with respect to its target by the ABI 7500 Fast Real-Time PCR software (Applied Biosystems, Waltham, MA) using the auto C_t_ command. Each C_t_ value represents a single technical replicate from the mix of the cDNA sample.

To determine which genes showed the least variation across time and would be optimal for use as reference genes over circadian time, we first rejected extremely low-expressing and nondetected genes with a log-transformed fragments per kilobase per million (FPKM) value cutoff of −2 from our RNA-Seq data. Next, we used our Jonckheere-Terpstra-Kendall (JTK) cycle (Hughes *et al.* 2010) results from Hurley *et al.* (2014), accounting for replicates, to reject genes called with a high confidence as being circadianly regulated (Benjamini Hochberg corrected q < 0.05). We then performed an ANOVA on the log_10_ FPKM values for the remaining genes, comparing within time point variation against between time point variation for each gene and rejected genes for which *P* < 0.05. Because we chose genes to reject, we did not adjust for multiple comparisons because that would introduce more type I errors while reducing type II errors; our goal is to screen out genes with strong variation and the cutoff is itself arbitrary, making such an adjustment unnecessary. This left us with a set of expressing, noncircadian, ANOVA selected (ENCAS) genes. In the last step of our analysis, linear regressions were performed on the same log_10_ FPKM values used for all previous calculations for each ENCAS gene using the scikit learn package (a set of data mining and machine learning tools) in python. These linear regressions were used to create prediction intervals (95%) for the expected mean at each time point for each gene. Genes were then scored based on the sum of the absolute value of the differences of the upper and lower bounds of these prediction intervals with respect to the overall mean expression of the gene at each time point. Under this scoring metric, which we have termed the prediction interval ranking score (PIRS), genes that are more stable across circadian time will have lower scores. For both the light-induction and quinic acid–induction (QA) time courses, neither the JTK cycle nor the ANOVA-based screening methods were applicable because the time courses were not circadian in nature and did not contain replicates. It is important to note that the light-induction data were all normalized to the initial time point for each gene; because of this, we could not examine absolute expression levels and identify the best genes from each expression quintile for the light-induction data set.

In addition, as an independent analysis, normalized, log-transformed FPKM values (Hurley *et al.* 2014) were averaged across each of the three reported circadian time courses as well as averaged across an average of the three time courses, and the SD of the log_10_ FPKM values for each gene over circadian time was determined. Genes where the SD of the log_10_ FPKM values was 0.5% or less of the log_10_ FPKM averages were selected as candidates. The list of genes was compiled by selecting genes that appeared in the averaged time course as well as at least one of the three individual time courses (Hurley *et al.* 2014) (Figure S1).

### Data availability

All strain, data analysis programs and ancillary information are either freely available or available upon request. Table S1 and File S1 contain all necessary information regarding the generated RT-PCR primers.

## Results

### Identifying a constitutively expressed reference gene

**Figure 1:**
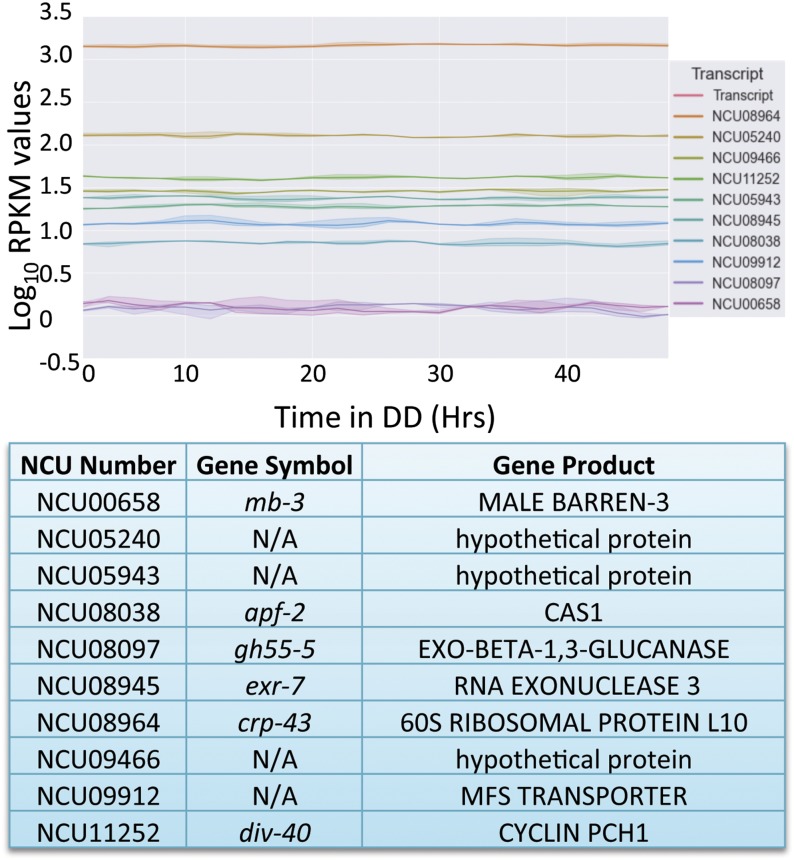
Optimal reference genes for RT-PCR in Neurospora. A graphical representation of the log_10_ of FPKM values from the RNA-Seq data set for the 10 Neurospora genes (two reported from each quintile) that demonstrated the least variability in expression over 2 d in culture. The chart reports the gene name as well as the gene symbol for each of the NCUs reported. Gene symbols are from the Neurospora e-Compendium at Leeds (http://www.bioinf.leeds.ac.uk/∼gen6ar/newgenelist/genes/gene_list.htm).

**Figure 2:**
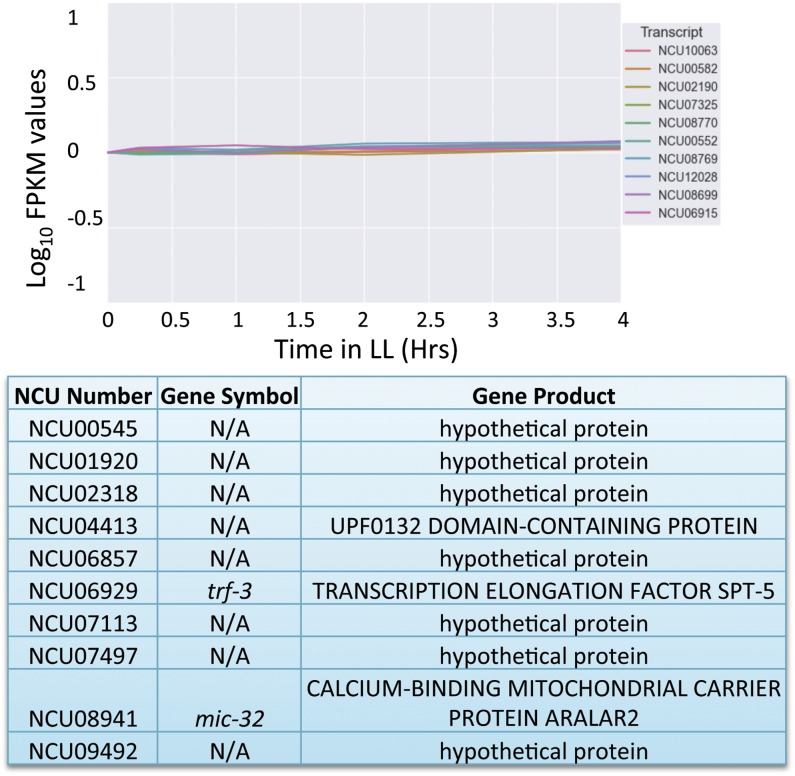
Optimal reference genes for RT-PCR under light induction in Neurospora. A graphical representation of the log_10_ of FPKM values from the RNA-Seq data set for the 10 Neurospora genes that demonstrated the least variability following light induction. Note that the light-induction data are normalized to the initial time point for each gene. The chart reports the gene name as well as gene symbol for each of the NCUs reported. Gene symbols are from the Neurospora e-Compendium at Leeds.

**Figure 3:**
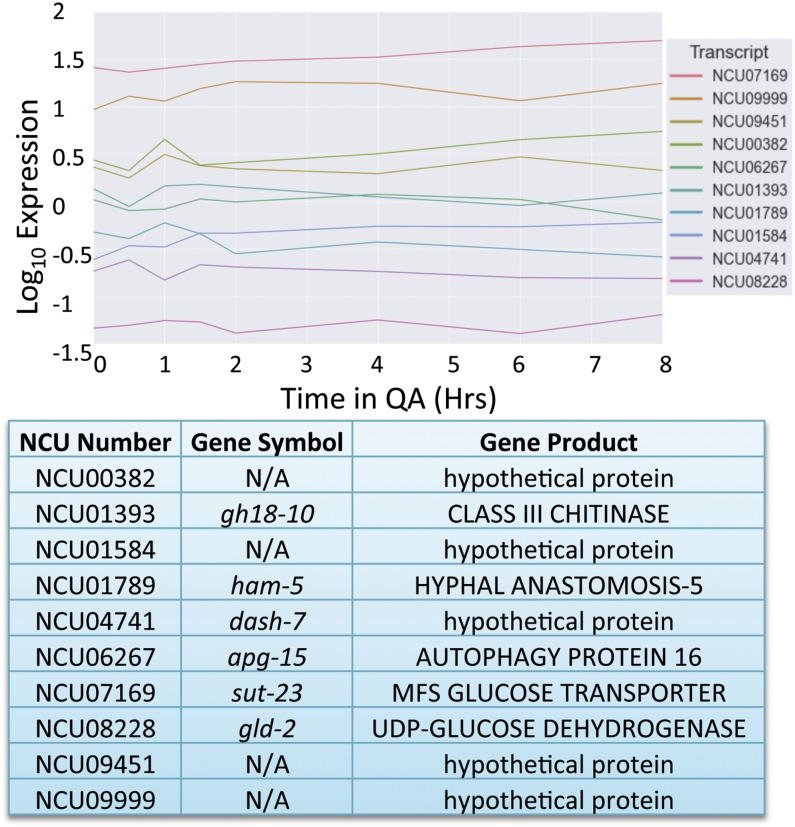
Optimal reference genes for RT-PCR under quinic acid (QA) induction. A graphic representation of the log_10_ of FPKM values from the RNA-Seq data set for the 10 Neurospora genes (two reported from each quintile) that demonstrated the least variability after the addition of QA. The chart reports the gene name as well as gene symbol for each of the NCUs reported. Gene symbols are from the Neurospora e-Compendium at Leeds.

To identify genes with near-constant expression in Neurospora, we examined the global gene expression profiles in Neurospora informed by previous RNA-Seq and microarray experiments/studies (Hurley *et al.* 2014; Wu *et al.* 2014; Logan *et al.* 2007). Using our analysis (*Materials and Methods*) we assigned a prediction interval ranking score (PIRS) to each gene. The resulting PIRS was minimized for genes that exhibit both flat expression as a function of time and low variance in observed expression at each time point (Figure 1). The gene ranks produced by our scoring metric were compared to rankings based on SD and relative SD (RSD) of expression using the Jaccard similarity coefficient (Levandowsky and Winter 1971) [Figure S1 and Figure S2 (Hintze and Nelson 1998)]. The rankings produced by our metric (PIRS) corresponded very well with SD and were noticeably more similar to RSD than random ranking. The majority of the difference between our method and RSD likely resulted from RSD’s preference for highly expressing genes, because the similarity score markedly improved when comparing the highest expressing quintile of genes under our scoring metric with the RSD results. A similar analysis was applied to the light-induced and QA-induced data sets with a few differences (Figure 2 and Figure 3). The selection criteria for nonlight-induced genes and non-QA-induced genes were identical to those for noncircadian genes except that the removal of noncircadian genes and the ANOVA were omitted (due to lack of replicate datasets).

For both the circadian and QA induction datasets, we selected the top two genes in each quintile of expression (Figure 1 and Figure 3). Because only relative expression data were available for the light-induction time course, we have reported the top 10 scoring genes overall (Figure 2). The top ranking genes in each quintile outperformed the average gene, whereas the top three quintiles were markedly higher than the average gene [Figure S3 (Scott 1992)]. In all cases, the genes selected by our metric show flat expression with low variation, whereas genes that rank poorly show high variation both within and between time points (compare Figure 1, Figure 2, and Figure 3 to Figure S4). The average FPKM values of the noncircadian genes ranged from log_10_ 0.087 to log_10_ 3.16, whereas the non-QA-induced genes ranged from log_10_ −1.30 to log_10_ 1.49, giving a wide variety of expression levels from which to choose from when selecting the appropriate reference gene. Not surprisingly, of the genes with predicted function, classifications included ribosomal, cytoskeletal, or protease genes, all proteins that could logically be expected to be housekeeping genes.

**Figure 4:**
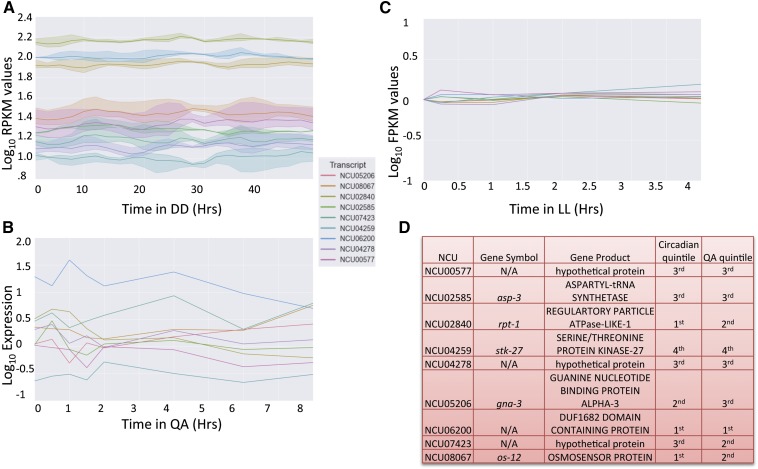
Least variably expressed genes for circadian RT-PCR in Neurospora. (A–C) A graphic representation of the log_10_ expression values from the (A) circadian, (B) quinic acid, and (C) light-induction data sets for the nine Neurospora genes in each category that were reported as the most stably expressed according to pareto optimization and normalized summed PIRS. (D) The chart reports the NCUs plotted in (A–C) with the quintile of expression associated with each gene from the circadian and QA time courses as well as the gene symbol and name for each of the NCUs reported. Gene symbols are from the Neurospora e-Compendium at Leeds.

As a way to identify genes that could be used as reference genes, we subjected all of the genes that were in our ENCAS set to pareto optimization to select nondominated genes across the three data sets. Pareto optimization is a technique for multi-objective optimization that aims to find the set of all choices that might be considered optimal under any of the possible combinations of weightings of individual scores (Pareto 1906). The algorithm rejects any choice for which another choice is available with superior scores under every metric. Genes for which there is an alternative gene with superior scores under all available metrics are termed "dominated" and genes for which any scoring metric exists under which they are optimal are termed "nondominated." This produced a list of 27 genes from the three data sets. Because it was possible that some of the genes on the pareto plane represent an optimal combination of PIRS for one experiment individually as opposed to all experiments in combination, we then compared these 27 genes to the summed normalized PIRS scores (Figure 4). We selected the overlap between the top 10 summed normalized scores and the pareto optimized set, leaving us with nine genes as good candidates for reference. These nine genes spanned the top four quintiles of expression (Figure 4), suggesting genes buffered against variation are not specific to a given level of expression except at the limits of detection (Figure S3).

### Generation of a genome-wide RT-PCR primer data set

**Figure 5:**
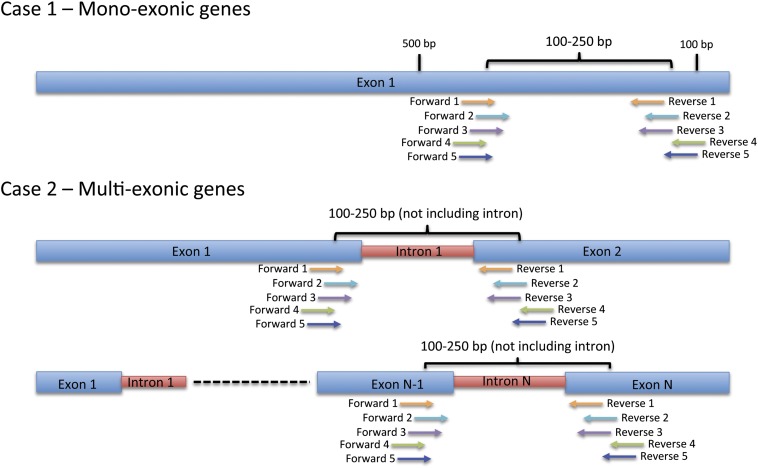
Criteria for the selection of RT-PCR primers. A diagrammatic representation of the rules used for primer design. In all cases, the primers are designed to create fragments between 100 and 250 bp in length. In the case of genes with no introns (mono-exonic genes), five unique primer pairs are designed to fall between 500 and 100 bp from the end of the gene. In the case of a gene with one intron, the primer pairs are designed to exclude the intron. In the case of genes with more than one intron, the primer pairs are designed to exclude the last intron in the gene, no matter how many introns there are.

Although there are many ways to analyze and validate mRNA levels and consistency between any two methods constitutes validation, it has become common practice in Neurospora to test mRNA steady-state levels using RT-PCR (Hurley *et al.* 2012, 2014; Wang *et al.* 2014a; Sancar *et al.* 2012; Hong *et al.* 2014; Hutchison *et al.* 2009). To analyze mRNA, RT-PCR primers are generally designed for each gene by hand, a time-consuming process when done on a large scale. To streamline this process, a catalog of RT-PCR primers was created for each gene designated in Neurospora by the Broad database. To achieve this we created a program that would design five primer sets for each gene based on the standard RT-PCR primer criteria (see *Materials and Methods*). In addition, each gene should have five primer pair sets that are not identical to one another. In the case of mono-exonic genes, the primer pairs were all selected from sequence in the last 500 base pairs of the gene (Figure 5). In the case of multi-exonic genes with a single intron, the primer pairs were designed to encompass the intron; in multi-exonic genes with multiple introns, the primer pairs were designed to encompass the last intron in the gene (Figure 5).

Using our program we were able to successfully design primers for 10,798 (99.9%) of the 10,802 ORFs predicted by Assembly 12 of the Neurospora genome (www.broadinstitute.org/annotation/genome/neurospora/GenomeDescriptions.html#NC12; http://fungidb.org/fungidb/; http://www.ncbi.nlm.nih.gov/genome/?term=txid367110) (Stajich *et al.* 2012) (File S1). The outline of the database can be seen in Table 1. For each transcript, the number of primer pairs that was designed is noted. In a few cases (five total), fewer than five unique primer pairs were designed; in theory, this is due to the limitations of the gene sequence and the parameters that were set. Each primer pair has a penalty score associated with it and that score, assigned by Primer3, represents how close the primer pair comes to the optimum conditions set by the program. The lower the score, the better the primer pair. The pair with the lowest score is ordered first, the second lowest score is second, and this pattern continues through to the fifth pair.

**Table 1:** Setup of the RT-PCR primer data set

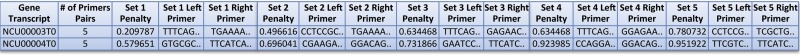

An example of the contents of the RT-PCR primer data set. Each transcript in Neurospora is listed with five primer pairs. Each primer pair is assigned a penalty score by Primer3 that represents the strength of the primer pair: the lower the score, the closer the primer pair is to the required/enforced criteria.

### Analyzing the generated RT-PCR primers

To confirm that the primers generated by our program were capable of producing valid RT-PCR products, we tested 49 genes with three or more of the generated primer pairs (Table 2). In our work, transcripts with low abundance generally give rise to highly variable RT-PCR estimates (Table 2). As a standard, we decided C_t_ values had to be below 30 to consider the cDNA target as being reliably detected by RT-PCR. We noted that genes that we classified as significantly amplified had an average Primer3 primer score of 0.4, whereas genes that were classified as not significantly amplified had an average Primer3 primer score of 0.9, suggesting that there is a correlation between Primer3 primer score and the successful amplification of PCR products.

**Table 2:** Designed RT-PCR primers detect mRNAs in Neurospora

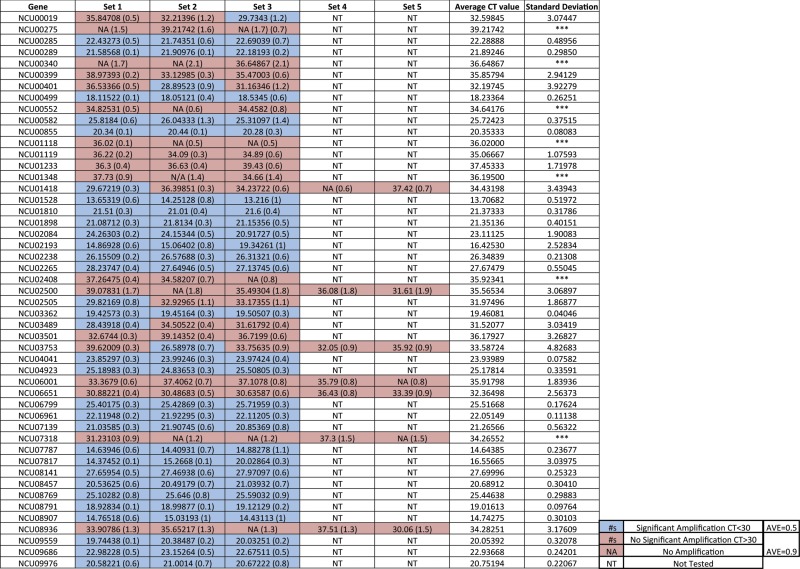

A chart of C_t_ values from primer pairs from the RT-PCR primer data set tested using RT-PCR with the Primer3 primer scores noted next to the C_t_ values in parentheses. Scores represent a single biological replicate. Average C_t_ values are an average of all samples that had a readable C_t_ score, whereas SDs of C_t_ values are only presented for genes that had three or more readable C_t_ values in the analysis. The average value of the Primer3 primer scores of gene with or without significant amplification is noted next to the key. Blue boxed numbers represent significant amplification C_t_ < 30; red boxed numbers represent no significant amplification C_t_ > 30; red boxed NA, amplification; NT, not tested.

Of the 49 genes analyzed, 34 (69%) returned a C_t_ value under 30 in at least one of the primer pairs (Table 2), meaning that these primer pairs were viable for use in RT-PCR studies. For medium to highly expressed genes with an average C_t_ value under 28, all of the primer pairs (28) were successfully able to return C_t_ values under 30. The remaining 15 (31%) genes had C_t_ values over 30 across the three primer pairs, indicating that they were very poorly expressed. For five of the 15 genes that had C_t_ values over 30, we tested the remaining two primer sets to see if we could obtain a reliable signal. None of the genes tested were able to generate meaningful C_t_ values, suggesting that genes that were not detected by our designed primers are either in extremely low abundance under the conditions tested or poor candidates for RT-PCR analysis rather than being an inherent program primer design failure. Furthermore, when comparing the average C_t_ values of all the tested primer pairs for genes with C_t_ values below 30 to the average C_t_ values of test primer pairs for genes with C_t_ values above 30, the SD of the average C_t_ value was strikingly lower in genes with C_t_ values below 30 than those genes with C_t_ values over 30 (Table 2) (*i.e.*, compare NCU06651 to NCU6799). The average SD for genes where all primer pairs reported a C_t_ values below 30 was 0.52 (this calculation does not include genes where some of the primer pairs had C_t_ values above 30), whereas the average SD of those genes with a C_t_ value above 30 (this calculations includes only the genes where a SD could be determined) was 2.45.

## Discussion

Finding a functional and truly constitutive “housekeeping” gene to serve as a reference gene in RT-PCR has often been difficult because changing cell or experimental conditions have been shown to have a large influence on gene expression levels (Schmittgen and Zakrajsek 2000), *i.e.*, reference genes tend to change expression levels under different conditions. This problem is augmented in circadian biology because many commonly used RT-PCR reference genes have been shown to be regulated by the circadian clock (*e.g.*, GAPDH) (Shinohara *et al.* 1998). Here, we report a partially validated genome-wide set of RT-PCR primers for Neurospora that reliably reports mRNA levels for all moderately to highly expressed genes and for 70% of all genes. There are many reasons for a less than perfect reporter rate, the most likely being that we only investigated the mRNA levels in senescent hyphal growth in a single type of growth medium. If we tracked mRNA extracted from a number of different stages of the Neurospora life cycle in a variety of different nutrient conditions, it seems likely that a greater percentage of gene targets would be more highly expressed and could be identified by our primers. In addition, we did not discern if the mRNAs investigated by this study were known to be poor candidates for RT-PCR for some unknown reason (*i.e.*, high levels of secondary structure). To develop manually a single RT-PCR primer sequence that meets all of the standards set forth in our criteria takes approximately 5–10 min, suggesting that these predesigned primers may afford considerable time-savings.

Circadian rhythms present a unique challenge for those investigating mRNA levels via RT-PCR. Because as much as 40% of the genome could be rhythmic at the mRNA level (Hurley *et al.* 2014), finding genes that show little change in expression over the circadian day to serve as reference genes has been be difficult. Even well-known housekeeping genes that are used regularly as RT-PCR reference genes have been shown to cycle over circadian time (Shinohara *et al.* 1998). In this work, we used an in-depth investigation into mRNA expression levels to identify genes that demonstrated little change at the steady-state mRNA level over circadian time, light induction, and QA response (Hurley *et al.* 2014). We identified nine potential candidates that could be used as controls for any experiments involving long-term culture, with mRNA levels that are minimally variable across multiple modes of regulation. Many of the genes identified have orthologs in higher eukaryotes (Figure 1, Figure 2, Figure 3, and Figure 4 and Figure S1) and therefore have the potential to be useful to other species that serve as model organisms.

## 

## Supplementary Material

Supporting Information
