# Supplementary material for: A Tool Set for the Genome-Wide Analysis of Neurospora crassa by RT-PCR
Source: G3 (Bethesda). 2015 Aug 6;5(10):2043–9. doi: 10.1534/g3.115.019141 (PMC4592987; doi:10.1534/g3.115.019141)
Supplement: Supporting Information [file supp_5_10_2043__index.html]

A Tool Set for the Genome-Wide Analysis of Neurospora crassa by RT-PCR — Supporting Information 

# A Tool Set for the Genome-Wide Analysis of *Neurospora crassa* by RT-PCR

## Supporting Information for Hurley *et al.*, 2015

**Files in this Data Supplement:**

- Supporting Information - Figures S1-S4, Table S1, and File S1 (PDF, 1 MB)
- Figure S1 - Optimal reference genes for RT-PCR in *Neurospora* identified by RSD. (PDF, 429 KB)
- Figure S2 - PIRS analysis compares favorably with other methods. (PDF, 218 KB)
- Figure S3 - PIRS analysis identifies genes that show stable expression under different experimental conditions. (PDF, 227 KB)
- Figure S4 - Least stably expressed genes for circadian RT-PCR in *Neurospora*. (PDF, 512 KB)
- Table S1 - The RT-PCR primer Catalogue. For every transcript in *Neurospora*, 5 primers are listed. A penalty score is assigned to each primer pair, which, according to Primer3, represents the strength of the primer pair, with the lower scores highlighting the better primer pairs. (.xlsx, 1 MB)
- File S1 - Supplemental Catalogue 1. A detailed list of primers generated including their source sequences. (.zip, 20 MB)
