# Supplementary material for: A Tool Set for the Genome-Wide Analysis of Neurospora crassa by RT-PCR
Source: G3 (Bethesda). 2015 Aug 6;5(10):2043–9. doi: 10.1534/g3.115.019141 (PMC4592987; doi:10.1534/g3.115.019141)
Supplement: Supporting Information [file supp_g3.115.019141_019141SI.pdf]

G3: Genes | Genomes | Genetics

A tool set for the genome wide analysis of *Neurospora crassa* by RT-PCR

Jennifer H. Hurley[1*], Arko Dasgupta[1*], Peter Andrews[2], Alexander M. Crowell[1], Carol Ringelberg[1], Jennifer J. Loros[3], Jay C. Dunlap[1]§

[1]Department of Genetics, Geisel School of Medicine, Hanover, NH 03755

[2]Institute for Biomedical Informatics, Perelman School of Medicine, Philadelphia, Pa 19104

[3]Department of Biochemistry, Geisel School of Medicine, Hanover, NH 03755

[*] These authors contributed equally to this work

§ Correspondence: jay.c.dunlap@dartmouth.edu

Jay C. Dunlap

Geisel School of Medicine at Dartmouth

HB 7400

Hanover NH 03755

Office: 603-650-1907

Phone: 603-650-1108

Fax: 603-650-1233

jay.c.dunlap@dartmouth.edu

DOI: 10.1534/g3.115.019141

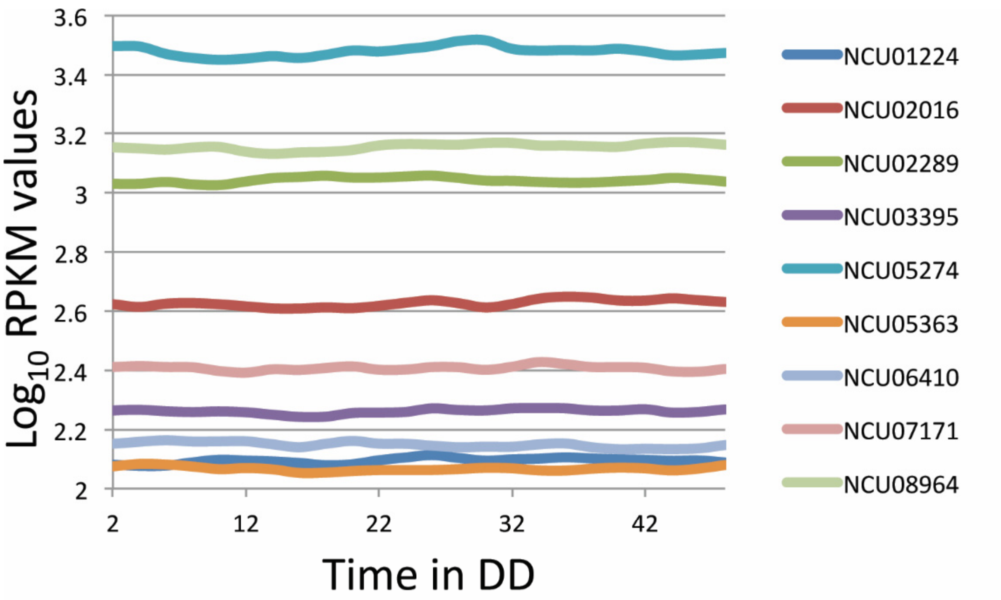

NCU Number	Gene Symbol	Gene Product
NCU01224	*rpt-2*	REGULARTORY PARTICLE ATPase-LIKE-2
NCU02016	N/A	hypothetical protein
NCU02289	*uce-6*	UBIQUITIN-CONJUGATING ENZYME E2
NCU03395	*vma-6*	VACUOLAR MEMBRANE ATPase-6
NCU05274	*eif5A*	EUKARYOTIC INITATION FACTOR 5A
NCU05363	*rpt-6*	26S PROTEASE REGULATORY SUBUNIT 8
NCU06410	*gtp-13*	GTP-BINDING PROTEIN YPT52
NCU07171	*arp2*	ACTIN-RELATED PROTEIN 2
NCU08964	*crp-43*	60S RIBOSOMAL PROTEIN L10

Figure S1 Optimal reference genes for RT-PCR in *Neurospora* identified by RSD. A graphical representation of the $\log_{10}$ of FPKM values from the RNA-Seq data set for the nine *Neurospora* genes for which the standard deviation of the $\log_{10}$FPKM values was less than 0.5% of the average of the $\log_{10}$FPKM values. The chart below reports the gene name as well as gene symbol for each of the NCUs reported above. Gene symbols are from the *Neurospora* e-Compendium at Leeds.

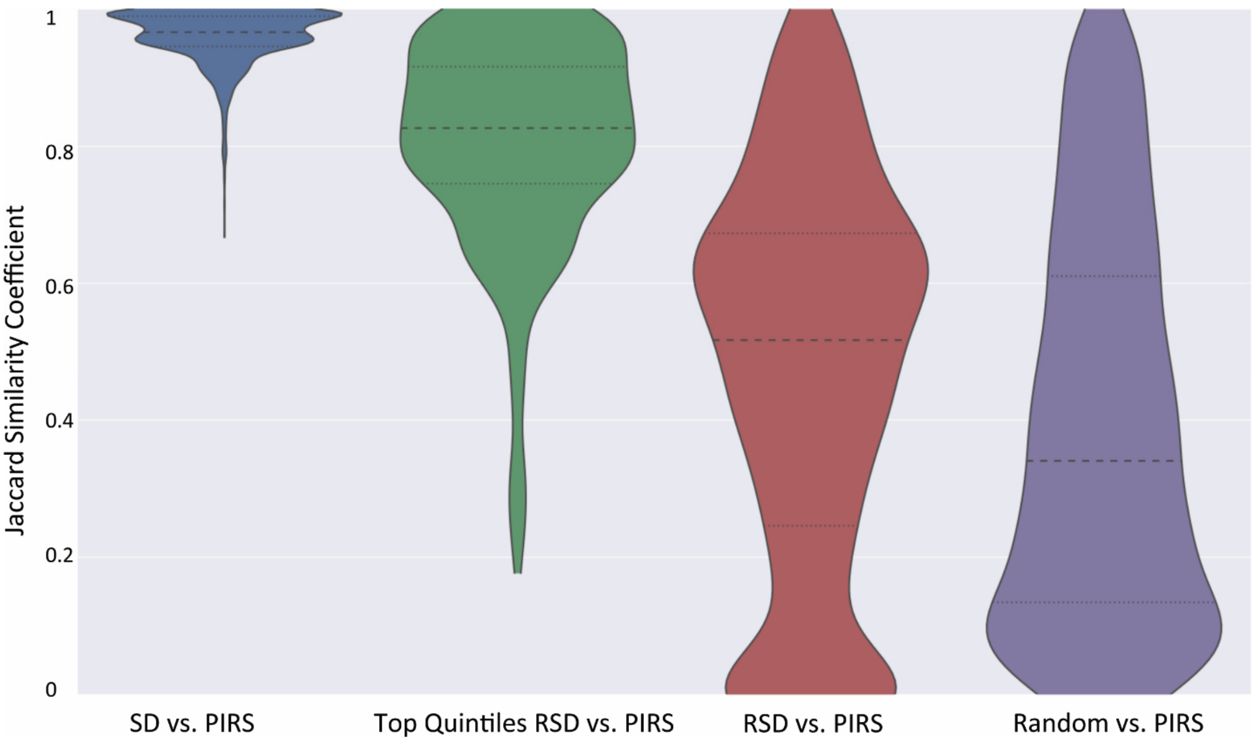

Figure S2 PIRS analysis compares favorably with other methods. Violin plots (Hintze and Nelson 1998) of the distributions of Jaccard Similarity Coefficients (JSC) (Levandowsky and Winter 1971) comparing ranking methods on the circadian dataset for lists of the top n genes under each method where n ranges from zero to all genes. Width of each plot at a given JSC represents the number of occurrences of that score when comparing the two ranking methods. The higher the JSC the more is the overlap in the rankings, and the wider the plot at a given JSC the more is the degree of overlap; i.e. in the case of SD, many of the genes that are considered to be constitutively expressed are also considered to be constitutively expressed in PIRS.

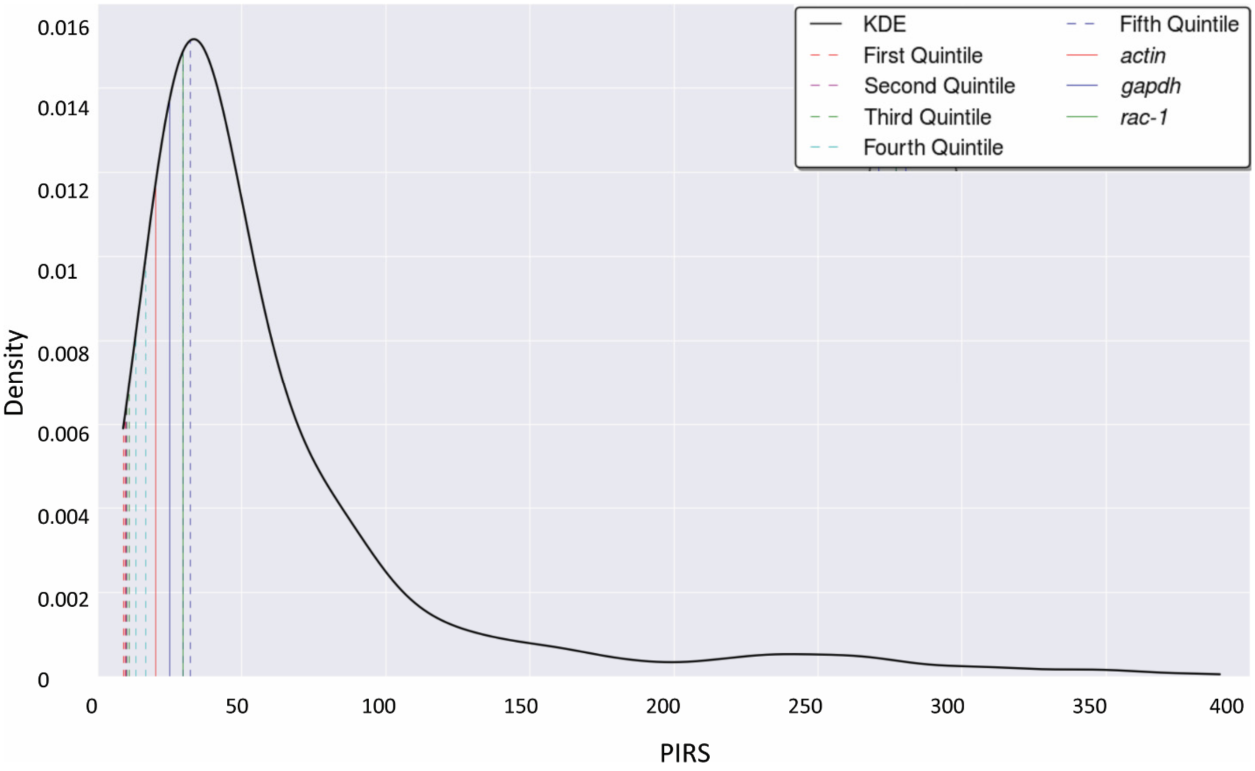

Figure S3 PIRS analysis identifies genes that show stable expression under different experimental conditions. Kernel Density Estimate (Scott 1992) of the distribution of PIRS values for all genes in the circadian dataset. The higher the density value, the more frequent the PIRS.

The PIRS value for the top two genes in each quintile is labeled using vertical dashed lines and commonly used reference genes are labeled using solid lines. Note that three commonly used “invariant standards for normalization of gene expression”, *actin*, *gapdh*, and *rac*-1, are actually as or more variable than 50% of genes.

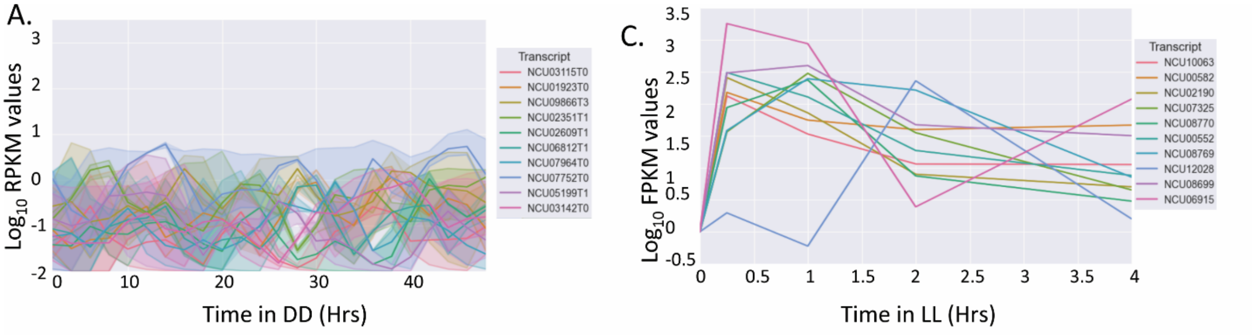

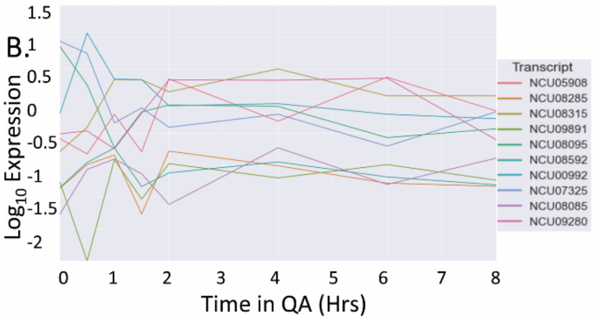

D.

Circadian Genes	Light Induced genes	QA induced genes
NCU01923	NCU00552	NCU00992
NCU02351	NCU00582	NCU05908
NCU02609	NCU02190	NCU07325
NCU03115	NCU06915	NCU08085
NCU03142	NCU07325	NCU08095
NCU05199	NCU08699	NCU08285
NCU06812	NCU08769	NCU08315
NCU07752	NCU08770	NCU08592
NCU07964	NCU10063	NCU09280
NCU09866	NCU12028	NCU09891

Figure S4 Least stably expressed genes for circadian RT-PCR in *Neurospora*. A.-C. A graphic representation of the $\log_{10}$ expression values from the A. circadian, B. quinic acid and C. light-induction data sets for the ten *Neurospora* genes in each category that were reported as the least stably expressed according to our analysis. D. The chart reports the NCUs plotted in A-C.

Table S1 The RT-PCR primer Catalogue. For every transcript in *Neurospora*, 5 primers are listed. A penalty score is assigned to each primer pair, which, according to Primer3, represents the strength of the primer pair, with the lower scores highlighting the better primer pairs.

Available for download as an Excel file at www.g3journal.org/lookup/suppl/doi:10.1534/g3.115.019141/-/DC1

File S1

Supplemental Catalogue 1

A detailed list of primers generated including their source sequences

Available for download as a .zip file at www.g3journal.org/lookup/suppl/doi:10.1534/g3.115.019141/-/DC1
