# Supplementary material for: A Tool Set for the Genome-Wide Analysis of Neurospora crassa by RT-PCR
Source: G3 (Bethesda). 2015 Aug 6;5(10):2043–9. doi: 10.1534/g3.115.019141 (PMC4592987; doi:10.1534/g3.115.019141)
Supplement: Supporting Information [file supp_g3.115.019141_FigureS1.pdf]

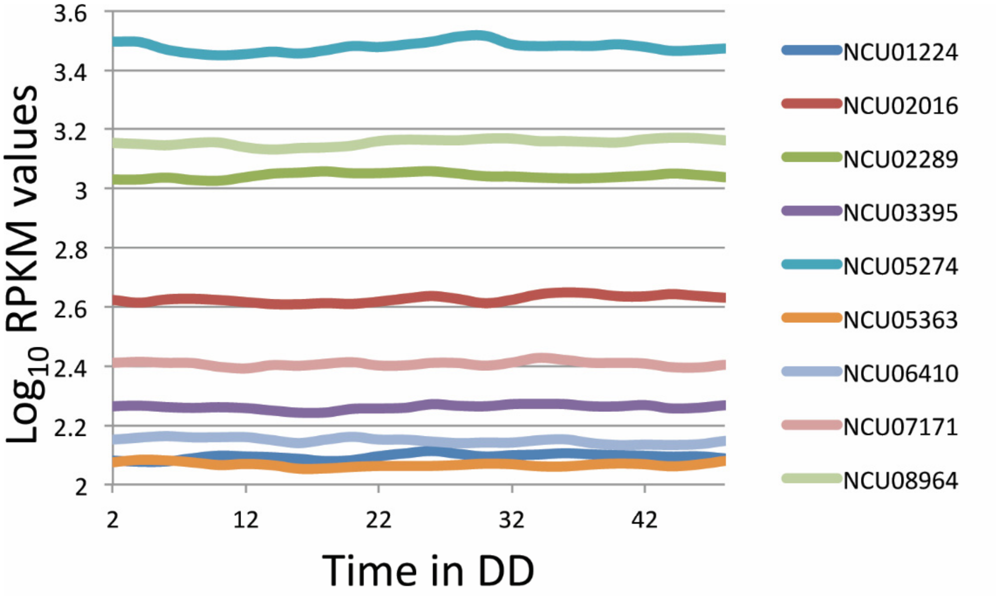

NCU Number	Gene Symbol	Gene Product
NCU01224	*rpt-2*	REGULARTORY PARTICLE ATPase-LIKE-2
NCU02016	N/A	hypothetical protein
NCU02289	*uce-6*	UBIQUITIN-CONJUGATING ENZYME E2
NCU03395	*vma-6*	VACUOLAR MEMBRANE ATPase-6
NCU05274	*eif5A*	EUKARYOTIC INITATION FACTOR 5A
NCU05363	*rpt-6*	26S PROTEASE REGULATORY SUBUNIT 8
NCU06410	*gtp-13*	GTP-BINDING PROTEIN YPT52
NCU07171	*arp2*	ACTIN-RELATED PROTEIN 2
NCU08964	*crp-43*	60S RIBOSOMAL PROTEIN L10

Figure S1 Optimal reference genes for RT-PCR in *Neurospora* identified by RSD. A graphical representation of the $\log_{10}$ of FPKM values from the RNA-Seq data set for the nine *Neurospora* genes for which the standard deviation of the $\log_{10}$FPKM values was less than 0.5% of the average of the $\log_{10}$FPKM values. The chart below reports the gene name as well as gene symbol for each of the NCUs reported above. Gene symbols are from the *Neurospora* e-Compendium at Leeds.
