# Supplementary material for: A Tool Set for the Genome-Wide Analysis of Neurospora crassa by RT-PCR
Source: G3 (Bethesda). 2015 Aug 6;5(10):2043–9. doi: 10.1534/g3.115.019141 (PMC4592987; doi:10.1534/g3.115.019141)
Supplement: Supporting Information [file supp_g3.115.019141_FigureS2.pdf]

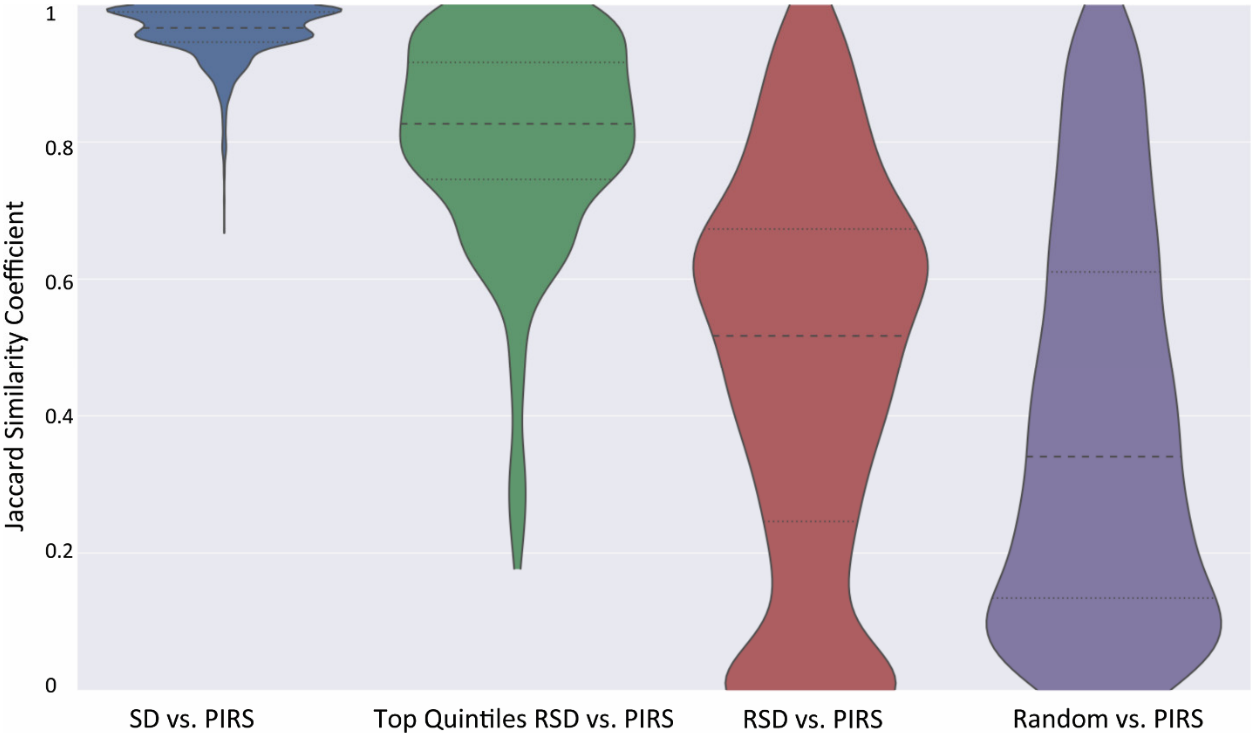

Figure S2 PIRS analysis compares favorably with other methods. Violin plots (Hintze and Nelson 1998) of the distributions of Jaccard Similarity Coefficients (JSC) (Levandowsky and Winter 1971) comparing ranking methods on the circadian dataset for lists of the top n genes under each method where n ranges from zero to all genes. Width of each plot at a given JSC represents the number of occurrences of that score when comparing the two ranking methods. The higher the JSC the more is the overlap in the rankings, and the wider the plot at a given JSC the more is the degree of overlap; i.e. in the case of SD, many of the genes that are considered to be constitutively expressed are also considered to be constitutively expressed in PIRS.
