# Supplementary material for: A Tool Set for the Genome-Wide Analysis of Neurospora crassa by RT-PCR
Source: G3 (Bethesda). 2015 Aug 6;5(10):2043–9. doi: 10.1534/g3.115.019141 (PMC4592987; doi:10.1534/g3.115.019141)
Supplement: Supporting Information [file supp_g3.115.019141_FigureS3.pdf]

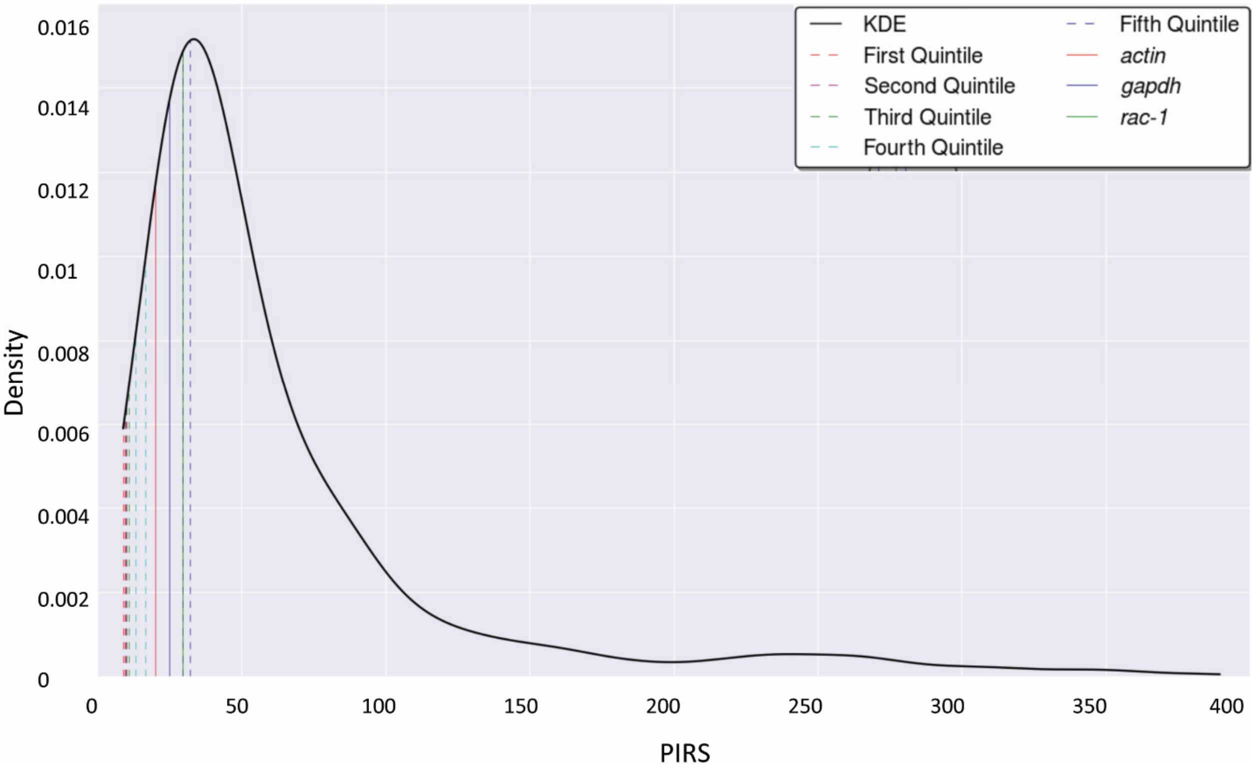

Figure S3 PIRS analysis identifies genes that show stable expression under different experimental conditions. Kernel Density Estimate (Scott 1992) of the distribution of PIRS values for all genes in the circadian dataset. The higher the density value, the more frequent the PIRS.

The PIRS value for the top two genes in each quintile is labeled using vertical dashed lines and commonly used reference genes are labeled using solid lines. Note that three commonly used "invariant standards for normalization of gene expression", *actin*, *gapdh*, and *rac*-1, are actually as or more variable than 50% of genes.
