# Supplementary material for: A Tool Set for the Genome-Wide Analysis of Neurospora crassa by RT-PCR
Source: G3 (Bethesda). 2015 Aug 6;5(10):2043–9. doi: 10.1534/g3.115.019141 (PMC4592987; doi:10.1534/g3.115.019141)
Supplement: Supporting Information [file supp_g3.115.019141_FigureS4.pdf]

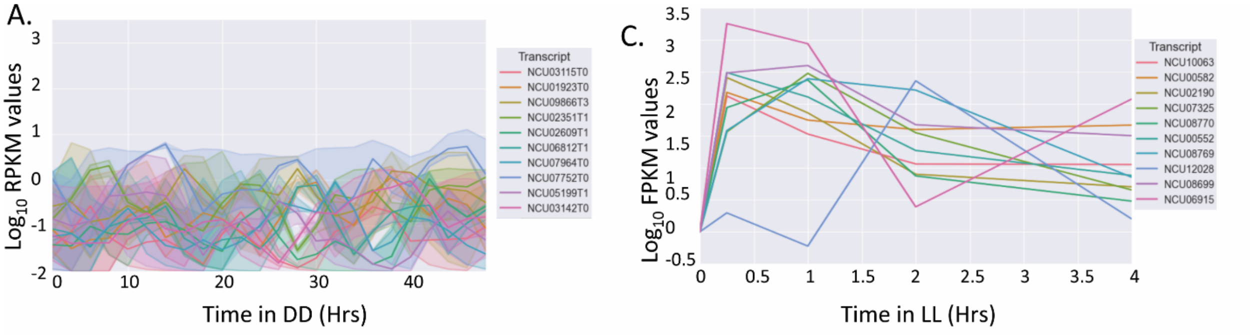

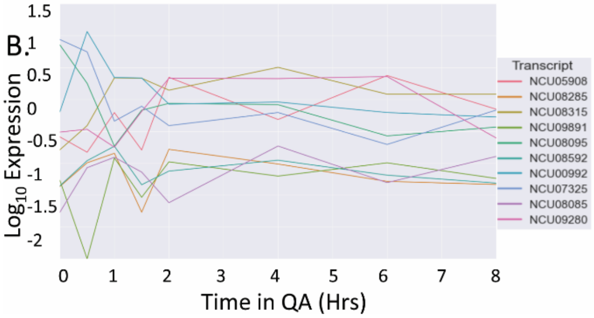

D.

Circadian Genes	Light Induced genes	QA induced genes
NCU01923	NCU00552	NCU00992
NCU02351	NCU00582	NCU05908
NCU02609	NCU02190	NCU07325
NCU03115	NCU06915	NCU08085
NCU03142	NCU07325	NCU08095
NCU05199	NCU08699	NCU08285
NCU06812	NCU08769	NCU08315
NCU07752	NCU08770	NCU08592
NCU07964	NCU10063	NCU09280
NCU09866	NCU12028	NCU09891

Figure S4 Least stably expressed genes for circadian RT-PCR in *Neurospora*. A.-C. A graphic representation of the log_{10} expression values from the A. circadian, B. quinic acid and C. light-induction data sets for the ten *Neurospora* genes in each category that were reported as the least stably expressed according to our analysis. D. The chart reports the NCUs plotted in A-C.
