# Supplementary material for: A Tool Set for the Genome-Wide Analysis of Neurospora crassa by RT-PCR
Source: G3 (Bethesda). 2015 Aug 6;5(10):2043–9. doi: 10.1534/g3.115.019141 (PMC4592987; doi:10.1534/g3.115.019141)
Supplement: Supporting Information [file supp_g3.115.019141_FileS1.zip › neurospora_crassa_or74a_12_primers.html]

Primer report for: neurospora\_crassa\_or74a\_12


# Primer report for: neurospora\_crassa\_or74a\_12

| gene transcript | # of exons | # of primers pairs | input file | output file | PRIMER\_PAIR\_0\_PENALTY | PRIMER\_LEFT\_0 | PRIMER\_RIGHT\_0 | PRIMER\_PAIR\_1\_PENALTY | PRIMER\_LEFT\_1 | PRIMER\_RIGHT\_1 | PRIMER\_PAIR\_2\_PENALTY | PRIMER\_LEFT\_2 | PRIMER\_RIGHT\_2 | PRIMER\_PAIR\_3\_PENALTY | PRIMER\_LEFT\_3 | PRIMER\_RIGHT\_3 | PRIMER\_PAIR\_4\_PENALTY | PRIMER\_LEFT\_4 | PRIMER\_RIGHT\_4 |
| --- | --- | --- | --- | --- | --- | --- | --- | --- | --- | --- | --- | --- | --- | --- | --- | --- | --- | --- | --- |
| NCU00003T0 | 3 | 5 | NCU00003T0\_input.txt | NCU00003T0\_output.txt | 0.209787 TTTCAGGATGAGTCTCGGCG | TGAAAAGGGGAGAACGAGCC | 0.496616 CCTCCGCCGTATCCATATCC | TGAAAAGGGGAGAACGAGCC | 0.634468 TTTCAGGATGAGTCTCGGCG | GAGAACGAGCCATGACAGGG | 0.634468 TTTCAGGATGAGTCTCGGCG | GGAGAACGAGCCATGACAGG | 0.780732 CCTCCGCCGTATCCATATCC | TCGCTGAAAAGGGGAGAACG | | | | | |
| NCU00004T0 | 2 | 5 | NCU00004T0\_input.txt | NCU00004T0\_output.txt | 0.579651 GTGCGCGTCATTGAGAATCC | TTCATCAGGCCCTGGATTCC | 0.696041 CGAAGAAGCTGTTGCAGACC | GGACAGGGCTGTATTGGAGG | 0.731866 GAATCCCATTCGTCCAGGGG | TTCATCAGGCCCTGGATTCC | 0.923985 CCAGGAGGAAGCGAAGAAGC | GGACAGGGCTGTATTGGAGG | 0.951922 TTCGTCCAGGGGAAAAGTCC | TTCATCAGGCCCTGGATTCC | | | | | |
| NCU00005T0 | 1 | 5 | NCU00005T0\_input.txt | NCU00005T0\_output.txt | 0.073798 TACGGTGTTGGGGATTTGGG | GACCTCTTAGCCCCAACACC | 0.286136 TGGTATACGGTTGCCAGTGG | GACCTCTTAGCCCCAACACC | 0.430655 AGTGGACTTACGGTGTTGGG | GACCTCTTAGCCCCAACACC | 0.486264 GCGCTGGAGGAGTATTTTGC | ACGGCTTATTGGAGCAGTCC | 0.498917 GTGTTGGGGCTAAGAGGTCG | ACGGCTTATTGGAGCAGTCC | | | | | |
| NCU00006T0 | 6 | 5 | NCU00006T0\_input.txt | NCU00006T0\_output.txt | 0.769317 ATGTAGGAGCAGCGAACTGG | TGAAATTCACAGCCTGCACG | 0.776129 TGATCCCCAAGCATAGTGGC | TGAAATTCACAGCCTGCACG | 0.918381 TGATCCCCAAGCATAGTGGC | GATGTTGGATGGGTGAAGCG | 1.195578 TGATCCCCAAGCATAGTGGC | CTTGTCATGCGTGATGGAGG | 1.250297 CGACGTATGTAGGAGCAGCG | TGAAATTCACAGCCTGCACG | | | | | |
| NCU00006T1 | 5 | 5 | NCU00006T1\_input.txt | NCU00006T1\_output.txt | 0.769317 ATGTAGGAGCAGCGAACTGG | TGAAATTCACAGCCTGCACG | 0.776129 TGATCCCCAAGCATAGTGGC | TGAAATTCACAGCCTGCACG | 0.918381 TGATCCCCAAGCATAGTGGC | GATGTTGGATGGGTGAAGCG | 1.195578 TGATCCCCAAGCATAGTGGC | CTTGTCATGCGTGATGGAGG | 1.250297 CGACGTATGTAGGAGCAGCG | TGAAATTCACAGCCTGCACG | | | | | |
| NCU00007T0 | 1 | 5 | NCU00007T0\_input.txt | NCU00007T0\_output.txt | 0.710625 TCATCACCGTCGGAATGTCC | TGCAGACAGAACTTGGCAGG | 0.766049 GACACCGTCTCCTTGACACG | TTTGATCCTCGCTAGACGCC | 0.897462 CGAGAAGCGACTCCTCAACG | TTTGATCCTCGCTAGACGCC | 0.907474 GACACCGTCTCCTTGACACG | TCTCACTTCTTCCTTCCGCG | 0.911263 GACACCGTCTCCTTGACACG | GCTAGACGCCGAAACAATGC | | | | | |
| NCU00008T0 | 3 | 5 | NCU00008T0\_input.txt | NCU00008T0\_output.txt | 0.073356 TCCTGGGAATGGAAAAGCCC | GTAGTGGCGCAGGTAGATCC | 0.204844 TCACCTACATCGGTCTTGCG | GTAGTGGCGCAGGTAGATCC | 0.344930 TCACCTACATCGGTCTTGCG | GAGTCTCCCAGTTGAGCTCG | 0.493700 TCACCTACATCGGTCTTGCG | AGAGGCTGCACTTGTACTGC | 0.563576 TCACCTACATCGGTCTTGCG | GCTGAGTCTCCCAGTTGAGC | | | | | |
| NCU00009T0 | 2 | 5 | NCU00009T0\_input.txt | NCU00009T0\_output.txt | 0.884866 AGGGGGATGGGTGAGTATGG | AAGAAGTCTTGGGGTTGGGG | 1.018870 AGGGGGATGGGTGAGTATGG | GGTTGGGGTAGTGGTAGTGG | 1.074624 CAAAAGGGAGACAGGGGTGC | TGCCGATTGAGCATGAGAGG | 1.143774 CAAAAGGGAGACAGGGGTGC | GCATTTCGAGTTGATGGCGG | 1.163490 ATGCTAGTCGGAAAAGGGGG | GGTTGGGGTAGTGGTAGTGG | | | | | |
| NCU00010T0 | 4 | 5 | NCU00010T0\_input.txt | NCU00010T0\_output.txt | 1.125431 CTGACCCAGCAACGTAAACC | ACCCAGATCGATGGTGTTGG | 1.187639 CTGACCCAGCAACGTAAACC | TGGTTCACTCTGTCAGCTCG | 1.533133 CGATATTCGGGCTGTGTTCC | TGGTTCACTCTGTCAGCTCG | 1.601503 CTGACCCAGCAACGTAAACC | CAGATCGATGGTGTTGGTGC | 1.784874 CTGACCCAGCAACGTAAACC | AGTGGTGGAGCAAGATACCC | | | | | |
| NCU00011T0 | 2 | 5 | NCU00011T0\_input.txt | NCU00011T0\_output.txt | 0.288929 GCCGAACAACAGGCTTAAGC | GAACCTCGAACCTCGTCTCG | 0.359224 GCCGAACAACAGGCTTAAGC | CGATCCTCCAATGTCCGACC | 0.432191 GCCGAACAACAGGCTTAAGC | TCAAGAGGCCATGGAAACGG | 0.479666 GCCGAACAACAGGCTTAAGC | TCATCGAAGCGCAAATGTCG | 0.493226 GCCGAACAACAGGCTTAAGC | CGCAAATGTCGCATCGATCC | | | | | |
| NCU00013T0 | 2 | 5 | NCU00013T0\_input.txt | NCU00013T0\_output.txt | 0.428244 TGCTGATTACCCTGGTGTCG | TCACGGCAAAGGTTTGTTGC | 0.430157 AAGCCACCGGACCTAAAACC | TCACGGCAAAGGTTTGTTGC | 0.633069 TGCTGATTACCCTGGTGTCG | TTTGTTGCGAAGGCTTCACC | 0.634982 AAGCCACCGGACCTAAAACC | TTTGTTGCGAAGGCTTCACC | 1.065945 TGCTGATTACCCTGGTGTCG | ATGCTGACCGTCATCATGGC | | | | | |
| NCU00014T0 | 2 | 5 | NCU00014T0\_input.txt | NCU00014T0\_output.txt | 0.556547 GCGATCGGCCATTAATTCGG | TTACATCACCTCCGTTGGCG | 0.569673 GGTCTTGATCGAAGAGGGCC | TTACATCACCTCCGTTGGCG | 0.695718 GCGATCGGCCATTAATTCGG | TATCGTGGCGCACATCTTCC | 0.708843 GGTCTTGATCGAAGAGGGCC | TATCGTGGCGCACATCTTCC | 0.774074 GCGATCGGCCATTAATTCGG | TGATTCTGGACTGTCGTGGG | | | | | |
| NCU00015T0 | 2 | 5 | NCU00015T0\_input.txt | NCU00015T0\_output.txt | 0.783660 ATGTAGAAACCGTTGGCCCG | ATTCACCGGCTTCTTCCTCG | 0.854595 ATGTAGAAACCGTTGGCCCG | CAGGGCTACAGATTCACCGG | 0.854595 ATGTAGAAACCGTTGGCCCG | CCAGGGCTACAGATTCACCG | 0.977377 CTCACAGCTATGCTCGACCG | GTTTGACGCTGCGTTACACC | 1.059382 ATGTAGAAACCGTTGGCCCG | GTTTGACGCTGCGTTACACC | | | | | |
| NCU00017T0 | 2 | 5 | NCU00017T0\_input.txt | NCU00017T0\_output.txt | 0.556518 AGTACTGACTCGGATTCGCG | TTTGGTCTTGGGCTCTTGGG | 0.556588 AGTACTGACTCGGATTCGCG | CAGCTATGAAGAGGTGGGGC | 0.624738 AGTACTGACTCGGATTCGCG | ACATTCCAGGCGCATTTTGG | 0.766461 AGTACTGACTCGGATTCGCG | CAGGCGCATTTTGGTCTTGG | 1.047716 AGTACTGACTCGGATTCGCG | AATGCATACATTCCAGGCGC | | | | | |
| NCU00018T1 | 7 | 5 | NCU00018T1\_input.txt | NCU00018T1\_output.txt | 0.570119 CAGTCTTCCGCTAGCCTTGG | CCGAATTTGGAGGCAGTTGC | 0.571356 CTGAGGGTGGTGTTGAGAGC | TTGCTCTGAATCGACCCTGG | 0.641895 TCCCAGCAGATGAAGAACGC | TTGCTCTGAATCGACCCTGG | 0.779093 CAGTCTTCCGCTAGCCTTGG | CAAACACAAAGCCAGCGAGG | 0.842788 CAGTCTTCCGCTAGCCTTGG | AGTCAAACACAAAGCCAGCG | | | | | |
| NCU00018T0 | 6 | 5 | NCU00018T0\_input.txt | NCU00018T0\_output.txt | 0.287585 TGGATTCTATCGCCAAGGCC | CCTCATCAGGAAGGGGAACG | 0.358819 TGGATTCTATCGCCAAGGCC | TCTTCTTGGAGGTCATGCCG | 0.359273 GGTTCGGTGAGTCTGAGAGC | TCTTCTTGGAGGTCATGCCG | 0.415731 TGGATTCTATCGCCAAGGCC | GAGAATACCGAGACGACCCG | 0.429858 TCTGTTGGTGATGCTGGTGG | CCTCATCAGGAAGGGGAACG | | | | | |
| NCU00019T0 | 4 | 5 | NCU00019T0\_input.txt | NCU00019T0\_output.txt | 0.497129 CTGCCACTTCATGCCTTTGC | ACACACATATGGACAGCCCG | 1.187315 GCCACTTCATGCCTTTGCC | ACACACATATGGACAGCCCG | 1.204639 CTGCCACTTCATGCCTTTGC | CACACATATGGACAGCCCGG | 1.287732 ACAATCTGCCACTTCATGCC | ACACACATATGGACAGCCCG | 1.299029 TCATATCTTCACGGGCGTCG | ACTCATAGTGACTCGCATCGC | | | | | |
| NCU00021T0 | 3 | 5 | NCU00021T0\_input.txt | NCU00021T0\_output.txt | 0.422610 AACGATGGTCACGACGAAGG | TCATGATGGGATGAGCGAGC | 0.491129 GTTGTGCCATTGACATCGGC | TCATGATGGGATGAGCGAGC | 0.493511 GTTGTGCCATTGACATCGGC | AGAAGAGGAGGCAACGAAGC | 0.711258 GGTGTTGGGGAGAACGATGG | TCATGATGGGATGAGCGAGC | 0.831725 GGAGAACGATGGTCACGACG | TCATGATGGGATGAGCGAGC | | | | | |
| NCU00022T0 | 1 | 5 | NCU00022T0\_input.txt | NCU00022T0\_output.txt | 0.286702 CGGTAGTGCTAGGGTCAACG | TTTCTCCCCCGTAAATCGCC | 0.287851 GGGGAGAAACCGAAGAGACG | TGACGATCCGTTACCATGCC | 0.287898 ACGGTGTGAATACTGCTCGG | TGACGATCCGTTACCATGCC | 0.287901 CGGTAGTGCTAGGGTCAACG | CGTCTCTTCGGTTTCTCCCC | 0.428821 CGGTAGTGCTAGGGTCAACG | GTCTCTTCGGTTTCTCCCCC | | | | | |
| NCU00023T0 | 2 | 5 | NCU00023T0\_input.txt | NCU00023T0\_output.txt | 0.271814 GGGCTACCTTTACCGTACGG | ACGCCTGAATTGCAATGACG | 0.276002 ATGGCTGGGTCGTATTGTGG | ACGCCTGAATTGCAATGACG | 0.342043 TTTACCGTACGGCTGCTACC | ACGCCTGAATTGCAATGACG | 0.342043 TACCTTTACCGTACGGCTGC | ACGCCTGAATTGCAATGACG | 0.499496 TCGAGGGCATCTTCAAGAGC | CCCACAAGACCATGACAGGG | | | | | |
| NCU00024T1 | 4 | 5 | NCU00024T1\_input.txt | NCU00024T1\_output.txt | 0.217915 GCGACAACCGTAACAACACC | CCGAGTTGAAGAGGTAGGCC | 0.284127 ATGTCTACCTCAACAGCGGC | CCGAGTTGAAGAGGTAGGCC | 0.287256 ATGTCTACCTCAACAGCGGC | CGTACTTCCATCCGACCACC | 0.425021 GCGGCAAGATTTATGGGTGC | CCGAGTTGAAGAGGTAGGCC | 0.427099 GCGGCAAGATTTATGGGTGC | TTCTCGTTGGGCTCATAGGC | | | | | |
| NCU00024T0 | 3 | 5 | NCU00024T0\_input.txt | NCU00024T0\_output.txt | 0.217915 GCGACAACCGTAACAACACC | CCGAGTTGAAGAGGTAGGCC | 0.284127 ATGTCTACCTCAACAGCGGC | CCGAGTTGAAGAGGTAGGCC | 0.287256 ATGTCTACCTCAACAGCGGC | CGTACTTCCATCCGACCACC | 0.425021 GCGGCAAGATTTATGGGTGC | CCGAGTTGAAGAGGTAGGCC | 0.427099 GCGGCAAGATTTATGGGTGC | TTCTCGTTGGGCTCATAGGC | | | | | |
| NCU00025T0 | 3 | 5 | NCU00025T0\_input.txt | NCU00025T0\_output.txt | 0.501623 CTGGAGGGTCTGAATGACGG | TAGGGGTTCACTTCTGCAGC | 0.641414 CTGGAGGGTCTGAATGACGG | TGTCAAAGGGTCACGTTGGG | 0.711421 CAAAGTGCTTCCGCTTGAGC | TAGGGGTTCACTTCTGCAGC | 0.781257 CTGGAGGGTCTGAATGACGG | TTGAAGGGACGGACGTAAGG | 0.851213 CAAAGTGCTTCCGCTTGAGC | TGTCAAAGGGTCACGTTGGG | | | | | |
| NCU00026T0 | 1 | 5 | NCU00026T0\_input.txt | NCU00026T0\_output.txt | 0.209242 GATGGGCCAGACCTCAATCC | GTGTTTTGGTCCCGTCAAGC | 0.213677 GATGGGCCAGACCTCAATCC | AAGCCATCTCACAAGCTCCC | 0.273215 CGACATAACCACAAACCGCC | TTTCTGGAGGGTGTGTCACG | 0.276593 CGACATAACCACAAACCGCC | AGTGTCTGCAGGTTTCTGGG | 0.281006 AGAAACCCAGAAAGACGGCC | GTGTTTTGGTCCCGTCAAGC | | | | | |
| NCU00027T0 | 1 | 5 | NCU00027T0\_input.txt | NCU00027T0\_output.txt | 0.071732 GAAAGGGAGAGGGGAAACGG | TCTGCGCTTGGTTTCCTAGG | 0.071920 GAAAGGGAGAGGGGAAACGG | AGATGACTGCTCTGTGTGGC | 0.143897 ATTGATCGGTGGCAGTGAGG | TCTGCGCTTGGTTTCCTAGG | 0.144085 ATTGATCGGTGGCAGTGAGG | AGATGACTGCTCTGTGTGGC | 0.288175 ATGAAACCTGGGGTTGAGGC | TCTGCGCTTGGTTTCCTAGG | | | | | |
| NCU00028T1 | 5 | 5 | NCU00028T1\_input.txt | NCU00028T1\_output.txt | 0.436720 ATGAAACCTGGGGTTGAGGC | AGATGAGAGGTCGAGGGAGG | 0.501825 ATGAAACCTGGGGTTGAGGC | CATCGGGGTGCACATAGAGG | 0.506942 AAGGCAGTAGAAGCAGGTGC | AGATGAGAGGTCGAGGGAGG | 0.509486 TGGCCCATGATGAAACCTGG | AGATGAGAGGTCGAGGGAGG | 0.568309 ATGAAACCTGGGGTTGAGGC | ATGTCGATCTGCTCATCGGG | | | | | |
| NCU00028T0 | 5 | 5 | NCU00028T0\_input.txt | NCU00028T0\_output.txt | 0.646981 CTACCCATCACCTCCAACGC | AGATGAGAGGTCGAGGGAGG | 0.777388 CATATCCCTCGACAGCCTCG | AGACAGTCTTGCGTTTGGGG | 0.859988 CTACCCATCACCTCCAACGC | TGAGAGCTTGGAGGTGTTGG | 0.913359 CGAGGGGCATGATTTTGTCG | AGACAGTCTTGCGTTTGGGG | 0.940619 GGCCCATCTGTACTCTCAGG | AGATGAGAGGTCGAGGGAGG | | | | | |
| NCU00029T0 | 3 | 5 | NCU00029T0\_input.txt | NCU00029T0\_output.txt | 0.207606 GGAAAGCCACTAGAGCCAGG | CGTCGACCCCTACTTAGTGC | 0.566538 GGAAAGCCACTAGAGCCAGG | GTCGACCCCTACTTAGTGCC | 0.641411 GGAAAGCCACTAGAGCCAGG | ATTACCAAGACGTCGACCCC | 1.065586 TGTTGAAAAAGCGGATGGGG | CGTCGACCCCTACTTAGTGC | 1.100318 CTTGCAAGGTCAAGGAAAGCC | CGTCGACCCCTACTTAGTGC | | | | | |
| NCU00030T0 | 1 | 5 | NCU00030T0\_input.txt | NCU00030T0\_output.txt | 0.420940 TGTACTTGCATACAGGCGGG | TCGACTTCGGACAGTGTTCC | 0.483014 CTCGATTTCTTGCCGTGACG | TGTTCCCTTTCCAATGGGGG | 0.500799 AGGAGGAAGAGGTTGTGTGC | CTTGGGTGATACCGTCCTCG | 0.679914 CTCGATTTCTTGCCGTGACG | TCGACTTCGGACAGTGTTCC | 0.762183 CTCGATTTCTTGCCGTGACG | TCGGACAGTGTTCCCTTTCC | | | | | |
| NCU00031T0 | 3 | 5 | NCU00031T0\_input.txt | NCU00031T0\_output.txt | 0.072614 TTGGGGACTTGCTCGTTACC | ACTAAGGTTGGCTGTGGAGC | 0.294514 TTGGGGACTTGCTCGTTACC | ATCACCATGGTTGCTACCCC | 0.340973 CGTAGTAGTCGAAGCCTCCG | ACTAAGGTTGGCTGTGGAGC | 0.491879 AAGCCGTAGTAGTCGAAGCC | ACTAAGGTTGGCTGTGGAGC | 0.503768 TTGGGGACTTGCTCGTTACC | ACTTTGAGTTGCCTGGTGGG | | | | | |
| NCU00032T0 | 1 | 5 | NCU00032T0\_input.txt | NCU00032T0\_output.txt | 0.414802 TGGTCAGAAGTCGTTCGACC | TCGCTGTCCTTACATACCGC | 0.419192 TGGTCAGAAGTCGTTCGACC | CTGTCCTTACATACCGCCCC | 0.419937 TGGTCAGAAGTCGTTCGACC | CTCGATTAGTTCCCCTGCCC | 0.424309 TGGTCAGAAGTCGTTCGACC | CAAACGAAGACTTGCGACGG | 0.712576 ATGTAAGGACAGCGATGGGC | GCCCCCGGTAGGAAATAACG | | | | | |
| NCU00033T0 | 2 | 5 | NCU00033T0\_input.txt | NCU00033T0\_output.txt | 0.284521 TCAGACGACATCACACCACG | TCCAGAGAAGAGTGAAGCGC | 0.353608 CCCTCAGACGACATCACACC | TCCAGAGAAGAGTGAAGCGC | 0.369123 TCAGACGACATCACACCACG | CAGTGGAGGGAAGGAAAGGG | 0.369123 TCAGACGACATCACACCACG | CCAGTGGAGGGAAGGAAAGG | 0.424131 GCCCAGGCTACATTTTTCGC | TCCAGAGAAGAGTGAAGCGC | | | | | |
| NCU00034T1 | 2 | 5 | NCU00034T1\_input.txt | NCU00034T1\_output.txt | 0.149829 CGGCTCGACGAATACCTACC | GAGGTTGAGGTCGGAGTTCG | 0.366841 CGGCTCGACGAATACCTACC | TAGGGACTTGGGCTGGTAGG | 0.423528 AATACGAAGGAGATCGCGGG | GAGGTTGAGGTCGGAGTTCG | 0.435556 AACATCGTCCAATTTGCGCC | TAGGGACTTGGGCTGGTAGG | 0.707180 CGGCTCGACGAATACCTACC | GTTGACGAGGTTGAGGTCGG | | | | | |
| NCU00034T0 | 1 | 5 | NCU00034T0\_input.txt | NCU00034T0\_output.txt | 0.275629 CTTCTTTTGGTGGGCGTTGG | CAAACGGAATCTTGGTGCCC | 0.279941 AGGCACCCGAGTTACTTTGG | CAAACGGAATCTTGGTGCCC | 0.348675 GGATCTACGGCAGGTTCAGG | CAAACGGAATCTTGGTGCCC | 0.645475 ACCGAGCTTCAACCACTTGG | CAAAGCTAACGACGTTGCCC | 0.689361 CTTCTTTTGGTGGGCGTTGG | CTGTCGAAAAAGCACCACGC | | | | | |
| NCU00035T1 | 5 | 5 | NCU00035T1\_input.txt | NCU00035T1\_output.txt | 0.289798 AGCCGATGCATATCAGGACG | TTAGCCATGGTGTTCGAGGG | 0.836413 CTCCTCGGTTGGGCTATTGG | CTGTCGAAAAAGCACCACGC | 1.017693 AGCCGATGCATATCAGGACG | CACGTAAAGCGACTACCACG | 1.133034 TTTCCGAAAGTCATGGCAGC | TTAGCCATGGTGTTCGAGGG | 1.202294 AGCCGATGCATATCAGGACG | TGCTTTAGCCATGGTGTTCG | | | | | |
| NCU00035T0 | 4 | 5 | NCU00035T0\_input.txt | NCU00035T0\_output.txt | 0.289798 AGCCGATGCATATCAGGACG | TTAGCCATGGTGTTCGAGGG | 0.836413 CTCCTCGGTTGGGCTATTGG | CTGTCGAAAAAGCACCACGC | 1.017693 AGCCGATGCATATCAGGACG | CACGTAAAGCGACTACCACG | 1.133034 TTTCCGAAAGTCATGGCAGC | TTAGCCATGGTGTTCGAGGG | 1.202294 AGCCGATGCATATCAGGACG | TGCTTTAGCCATGGTGTTCG | | | | | |
| NCU00037T0 | 4 | 5 | NCU00037T0\_input.txt | NCU00037T0\_output.txt | 0.144519 CACCTGTTTTTCACTCGCCG | GATGGTACGGAGTCTGCTGG | 0.288772 CACCTGTTTTTCACTCGCCG | TCTGCTGGATTGGTGACTCG | 0.350709 GTGAAGTTGAAAGGCCCAGC | AACAGCCTTCGTCCTTCTGG | 0.350709 GCTGGTGAAGTTGAAAGGCC | AACAGCCTTCGTCCTTCTGG | 0.421062 GCACCTGTTTTTCACTCGCC | GATGGTACGGAGTCTGCTGG | | | | | |
| NCU00037T1 | 4 | 5 | NCU00037T1\_input.txt | NCU00037T1\_output.txt | 0.144519 CACCTGTTTTTCACTCGCCG | GATGGTACGGAGTCTGCTGG | 0.288772 CACCTGTTTTTCACTCGCCG | TCTGCTGGATTGGTGACTCG | 0.350709 GTGAAGTTGAAAGGCCCAGC | AACAGCCTTCGTCCTTCTGG | 0.350709 GCTGGTGAAGTTGAAAGGCC | AACAGCCTTCGTCCTTCTGG | 0.421062 GCACCTGTTTTTCACTCGCC | GATGGTACGGAGTCTGCTGG | | | | | |
| NCU00038T0 | 2 | 5 | NCU00038T0\_input.txt | NCU00038T0\_output.txt | 0.645505 TGCTGGACCCAATCAGTAGC | TACCATTTCCGAGTGCTGCC | 0.714025 TTGGACTTGAGCATGGTCGG | TACCATTTCCGAGTGCTGCC | 0.784575 TGCTGGACCCAATCAGTAGC | CGAGGAACAGGCAGCTATGG | 0.853095 TTGGACTTGAGCATGGTCGG | CGAGGAACAGGCAGCTATGG | 0.927334 GTGAGAGTTCCTCCTCCTGC | TACCATTTCCGAGTGCTGCC | | | | | |
| NCU00039T0 | 2 | 5 | NCU00039T0\_input.txt | NCU00039T0\_output.txt | 0.350761 CTGAGGAAACAGGTCCGAGG | GCGAATTGGGGGAAGAATGC | 0.564508 CTGAGGAAACAGGTCCGAGG | ATGATTCGACCTCGTACGCG | 0.568946 CTGAGGAAACAGGTCCGAGG | TGTGGATGTGTACGCCAAGG | 0.629537 CTGAGGAAACAGGTCCGAGG | CGAATTGGGGGAAGAATGCG | 0.646811 CTGAGGAAACAGGTCCGAGG | AGTGTAAGGTTTGGGGGACG | | | | | |
| NCU00040T0 | 2 | 5 | NCU00040T0\_input.txt | NCU00040T0\_output.txt | 0.072391 AACCGTGATCTGCTGTGAGG | TTGACCAAAGATCCGAGGGC | 0.279075 GGTTGGATTGTCGCAAAGGG | TTGACCAAAGATCCGAGGGC | 0.287033 AACCGTGATCTGCTGTGAGG | GATCAAGATGAGGACGGGGC | 0.423959 GTTGGATTGTCGCAAAGGGC | TTGACCAAAGATCCGAGGGC | 0.487935 AACCGTGATCTGCTGTGAGG | GAGTTACTCTCCGTCCACCG | | | | | |
| NCU00041T0 | 3 | 5 | NCU00041T0\_input.txt | NCU00041T0\_output.txt | 0.202311 GAGGATTCCGGTTAGCCAGG | GAAACGCTGAACTGATCGCC | 0.282884 GAGGATTCCGGTTAGCCAGG | GTGACTCGAATGTCCTCCCC | 0.346551 CCGACTACGTGAGGACTTGG | GTGACTCGAATGTCCTCCCC | 0.353806 GAGGATTCCGGTTAGCCAGG | CGAAACGCTGAACTGATCGC | 0.429440 GAGGATTCCGGTTAGCCAGG | ATGTCCTCCCCTTTTGTCGC | | | | | |
| NCU00042T0 | 2 | 5 | NCU00042T0\_input.txt | NCU00042T0\_output.txt | 0.133138 TTCCTCGTGTTACCATCCGC | GTCGACGATAAAAGTGCCGC | 0.282227 TTCCTCGTGTTACCATCCGC | GAGTGGTGGTAGAAGCTCGG | 0.282227 TTCCTCGTGTTACCATCCGC | GGAGTGGTGGTAGAAGCTCG | 0.283137 TTCCTCGTGTTACCATCCGC | GCCGAGAGAAGTGGAGAAGG | 0.424274 CAAGAGGCCTCCATCTACGC | GCCGAGAGAAGTGGAGAAGG | | | | | |
| NCU00043T0 | 6 | 5 | NCU00043T0\_input.txt | NCU00043T0\_output.txt | 0.423978 TCTGTTTCTTCTCTGCGGGC | GGCATACGGTGACTGAGAGG | 0.568506 TCTGTTTCTTCTCTGCGGGC | TCGAGAAAGAGGAGGCAAGC | 0.637460 TCTGTTTCTTCTCTGCGGGC | GTGAAAACGTGAACTGGCGG | 0.849454 TCTGTTTCTTCTCTGCGGGC | GCATACGGTGACTGAGAGGC | 0.974094 TCTGTTTCTTCTCTGCGGGC | CGTGAAAACGTGAACTGGCG | | | | | |
| NCU00045T0 | 2 | 5 | NCU00045T0\_input.txt | NCU00045T0\_output.txt | 0.356764 AGAAGGCAAGAAGAAGCGGG | TGGGTTCCACTCATGTGTCG | 0.421614 CGTAGATATGCGACCGTGGG | TGGGTTCCACTCATGTGTCG | 0.491068 GTAGGCCTGAAGACGTGACG | TGGGTTCCACTCATGTGTCG | 0.581947 AGAAGGCAAGAAGAAGCGGG | AATGGATGGAGCTGGGTTCC | 0.646797 CGTAGATATGCGACCGTGGG | AATGGATGGAGCTGGGTTCC | | | | | |
| NCU00046T1 | 3 | 5 | NCU00046T1\_input.txt | NCU00046T1\_output.txt | 0.682685 GAGTTTGCGGGAGTTTGTCG | ACGCCTCGATTTGATGTTGC | 0.830437 GTTTGTCGTGGCGAAAGTCG | ACGCCTCGATTTGATGTTGC | 1.046165 GAGTTTGCGGGAGTTTGTCG | GAAGGGGAGAATTTGTGCGG | 1.079218 GGTAGAGAGGTGCGTTTCCC | TCCAACCTAGAGCACGAGGG | 1.114076 TGCGGTCTGTAGAGTTTGCG | ACGCCTCGATTTGATGTTGC | | | | | |
| NCU00046T0 | 2 | 5 | NCU00046T0\_input.txt | NCU00046T0\_output.txt | 1.054474 GGAGTGCTGGAGAAACCACC | ACGCCTCGATTTGATGTTGC | 1.215176 AAGATGGACGATGGGATGGG | ACGCCTCGATTTGATGTTGC | 1.397198 GAAACCACCAAGAACGACCC | ACGCCTCGATTTGATGTTGC | 1.417954 GGAGTGCTGGAGAAACCACC | GAAGGGGAGAATTTGTGCGG | 1.577609 GGAGTGCTGGAGAAACCACC | TCCAACCTAGAGCACGAGGG | | | | | |
| NCU00047T0 | 2 | 5 | NCU00047T0\_input.txt | NCU00047T0\_output.txt | 0.216035 CCCAGTCTCCCACAAAGACC | TTCGTGGTTTTGTGGTTGCG | 0.274578 GAGTTCACTCGATTTGGCGC | TTCGTGGTTTTGTGGTTGCG | 0.414031 GAGTTCACTCGATTTGGCGC | CCACCACCATTCAAACACGC | 0.429952 CCCAGTCTCCCACAAAGACC | TTTAGCTAGCCTCCATCCGC | 0.605776 GAGTTCACTCGATTTGGCGC | GAGTTTGTGCAGACAGAGCG | | | | | |
| NCU00048T0 | 4 | 5 | NCU00048T0\_input.txt | NCU00048T0\_output.txt | 0.066239 CAAACTGTTGCTCCCTTGGC | AGCTTCCATTGGAGAAGCCC | 0.209459 TTAGACATGCTGAGACGCCC | AGCTTCCATTGGAGAAGCCC | 0.415772 CAAACTGTTGCTCCCTTGGC | CATTGGAGAAGCCCATTCGC | 0.497734 TGTTCGGAATCGGATCCTGG | AGCTTCCATTGGAGAAGCCC | 0.497828 CAAACTGTTGCTCCCTTGGC | ACAAGGTTGCAAAAGCCAGG | | | | | |
| NCU00050T0 | 2 | 5 | NCU00050T0\_input.txt | NCU00050T0\_output.txt | 0.639477 GTGGATGCTGATGAGGGTCG | CAAGGTCTGCCACTAGAGCC | 0.707673 GCTGATGAGGGTCGATGAGC | CAAGGTCTGCCACTAGAGCC | 0.708991 TGTCAAGAAGTCTGGCTGCG | CAAGGTCTGCCACTAGAGCC | 0.709136 GTGGATGCTGATGAGGGTCG | CTGCCACTAGAGCCTTCTGG | 0.776480 AACCCAGAAAAAGCTGTCGC | CAAGGTCTGCCACTAGAGCC | | | | | |
| NCU00051T0 | 1 | 5 | NCU00051T0\_input.txt | NCU00051T0\_output.txt | 0.825868 CAAGGTCTATCTGAGGGGCG | CGCTAATATTCGCACTCGGC | 0.971014 GCCGAGTGCGAATATTAGCG | AGCAGCTTTGGACACACTCC | 1.168737 CTCAGGAGGTCGTTCTACCG | CGCTAATATTCGCACTCGGC | 1.188811 TATGACTTCTGGATGGGCGC | CAACCGCAACTCCTTTCGC | 1.188811 TATGACTTCTGGATGGGCGC | GCAACCGCAACTCCTTTCG | | | | | |
| NCU00052T0 | 2 | 5 | NCU00052T0\_input.txt | NCU00052T0\_output.txt | 1.139141 AGAATAAGGTCGGGTAGCCG | GGAGTCGTCGAAGGACATCC | 1.145641 AGAATAAGGTCGGGTAGCCG | GGTGGGGGTATTTGGAGTCG | 1.217372 AGAATAAGGTCGGGTAGCCG | GGGGGTATTTGGAGTCGTCG | 1.255066 CCAGCGGCGACAGAATAAGG | GGAGTCGTCGAAGGACATCC | 1.358330 AGAATAAGGTCGGGTAGCCG | TTCCAAAAATCCTTGGCGGC | | | | | |
| NCU00054T0 | 6 | 5 | NCU00054T0\_input.txt | NCU00054T0\_output.txt | 0.217916 ACAAGACTATCACCGACGCG | ACATTGGGAGCCTTCTGTGG | 0.350402 ACAAGACTATCACCGACGCG | GCGAAAAGGATGGTGAAGGC | 0.436685 TTGACCCACAAGCAGAGAGG | ACATTGGGAGCCTTCTGTGG | 0.569171 TTGACCCACAAGCAGAGAGG | GCGAAAAGGATGGTGAAGGC | 0.626972 ACAAGACTATCACCGACGCG | CGCGAAAAGGATGGTGAAGG | | | | | |
| NCU00055T0 | 2 | 5 | NCU00055T0\_input.txt | NCU00055T0\_output.txt | 0.917379 ACTGAGAAGAGGTGAAGGCG | AGGTGCTGTTGAAACATGCG | 0.996449 ACTGAGAAGAGGTGAAGGCG | ACTGTGGAGCGATATGAGCC | 1.043304 TAGTTGTTCTTCGGCTCGCG | AGGTGCTGTTGAAACATGCG | 1.050902 CTGAGAAGAGGTGAAGGCGC | AGGTGCTGTTGAAACATGCG | 1.053167 TAGTTGTTCTTCGGCTCGCG | CTCGGGGGAAGAAACTGTGG | | | | | |
| NCU00056T0 | 3 | 5 | NCU00056T0\_input.txt | NCU00056T0\_output.txt | 0.059673 CGACGAGTCCAGGATTACGG | TTGACTTCCTTTTGCTGCGC | 0.065909 CGACGAGTCCAGGATTACGG | GGTCAAGTTGAGAGTGGGGG | 0.067009 CGACGAGTCCAGGATTACGG | CAAAGCTGTTGGACATGGCC | 0.067871 GGGTGAAGAAGGAGTGGACG | TTGACTTCCTTTTGCTGCGC | 0.074107 GGGTGAAGAAGGAGTGGACG | GGTCAAGTTGAGAGTGGGGG | | | | | |
| NCU00057T0 | 2 | 5 | NCU00057T0\_input.txt | NCU00057T0\_output.txt | 0.135641 CGACTACACCATCACTCCCG | GAGAAGGTTGGGAGAAGGCC | 0.214067 GCCGACTACACCATCACTCC | GAGAAGGTTGGGAGAAGGCC | 0.352023 CGACTACACCATCACTCCCG | CCCAGGTTGAGAAGGTTGGG | 0.421190 CGACTACACCATCACTCCCG | GGTGGAAGAAGTGGTCGACC | 0.421452 CGACTACACCATCACTCCCG | CCTGGTGGAAGAAGTGGTCG | | | | | |
| NCU00059T0 | 2 | 5 | NCU00059T0\_input.txt | NCU00059T0\_output.txt | 0.289158 GCTTGATCGGCATTTTGGGG | CAACATGGCAGTGACGAAGC | 0.289158 GGCTTGATCGGCATTTTGGG | CAACATGGCAGTGACGAAGC | 0.289158 GGGCTTGATCGGCATTTTGG | CAACATGGCAGTGACGAAGC | 0.417391 GCTTGATCGGCATTTTGGGG | GGTTCAACATGGCAGTGACG | 0.417391 GGCTTGATCGGCATTTTGGG | GGTTCAACATGGCAGTGACG | | | | | |
| NCU00060T2 | 2 | 5 | NCU00060T2\_input.txt | NCU00060T2\_output.txt | 0.566901 TGATTCCGTCACGTCTTCCG | GCAAGTGATTAGGGCCAAGC | 0.717588 TGATTCCGTCACGTCTTCCG | TGGATGAAGTGGACAGGTCG | 0.852555 TGATTCCGTCACGTCTTCCG | GGCCAAGCAGATCACATTCC | 0.979807 CAAGTGAGAGTGCAAAGGCG | GGCCAAGCAGATCACATTCC | 1.001521 TGATTCCGTCACGTCTTCCG | TGGGTTTTTATGGAAGCCGC | | | | | |
| NCU00060T1 | 2 | 5 | NCU00060T1\_input.txt | NCU00060T1\_output.txt | 0.566901 TGATTCCGTCACGTCTTCCG | GCAAGTGATTAGGGCCAAGC | 0.717588 TGATTCCGTCACGTCTTCCG | TGGATGAAGTGGACAGGTCG | 0.852555 TGATTCCGTCACGTCTTCCG | GGCCAAGCAGATCACATTCC | 0.979807 CAAGTGAGAGTGCAAAGGCG | GGCCAAGCAGATCACATTCC | 1.001521 TGATTCCGTCACGTCTTCCG | TGGGTTTTTATGGAAGCCGC | | | | | |
| NCU00060T0 | 1 | 5 | NCU00060T0\_input.txt | NCU00060T0\_output.txt | 0.142272 AGCACTTCTTCGGTAGTGGC | CTCCACCTGATTCCGCTACC | 0.208687 AGCACTTCTTCGGTAGTGGC | CTACACTCAGACTGGTCGGC | 0.276494 GTAGCGGAATCAGGTGGAGG | CTACACTCAGACTGGTCGGC | 0.428668 GTAGCGGAATCAGGTGGAGG | TTCTTCCCGGCACAGTAAGG | 0.496833 GTAGCGGAATCAGGTGGAGG | GCCCTTCAACTCTTCCTCCG | | | | | |
| NCU00061T0 | 1 | 5 | NCU00061T0\_input.txt | NCU00061T0\_output.txt | 0.444856 AGGGAGAGGGATGAGGATGG | ATCCAAGCAACCAACCCTCC | 0.646459 AGGGAGAGGGATGAGGATGG | CCCCAGAAATCAAAGCGAGC | 1.060542 AACGCCACGGATGAAAATCC | ATCCAAGCAACCAACCCTCC | 1.198520 AGGGAGAGGGATGAGGATGG | TATCCAAGCAACCAACCCTCC | 1.248607 TAACGGGGCAATGAGGATGG | GCTTCTCTCTACCACAACCCC | | | | | |
| NCU00064T0 | 5 | 5 | NCU00064T0\_input.txt | NCU00064T0\_output.txt | 0.216481 CCTTCTATGAGACGTGGCCC | CACGTTGAACCGCTGTAACG | 0.221498 CAATCGCTTGACACCTGTGC | CACGTTGAACCGCTGTAACG | 0.287202 TGCCTTCTATGAGACGTGGC | CACGTTGAACCGCTGTAACG | 0.289245 CAATCGCTTGACACCTGTGC | GCTGGAAGCGACAAAATCCG | 0.361651 CCTCAGGGATCCATGAAGCG | CACGTTGAACCGCTGTAACG | | | | | |
| NCU00065T0 | 2 | 5 | NCU00065T0\_input.txt | NCU00065T0\_output.txt | 0.545842 GAACGAAGCTTCATGGCAGG | GAGTCACGGTATTTTGGCGC | 0.625444 CTGCGACAACAGCTTCTTCG | TATGTTGTTCATCGGCGGGG | 0.912325 GAACGAAGCTTCATGGCAGG | TATGTTGTTCATCGGCGGGG | 0.968999 CTGCGACAACAGCTTCTTCG | GGAGAAACGATCGGGTCAGC | 0.969800 GAACGAAGCTTCATGGCAGG | CACGGTATTTTGGCGCTTCG | | | | | |
| NCU00066T0 | 1 | 5 | NCU00066T0\_input.txt | NCU00066T0\_output.txt | 0.062913 TTCGCCTGGTGATCGATACG | AGTCAGTTCTCAGCACACCG | 0.357164 TGGCCACTAGAATCTGGTGC | AAGGAAGTTTTGGTTGCGGC | 0.502439 AAGAGGAGGTCACCAACTGC | AAGGAAGTTTTGGTTGCGGC | 0.556304 TGTCGTCTCAAGATCTCGCC | AAGGAAGTTTTGGTTGCGGC | 0.563878 GAAGGAGGTGAGGGTGTACG | AGTCAGTTCTCAGCACACCG | | | | | |
| NCU00067T0 | 3 | 5 | NCU00067T0\_input.txt | NCU00067T0\_output.txt | 0.145521 TTGAACACTCGGCTAACCCC | CGTTTTGCAAGGGGTGATCG | 0.219007 AGCTCGACAAACTCGTAGGC | CGTTTTGCAAGGGGTGATCG | 0.279375 TTGAACACTCGGCTAACCCC | GTTTTGCAAGGGGTGATCGG | 0.352861 AGCTCGACAAACTCGTAGGC | GTTTTGCAAGGGGTGATCGG | 0.356861 TTGAACACTCGGCTAACCCC | GATGAGGATCACGGGGATGC | | | | | |
| NCU00068T1 | 3 | 5 | NCU00068T1\_input.txt | NCU00068T1\_output.txt | 0.506187 TGTCCTTGCCATTCTTCCGG | GGGCTAGGATGGAGTTTGGG | 0.506187 TGTCCTTGCCATTCTTCCGG | GGGGCTAGGATGGAGTTTGG | 0.636649 CGAGCACAGAGGATCATCCG | GACACTGGGCAAATGTTGGG | 0.636649 CGAGCACAGAGGATCATCCG | GGACACTGGGCAAATGTTGG | 0.640576 TGTCCTTGCCATTCTTCCGG | GACACTGGGCAAATGTTGGG | | | | | |
| NCU00068T0 | 3 | 5 | NCU00068T0\_input.txt | NCU00068T0\_output.txt | 0.506187 TGTCCTTGCCATTCTTCCGG | GGGCTAGGATGGAGTTTGGG | 0.506187 TGTCCTTGCCATTCTTCCGG | GGGGCTAGGATGGAGTTTGG | 0.636649 CGAGCACAGAGGATCATCCG | GACACTGGGCAAATGTTGGG | 0.636649 CGAGCACAGAGGATCATCCG | GGACACTGGGCAAATGTTGG | 0.640576 TGTCCTTGCCATTCTTCCGG | GACACTGGGCAAATGTTGGG | | | | | |
| NCU00069T0 | 2 | 5 | NCU00069T0\_input.txt | NCU00069T0\_output.txt | 0.429378 TCGCAGGCTATGGTAAACCC | GATATGCCGGACTGTCACCC | 0.491257 TCGCAGGCTATGGTAAACCC | GCAACCCACATGATATGCCG | 0.565946 TCGCAGGCTATGGTAAACCC | CTCGCTTTTGCTTGTCTGCC | 0.643503 TCGCAGGCTATGGTAAACCC | TGATATGCCGGACTGTCACC | 0.767720 TCGCAGGCTATGGTAAACCC | TCTCGCTTTTGCTTGTCTGC | | | | | |
| NCU00070T0 | 2 | 5 | NCU00070T0\_input.txt | NCU00070T0\_output.txt | 0.274103 TGGTAGTCGTGGCGTTATCG | TCCCATAGCGGAAGTTGACG | 0.565448 TGGTAGTCGTGGCGTTATCG | ATGGCCCTTGTTATGGAGCG | 0.774049 AACACTTGGTAGTCGTGGCG | TCCCATAGCGGAAGTTGACG | 0.834272 TAGTCGTGGCGTTATCGAGG | TCCCATAGCGGAAGTTGACG | 0.888728 ATGATGTCGTCGTTTCGTGC | TCCCATAGCGGAAGTTGACG | | | | | |
| NCU00071T0 | 4 | 5 | NCU00071T0\_input.txt | NCU00071T0\_output.txt | 0.832528 TACCCGTTGATGGTGATGGC | GTATCAAACCGAACAGGGCG | 0.924236 TACCCGTTGATGGTGATGGC | CAGGGCGAGATTGTCCATGG | 0.975817 TACCCGTTGATGGTGATGGC | GCCTAACAGCATACGCATCC | 1.191781 TACCCGTTGATGGTGATGGC | CGAACAGGGCGAGATTGTCC | 1.199758 TACCCGTTGATGGTGATGGC | GGTATCAAACCGAACAGGGC | | | | | |
| NCU00072T0 | 3 | 5 | NCU00072T0\_input.txt | NCU00072T0\_output.txt | 0.839047 GGATGGTAGACAGCGGATGG | GTTCTCTTGGACGCCAATGG | 0.839047 GATGGGATGGTAGACAGCGG | GTTCTCTTGGACGCCAATGG | 0.839047 GGATGGGATGGTAGACAGCG | GTTCTCTTGGACGCCAATGG | 0.840728 TTTCCGTACCAACCCAGAGC | GTTCTCTTGGACGCCAATGG | 1.013759 TACCAACCCAGAGCAAGTCC | TTGCGGTTAGGGGATGTTGG | | | | | |
| NCU00073T0 | 3 | 5 | NCU00073T0\_input.txt | NCU00073T0\_output.txt | 1.061465 GGATTGGAAGACACCGACGG | GTGGGGAGCACTGTAGTTGG | 1.061465 GGGATTGGAAGACACCGACG | GTGGGGAGCACTGTAGTTGG | 1.600290 CGGTCGTGAGGTTTGTTGC | GTGGGGAGCACTGTAGTTGG | 1.608219 GGATTGGAAGACACCGACGG | GCAGTCAAAACTCCACGAGG | 1.608219 GGGATTGGAAGACACCGACG | GCAGTCAAAACTCCACGAGG | | | | | |
| NCU00074T0 | 2 | 5 | NCU00074T0\_input.txt | NCU00074T0\_output.txt | 0.426592 TACCTACCTACCACTCGCCC | TTGTTGGCGAGGTAGAACCG | 0.430129 TTACCAGTCGTTCTCGTCGC | TTGTTGGCGAGGTAGAACCG | 0.562090 ACCAGTCGTGCTTACGTACC | TTGTTGGCGAGGTAGAACCG | 0.700251 TTACCAGTCGTTCTCGTCGC | CTGCTGAGACTGGTTTGTGC | 0.710545 GAAGTCTTCCGCAGAGTGGG | TTGTTGGCGAGGTAGAACCG | | | | | |
| NCU00075T0 | 3 | 5 | NCU00075T0\_input.txt | NCU00075T0\_output.txt | 0.429277 TACAAGGGTGGTTTCGAGCC | GTGGTTGAGGAGCATGAGGG | 0.429277 TACAAGGGTGGTTTCGAGCC | GGTGGTTGAGGAGCATGAGG | 0.497787 TACAAGGGTGGTTTCGAGCC | GACCTTGGTGGCCAGATACG | 0.827025 GGCTTCGTTGATTCTCTCGC | ACTGCATCGAGACGGATTGG | 0.906088 ACAGATGTCATGCCTGAGCC | CTTGTCCTTCGTGATGGTGC | | | | | |
| NCU00076T1 | 7 | 5 | NCU00076T1\_input.txt | NCU00076T1\_output.txt | 0.348436 ACTCTCCCTCGATGGTCTCC | AAAGCGAGCATCAAACCAGC | 0.621546 ACTCTCCCTCGATGGTCTCC | TCTGGCAATGTTGTAACGCG | 0.622488 GGAATACAAGGACCACGGGG | TCTGGCAATGTTGTAACGCG | 0.632489 GGTCTCCTTCAGAACGCTGG | AAAGCGAGCATCAAACCAGC | 0.706675 GGTCTCCTTCAGAACGCTGG | CACAGGCAGGCAAGAAAAGG | | | | | |
| NCU00076T0 | 7 | 5 | NCU00076T0\_input.txt | NCU00076T0\_output.txt | 0.348436 ACTCTCCCTCGATGGTCTCC | AAAGCGAGCATCAAACCAGC | 0.621546 ACTCTCCCTCGATGGTCTCC | TCTGGCAATGTTGTAACGCG | 0.622488 GGAATACAAGGACCACGGGG | TCTGGCAATGTTGTAACGCG | 0.632489 GGTCTCCTTCAGAACGCTGG | AAAGCGAGCATCAAACCAGC | 0.706675 GGTCTCCTTCAGAACGCTGG | CACAGGCAGGCAAGAAAAGG | | | | | |
| NCU00077T0 | 7 | 5 | NCU00077T0\_input.txt | NCU00077T0\_output.txt | 0.279632 GGGAATCGCATTTTCCGTGG | CGAATTTGGGTCTGGCATGC | 0.283433 CCCGGATAGAGGTTTGACGG | CGAATTTGGGTCTGGCATGC | 0.346043 GGGAATCGCATTTTCCGTGG | ATGCACAGTGCCAATTCTGC | 0.349843 CCCGGATAGAGGTTTGACGG | ATGCACAGTGCCAATTCTGC | 0.492953 CCCGGATAGAGGTTTGACGG | CTTGTTCGTCACTCCCTCCG | | | | | |
| NCU00078T0 | 4 | 5 | NCU00078T0\_input.txt | NCU00078T0\_output.txt | 0.207847 ATCTTCTTTACCGGCCTGGC | AGCCATACACGACAGTAGCG | 0.216762 ATCTTCTTTACCGGCCTGGC | CGTGAAGCCAATGTGAAGCC | 0.344318 CAACCTTTCCGTGATTGGGC | AGCCATACACGACAGTAGCG | 0.344318 GCCAACCTTTCCGTGATTGG | AGCCATACACGACAGTAGCG | 0.353233 CAACCTTTCCGTGATTGGGC | CGTGAAGCCAATGTGAAGCC | | | | | |
| NCU00079T0 | 1 | 5 | NCU00079T0\_input.txt | NCU00079T0\_output.txt | 0.773228 TTCGGTGTTTGATCATGCGC | TCCGTTGTTCCATCACTGCC | 0.834485 TTCGGTGTTTGATCATGCGC | ACTGCCGTTGTACTTCGAGC | 0.851954 AGATCGGGTGGAGTTGTTCG | TCCGTTGTTCCATCACTGCC | 0.913212 AGATCGGGTGGAGTTGTTCG | ACTGCCGTTGTACTTCGAGC | 0.966684 TTCGGTGTTTGATCATGCGC | CTCCGTTGTTCCATCACTGC | | | | | |
| NCU00080T0 | 3 | 5 | NCU00080T0\_input.txt | NCU00080T0\_output.txt | 0.135889 AGCACGAACGATATCCCACC | TCTTCGAACACAGTGGGACG | 0.138362 AGCACGAACGATATCCCACC | AGCTCTTCGAACACAGTGGG | 0.283250 CAGCTGTAGAGAGCTCAGGC | ATAATGTGTCAGGGGTCCGC | 0.284710 AGCACGAACGATATCCCACC | ATAATGTGTCAGGGGTCCGC | 0.652814 AGCACGAACGATATCCCACC | AGGTTTCTGGGGCATCTAGC | | | | | |
| NCU00081T0 | 6 | 5 | NCU00081T0\_input.txt | NCU00081T0\_output.txt | 0.215133 TCTTGCGGGAGAGTATTGCG | AAGCCTGAGTTCTGTGAGGC | 0.276968 AAGGCAATTTGCGAAGGTCG | AAGCCTGAGTTCTGTGAGGC | 0.359517 TCTTGCGGGAGAGTATTGCG | TCTAGAATTCAAGCCCCGCC | 0.421352 AAGGCAATTTGCGAAGGTCG | TCTAGAATTCAAGCCCCGCC | 0.720501 TGATCAGTCACGGGAGGAGG | AAGCCTGAGTTCTGTGAGGC | | | | | |
| NCU00082T0 | 3 | 5 | NCU00082T0\_input.txt | NCU00082T0\_output.txt | 0.148481 TTGCTTTCGCCTCATGTTGC | GTCGTCCACTAGTCACCAGC | 0.484331 CGGAATCTCCTTGCTTTCGC | GTCGTCCACTAGTCACCAGC | 0.636791 ATCATTTCGTTGCCCTGTGC | GTCGTCCACTAGTCACCAGC | 0.699958 GTCCGCGACATACTACACGG | GTCGTCCACTAGTCACCAGC | 0.735179 TTGCTTTCGCCTCATGTTGC | TACCTCATTGTGTGTGGGGG | | | | | |
| NCU00083T0 | 2 | 5 | NCU00083T0\_input.txt | NCU00083T0\_output.txt | 0.198944 GAGTTACTTTGCCAGCTGCG | CAGCAGATGCCATGATTGCC | 0.209546 GAGTTACTTTGCCAGCTGCG | TGGAGTTGGGAGGAGTAGGG | 0.412354 GAGTTACTTTGCCAGCTGCG | TTAAGTTGTCTCGGCCGAGG | 0.475999 ATCATCGCGACAACAACAGC | CAGCAGATGCCATGATTGCC | 0.507022 GAGTTACTTTGCCAGCTGCG | AGTTGGGAGGAGTAGGGAGG | | | | | |
| NCU00084T0 | 4 | 5 | NCU00084T0\_input.txt | NCU00084T0\_output.txt | 0.559008 TGAGTTTTGCCGTTTGTGCG | GAGGAGATCATGGGGAAGCG | 0.717461 TGAGTTTTGCCGTTTGTGCG | AGGGAGAGGAGATCATGGGG | 0.857972 TTCCAGTCACTGCTTTGGGC | GAGGAGATCATGGGGAAGCG | 0.930815 TTCCAGTCACTGCTTTGGGC | GGAGGAGATTGAGTGGGTGC | 0.989068 GTTCCAGTCACTGCTTTGGG | GAGGAGATCATGGGGAAGCG | | | | | |
| NCU00085T0 | 1 | 5 | NCU00085T0\_input.txt | NCU00085T0\_output.txt | 0.144545 CAAGCAGGATGGTCTGAGGG | AAGTAAGGTCTCACTGCCGC | 0.210667 ATTGTCCGACTCGATGCTCC | GCCATTCTGTCTCCCAGTCC | 0.272216 ATTGTCCGACTCGATGCTCC | GAGTTGGACCAAATCGCTGC | 0.281139 AGTCCGGTTGAGTGTGATCG | AAGTAAGGTCTCACTGCCGC | 0.282342 ATTGTCCGACTCGATGCTCC | CTTGTCCTAGAGAACCGCCG | | | | | |
| NCU00086T0 | 2 | 5 | NCU00086T0\_input.txt | NCU00086T0\_output.txt | 0.486905 GTGTGCTTCAACAACGAGGG | CTGGTCGTAGTTACAGCCCG | 0.553300 GTGTGCTTCAACAACGAGGG | TGCTGGTCGTAGTTACAGCC | 0.572222 CAGGGAGTGGTAATAGGCGG | CTGGTCGTAGTTACAGCCCG | 0.572222 CCAGGGAGTGGTAATAGGCG | CTGGTCGTAGTTACAGCCCG | 0.630576 GTGTGCTTCAACAACGAGGG | TGCCCTTAAAGCTCTGCTGG | | | | | |
| NCU00087T0 | 1 | 5 | NCU00087T0\_input.txt | NCU00087T0\_output.txt | 0.071515 TTGACTTGCTTGCCCATTGC | TTTCTCTTTCCCTTCCGCCC | 0.137169 GTTGCTTGGCTTATCGCAGG | TTTCTCTTTCCCTTCCGCCC | 0.207140 CTCCCACTTAACCTCGTCCG | AAGCCAAGCAACAGCAATGG | 0.276067 TTGACTTGCTTGCCCATTGC | CCTCCTATCGCTGGTCATCG | 0.280020 CGATGACCAGCGATAGGAGG | TTTCTCTTTCCCTTCCGCCC | | | | | |
| NCU00088T0 | 3 | 5 | NCU00088T0\_input.txt | NCU00088T0\_output.txt | 1.829432 TGTTTTCGAGGGTGTTGTCG | AAGACCTTGTTCTTGGGCGG | 1.903415 AGGTGTTTTTGTTCGACTGCG | TCTGCTGGGATTCCTAAGCC | 2.132008 TGTTTTCGAGGGTGTTGTCG | TCTGCTGGGATTCCTAAGCC | 2.293439 TGTTTTCGAGGGTGTTGTCG | CAAAGACCTTGTTCTTGGGCG | 2.302737 GGTGTTTTTGTTCGACTGCG | TCTGCTGGGATTCCTAAGCC | | | | | |
| NCU00089T0 | 2 | 5 | NCU00089T0\_input.txt | NCU00089T0\_output.txt | 0.073410 TCCCAAGGTACCGTCATTGC | TTCCATGGTGGGAAACGAGG | 0.286591 TCCCAAGGTACCGTCATTGC | AGTTGGTCGAAAAGAGGCCC | 0.287247 ATCTTTGATCTCTCCCGCGC | TTCCATGGTGGGAAACGAGG | 0.295467 TCCCAAGGTACCGTCATTGC | TGGGGATGAGCTTTCCATGG | 0.500428 ATCTTTGATCTCTCCCGCGC | AGTTGGTCGAAAAGAGGCCC | | | | | |
| NCU00089T1 | 2 | 5 | NCU00089T1\_input.txt | NCU00089T1\_output.txt | 0.353794 TCTTTCTTGTCGACGGTCCC | TTCCATGGTGGGAAACGAGG | 0.429799 ACCCGGAAAGATATTGGGCG | AGTTGGTCGAAAAGAGGCCC | 0.508387 TTGGGCGGAATAAGGAAGGG | AGTTGGTCGAAAAGAGGCCC | 0.566974 TCTTTCTTGTCGACGGTCCC | AGTTGGTCGAAAAGAGGCCC | 0.575851 TCTTTCTTGTCGACGGTCCC | TGGGGATGAGCTTTCCATGG | | | | | |
| NCU00089T2 | 2 | 5 | NCU00089T2\_input.txt | NCU00089T2\_output.txt | 0.295206 TTGGGCGGAATAAGGAAGGG | TTCCATGGTGGGAAACGAGG | 0.353794 TCTTTCTTGTCGACGGTCCC | TTCCATGGTGGGAAACGAGG | 0.429799 ACCCGGAAAGATATTGGGCG | AGTTGGTCGAAAAGAGGCCC | 0.508387 TTGGGCGGAATAAGGAAGGG | AGTTGGTCGAAAAGAGGCCC | 0.566974 TCTTTCTTGTCGACGGTCCC | AGTTGGTCGAAAAGAGGCCC | | | | | |
| NCU00089T3 | 2 | 5 | NCU00089T3\_input.txt | NCU00089T3\_output.txt | 0.216619 ACCCGGAAAGATATTGGGCG | TTCCATGGTGGGAAACGAGG | 0.288103 GTTCAGGGTGTAGAGCTGGG | TTCCATGGTGGGAAACGAGG | 0.295206 TTGGGCGGAATAAGGAAGGG | TTCCATGGTGGGAAACGAGG | 0.355528 GTAGTATCCGGACTGCAGCG | TTCCATGGTGGGAAACGAGG | 0.429799 ACCCGGAAAGATATTGGGCG | AGTTGGTCGAAAAGAGGCCC | | | | | |
| NCU00090T0 | 3 | 5 | NCU00090T0\_input.txt | NCU00090T0\_output.txt | 0.549821 AGTTCCACTGGCATGTGAGG | GTGAAAAATGGCGGCAAAGC | 0.569418 AGTTCCACTGGCATGTGAGG | AGATGGTGAAAAATGGCGGC | 0.576354 AGTTCCACTGGCATGTGAGG | GGGAGTGTCTGACCTCATGG | 0.980663 AGTTCCACTGGCATGTGAGG | TGAAAAATGGCGGCAAAGCG | 0.987604 AAGGTTGTTGGTGCTCTTGC | GTTTGTGTGGAATCTGGCCG | | | | | |
| NCU00091T1 | 5 | 5 | NCU00091T1\_input.txt | NCU00091T1\_output.txt | 0.359225 ATCATCCCGCCATCTGAAGC | AGCACTTACCATATGGGCCG | 0.567822 ATCTGAAGCTGCTTCGTCGG | AGCACTTACCATATGGGCCG | 0.752916 CATCTGAAGCTGCTTCGTCG | AGCACTTACCATATGGGCCG | 1.189086 TCTGAAGCTGCTTCGTCGG | AGCACTTACCATATGGGCCG | 1.255515 TCATCCCGCCATCTGAAGC | AGCACTTACCATATGGGCCG | | | | | |
| NCU00091T0 | 4 | 5 | NCU00091T0\_input.txt | NCU00091T0\_output.txt | 0.290845 ATCATCCCGCCATCTGAAGC | AAAGGGGATTCCACTGCTGG | 0.356807 ATCATCCCGCCATCTGAAGC | TCGGTAGATGAGTGGGGAGG | 0.499441 ATCTGAAGCTGCTTCGTCGG | AAAGGGGATTCCACTGCTGG | 0.500778 GGTCAGGAGCATGTGAGTGG | AGCACTTACCATATGGGCCG | 0.565404 ATCTGAAGCTGCTTCGTCGG | TCGGTAGATGAGTGGGGAGG | | | | | |
| NCU00092T0 | 2 | 5 | NCU00092T0\_input.txt | NCU00092T0\_output.txt | 0.205039 CGTGTCAGAGCAATTGAGCG | CGGCTTCGTCTTTGGTTTCG | 0.290027 GTGTCAGAGCAATTGAGCGC | CGGCTTCGTCTTTGGTTTCG | 0.290027 GCGTGTCAGAGCAATTGAGC | CGGCTTCGTCTTTGGTTTCG | 0.422925 AAGAACGAAAAGCTGCCTGC | CGGCTTCGTCTTTGGTTTCG | 0.491186 AAGAACGAAAAGCTGCCTGC | TTTTAGCCTGGCGATCTCGG | | | | | |
| NCU00093T0 | 1 | 5 | NCU00093T0\_input.txt | NCU00093T0\_output.txt | 0.210258 CCCATAGAAAGCGTGGGAGG | AATGGCTATGGGGGTTTGGG | 0.212548 CCCATAGAAAGCGTGGGAGG | CTATGGGGGTTTGGGTCACC | 0.282071 TGAATGAGGTCATCGAGGCG | CCTCCCACGCTTTCTATGGG | 0.357286 AGATGACTGGTGCCAGAACG | CATTCAAACAAACCGCCCCC | 0.360396 CCCATAGAAAGCGTGGGAGG | CACCCCCTCTATCACTTGGC | | | | | |
| NCU00095T0 | 2 | 5 | NCU00095T0\_input.txt | NCU00095T0\_output.txt | 0.206636 TCTCATGCCTTGGTCGTACG | ACTTGAAGTGGACCTGCTCG | 0.344913 CGTCTTTGTCAACTTGGGCC | ACTTGAAGTGGACCTGCTCG | 0.346285 TCTCATGCCTTGGTCGTACG | CTCGAGTTGGGTAGAGGTGC | 0.350009 TCGAAAGCTACGAGATCGCG | ACTTGAAGTGGACCTGCTCG | 0.353270 TCGTACGTGGCTGAACAAGG | ACTTGAAGTGGACCTGCTCG | | | | | |
| NCU00096T0 | 3 | 5 | NCU00096T0\_input.txt | NCU00096T0\_output.txt | 0.271557 CCGCTTTGTCGTTTCCTTCC | ACAGACCGCTTGTTCTGAGG | 0.272265 CCGCTTTGTCGTTTCCTTCC | AGGTGTCATCATGCTGGTGG | 0.281519 GACATGGAAAAAGGCCAGCC | ACAGACCGCTTGTTCTGAGG | 0.282227 GACATGGAAAAAGGCCAGCC | AGGTGTCATCATGCTGGTGG | 0.487836 CCGCTTTGTCGTTTCCTTCC | ACGAAAGCTGTCCTTGTCCC | | | | | |
| NCU00097T0 | 3 | 5 | NCU00097T0\_input.txt | NCU00097T0\_output.txt | 0.141978 GAGATTCTGCTGGGATCCGG | ATTTAAACCTCTCGGGGCCG | 0.142319 GAGATTCTGCTGGGATCCGG | TCCTTGATTACAGCAGCCGG | 0.146448 GAGATTCTGCTGGGATCCGG | AGCTATGGGCATGGACATGG | 0.213569 GAGATTCTGCTGGGATCCGG | AATATGCCACCCCCATGAGC | 0.275544 GCCACGTCTCTTGTTCTTGC | TCACAGCCATTTTCCTCCCC | | | | | |
| NCU00099T0 | 2 | 5 | NCU00099T0\_input.txt | NCU00099T0\_output.txt | 0.206144 CAGCCTTTACGCAGATTGCC | TCTGAGTGTCCAACAGCACC | 0.285802 TTAACATACCTGCGGCGAGG | TCTGAGTGTCCAACAGCACC | 0.407196 CAGCCTTTACGCAGATTGCC | GTGTCCAACAGCACCTTTCG | 0.425455 TTAACATACCTGCGGCGAGG | CAAGATTTCCAAACGGGGGC | 0.486855 TTAACATACCTGCGGCGAGG | GTGTCCAACAGCACCTTTCG | | | | | |
| NCU00100T0 | 6 | 5 | NCU00100T0\_input.txt | NCU00100T0\_output.txt | 0.714019 CATCGGAGGGGGATCTTTCG | CTGGGAAAGGTTAAGGGGCG | 0.736375 CATCGGAGGGGGATCTTTCG | TCTGGGAAAGGTTAAGGGGC | 0.832834 CATCGGAGGGGGATCTTTCG | GGTTCAGTTCATGTCAGCCG | 0.854132 CATCGGAGGGGGATCTTTCG | TGTTGGGTGCCGTTTGTAGG | 0.977765 CCTGGGTTAGGGGCAATAGC | GGTTCAGTTCATGTCAGCCG | | | | | |
| NCU00101T0 | 2 | 5 | NCU00101T0\_input.txt | NCU00101T0\_output.txt | 0.646716 CCATCTGTTCTACACCCCGG | GGGGAAAGGTCTACCGATGG | 0.718943 TCAGTGCGAAAACCCAGAGG | GGGGAAAGGTCTACCGATGG | 0.852025 CCATCTGTTCTACACCCCGG | GAGAGAAACGGTCCCTTGGC | 0.863165 CCATCTGTTCTACACCCCGG | TGAGAGAAACGGTCCCTTGG | 0.926091 GCAATCACCCCTTTGCTAGC | GGGGAAAGGTCTACCGATGG | | | | | |
| NCU00102T0 | 4 | 5 | NCU00102T0\_input.txt | NCU00102T0\_output.txt | 0.566658 AGGAGGCGTTTGAGAAGTGG | ATTCTTGTCGACCGTCTCCC | 0.569945 ACCCCAAGATGCTCAAGTGG | ATTCTTGTCGACCGTCTCCC | 0.635321 TGATGCGATTGGGAGTACCG | ATTCTTGTCGACCGTCTCCC | 0.635871 GATGAGAGCCAGAGGACAGC | ATTCTTGTCGACCGTCTCCC | 0.706004 AGGAGGCGTTTGAGAAGTGG | AGTCGGCCACATTCTTGTCG | | | | | |
| NCU00103T0 | 2 | 5 | NCU00103T0\_input.txt | NCU00103T0\_output.txt | 0.360511 AAGCAAGTCGACTCGGATCG | GTCCATGCCACCCTTCTAGG | 0.797618 AAGCAAGTCGACTCGGATCG | TAAGACAGTCCATGCCACCC | 0.981768 AAGCAAGTCGACTCGGATCG | GTCGATTGACCTCGCTGACC | 1.120087 GTATACAGGTAGGCGGCAGG | GTCGATTGACCTCGCTGACC | 1.183438 AAGCAAGTCGACTCGGATCG | TTAAGACAGTCCATGCCACCC | | | | | |
| NCU00104T0 | 1 | 5 | NCU00104T0\_input.txt | NCU00104T0\_output.txt | 0.070837 AGTGTGCGAGACTCTGTTGG | AAGCCTCACGTCACACTAGC | 0.080141 AAGCTAGTGTGACGTGAGGC | ACTCCTTCTGACCTCTCCCC | 0.277971 CTTGGTGGGGAGAGAAGTCG | TATGATGTCGTCGCTTCCCG | 0.282151 TATTCGTGAGCCTTGGTGGG | TATGATGTCGTCGCTTCCCG | 0.495154 AAGCTAGTGTGACGTGAGGC | CAACTCGCGACTTCTCTCCC | | | | | |
| NCU00105T0 | 5 | 5 | NCU00105T0\_input.txt | NCU00105T0\_output.txt | 0.281638 CTTCCTCTACGGCTCGAACG | CGATACCCGTGGGATCAAGG | 0.353404 CGCCCGTCATAAGATCTGGG | CGATACCCGTGGGATCAAGG | 0.358447 ATCTGGGTCAAGGACAACGG | CGATACCCGTGGGATCAAGG | 0.422895 ACGCCCGTCATAAGATCTGG | CGATACCCGTGGGATCAAGG | 0.427124 CTTCCTCTACGGCTCGAACG | AACCAGCAAAGAGGGTGTCC | | | | | |
| NCU00106T0 | 6 | 5 | NCU00106T0\_input.txt | NCU00106T0\_output.txt | 0.766749 GGATCACGCCCATATCGAGC | CGTAAGTCGGGATCTGGACG | 0.912560 GGATCACGCCCATATCGAGC | TTGCGTAAGTCGGGATCTGG | 0.942756 GCGTATCGTTCTCGTTGACG | CAACGAGGTCGAGAGGATCC | 1.056926 GGATCACGCCCATATCGAGC | TGAGTGTTGCGTAAGTCGGG | 1.083313 GCGTATCGTTCTCGTTGACG | CGAACAATGGCTTCCACAGG | | | | | |
| NCU00107T0 | 2 | 5 | NCU00107T0\_input.txt | NCU00107T0\_output.txt | 0.217761 TGGAAGGTGGATTGGTTCGG | TGCCATAACCGACTGGATGG | 0.354693 TGGAAGGTGGATTGGTTCGG | CCTCGGACAATAGACTCGCG | 0.354837 CAGGGTGGTGGGTTTAGTCC | CCTCGGACAATAGACTCGCG | 0.411306 TGGAAGGTGGATTGGTTCGG | GTAAGCGTCCAATTGCCTCG | 0.411450 CAGGGTGGTGGGTTTAGTCC | GTAAGCGTCCAATTGCCTCG | | | | | |
| NCU00108T0 | 4 | 5 | NCU00108T0\_input.txt | NCU00108T0\_output.txt | 1.187069 GGCGGATGAAACAATGGTCG | CGCCACAAGATCAGAGCTGG | 1.232796 GGCGGATGAAACAATGGTCG | GTATTGTCAAGGGGAGGAGGC | 1.237786 GGCGGATGAAACAATGGTCG | CGTATTGTCAAGGGGAGGAGG | 1.312024 GGCGGATGAAACAATGGTCG | TATTGTCAAGGGGAGGAGGC | 1.464492 TCGTCGACTCTACGAATGCC | CGCCACAAGATCAGAGCTGG | | | | | |
| NCU00109T0 | 2 | 5 | NCU00109T0\_input.txt | NCU00109T0\_output.txt | 0.274614 CTGTGCCGGATGTTTATGCG | TAACGCTCTCAACTCCCTGC | 0.357575 CTGTGCCGGATGTTTATGCG | ACCTGATCCAGTTTGAGGCC | 0.414763 GTTGGATAACGTGCTGGTGC | TAACGCTCTCAACTCCCTGC | 0.430995 AGCGAGCTAGATGACAACGG | ACCTGATCCAGTTTGAGGCC | 0.437733 AGCTAGATGACAACGGGTGC | ACCTGATCCAGTTTGAGGCC | | | | | |
| NCU00109T1 | 3 | 5 | NCU00109T1\_input.txt | NCU00109T1\_output.txt | 0.274614 CTGTGCCGGATGTTTATGCG | TAACGCTCTCAACTCCCTGC | 0.357575 CTGTGCCGGATGTTTATGCG | ACCTGATCCAGTTTGAGGCC | 0.414763 GTTGGATAACGTGCTGGTGC | TAACGCTCTCAACTCCCTGC | 0.430995 AGCGAGCTAGATGACAACGG | ACCTGATCCAGTTTGAGGCC | 0.437733 AGCTAGATGACAACGGGTGC | ACCTGATCCAGTTTGAGGCC | | | | | |
| NCU00110T0 | 1 | 5 | NCU00110T0\_input.txt | NCU00110T0\_output.txt | 0.282577 CTGGGGTTGTCGATATCGGG | CTCCGATCTACCTGCAACGG | 0.282577 CTGGGGTTGTCGATATCGGG | CCTCCGATCTACCTGCAACG | 0.282577 CCTGGGGTTGTCGATATCGG | CTCCGATCTACCTGCAACGG | 0.282577 CCTGGGGTTGTCGATATCGG | CCTCCGATCTACCTGCAACG | 0.284713 CTGGGGTTGTCGATATCGGG | GGAAACTGAACAGGACCCCC | | | | | |
| NCU00111T0 | 3 | 5 | NCU00111T0\_input.txt | NCU00111T0\_output.txt | 0.152918 CTTTTCCAAGACGCTCGTGC | GCCATTTTGTGTTGCAACGC | 0.283349 CGGTATGACTACCACCCAGC | ATGCCTACCAAGTCGATGCC | 0.412547 CTAATGTCGACTTGTGCCGC | GCCATTTTGTGTTGCAACGC | 0.412547 GCTAATGTCGACTTGTGCCG | GCCATTTTGTGTTGCAACGC | 0.426999 CAAGACGCTCGTGCTTTTGG | GCCATTTTGTGTTGCAACGC | | | | | |
| NCU00112T0 | 4 | 5 | NCU00112T0\_input.txt | NCU00112T0\_output.txt | 0.498478 AATACCGCAGTGTCCAGAGC | TGTCTTGATGCCGACTCTGC | 0.628629 AAAATTGTGCGCCTGAGTCG | TGTCTTGATGCCGACTCTGC | 0.779990 CCCTTTCGGGCTTGTAGTGG | ATGTAGCGAAAGGAGCGAGG | 0.848191 AAGACTTCCTCTCGGCATCG | TGTCTTGATGCCGACTCTGC | 0.912843 TGTAGTGGATGATTGCGCCG | ATGTAGCGAAAGGAGCGAGG | | | | | |
| NCU00114T0 | 5 | 5 | NCU00114T0\_input.txt | NCU00114T0\_output.txt | 0.137165 GATCACCTCCTGCTCTTCGG | CCACTGGAGGAGATCGATGC | 0.212262 GGGACATGATCACCTCCTGC | CCACTGGAGGAGATCGATGC | 0.422467 GATCACCTCCTGCTCTTCGG | CACTGGAGGAGATCGATGCG | 0.497564 GGGACATGATCACCTCCTGC | CACTGGAGGAGATCGATGCG | 0.571321 GATCACCTCCTGCTCTTCGG | ATTGCCACTGGAGGAGATCG | | | | | |
| NCU00115T0 | 2 | 5 | NCU00115T0\_input.txt | NCU00115T0\_output.txt | 0.637707 GCAAGCAATTGGTGAGAGGG | AACAGTATCCCTTGTCCGCG | 0.640939 ACAAGCCGAGTACAGAAGCC | ATGTCACAGAACTGGTGCCG | 0.782769 ATCTCAGCATCGGACAAGCC | ATGTCACAGAACTGGTGCCG | 0.829873 ACAAGCCGAGTACAGAAGCC | CGATGTCACAGAACTGGTGC | 0.971702 ATCTCAGCATCGGACAAGCC | CGATGTCACAGAACTGGTGC | | | | | |
| NCU00116T1 | 5 | 5 | NCU00116T1\_input.txt | NCU00116T1\_output.txt | 0.424703 CTCTGGTCAGGTACAACCCG | TTCATTACTCCTGCTCCCGC | 0.501246 AACAATGCCTCCTCAGCAGG | TTCATTACTCCTGCTCCCGC | 0.561886 CTCTGGTCAGGTACAACCCG | TATCTCCTCCAGAATGCGCG | 0.697767 CTCCTGACTACACGCTAGGC | TATCTCCTCCAGAATGCGCG | 0.697767 GCTCCTGACTACACGCTAGG | TATCTCCTCCAGAATGCGCG | | | | | |
| NCU00116T2 | 5 | 5 | NCU00116T2\_input.txt | NCU00116T2\_output.txt | 0.424703 CTCTGGTCAGGTACAACCCG | TTCATTACTCCTGCTCCCGC | 0.501246 AACAATGCCTCCTCAGCAGG | TTCATTACTCCTGCTCCCGC | 0.561886 CTCTGGTCAGGTACAACCCG | TATCTCCTCCAGAATGCGCG | 0.697767 CTCCTGACTACACGCTAGGC | TATCTCCTCCAGAATGCGCG | 0.697767 GCTCCTGACTACACGCTAGG | TATCTCCTCCAGAATGCGCG | | | | | |
| NCU00116T0 | 4 | 5 | NCU00116T0\_input.txt | NCU00116T0\_output.txt | 0.424703 CTCTGGTCAGGTACAACCCG | TTCATTACTCCTGCTCCCGC | 0.501246 AACAATGCCTCCTCAGCAGG | TTCATTACTCCTGCTCCCGC | 0.561886 CTCTGGTCAGGTACAACCCG | TATCTCCTCCAGAATGCGCG | 0.697767 CTCCTGACTACACGCTAGGC | TATCTCCTCCAGAATGCGCG | 0.697767 GCTCCTGACTACACGCTAGG | TATCTCCTCCAGAATGCGCG | | | | | |
| NCU00117T0 | 8 | 5 | NCU00117T0\_input.txt | NCU00117T0\_output.txt | 0.216551 CATTTTCGAGGTTGGCGACG | TCACAATGGTCTCCTCACGC | 0.286398 CTTGACGTCTGGGGATAGCG | TCACAATGGTCTCCTCACGC | 0.354985 CATTTTCGAGGTTGGCGACG | TGGTCTCCTCACGCAATAGC | 0.358362 CATTTTCGAGGTTGGCGACG | TCTCCTCACGCAATAGCACG | 0.424833 CTTGACGTCTGGGGATAGCG | TGGTCTCCTCACGCAATAGC | | | | | |
| NCU00118T0 | 11 | 5 | NCU00118T0\_input.txt | NCU00118T0\_output.txt | 0.143954 AAGAGCCATTCCCTGAACCG | ACAGCGATCAGTCACTGAGC | 0.203183 AAGAGCCATTCCCTGAACCG | CATCGCGGAAGTTTTGACCC | 0.205861 ATTTCACCTCCAACGGACCC | CATCGCGGAAGTTTTGACCC | 0.355302 AAGAGCCATTCCCTGAACCG | CACTGAGCGGGATAGATGGC | 0.418915 AAGAGCCATTCCCTGAACCG | GAAGTTTTGACCCGCGTTCG | | | | | |
| NCU00119T0 | 1 | 5 | NCU00119T0\_input.txt | NCU00119T0\_output.txt | 0.282979 GGGAGTTGTTTTTGTGGGCG | AGCCCTTTCCTCCATCAAGC | 0.283993 TGCAGTGTCAGTGCCTTACC | AGCCCTTTCCTCCATCAAGC | 0.349043 ACCGCTTGATGGAGGAAAGG | GTTCCCTTTTGACCCAAGCG | 0.349043 ACCGCTTGATGGAGGAAAGG | CGTTCCCTTTTGACCCAAGC | 0.489252 ACCGCTTGATGGAGGAAAGG | ACACACAAAGTTTGGACGGC | | | | | |
| NCU00120T0 | 1 | 5 | NCU00120T0\_input.txt | NCU00120T0\_output.txt | 0.217222 CTGATGCCTGTCTCTGTCCC | GAGGCTCATCACGAGATCGG | 0.217222 CCTGATGCCTGTCTCTGTCC | GAGGCTCATCACGAGATCGG | 0.218474 CTGATGCCTGTCTCTGTCCC | CGATCATCGACACTCCGAGG | 0.218474 CCTGATGCCTGTCTCTGTCC | CGATCATCGACACTCCGAGG | 0.348920 CCCTCTCGCTATCACTGTCG | GAGGCTCATCACGAGATCGG | | | | | |
| NCU00121T0 | 4 | 5 | NCU00121T0\_input.txt | NCU00121T0\_output.txt | 0.356489 GCTTTTCGTCAAGTTGGGGC | GCACACACACTCTCTCCTCC | 0.356784 CGCATGGACCCAAAAACAGG | TCTTGCTCCTCCAACTCACG | 0.361419 TCGTCAAGTTGGGGCTAAGG | GCACACACACTCTCTCCTCC | 0.430028 ACAGGCACCAGTAGCTTTGG | AGATGCGTGATGTCGTCTGG | 0.497826 GCTTTTCGTCAAGTTGGGGC | AGATGCGTGATGTCGTCTGG | | | | | |
| NCU00122T0 | 2 | 5 | NCU00122T0\_input.txt | NCU00122T0\_output.txt | 0.641184 CGGGGTAGCTGTCATTCACC | GGCATACCGCAAAAAGGAGC | 2.624455 CGGGGTAGCTGTCATTCACC | GGAGCGTTGGAAAAGAGAATGG | 2.909291 CGGGGTAGCTGTCATTCACC | TTTGGCATACCGCAAAAAGG | 3.275358 GATAGGATAGGCGGGGTAGC | GGAGCGTTGGAAAAGAGAATGG | 3.285689 CGGGGTAGCTGTCATTCACC | TGGCATACCGCAAAAAGGAGC | | | | | |
| NCU00123T0 | 5 | 5 | NCU00123T0\_input.txt | NCU00123T0\_output.txt | 0.208998 GTTGACGATGGTCCTCCTGG | GACGTAGGTAGCTGGAGTGC | 0.209475 AAGCTGGATCGTTGACTCCG | GACGTAGGTAGCTGGAGTGC | 0.346400 TGGATCGTTGACTCCGAACC | GACGTAGGTAGCTGGAGTGC | 0.419901 CGTACATCCAGCAGATCCGG | GACGTAGGTAGCTGGAGTGC | 0.419901 CCGTACATCCAGCAGATCCG | GACGTAGGTAGCTGGAGTGC | | | | | |
| NCU00124T1 | 4 | 5 | NCU00124T1\_input.txt | NCU00124T1\_output.txt | 0.218016 GCTTGGTCAGAAAGCTTGCC | CGCGTTGATGAAGTTGAGGC | 0.218016 GCTTGGTCAGAAAGCTTGCC | GCGCGTTGATGAAGTTGAGG | 0.361617 GCTTGGTCAGAAAGCTTGCC | TGATGAAGTTGAGGCTGGCC | 0.637363 GCTTGGTCAGAAAGCTTGCC | ATAGCGTAGATGGACCTGCG | 0.699776 GGCAGTGCTCAAAATACGCG | TGGGCCGTAATCGTTAAGGG | | | | | |
| NCU00124T0 | 2 | 5 | NCU00124T0\_input.txt | NCU00124T0\_output.txt | 0.218016 GCTTGGTCAGAAAGCTTGCC | CGCGTTGATGAAGTTGAGGC | 0.218016 GCTTGGTCAGAAAGCTTGCC | GCGCGTTGATGAAGTTGAGG | 0.361617 GCTTGGTCAGAAAGCTTGCC | TGATGAAGTTGAGGCTGGCC | 0.637363 GCTTGGTCAGAAAGCTTGCC | ATAGCGTAGATGGACCTGCG | 0.699776 GGCAGTGCTCAAAATACGCG | TGGGCCGTAATCGTTAAGGG | | | | | |
| NCU00125T0 | 2 | 5 | NCU00125T0\_input.txt | NCU00125T0\_output.txt | 0.205157 TAACCCCCGTCTTCGTTTCG | GTGAACGCATCTCCAGTTGC | 0.430536 TAACCCCCGTCTTCGTTTCG | ATCTCCAGTTGCTCAAGCGG | 0.489326 TAACCCCCGTCTTCGTTTCG | GCATCTCCAGTTGCTCAAGC | 0.648517 TAACCCCCGTCTTCGTTTCG | TACTGCTGCTTCTGGTGAGG | 0.764029 TAACCCCCGTCTTCGTTTCG | CATCTCCAGTTGCTCAAGCG | | | | | |
| NCU00125T1 | 3 | 5 | NCU00125T1\_input.txt | NCU00125T1\_output.txt | 0.915612 TGTCCAAGCGAAACAGGAGC | CAGGCCGACTTACTCGATCC | 0.989785 CCAAGGCCACGGTTACTTCC | CAGGCCGACTTACTCGATCC | 1.044952 CATGAACCACCTCCGACACG | CAGGCCGACTTACTCGATCC | 1.161469 GTTACTTCCAGCGCATGAACC | CAGGCCGACTTACTCGATCC | 1.226921 GGAGAGGGAACAGTACAGGG | CAGGCCGACTTACTCGATCC | | | | | |
| NCU00126T0 | 2 | 5 | NCU00126T0\_input.txt | NCU00126T0\_output.txt | 0.570345 CGATCGAGTTGTTGGCTTGC | TCGCCGATCTCCAAACAAGG | 0.636670 ACCACGATCAACCCTGTACG | TCGCCGATCTCCAAACAAGG | 0.710967 CGATCGAGTTGTTGGCTTGC | GAAATCCGGGAGCTCTCAGC | 0.712783 AGCTTGACCACGATCAACCC | TCGCCGATCTCCAAACAAGG | 0.781834 GGCTTGCTCCCCTTATCTCG | GAAATCCGGGAGCTCTCAGC | | | | | |
| NCU00127T0 | 2 | 5 | NCU00127T0\_input.txt | NCU00127T0\_output.txt | 0.062030 TCGATTCCGATGAGCATGGG | GGGCTGATTCGACGATTTGC | 0.071663 GGTCTCTCACAGTTCCGTGG | AGCTGTTACTGTGGGTGACG | 0.075856 GGTCTCTCACAGTTCCGTGG | TGGGTGACGTTCCATCTTCG | 0.204481 TCGATTCCGATGAGCATGGG | ACGTTCCATCTTCGTCGAGG | 0.208720 GGTCTCTCACAGTTCCGTGG | ACGTTCCATCTTCGTCGAGG | | | | | |
| NCU00128T0 | 2 | 5 | NCU00128T0\_input.txt | NCU00128T0\_output.txt | 0.205765 GTACCTACGACGACGAACCC | CAGTGGTTCCTAGGCTGACG | 0.414310 GTACCTACGACGACGAACCC | TTAGGATGAGTATGGCGCCG | 0.489063 GGCATCAACATCAGTGGTGC | TTAGGATGAGTATGGCGCCG | 0.758090 CCTCTGTACCTACGACGACG | CAGTGGTTCCTAGGCTGACG | 0.769493 GTACCTACGACGACGAACCC | ACCATACCGCAGACGATACC | | | | | |
| NCU00129T0 | 3 | 5 | NCU00129T0\_input.txt | NCU00129T0\_output.txt | 0.423109 TACCGCCTCATCAAAAGGGC | CTATCGGGCGCATTGTTTGG | 0.564147 TACCGCCTCATCAAAAGGGC | GAGCGGTTATGGTGTTTGGC | 0.710324 TTGAGACCCTCCATTACCGC | GAGCGGTTATGGTGTTTGGC | 1.044908 CATCAAAAGGGCCGCAAAGC | CTATCGGGCGCATTGTTTGG | 1.185946 CATCAAAAGGGCCGCAAAGC | GAGCGGTTATGGTGTTTGGC | | | | | |
| NCU00130T0 | 3 | 5 | NCU00130T0\_input.txt | NCU00130T0\_output.txt | 0.490674 AGAATTTGCACTTACGCCGC | CTGGCTTGTTAGTGGGACCC | 0.490674 AGAATTTGCACTTACGCCGC | CCTGGCTTGTTAGTGGGACC | 0.787100 CGAAGCCCCAGAGGAAATCC | CTGGCTTGTTAGTGGGACCC | 0.787100 CGAAGCCCCAGAGGAAATCC | CCTGGCTTGTTAGTGGGACC | 0.911118 AGAATTTGCACTTACGCCGC | ACATATTGTGTCCGTGGGCG | | | | | |
| NCU00130T1 | 2 | 5 | NCU00130T1\_input.txt | NCU00130T1\_output.txt | 0.503896 ACCCTAGCTGCTATGCAACC | CTGGCTTGTTAGTGGGACCC | 0.503896 ACCCTAGCTGCTATGCAACC | CCTGGCTTGTTAGTGGGACC | 0.646362 CTGGGAAAGAAAGGGAGCCC | CTGGCTTGTTAGTGGGACCC | 0.646362 CTGGGAAAGAAAGGGAGCCC | CCTGGCTTGTTAGTGGGACC | 0.709747 CGGCTATCTGTCAGACTGGG | CTGGCTTGTTAGTGGGACCC | | | | | |
| NCU00132T0 | 2 | 5 | NCU00132T0\_input.txt | NCU00132T0\_output.txt | 0.557124 TGAAGCAGGCAAACAAACGG | GGGACGTGTCTACACCTAGC | 0.564231 GTGACGGAAGGAAGTCGAGG | GGGACGTGTCTACACCTAGC | 0.909625 CGAGTGACGGAAGGAAGTCG | GGGACGTGTCTACACCTAGC | 0.974082 GGCAACAAAGGAGAGCTTCG | GGGACGTGTCTACACCTAGC | 1.123540 TATGACGATCAGGGCAGACG | GGGACGTGTCTACACCTAGC | | | | | |
| NCU00133T0 | 5 | 5 | NCU00133T0\_input.txt | NCU00133T0\_output.txt | 0.977996 GTGGTGATGCAGTTCAAGGC | TTGATGCCGTACTGCTGACG | 1.190432 CACTTCATCAGGTTGTGGCG | TATGAGATGTACGTGGCCGG | 1.254614 CACTTCATCAGGTTGTGGCG | TTGATGCCGTACTGCTGACG | 1.344108 TGACCTGAACATCACCGAGG | TTGATGCCGTACTGCTGACG | 1.359151 AGTCACTACACCAGCCAAGG | TATGAGATGTACGTGGCCGG | | | | | |
| NCU00134T0 | 3 | 5 | NCU00134T0\_input.txt | NCU00134T0\_output.txt | 0.290332 CCTTGTGGGCAACATGTTCG | GCAGAATAGCTGGATGGGGG | 0.429843 GGATTTGGATTTGGCCCTGC | GCAGAATAGCTGGATGGGGG | 0.560568 GGATTTGGATTTGGCCCTGC | GCACGGGAGCAATGTATTGG | 0.635026 TTGCTCACAAAAGCGAACCC | GCAGAATAGCTGGATGGGGG | 0.765750 TTGCTCACAAAAGCGAACCC | GCACGGGAGCAATGTATTGG | | | | | |
| NCU00134T1 | 2 | 5 | NCU00134T1\_input.txt | NCU00134T1\_output.txt | 0.290332 CCTTGTGGGCAACATGTTCG | GCAGAATAGCTGGATGGGGG | 0.429843 GGATTTGGATTTGGCCCTGC | GCAGAATAGCTGGATGGGGG | 0.560568 GGATTTGGATTTGGCCCTGC | GCACGGGAGCAATGTATTGG | 0.635026 TTGCTCACAAAAGCGAACCC | GCAGAATAGCTGGATGGGGG | 0.765750 TTGCTCACAAAAGCGAACCC | GCACGGGAGCAATGTATTGG | | | | | |
| NCU00135T0 | 4 | 5 | NCU00135T0\_input.txt | NCU00135T0\_output.txt | 0.626713 GAGAAGGTCAGCCAGTTGGG | CAGCAACCTTAATAGGCGCG | 0.913836 AGGCGAGATGGTGGTTTTGG | CAGCAACCTTAATAGGCGCG | 0.994328 AGAGAAGGTCAGCCAGTTGG | CAGCAACCTTAATAGGCGCG | 1.045573 ATGGTGTGGATGTTCTCGCG | CAGCAACCTTAATAGGCGCG | 1.220328 GAGAAGGTCAGCCAGTTGGG | TTGCCCTGAGAGACTACAGC | | | | | |
| NCU00135T1 | 3 | 5 | NCU00135T1\_input.txt | NCU00135T1\_output.txt | 0.626713 GAGAAGGTCAGCCAGTTGGG | CAGCAACCTTAATAGGCGCG | 0.913836 AGGCGAGATGGTGGTTTTGG | CAGCAACCTTAATAGGCGCG | 0.994328 AGAGAAGGTCAGCCAGTTGG | CAGCAACCTTAATAGGCGCG | 1.045573 ATGGTGTGGATGTTCTCGCG | CAGCAACCTTAATAGGCGCG | 1.220328 GAGAAGGTCAGCCAGTTGGG | TTGCCCTGAGAGACTACAGC | | | | | |
| NCU00136T0 | 2 | 5 | NCU00136T0\_input.txt | NCU00136T0\_output.txt | 1.126932 GATCGCATGGAGGCAAAAGG | CTGACCTCTGACATGCTGGC | 1.126932 GATCGCATGGAGGCAAAAGG | GCTGACCTCTGACATGCTGG | 1.216519 GATCGCATGGAGGCAAAAGG | TAAGATGAGGGCTGCTGACC | 1.524888 GATCGCATGGAGGCAAAAGG | TGTGGATGCTAAGATGAGGGC | 1.585858 GATCGCATGGAGGCAAAAGG | CTAAGATGAGGGCTGCTGACC | | | | | |
| NCU00137T0 | 3 | 5 | NCU00137T0\_input.txt | NCU00137T0\_output.txt | 0.344993 CGTTGAAGGATGTTGACGGC | TCGCCATAGACAATGCAGGG | 0.567054 TGGTGCTGATGTGACGTACG | TCGCCATAGACAATGCAGGG | 0.702384 GATGTGAAATGGAACGGCGC | TCGCCATAGACAATGCAGGG | 0.775409 CTCCTCATCGCTCCAGTTCC | TGCAGAGCTTCCATGTGTCG | 0.783357 CGTTGAAGGATGTTGACGGC | AGACAATGCAGGGTCAGAGG | | | | | |
| NCU00137T1 | 2 | 5 | NCU00137T1\_input.txt | NCU00137T1\_output.txt | 0.772120 TTGAGGGCTTGACTGGTTGG | CTTTGGGTTTCAGCAGGACG | 0.787541 TCTAGAGGGGTTCCAGCTGG | TTCACGAATGGGCTCGATGG | 0.843362 TGAAGCTGTTGGAGGATCGG | CTTTGGGTTTCAGCAGGACG | 0.850464 TTGAGGGCTTGACTGGTTGG | TGCAGAGCTTCCATGTGTCG | 0.918799 TCTAGAGGGGTTCCAGCTGG | CTTTGGGTTTCAGCAGGACG | | | | | |
| NCU00138T0 | 4 | 5 | NCU00138T0\_input.txt | NCU00138T0\_output.txt | 0.213177 CCCGATGAGTCTGAACCACC | GGTATGAGAGCCTTCACCCG | 0.213177 CCCGATGAGTCTGAACCACC | CGGTATGAGAGCCTTCACCC | 0.213177 CCCGATGAGTCTGAACCACC | CCGGTATGAGAGCCTTCACC | 0.215277 CAAGTTCGCAATCGTGGTGG | GGTATGAGAGCCTTCACCCG | 0.215277 CAAGTTCGCAATCGTGGTGG | CGGTATGAGAGCCTTCACCC | | | | | |
| NCU00142T0 | 1 | 5 | NCU00142T0\_input.txt | NCU00142T0\_output.txt | 0.208607 CAGACGGAAGGGGGAATACG | ATTGGATAGTCGGTCGGTGC | 0.643973 AAAAAGGCAGACGGAAGGGG | ATTGGATAGTCGGTCGGTGC | 0.976770 ACACTCACACCACACCATCC | TATCCCCCTCCTCTTCCTCC | 1.072190 CAGACGGAAGGGGGAATACG | TTTGTGTGGTGGTGGTGTGG | 1.103851 CAACACACGTCATCAGCACC | TATCCCCCTCCTCTTCCTCC | | | | | |
| NCU00143T0 | 2 | 5 | NCU00143T0\_input.txt | NCU00143T0\_output.txt | 0.403216 GAACTCGGCATCGGAATTGC | GCTGCTATTCAGAACACGCC | 0.478860 TCACTCGCTCATCATCTGCC | GCTGCTATTCAGAACACGCC | 0.486653 GAACTCGGCATCGGAATTGC | TCGGCGAATCAGAAGAGTGC | 0.562298 TCACTCGCTCATCATCTGCC | TCGGCGAATCAGAAGAGTGC | 0.675520 GAACTCGGCATCGGAATTGC | CTGCTATTCAGAACACGCCG | | | | | |
| NCU00144T1 | 4 | 5 | NCU00144T1\_input.txt | NCU00144T1\_output.txt | 0.074530 TCAAATTTCCCGGCTTTGCG | TGTTCGGGTATGCCAAGACC | 0.214699 TCAAATTTCCCGGCTTTGCG | GAAGGCTGAGGGTACTTCGG | 0.214994 GTATTTGGGTTTCCGCTGCG | TGTTCGGGTATGCCAAGACC | 0.214994 CGTATTTGGGTTTCCGCTGC | TGTTCGGGTATGCCAAGACC | 0.277301 TTGCGTATTTGGGTTTCCGC | TGTTCGGGTATGCCAAGACC | | | | | |
| NCU00144T0 | 3 | 5 | NCU00144T0\_input.txt | NCU00144T0\_output.txt | 0.074530 TCAAATTTCCCGGCTTTGCG | TGTTCGGGTATGCCAAGACC | 0.214699 TCAAATTTCCCGGCTTTGCG | GAAGGCTGAGGGTACTTCGG | 0.214994 GTATTTGGGTTTCCGCTGCG | TGTTCGGGTATGCCAAGACC | 0.214994 CGTATTTGGGTTTCCGCTGC | TGTTCGGGTATGCCAAGACC | 0.277301 TTGCGTATTTGGGTTTCCGC | TGTTCGGGTATGCCAAGACC | | | | | |
| NCU00145T0 | 3 | 5 | NCU00145T0\_input.txt | NCU00145T0\_output.txt | 0.141350 AGTTGCGGATCTCCTTGAGC | TAGGAAGGGAGAGGACACGG | 0.354703 TGTAACGCCTCTTCTTGCCC | TAGGAAGGGAGAGGACACGG | 0.426729 AGTTGCGGATCTCCTTGAGC | GATATGTCCCGTGACTCCGC | 0.571593 TGTAACGCCTCTTCTTGCCC | TGTCTTCTTGCTCGGTTGGG | 0.571852 TGTAACGCCTCTTCTTGCCC | TTGCTCGGTTGGGTTGTAGG | | | | | |
| NCU00146T0 | 3 | 5 | NCU00146T0\_input.txt | NCU00146T0\_output.txt | 0.065771 GTATCCTCCTCTTCACGGCG | TACAACCGGCTTCCCATTCC | 0.204163 GTATCCTCCTCTTCACGGCG | CATTCCCCAATTCACACGCC | 0.204163 GTATCCTCCTCTTCACGGCG | CCATTCCCCAATTCACACGC | 0.425466 TGGTGGCGTCGTATCTTTGG | TACAACCGGCTTCCCATTCC | 0.482691 GTATCCTCCTCTTCACGGCG | CCCAATTCACACGCCTTAGC | | | | | |
| NCU00147T0 | 3 | 5 | NCU00147T0\_input.txt | NCU00147T0\_output.txt | 0.142857 TGTTTACGCCCTCGACATCC | TAGCTGGTTTGACGTGGACC | 0.150095 TGTTTACGCCCTCGACATCC | GTTGTCCTGAACGAAACCGC | 0.343404 TGTTTACGCCCTCGACATCC | CTGAACGAAACCGCAAGACC | 0.355371 AACCGGAAGTAAAGGACGCC | TAGCTGGTTTGACGTGGACC | 0.362610 AACCGGAAGTAAAGGACGCC | GTTGTCCTGAACGAAACCGC | | | | | |
| NCU00148T0 | 2 | 5 | NCU00148T0\_input.txt | NCU00148T0\_output.txt | 0.850918 TGGCGCTTCAGAGTACATCG | CAGCTCTTGACCAGGGAACG | 1.137025 TGGCGCTTCAGAGTACATCG | GGTTGAGGCAGCTCTTGACC | 1.282013 CCTTCACTTGGGCCTTCTGG | CAGCTCTTGACCAGGGAACG | 1.480536 ATCGAATTCAAACCACCGCC | CAGCTCTTGACCAGGGAACG | 1.635659 CACGGAAGCCCCTAGTTTGG | GGTCAATGACCCGGTTGACC | | | | | |
| NCU00149T0 | 1 | 5 | NCU00149T0\_input.txt | NCU00149T0\_output.txt | 0.144339 AAGTGAGGGATGGTCTTGCG | ACTTACAACCCATCTCCGCG | 0.204783 GATGTAAACGCAAGGGTCGC | ACTTACAACCCATCTCCGCG | 0.216416 AAGTGAGGGATGGTCTTGCG | AATGACATCCCTGTACCCGC | 0.272444 GATGTAAACGCAAGGGTCGC | CCGCCACTCCTTTACTCTCC | 0.276861 GATGTAAACGCAAGGGTCGC | AATGACATCCCTGTACCCGC | | | | | |
| NCU00150T0 | 3 | 5 | NCU00150T0\_input.txt | NCU00150T0\_output.txt | 0.059710 CTGGGAAAACTTGCCGTTGG | CATCGGAGACTCGGTGTACG | 0.140655 CTGGGAAAACTTGCCGTTGG | GCAACAACTAATGCAGGGGC | 0.203001 CTGGGAAAACTTGCCGTTGG | CAACAACTAATGCAGGGGCG | 0.203001 CTGGGAAAACTTGCCGTTGG | CGCAACAACTAATGCAGGGG | 0.203001 CTGGGAAAACTTGCCGTTGG | CCGCAACAACTAATGCAGGG | | | | | |
| NCU00150T1 | 4 | 5 | NCU00150T1\_input.txt | NCU00150T1\_output.txt | 0.131654 TGCCTTTTTGCAACCCTTCG | CATCGGAGACTCGGTGTACG | 1.050167 TGCAACCCTTCGTTATCGGC | CATCGGAGACTCGGTGTACG | 1.181225 AACCCTTCGTTATCGGCGG | CATCGGAGACTCGGTGTACG | 1.297969 ATGCCTTTTTGCAACCCTTCG | CATCGGAGACTCGGTGTACG | 1.424493 TGCCTTTTTGCAACCCTTCG | CGAGCACGTTTATTGAGACCG | | | | | |
| NCU00150T2 | 3 | 5 | NCU00150T2\_input.txt | NCU00150T2\_output.txt | 0.059710 CTGGGAAAACTTGCCGTTGG | CATCGGAGACTCGGTGTACG | 0.140655 CTGGGAAAACTTGCCGTTGG | GCAACAACTAATGCAGGGGC | 0.203001 CTGGGAAAACTTGCCGTTGG | CAACAACTAATGCAGGGGCG | 0.203001 CTGGGAAAACTTGCCGTTGG | CGCAACAACTAATGCAGGGG | 0.203001 CTGGGAAAACTTGCCGTTGG | CCGCAACAACTAATGCAGGG | | | | | |
| NCU00151T0 | 2 | 5 | NCU00151T0\_input.txt | NCU00151T0\_output.txt | 0.211775 ATGCGGTAGATTCAGGCTCG | GAGTTGGAGTCGAGGATGGC | 0.281958 ATGCGGTAGATTCAGGCTCG | GCCGTTTGTTTGGGAGTTGG | 0.287995 CAGCGACAGTGAAGAGGAGG | GAGTTGGAGTCGAGGATGGC | 0.358098 AAAGGACCCGTCAAAGGTGG | GAGTTGGAGTCGAGGATGGC | 0.358178 CAGCGACAGTGAAGAGGAGG | GCCGTTTGTTTGGGAGTTGG | | | | | |
| NCU00152T1 | 6 | 5 | NCU00152T1\_input.txt | NCU00152T1\_output.txt | 0.481193 CCGTTGTCCAAGGTCAAAGC | GCCGGTTGTTCTATGTTGCC | 0.831359 AAGCAAACCAAGTCCGTTCG | GCCGGTTGTTCTATGTTGCC | 1.189270 CCGTTGTCCAAGGTCAAAGC | TGCCGGTTGTTCTATGTTGC | 1.235291 AAAGCAAACCAAGTCCGTTCG | GCCGGTTGTTCTATGTTGCC | 1.439389 GGTCAAAGCAAACCAAGTCCG | GCCGGTTGTTCTATGTTGCC | | | | | |
| NCU00152T0 | 5 | 5 | NCU00152T0\_input.txt | NCU00152T0\_output.txt | 0.481193 CCGTTGTCCAAGGTCAAAGC | GCCGGTTGTTCTATGTTGCC | 0.831359 AAGCAAACCAAGTCCGTTCG | GCCGGTTGTTCTATGTTGCC | 1.189270 CCGTTGTCCAAGGTCAAAGC | TGCCGGTTGTTCTATGTTGC | 1.235291 AAAGCAAACCAAGTCCGTTCG | GCCGGTTGTTCTATGTTGCC | 1.439389 GGTCAAAGCAAACCAAGTCCG | GCCGGTTGTTCTATGTTGCC | | | | | |
| NCU00153T0 | 2 | 5 | NCU00153T0\_input.txt | NCU00153T0\_output.txt | 0.280373 GCTTCCATTACACGCTTCGC | ATGCGCTCATACTTCTCGGG | 0.406394 GGCTTCCATTACACGCTTCG | ATGCGCTCATACTTCTCGGG | 0.695831 GGATGGCTTCCATTACACGC | ATGCGCTCATACTTCTCGGG | 1.068996 GCTTCCATTACACGCTTCGC | GAAGGAATAGCCAGCCCAGC | 1.195017 GGCTTCCATTACACGCTTCG | GAAGGAATAGCCAGCCCAGC | | | | | |
| NCU00154T1 | 3 | 5 | NCU00154T1\_input.txt | NCU00154T1\_output.txt | 0.283228 GGGATGTCGTAGGTGACTGC | CATGGATGGCATTGCTGTGG | 0.502741 GGGATGTCGTAGGTGACTGC | TGGTTGTCAGGCATGGATGG | 0.697383 GATAGGCGTGGGTCGTTACG | CATGGATGGCATTGCTGTGG | 0.940521 ACCATAGGGATAGGCGTGGG | CATGGATGGCATTGCTGTGG | 1.002909 ATAGGGATAGGCGTGGGTCG | CATGGATGGCATTGCTGTGG | | | | | |
| NCU00154T0 | 5 | 5 | NCU00154T0\_input.txt | NCU00154T0\_output.txt | 0.283228 GGGATGTCGTAGGTGACTGC | CATGGATGGCATTGCTGTGG | 0.502741 GGGATGTCGTAGGTGACTGC | TGGTTGTCAGGCATGGATGG | 1.006197 GGGATGTCGTAGGTGACTGC | TGGTTGGTTGTCAGGCATGG | 1.320966 GGGATGTCGTAGGTGACTGC | GGGAGGCCTTCTTCTATGTGG | 1.530161 CGTTACGGCGGATCCAAAGG | CATGGATGGCATTGCTGTGG | | | | | |
| NCU00155T0 | 6 | 5 | NCU00155T0\_input.txt | NCU00155T0\_output.txt | 0.497409 ATTTGCACGTGGCCATATGC | ACCCGGCTTCTCATATCAGC | 1.721681 ATTTGCACGTGGCCATATGC | TACCCGGCTTCTCATATCAGC | 1.904948 TTTGCACGTGGCCATATGC | ACCCGGCTTCTCATATCAGC | 2.337752 ATTTGCACGTGGCCATATGC | ATACCCGGCTTCTCATATCAGC | 2.524121 CATTTGCACGTGGCCATATGC | ACCCGGCTTCTCATATCAGC | | | | | |
| NCU00157T0 | 5 | 5 | NCU00157T0\_input.txt | NCU00157T0\_output.txt | 0.287138 AGAAGGCCTTTAACGGACCG | TCTGGAGTGCCTTTGACTGC | 0.763419 CAAAGACGGGAAGCTGAACG | TCTGGAGTGCCTTTGACTGC | 0.775330 TCGAGGACGAGCTGTTTACC | TCTGGAGTGCCTTTGACTGC | 0.904612 CAAAGACGGGAAGCTGAACG | AGTGTCATCCGTGGTCATGC | 1.145567 TGGACACAATGCAGAAGGCC | TCTGGAGTGCCTTTGACTGC | | | | | |
| NCU00158T0 | 2 | 5 | NCU00158T0\_input.txt | NCU00158T0\_output.txt | 0.216508 AGCTTCAAGTCGGGTTACGG | TATTCGATCAGGGGAGGGGG | 0.562208 AGCTTCAAGTCGGGTTACGG | AAGCGGTGTTTGACGTTTGC | 0.717666 AGCTTCAAGTCGGGTTACGG | GATGGGTTAGTCTAGCGGGG | 0.782610 TGAGGCAAGCTTCAAGTCGG | TATTCGATCAGGGGAGGGGG | 0.800827 AGCTTCAAGTCGGGTTACGG | ATGGGTTAGTCTAGCGGGGG | | | | | |
| NCU00159T0 | 2 | 5 | NCU00159T0\_input.txt | NCU00159T0\_output.txt | 0.063874 TCCAGTCACAAGAGAACCGC | CGGGGATTTCGACGTAGAGG | 0.069514 TCCAGTCACAAGAGAACCGC | AAGAATGGGGTGACTGCTCG | 0.075988 CAGGCTCTACTATCGCACCG | AAGAATGGGGTGACTGCTCG | 0.134673 ACTCATCGATAACCCACCGC | CGGGGATTTCGACGTAGAGG | 0.140314 ACTCATCGATAACCCACCGC | AAGAATGGGGTGACTGCTCG | | | | | |
| NCU00160T0 | 4 | 5 | NCU00160T0\_input.txt | NCU00160T0\_output.txt | 0.563789 AGGCGAAAGTAATACCGGCG | AGCCAACATCTCCCTTCTGC | 0.688830 GAAAGTAATACCGGCGGTGC | AGCCAACATCTCCCTTCTGC | 0.776577 AGGCGAAAGTAATACCGGCG | GATCGCTTGAGTTCTTGCGC | 0.783297 AGGCGAAAGTAATACCGGCG | TCCCTTCTGCTCTTGATGCC | 0.845315 AGGCGAAAGTAATACCGGCG | GTTCTTGCGCGCATACATCC | | | | | |
| NCU00161T0 | 2 | 5 | NCU00161T0\_input.txt | NCU00161T0\_output.txt | 1.412282 TACCACCCGAACAAAGAGGC | AGAACTGCATCTCCATTGCG | 1.625491 GTCTCGGCGGTCTTATCACC | AGAACTGCATCTCCATTGCG | 1.777446 TACCACCCGAACAAAGAGGC | AAGAACTGCATCTCCATTGCG | 1.901359 CGGTCTTATCACCCGACACG | AGAACTGCATCTCCATTGCG | 1.990655 GTCTCGGCGGTCTTATCACC | AAGAACTGCATCTCCATTGCG | | | | | |
| NCU00161T1 | 3 | 5 | NCU00161T1\_input.txt | NCU00161T1\_output.txt | 1.412282 TACCACCCGAACAAAGAGGC | AGAACTGCATCTCCATTGCG | 1.625491 GTCTCGGCGGTCTTATCACC | AGAACTGCATCTCCATTGCG | 1.777446 TACCACCCGAACAAAGAGGC | AAGAACTGCATCTCCATTGCG | 1.901359 CGGTCTTATCACCCGACACG | AGAACTGCATCTCCATTGCG | 1.990655 GTCTCGGCGGTCTTATCACC | AAGAACTGCATCTCCATTGCG | | | | | |
| NCU00162T0 | 2 | 5 | NCU00162T0\_input.txt | NCU00162T0\_output.txt | 0.502198 TGCCATCTCGGAATGACAGC | TTGCATGGCGTTGTTACTGC | 1.598749 TGCCATCTCGGAATGACAGC | GGCGTTGTTACTGCGTTTAGG | 1.972665 CGAGAACGTTCAAGTGCTGC | GCGGACATCTCGAATTCTTCC | 1.974467 TGCCATCTCGGAATGACAGC | GCATGGCGTTGTTACTGCG | 2.041506 TGCCATCTCGGAATGACAGC | TGCATGGCGTTGTTACTGC | | | | | |
| NCU00163T0 | 3 | 5 | NCU00163T0\_input.txt | NCU00163T0\_output.txt | 0.214719 AGCTGGTCTTGCTCTATGCG | CTTGGCCAAGACCAGAGAGG | 0.217799 AGCTGGTCTTGCTCTATGCG | GAGAGGCGATCAGGATTCCG | 0.275885 GGATCATGTCGCAAGTTGCC | TGTGGTCGATGATGAGCAGG | 0.355538 AGCTGGTCTTGCTCTATGCG | TGTGGTCGATGATGAGCAGG | 0.356051 TGTAACGGAGCTGGTCTTGC | CTTGGCCAAGACCAGAGAGG | | | | | |
| NCU00164T0 | 8 | 5 | NCU00164T0\_input.txt | NCU00164T0\_output.txt | 0.140962 AAACCAAGGCGATAAGGGGG | CCTTAGCTGACTCCAAGGGC | 0.212613 AAACCAAGGCGATAAGGGGG | TCCGTCATCTTCACATCGCC | 0.286596 TTGTAGCGAGCAGATGACGG | CCTTAGCTGACTCCAAGGGC | 0.358248 TTGTAGCGAGCAGATGACGG | TCCGTCATCTTCACATCGCC | 0.494489 AAACCAAGGCGATAAGGGGG | CTTAGCTGACTCCAAGGGCG | | | | | |
| NCU00166T0 | 3 | 5 | NCU00166T0\_input.txt | NCU00166T0\_output.txt | 0.286404 CAAGAGAAGTTCCGCAACGC | CTGGGTGGTGACGTAGATGG | 0.430284 CAAGAGAAGTTCCGCAACGC | AGAGTTTTGTCGAGGTCGGC | 0.567023 GTCCATGGTTCACGAGACCC | CTGGGTGGTGACGTAGATGG | 0.686981 GTCAACGTCCATGGTTCACG | CTGGGTGGTGACGTAGATGG | 0.912085 CGTCCATGGTTCACGAGACC | CTGGGTGGTGACGTAGATGG | | | | | |
| NCU00167T0 | 3 | 5 | NCU00167T0\_input.txt | NCU00167T0\_output.txt | 0.551563 ACGGTCGACAACAAGTTTGC | CTGGGTGGTGACGTAGATGG | 0.770565 ACGGTCGACAACAAGTTTGC | TGATGCTTGTGAGATGGGGC | 0.858595 CGTTCTGGGCCCAATAGTCC | TGATGCTTGTGAGATGGGGC | 0.996072 GTGAGCGCCATCCTGTATCC | TGATGCTTGTGAGATGGGGC | 1.089206 TCTGGGCCCAATAGTCCTCC | TGATGCTTGTGAGATGGGGC | | | | | |
| NCU00168T0 | 3 | 5 | NCU00168T0\_input.txt | NCU00168T0\_output.txt | 0.139583 ATTGTGCCAGTTGTCGTTGC | ACGCTCGGGTACTTGTTAGC | 0.209749 ATTGTGCCAGTTGTCGTTGC | TCCTTAAGCATGAGGTCGCG | 0.286086 TGCCAACTATAGCCACGTCC | ACGCTCGGGTACTTGTTAGC | 0.356252 TGCCAACTATAGCCACGTCC | TCCTTAAGCATGAGGTCGCG | 0.358103 TTTCCACTTGGCTGTCCAGG | TCCTTAAGCATGAGGTCGCG | | | | | |
| NCU00169T0 | 2 | 5 | NCU00169T0\_input.txt | NCU00169T0\_output.txt | 0.417411 AAAGGTAAGGTGGTGCGACC | GAGCTGAATGTTCGTGTGCC | 0.419858 ACTGGTCGGATTGGTGATGG | GAGCTGAATGTTCGTGTGCC | 0.429837 AAAGGTAAGGTGGTGCGACC | CGAGCTGAATGTTCGTGTGC | 0.432284 ACTGGTCGGATTGGTGATGG | CGAGCTGAATGTTCGTGTGC | 0.631188 CTGGTCGGATTGGTGATGGG | GAGCTGAATGTTCGTGTGCC | | | | | |
| NCU00170T0 | 3 | 5 | NCU00170T0\_input.txt | NCU00170T0\_output.txt | 0.534753 GTGTAGATACGGGCCAGACG | CAGCGATTATTCCTGTCGCG | 0.539280 ATCCTTATACTTGCCGGCGG | CAGCGATTATTCCTGTCGCG | 0.755737 TGCAGTCCTCAAGAGCATCC | CAGCGATTATTCCTGTCGCG | 1.232195 GTGTAGATACGGGCCAGACG | GCGATTATTCCTGTCGCGC | 1.236721 ATCCTTATACTTGCCGGCGG | GCGATTATTCCTGTCGCGC | | | | | |
| NCU00171T0 | 3 | 5 | NCU00171T0\_input.txt | NCU00171T0\_output.txt | 0.431966 AGTGGGGATGGTTTGTACGG | GACGGGCTGGATAAGTCTGG | 0.505989 AGTGGGGATGGTTTGTACGG | GCAGGCTCTGTGATACTGGG | 0.505989 AGTGGGGATGGTTTGTACGG | GGCAGGCTCTGTGATACTGG | 0.944339 AGTGGGGATGGTTTGTACGG | AATCAGGACAGGCAGACAGG | 1.106927 TGGGGATGGTTTGTACGGC | GACGGGCTGGATAAGTCTGG | | | | | |
| NCU00172T0 | 3 | 5 | NCU00172T0\_input.txt | NCU00172T0\_output.txt | 0.412599 ATGAGCACGACAACTACCCG | TCGTGTATAGCTGAGCGACG | 0.574082 ATGAGCACGACAACTACCCG | GGAGATCCAGGCCTTTGAGC | 0.614339 TCAAGTTCACACGCATTGCC | TCGTGTATAGCTGAGCGACG | 0.624217 CACACCACCAACTCTACCGG | TCGTGTATAGCTGAGCGACG | 0.687487 ATGAGCACGACAACTACCCG | GTCGATCGTGCATGTGATGG | | | | | |
| NCU00173T0 | 2 | 5 | NCU00173T0\_input.txt | NCU00173T0\_output.txt | 0.073733 AGTGGTTGTAGGCTTCCAGC | AAGAGAAACTCTCCGGCAGC | 0.719084 AGTGGTTGTAGGCTTCCAGC | TAACTCGTCCTCCTTCCCCG | 0.720038 ACCCCGCTTGGATAAAAGGC | AAGAGAAACTCTCCGGCAGC | 1.012932 ATCAGGTAGGTCACACCCCG | AAGAGAAACTCTCCGGCAGC | 1.139837 CACCCCGCTTGGATAAAAGG | AAGAGAAACTCTCCGGCAGC | | | | | |
| NCU00175T0 | 3 | 5 | NCU00175T0\_input.txt | NCU00175T0\_output.txt | 0.428124 ACCTTTGGTGATCGTGGTCG | TGATGGATGACTTCACGGCC | 0.640321 ACCTTTGGTGATCGTGGTCG | CAGGTTGCTATCGGATCCGG | 0.640321 ACCTTTGGTGATCGTGGTCG | CCAGGTTGCTATCGGATCCG | 0.772204 ACCTTTGGTGATCGTGGTCG | GGATTGGTTAACTGGCTGCG | 0.772771 TGGTGATCGTGGTCGTTTCG | TGATGGATGACTTCACGGCC | | | | | |
| NCU00176T0 | 3 | 5 | NCU00176T0\_input.txt | NCU00176T0\_output.txt | 0.425110 CAACCTCTGAACTCGACCCC | GTAGCGGATCCAGAGCATCG | 0.425110 CCAACCTCTGAACTCGACCC | GTAGCGGATCCAGAGCATCG | 0.637116 CTGACTGCTACCGAGGATGC | GTAGCGGATCCAGAGCATCG | 0.710877 CAACCTCTGAACTCGACCCC | AGCATCGTGAAACCTTCGGG | 0.710877 CCAACCTCTGAACTCGACCC | AGCATCGTGAAACCTTCGGG | | | | | |
| NCU00177T0 | 1 | 5 | NCU00177T0\_input.txt | NCU00177T0\_output.txt | 0.205394 GGTATGGTTTTGGCTGTCGC | TTTAAGCAGCCCAGCTCTCC | 0.271558 GGTATGGTTTTGGCTGTCGC | CCGAATTTAAGCAGCCCAGC | 0.281142 GTGGCTCAGAGAGTCTGTGG | TTTAAGCAGCCCAGCTCTCC | 0.284061 GTGGCTCAGAGAGTCTGTGG | GTTGACCAGCTTGCCAATGG | 0.347306 GTGGCTCAGAGAGTCTGTGG | CCGAATTTAAGCAGCCCAGC | | | | | |
| NCU00178T0 | 2 | 5 | NCU00178T0\_input.txt | NCU00178T0\_output.txt | 0.356905 CACACAGTCACTGCGTTTGG | TCATTAACCCGTCCCAACCC | 0.370764 GAAAGCACACAGTCACTGCG | TCATTAACCCGTCCCAACCC | 0.370764 CGAAAGCACACAGTCACTGC | TCATTAACCCGTCCCAACCC | 0.578248 ACGAAACGGCGGAGATATGG | TCATTAACCCGTCCCAACCC | 0.931831 GAAACGGCGGAGATATGGGG | TCATTAACCCGTCCCAACCC | | | | | |
| NCU00179T0 | 3 | 5 | NCU00179T0\_input.txt | NCU00179T0\_output.txt | 0.420707 GAACCAGGAAGAGTGGCTCG | TAAGCATGTCCGTCCGATCG | 0.486457 ATCTTCAGTACGCCCACTCG | TAAGCATGTCCGTCCGATCG | 0.489726 ATCTTCAGTACGCCCACTCG | TTGTCTGAAGACTGGCGACC | 0.699860 AACGCCTACTGCTTTCGACC | TAAGCATGTCCGTCCGATCG | 0.702016 CAGCGAACCAGGAAGAGTGG | TAAGCATGTCCGTCCGATCG | | | | | |
| NCU00180T0 | 2 | 5 | NCU00180T0\_input.txt | NCU00180T0\_output.txt | 0.435604 CACAAGCTGAACACCAAGGC | AAGGTTTCTGTACCCAGGGC | 0.706073 CACAAGCTGAACACCAAGGC | CCAGGGCACTCCTCTTTTCG | 0.851252 CACAAGCTGAACACCAAGGC | AGCCCGGTACTCTCTGATCG | 0.915882 CACAAGCTGAACACCAAGGC | TTTCGCTTCTGACACTGGCC | 0.975564 CACAAGCTGAACACCAAGGC | TTTTCGCTTCTGACACTGGC | | | | | |
| NCU00181T0 | 1 | 5 | NCU00181T0\_input.txt | NCU00181T0\_output.txt | 0.288334 AAGACGTTCGCATAGGAGCC | GACTCGGACGAGAACAAGGG | 0.358681 GTTTCTTGAAGCTGGCACCG | CGCAAAACACCACCATCACC | 0.429100 TTGAAGGGTTGGAGACTGGC | CGCAAAACACCACCATCACC | 0.482942 GGCAAAGACGTTCGCATAGG | GACTCGGACGAGAACAAGGG | 0.509211 GTTTCTTGAAGCTGGCACCG | AACACCACCATCACCAACCC | | | | | |
| NCU00182T0 | 3 | 5 | NCU00182T0\_input.txt | NCU00182T0\_output.txt | 0.550878 GGGCTTCCTCTACTTCCAGC | GAAGGAGAATGAGTTGCGCG | 0.574544 GGGCTTCCTCTACTTCCAGC | AGCCCATGCAGACAATCAGG | 0.962815 CTTGTACGCCTTGGTGTTGG | GAAGGAGAATGAGTTGCGCG | 0.971899 CACCGGTCGGATATCTTGGC | GAAGGAGAATGAGTTGCGCG | 0.971899 GCACCGGTCGGATATCTTGG | GAAGGAGAATGAGTTGCGCG | | | | | |
| NCU00183T0 | 4 | 5 | NCU00183T0\_input.txt | NCU00183T0\_output.txt | 0.287632 AACCTTCGACTCGCTCATGG | AACTGTGTCCTGAACCCAGC | 0.358126 ATAGCCAACCTTCGACTCGC | AACTGTGTCCTGAACCCAGC | 0.570163 TAAGCTGAGGACTGGCTTCG | TGGTTTTGGAGGGGATGTCG | 0.585621 TGGGCCATTCTAAGCTGAGG | TGGTTTTGGAGGGGATGTCG | 0.711591 TAAGCTGAGGACTGGCTTCG | AACTGTGTCCTGAACCCAGC | | | | | |
| NCU00184T0 | 2 | 5 | NCU00184T0\_input.txt | NCU00184T0\_output.txt | 0.215579 TAACCCGACTTCTCAACCGC | CCTGCTCTAGCTTGGTGAGG | 0.421240 CCGCCTTCAACAAACCATCC | CCTGCTCTAGCTTGGTGAGG | 0.491276 TTCAACAAACCATCCTGCGC | CCTGCTCTAGCTTGGTGAGG | 0.496864 TAACCCGACTTCTCAACCGC | TCGCTTCCGTACCAAATCGG | 0.568719 TAACCCGACTTCTCAACCGC | TCTCGTTCTTCCGGACATGG | | | | | |
| NCU00185T0 | 5 | 5 | NCU00185T0\_input.txt | NCU00185T0\_output.txt | 0.774410 GAAAATCACCCGGGGTTTCG | ATGCTGCCCACACAAAAAGC | 1.117542 GAAAATCACCCGGGGTTTCG | CAAAAAGCAGACTCCACCGG | 1.208378 TTCCCGCTGAAAATCACCCG | ATGCTGCCCACACAAAAAGC | 1.250951 GCCCACGTGGTATAGATGAGG | ATGCTGCCCACACAAAAAGC | 1.298720 TAGATGAGGGAGGGCGTTGG | ATGCTGCCCACACAAAAAGC | | | | | |
| NCU00186T0 | 2 | 5 | NCU00186T0\_input.txt | NCU00186T0\_output.txt | 0.221486 ATCAATTGCAACGACACGGC | CGTCGCTCACACTTTTGTCG | 0.275237 ACGTTCGTCTCTACACCACG | AACTCCTGCGTTCCAGTACG | 0.276288 AGAGTCGAAGTTCACGGACG | AACTCCTGCGTTCCAGTACG | 0.349466 AGAGTCGAAGTTCACGGACG | CGTCGCTCACACTTTTGTCG | 0.349466 AGTCCTCGAAGTCGTTCACG | CGTCGCTCACACTTTTGTCG | | | | | |
| NCU00187T0 | 2 | 5 | NCU00187T0\_input.txt | NCU00187T0\_output.txt | 0.279445 TTCAAGATGGTCTCTCCGCG | TCGATGTCGGCAATTAGGGG | 0.418379 GATCTCATCGTCTGTCCCGG | TCGATGTCGGCAATTAGGGG | 0.565408 TTCAAGATGGTCTCTCCGCG | TGTCGGCAATTAGGGGAACG | 0.704342 GATCTCATCGTCTGTCCCGG | TGTCGGCAATTAGGGGAACG | 0.987491 TTCAAGATGGTCTCTCCGCG | CAATTAGGGGAACGGAGCGG | | | | | |
| NCU00188T0 | 3 | 5 | NCU00188T0\_input.txt | NCU00188T0\_output.txt | 0.572263 TGGAGCGCGACAATATGAGG | TCATGGTCTTTCAGCCTGCC | 0.572557 TCTTATTTAGGCGGGCGAGC | TCATGGTCTTTCAGCCTGCC | 0.709032 TGGAGCGCGACAATATGAGG | TCATTTCGTCGGTGGCTAGC | 0.909618 GCTTCTTATTTAGGCGGGCG | TCATGGTCTTTCAGCCTGCC | 1.051941 TGGAGCGCGACAATATGAGG | ATTGTCATTTCGTCGGTGGC | | | | | |
| NCU00189T0 | 2 | 5 | NCU00189T0\_input.txt | NCU00189T0\_output.txt | 0.146394 TGGTACTTAGCAGAGCACGC | TTGGTGTAGGATGTGTGCCC | 0.342295 GGCGATAACAATCACGCTGG | TTGGTGTAGGATGTGTGCCC | 0.353786 GCGATAACAATCACGCTGGC | TTGGTGTAGGATGTGTGCCC | 0.428853 ACGCTGGCTTGGTACTTAGC | TTGGTGTAGGATGTGTGCCC | 0.569122 ATCTTGCTCATTCTCGGCGG | TTGGTGTAGGATGTGTGCCC | | | | | |
| NCU00192T0 | 2 | 5 | NCU00192T0\_input.txt | NCU00192T0\_output.txt | 0.073043 ACATCACAGGCACAGGAAGG | TTAGGTTGTCCCGGTTGAGC | 0.144069 TACTTCTTGTGGCCGCTAGC | TTAGGTTGTCCCGGTTGAGC | 0.284197 CACAGTCAACAACAACGCCC | TTAGGTTGTCCCGGTTGAGC | 0.421972 ACATCACAGGCACAGGAAGG | ATAGTAGCGGTCGTCTTGGC | 0.492998 TACTTCTTGTGGCCGCTAGC | ATAGTAGCGGTCGTCTTGGC | | | | | |
| NCU00193T0 | 2 | 5 | NCU00193T0\_input.txt | NCU00193T0\_output.txt | 0.357306 GTTGGAGGTCGACAGGAAGG | TGTTTTGAGTAGGTCGGGCG | 0.556310 GTTGGAGGTCGACAGGAAGG | GGCGCAAGAGAACAAGAAGG | 0.558604 GTGTTTTGGTCGTGATGCCC | GGCGCAAGAGAACAAGAAGG | 0.698106 ACCTGGGAGTGTTTTGGTCG | GGCGCAAGAGAACAAGAAGG | 0.700386 GTTGGAGGTCGACAGGAAGG | GGTGGTTGTGTGAGAAGTGC | | | | | |
| NCU00194T1 | 3 | 5 | NCU00194T1\_input.txt | NCU00194T1\_output.txt | 0.134839 GGTAGAGAAGGCCGAGAAGC | GTGATGACTAGCGGAGGAGC | 0.276171 GCAAGGGACTGTTGAATGCC | GTGATGACTAGCGGAGGAGC | 0.276171 GGCAAGGGACTGTTGAATGC | GTGATGACTAGCGGAGGAGC | 0.276307 GCAAGGGACTGTTGAATGCC | TCGGACTTCGTGACAGTTGG | 0.276307 GGCAAGGGACTGTTGAATGC | TCGGACTTCGTGACAGTTGG | | | | | |
| NCU00194T0 | 4 | 5 | NCU00194T0\_input.txt | NCU00194T0\_output.txt | 0.134839 GGTAGAGAAGGCCGAGAAGC | GTGATGACTAGCGGAGGAGC | 0.276171 GCAAGGGACTGTTGAATGCC | GTGATGACTAGCGGAGGAGC | 0.276171 GGCAAGGGACTGTTGAATGC | GTGATGACTAGCGGAGGAGC | 0.276307 GCAAGGGACTGTTGAATGCC | TCGGACTTCGTGACAGTTGG | 0.276307 GGCAAGGGACTGTTGAATGC | TCGGACTTCGTGACAGTTGG | | | | | |
| NCU00195T0 | 3 | 5 | NCU00195T0\_input.txt | NCU00195T0\_output.txt | 1.830863 CGCGGTGTCAACATGTATCG | GGCTCATACGTACAGACACCG | 2.047159 CGCGGTGTCAACATGTATCG | AGGCTCATACGTACAGACACC | 2.121353 CGCGGTGTCAACATGTATCG | GCTCATACGTACAGACACCG | 2.309472 CGCGGTGTCAACATGTATCG | TCATACGTACAGACACCGAGG | 2.329332 CGCGGTGTCAACATGTATCG | CTCATACGTACAGACACCGAGG | | | | | |
| NCU00196T2 | 3 | 5 | NCU00196T2\_input.txt | NCU00196T2\_output.txt | 0.770099 AAAGATGCCCTCAACGTCGG | GACTTTGTTGCGCGGATACC | 0.818172 GAAAGATGCCCTCAACGTCG | GACTTTGTTGCGCGGATACC | 0.818172 GACGAAAGATGCCCTCAACG | GACTTTGTTGCGCGGATACC | 1.145615 AAAGATGCCCTCAACGTCGG | ACCCCATCCAGAGATACCCG | 1.175581 AAGATGCCCTCAACGTCGG | GACTTTGTTGCGCGGATACC | | | | | |
| NCU00196T0 | 3 | 5 | NCU00196T0\_input.txt | NCU00196T0\_output.txt | 0.295095 CATTTGGCAACCACGATGGG | TGTACTTTGGGGTGGTTGGG | 0.428880 TTGATGCTATCGCGAAGGGG | TGGAGAACCGGTTGTTTCCC | 0.436255 TTGATGCTATCGCGAAGGGG | TGTACTTTGGGGTGGTTGGG | 0.510343 GGGGGATGCTATACGAACCC | TGTACTTTGGGGTGGTTGGG | 0.572363 CATTTGGCAACCACGATGGG | TAATGCTCGGCCACACATCC | | | | | |
| NCU00196T1 | 2 | 5 | NCU00196T1\_input.txt | NCU00196T1\_output.txt | 0.582447 CTATCTCCTGCAAGAGGGCC | TGTACTTTGGGGTGGTTGGG | 0.635774 CTTGTTTGCTGAGATCGGGC | TGTACTTTGGGGTGGTTGGG | 0.859714 CTATCTCCTGCAAGAGGGCC | TAATGCTCGGCCACACATCC | 0.937123 CTATCTCCTGCAAGAGGGCC | GTACTTTGGGGTGGTTGGGG | 0.990451 CTTGTTTGCTGAGATCGGGC | GTACTTTGGGGTGGTTGGGG | | | | | |
| NCU00197T0 | 3 | 5 | NCU00197T0\_input.txt | NCU00197T0\_output.txt | 0.498421 GCTCGCACGAATCATGTTCC | CATGATCCCCTCTCCACACC | 0.511235 TTCCCCAAGGCACAAGATCC | CATGATCCCCTCTCCACACC | 0.565854 GCTCGCACGAATCATGTTCC | CCTCTCCACACCAGCTAAGG | 0.578669 TTCCCCAAGGCACAAGATCC | CCTCTCCACACCAGCTAAGG | 0.910174 GCTCGCACGAATCATGTTCC | AAGTGGAAAGGGAACGACGC | | | | | |
| NCU00198T0 | 3 | 5 | NCU00198T0\_input.txt | NCU00198T0\_output.txt | 1.058563 AAACAGACTTACAGCCGCCG | TGGAGGTGAAATCGTCGACG | 1.176518 GCAAGGGAAACAACAGTCACC | TGGAGGTGAAATCGTCGACG | 1.178686 CAAAACAGACTTACAGCCGCC | TGGAGGTGAAATCGTCGACG | 1.269788 AAACAGACTTACAGCCGCCG | TGGACATGGACTTGAGCACG | 1.345182 AAAACAGACTTACAGCCGCC | TGGAGGTGAAATCGTCGACG | | | | | |
| NCU00199T0 | 2 | 5 | NCU00199T0\_input.txt | NCU00199T0\_output.txt | 0.288137 TCCGTTGGTTGGTAGGTTGG | GCGTGATTGCCGGTTTTAGG | 0.352328 TCCGTTGGTTGGTAGGTTGG | TGATGTTTGGGCGTGATTGC | 0.358230 TTGGGCGGATTCGAGATTCC | GCGTGATTGCCGGTTTTAGG | 0.422422 TTGGGCGGATTCGAGATTCC | TGATGTTTGGGCGTGATTGC | 0.498471 CCTTCAGTTTGCTTGGCTGC | GCGTGATTGCCGGTTTTAGG | | | | | |
| NCU00200T1 | 1 | 5 | NCU00200T1\_input.txt | NCU00200T1\_output.txt | 0.342389 TTGTCGACAAAGCAGCAACG | CATGGTTGGGCAATATCGGC | 0.348993 AACGAGACAGCAACTCCTGG | CATGGTTGGGCAATATCGGC | 0.421745 AGCTTACGGTGCGGTTAAGG | AGGGGTCAGTCAGTATCCCC | 0.498872 AGCTTACGGTGCGGTTAAGG | GAACACTGAGGATGTCCCGG | 0.565272 GACTTCCACACCCTGACACC | CATGGTTGGGCAATATCGGC | | | | | |
| NCU00200T0 | 2 | 5 | NCU00200T0\_input.txt | NCU00200T0\_output.txt | 0.407904 TGTTCTAACGCCACTCCTCG | CGAAACACGAATGAACCCCG | 0.417611 TCACAAAAGACACCTTGCGC | GGTGTTCCTGAAGGAGTCCG | 0.489511 TCACAAAAGACACCTTGCGC | GCAGCTGTGGTGTAAATGCC | 0.545457 TCACAAAAGACACCTTGCGC | CGAAACACGAATGAACCCCG | 0.623104 GTTCTAACGCCACTCCTCGG | CGAAACACGAATGAACCCCG | | | | | |
| NCU00201T0 | 2 | 5 | NCU00201T0\_input.txt | NCU00201T0\_output.txt | 0.353922 GAACAGCGTGACCAAAACCG | CGTCCTTCACAGAACCCTCC | 0.353922 CGAACAGCGTGACCAAAACC | CGTCCTTCACAGAACCCTCC | 0.419170 GAACAGCGTGACCAAAACCG | TCCTAAGTCTCGCAATCCGC | 0.419170 CGAACAGCGTGACCAAAACC | TCCTAAGTCTCGCAATCCGC | 0.641962 GAACAGCGTGACCAAAACCG | GTCCTTCACAGAACCCTCCC | | | | | |
| NCU00202T0 | 7 | 5 | NCU00202T0\_input.txt | NCU00202T0\_output.txt | 0.268989 CGGGTTGCGACAAACTTACC | AAGGCAAGCAAAGGAAAGCG | 0.272685 GCGACAAACTTACCCCTTGC | AAGGCAAGCAAAGGAAAGCG | 0.491209 TCGTTCCTATCTCGGGTTGC | AAGGCAAGCAAAGGAAAGCG | 0.494724 GAATGTTGCCATCACTGCGG | ACTGTTCGTGTGAGCTCTCC | 0.552017 CGGGTTGCGACAAACTTACC | ACTGTTCGTGTGAGCTCTCC | | | | | |
| NCU00203T0 | 1 | 5 | NCU00203T0\_input.txt | NCU00203T0\_output.txt | 0.336852 AGATCTTGGGCGTTGTCTCG | CGAACGCGAAGAGATCATGC | 0.345063 AGATCTTGGGCGTTGTCTCG | GGTTCGTTAAACGCAGGTGG | 0.347318 TGGGGTTGTTCCTTTACGGG | GGTTCGTTAAACGCAGGTGG | 0.499474 AGATCTTGGGCGTTGTCTCG | ATGATACGGAGAGCACAGGC | 0.501729 TGGGGTTGTTCCTTTACGGG | ATGATACGGAGAGCACAGGC | | | | | |
| NCU00204T0 | 2 | 5 | NCU00204T0\_input.txt | NCU00204T0\_output.txt | 0.218428 ACGGATCAGAGCAGTTGTCG | ATTCGGATCTGATGGGTGGC | 0.359637 TGTTGCGGGAGAGAAACTGG | ATTCGGATCTGATGGGTGGC | 0.903195 GAAGACGACGGACTCGAAGC | ATTCGGATCTGATGGGTGGC | 0.981064 TCGAAATCAAGTGTTGCGGG | ATTCGGATCTGATGGGTGGC | 1.048250 TCGGGCAAAAAGTTCACTCG | CAGGTATGATGGTGGTCGGG | | | | | |
| NCU00205T0 | 1 | 5 | NCU00205T0\_input.txt | NCU00205T0\_output.txt | 0.072332 GAGGTTGTTGGAGGACTGGG | CACCCTCATCCGCCTTATCC | 0.072332 GGAGGTTGTTGGAGGACTGG | CACCCTCATCCGCCTTATCC | 0.072885 GAGGTTGTTGGAGGACTGGG | CCATATCACCCTCATCCGCC | 0.072885 GGAGGTTGTTGGAGGACTGG | CCATATCACCCTCATCCGCC | 0.143042 GAGGTTGTTGGAGGACTGGG | GCCTTATCCAACCCCTTCCC | | | | | |
| NCU00206T0 | 4 | 5 | NCU00206T0\_input.txt | NCU00206T0\_output.txt | 0.140472 TCTGAGAGAAACACCGCACC | GGAGTTCAGAGAATGGGGGC | 0.496263 TCTGAGAGAAACACCGCACC | GAGTTCAGAGAATGGGGGCG | 0.582014 TCTGAGAGAAACACCGCACC | GGGAGTTCAGAGAATGGGGG | 0.925007 AGAGAAACACCGCACCAACC | GGAGTTCAGAGAATGGGGGC | 1.123620 GCCTCTGAGAGAAACACCGC | GGAGTTCAGAGAATGGGGGC | | | | | |
| NCU00207T1 | 2 | 5 | NCU00207T1\_input.txt | NCU00207T1\_output.txt | 0.712105 AGATCCCGCAACTTCATCGG | GTGGAGGGTTCGAAGAGAGG | 0.984815 GTGAATCCGCACCAATGTCC | GTGGAGGGTTCGAAGAGAGG | 1.190872 CCGCAACTTCATCGGATTCC | GTGGAGGGTTCGAAGAGAGG | 1.190896 GCCATTGGTGCTCTACATCG | AAGACGTTTTCCCTCCAGCC | 1.212673 TGAATCCGCACCAATGTCCC | GTGGAGGGTTCGAAGAGAGG | | | | | |
| NCU00207T0 | 4 | 5 | NCU00207T0\_input.txt | NCU00207T0\_output.txt | 0.771811 GCAGGCATCACTCACTCTGG | TGTCGATCATCTCTGCGTCG | 0.842568 ATCACTCACTCTGGACTCGC | TGTCGATCATCTCTGCGTCG | 0.850718 GCAGGCATCACTCACTCTGG | TAGGCCTTGCTTGGATCAGC | 1.129049 GCAGGCATCACTCACTCTGG | CGATCATCTCTGCGTCGAGG | 1.199805 ATCACTCACTCTGGACTCGC | CGATCATCTCTGCGTCGAGG | | | | | |
| NCU00208T0 | 3 | 5 | NCU00208T0\_input.txt | NCU00208T0\_output.txt | 0.143822 AGTTCTCCTTGGTCTGCAGC | GTTCTCTTCCCCGCCTTACC | 0.216429 AGTTCTCCTTGGTCTGCAGC | ATTCCCCTCCCAAATCGACG | 0.430775 ACCATCAGTGAGTGGAGTGC | GTTCTCTTCCCCGCCTTACC | 0.497748 CCCGTTTCGACCAGAAGAGG | GTTCTCTTCCCCGCCTTACC | 0.499961 TAACTCGGTGTCTGTGCTGC | ATTCCCCTCCCAAATCGACG | | | | | |
| NCU00209T0 | 2 | 5 | NCU00209T0\_input.txt | NCU00209T0\_output.txt | 1.039537 CTATGTAGGTAGGCAGGCGC | CTTCGGCAGGTTCTTGTTTGG | 1.039537 GCTATGTAGGTAGGCAGGCG | CTTCGGCAGGTTCTTGTTTGG | 1.067064 CTATGTAGGTAGGCAGGCGC | TTCGGCAGGTTCTTGTTTGG | 1.067064 GCTATGTAGGTAGGCAGGCG | TTCGGCAGGTTCTTGTTTGG | 1.318149 CGCACTATGGTCGAGGTACG | CTTCGGCAGGTTCTTGTTTGG | | | | | |
| NCU00210T0 | 3 | 5 | NCU00210T0\_input.txt | NCU00210T0\_output.txt | 0.553325 GTAACATGCATCCCGAAGCG | GCCGCCAATGTACTTCATGC | 0.691906 GTAACATGCATCCCGAAGCG | AATCGAGAGCGTCCTGATGG | 0.766044 GTAACATGCATCCCGAAGCG | TTTGTAAGCGATCGAGGCCC | 0.833171 GTAACATGCATCCCGAAGCG | TCATGCAATCGAGAGCGTCC | 0.895437 GTAACATGCATCCCGAAGCG | CTTGATCTCGCCGCATTTGC | | | | | |
| NCU00211T0 | 3 | 5 | NCU00211T0\_input.txt | NCU00211T0\_output.txt | 1.171633 GACGGTCTCACCCTTCTTGG | AGAATGTGTGGAGCCATACCG | 1.336773 GACGGTCTCACCCTTCTTGG | GAATGTGTGGAGCCATACCG | 1.385302 TCACCCTTCTTGGCAAGAGC | AGAATGTGTGGAGCCATACCG | 1.418633 TCTGGAACTTGGACATGCGC | ATCATGGACACTCCTGCTCG | 1.498705 TCTGGAACTTGGACATGCGC | TCCATCATGGACACTCCTGC | | | | | |
| NCU00212T0 | 3 | 5 | NCU00212T0\_input.txt | NCU00212T0\_output.txt | 0.990997 GGTGGAAAGATTGGGAGGGG | GTGTCTCCTCAGCTTTGGCC | 1.083028 TAGCGGGCTTGGTAATACCC | TTCAATTCCAGCTGTCCCCG | 1.102313 GGTGGAAAGATTGGGAGGGG | TGATAATCCAGCGTCCTCTGC | 1.327769 GGTGGAAAGATTGGGAGGGG | CGTGTCTCCTCAGCTTTGGC | 1.327769 GGTGGAAAGATTGGGAGGGG | GCGTGTCTCCTCAGCTTTGG | | | | | |
| NCU00213T0 | 2 | 5 | NCU00213T0\_input.txt | NCU00213T0\_output.txt | 0.766844 CTGGCTGTCAGTCTCGTTCG | AGACCAGAATGAGTTGGCGG | 0.821989 CTTGATTTGCTTGTTGCGGC | AGACCAGAATGAGTTGGCGG | 0.852146 TTGTGGTTAGGTGCGGTAGC | CTCACTACTGGGTTCGGACC | 0.943061 AGGGTGAGACTTTGGTTGGG | GGAGGCCTGGTTATGTCTGC | 1.036395 TCCCTTGATTTGCTTGTTGCG | AGACCAGAATGAGTTGGCGG | | | | | |
| NCU00214T0 | 1 | 5 | NCU00214T0\_input.txt | NCU00214T0\_output.txt | 0.211455 CTGGATCTTCTGGACCGTCG | CCACTTTAAGCAGCAGCACG | 0.292592 CGTGCTGCTGCTTAAAGTGG | TGATCCGGTGCTAATTGGGG | 0.424554 GTGCTGCTGCTTAAAGTGGG | TGATCCGGTGCTAATTGGGG | 0.429610 ACATGTTCGACGTGGTCTGG | CCACTTTAAGCAGCAGCACG | 0.432064 CGTGCTGCTGCTTAAAGTGG | GGACCAAGGAGCAAGAGTGG | | | | | |
| NCU00215T0 | 3 | 5 | NCU00215T0\_input.txt | NCU00215T0\_output.txt | 0.214953 TCCTCCTCCTCCTTTACCGG | TGATCCGGTGCTAATTGGGG | 0.290191 CAGCCGTAAGAGGAAAGGGG | TGATCCGGTGCTAATTGGGG | 0.354424 TCCTCCTCCTCCTTTACCGG | GGACCAAGGAGCAAGAGTGG | 0.358189 CTTTCCGGGTTTCGCAATCG | AGGTGGTGAGGGAAAAGTGC | 0.361736 CTTTCCGGGTTTCGCAATCG | TGATCCGGTGCTAATTGGGG | | | | | |
| NCU00215T1 | 2 | 5 | NCU00215T1\_input.txt | NCU00215T1\_output.txt | 0.494952 CTGATCTCGGGCCTAAGACG | TGATCCGGTGCTAATTGGGG | 0.634424 CTGATCTCGGGCCTAAGACG | GGACCAAGGAGCAAGAGTGG | 0.715621 GGGCCTAAGACGCTGATTCC | TGATCCGGTGCTAATTGGGG | 0.844611 CTGATCTCGGGCCTAAGACG | GGCATAAGGCGTAGGTGACC | 0.855093 GGGCCTAAGACGCTGATTCC | GGACCAAGGAGCAAGAGTGG | | | | | |
| NCU00216T0 | 3 | 5 | NCU00216T0\_input.txt | NCU00216T0\_output.txt | 0.493093 CCAGGAGTTCGAGCTCAAGG | GAGATCGGGGTGTAGGAACG | 0.570385 CCAGGAGTTCGAGCTCAAGG | ATGAGCAGGTCGAAGTAGCC | 0.714826 CCAGGAGTTCGAGCTCAAGG | ACGCACAATCTCCTTGGTGG | 0.825814 AAATTCACGCGCTAACGACC | GAGATCGGGGTGTAGGAACG | 0.844953 ATCATCTCCCACAACGTCGC | GAGATCGGGGTGTAGGAACG | | | | | |
| NCU00218T0 | 5 | 5 | NCU00218T0\_input.txt | NCU00218T0\_output.txt | 0.905810 TACACATCCAGCGACTGTCG | GCCCTCCTTGAGATTTTCGC | 0.911207 TACACATCCAGCGACTGTCG | CCTGTCATGGCACTACACCG | 1.059074 TACACATCCAGCGACTGTCG | TGCATCTTCAACCACCCACG | 1.189264 TGTGTTTGCCAACAAGACCG | GCCCTCCTTGAGATTTTCGC | 1.194661 TGTGTTTGCCAACAAGACCG | CCTGTCATGGCACTACACCG | | | | | |
| NCU00219T0 | 3 | 5 | NCU00219T0\_input.txt | NCU00219T0\_output.txt | 0.926158 TGGAGGGATATCTTCGGGGG | TATTGGTCCTGAGAGCGAGC | 0.928145 TCGAATGCCAATGCCTCTGG | GGAAGGAGGCGTATTGGTCC | 1.001007 TCGAATGCCAATGCCTCTGG | TGTGGAAGGAGGCGTATTGG | 1.070478 TCGAATGCCAATGCCTCTGG | GGACAAAGGGACGGTCTTCC | 1.071637 TCGAATGCCAATGCCTCTGG | AAAGGGACGGTCTTCCATGC | | | | | |
| NCU00220T0 | 2 | 5 | NCU00220T0\_input.txt | NCU00220T0\_output.txt | 0.289099 CACATCGACGCGAAGAAAGC | CGTGTTGTTGTTGCTCGAGG | 0.497206 CACATCGACGCGAAGAAAGC | TGTTGTTGCTCGAGGAAGGG | 0.573838 CACATCGACGCGAAGAAAGC | AGGGTTGGTGTCGTATTGGG | 0.711506 TTCACCCGAGTCACAATCGC | CGTGTTGTTGTTGCTCGAGG | 0.790661 CACATCGACGCGAAGAAAGC | GGGAGACTTGGACCATGAGG | | | | | |
| NCU00221T0 | 4 | 5 | NCU00221T0\_input.txt | NCU00221T0\_output.txt | 0.075485 CACCTCTGTCTCGTCAACCC | TGTCTGTAAAGAGGCTGCCG | 0.075485 CCACCTCTGTCTCGTCAACC | TGTCTGTAAAGAGGCTGCCG | 0.222500 ACCCCTTCTTCACCACAACC | TGTCTGTAAAGAGGCTGCCG | 0.428499 CACCTCTGTCTCGTCAACCC | AGGTAGCTGATGTGTGTGCG | 0.428499 CCACCTCTGTCTCGTCAACC | AGGTAGCTGATGTGTGTGCG | | | | | |
| NCU00222T0 | 2 | 5 | NCU00222T0\_input.txt | NCU00222T0\_output.txt | 0.501675 AGTCGCCGATCAAAGAAGGG | CAGTTGAGGATCACCCCAGC | 0.634762 AAGTGGAGGAGGAGTCGAGG | CGGTTCTTTCCTTGTGTGGC | 0.717445 AAGTGGAGGAGGAGTCGAGG | CAGTTGAGGATCACCCCAGC | 0.776844 AGTCGCCGATCAAAGAAGGG | CGAGTCTTGCTGGAGTGTGG | 0.855085 TGGAGATGATGTCAGTCGCC | CAGTTGAGGATCACCCCAGC | | | | | |
| NCU00223T1 | 4 | 5 | NCU00223T1\_input.txt | NCU00223T1\_output.txt | 0.346673 TTGGCGGAAACACGAATTCG | TGCTGGTTTCCTTGAGGACC | 0.419411 GCAATTGGGTAACGGAGTGC | CAGGTCCGCTTGGAGTATCG | 0.426214 TAAAGATCCACCCGCCTTCG | CAGGTCCGCTTGGAGTATCG | 0.428246 GCCTTCGCAATTGGGTAACG | CAGGTCCGCTTGGAGTATCG | 0.485260 TTGGCGGAAACACGAATTCG | CAGGTCCGCTTGGAGTATCG | | | | | |
| NCU00223T0 | 4 | 5 | NCU00223T0\_input.txt | NCU00223T0\_output.txt | 0.346673 TTGGCGGAAACACGAATTCG | TGCTGGTTTCCTTGAGGACC | 0.419411 GCAATTGGGTAACGGAGTGC | CAGGTCCGCTTGGAGTATCG | 0.485260 TTGGCGGAAACACGAATTCG | CAGGTCCGCTTGGAGTATCG | 0.502546 AATTCGTTCTACCCCACGCC | TGCTGGTTTCCTTGAGGACC | 0.557840 TTGGCGGAAACACGAATTCG | GTATGCTTGGCCGGGATAGG | | | | | |
| NCU00224T0 | 2 | 5 | NCU00224T0\_input.txt | NCU00224T0\_output.txt | 0.501327 CCTGTTCCTCCAACCTGTCC | CCAGACGGAACCATCCTACC | 0.578737 CCTGTTCCTCCAACCTGTCC | ATGACCAGACGGAACCATCC | 0.713155 GAGTAGTGTAGAGCCGCAGC | CCAGACGGAACCATCCTACC | 0.790565 GAGTAGTGTAGAGCCGCAGC | ATGACCAGACGGAACCATCC | 0.854529 CCTGTTCCTCCAACCTGTCC | TGAGCTACATGACCAGACGG | | | | | |
| NCU00225T0 | 5 | 5 | NCU00225T0\_input.txt | NCU00225T0\_output.txt | 0.428014 CGCCACTCTAAGTCCATCCC | TCCTTCTGCTCAGTCTTGGC | 0.428014 CCGCCACTCTAAGTCCATCC | TCCTTCTGCTCAGTCTTGGC | 0.634594 CGCCACTCTAAGTCCATCCC | GCTCAGTCTTGGCGATCTCC | 0.634594 CCGCCACTCTAAGTCCATCC | GCTCAGTCTTGGCGATCTCC | 0.793316 GCCACTCTAAGTCCATCCCC | TCCTTCTGCTCAGTCTTGGC | | | | | |
| NCU00226T0 | 3 | 5 | NCU00226T0\_input.txt | NCU00226T0\_output.txt | 0.213751 GAGGAGAATGGGAGATGGCG | AAGCGGTGATCGAGTACTGC | 0.284913 GAGATGGCGGAGGAGAAAGG | AAGCGGTGATCGAGTACTGC | 0.358169 GCAGATGCCCAAGAGGTAGG | AAGCGGTGATCGAGTACTGC | 0.497575 TGCTGTTGAGGAAGACGAGG | AAGCGGTGATCGAGTACTGC | 0.504806 TAGGTCAGGGCTTGGAGAGG | AAGCGGTGATCGAGTACTGC | | | | | |
| NCU00227T0 | 4 | 5 | NCU00227T0\_input.txt | NCU00227T0\_output.txt | 0.139935 TGTCCTGCTTCGTCCTTTCC | GTAGGACTGGGCGATCTTGG | 0.140478 TTCTTCCCTTCCGCATGTCC | GTAGGACTGGGCGATCTTGG | 0.143033 TGTCCTGCTTCGTCCTTTCC | CAAGGAAGTAGGACTGGGCG | 0.143033 TGTCCTGCTTCGTCCTTTCC | CGGCAAGGAAGTAGGACTGG | 0.143576 TTCTTCCCTTCCGCATGTCC | CAAGGAAGTAGGACTGGGCG | | | | | |
| NCU00229T0 | 2 | 5 | NCU00229T0\_input.txt | NCU00229T0\_output.txt | 0.198684 TCGAGAACCTGACTTTGGCC | GTCGTTGTCTGTTGGCATCG | 0.273184 TCGAGAACCTGACTTTGGCC | GTTGTCTGTTGGCATCGTGG | 0.768366 CTGACTTTGGCCCAACATGG | GTCGTTGTCTGTTGGCATCG | 0.842866 CTGACTTTGGCCCAACATGG | GTTGTCTGTTGGCATCGTGG | 1.038762 TCGAGAACCTGACTTTGGCC | GAACTGTCGTTGTCTGTTGGC | | | | | |
| NCU00230T0 | 6 | 5 | NCU00230T0\_input.txt | NCU00230T0\_output.txt | 1.171650 CCGTGCATCCAAGAGATTCG | GGCGTTGTCTTTCTGCTTCC | 1.178705 GTTTCACTGGGCTAATCGCG | GAACCATTCGTGCTCAAGGG | 1.182006 CGCTCGAAACTGGACTACCC | GCTCTCTGAACCATTCGTGC | 1.193499 ACTACCCTGCTCCCGATACC | GCTCTCTGAACCATTCGTGC | 1.263014 CGCTCGAAACTGGACTACCC | GAACCATTCGTGCTCAAGGG | | | | | |
| NCU00231T0 | 1 | 5 | NCU00231T0\_input.txt | NCU00231T0\_output.txt | 0.193414 CTCTCCTGCACAGCATTTGC | GATAACGTTCGCGGTCAACG | 0.278205 CTCTCCTGCACAGCATTTGC | GTAGATGAGGAAGGGCTGCC | 0.345828 TTCTTCCTCTCCTGCACAGC | GATAACGTTCGCGGTCAACG | 0.430618 TTCTTCCTCTCCTGCACAGC | GTAGATGAGGAAGGGCTGCC | 0.463225 GTTGACCGCGAACGTTATCG | CTTCGGCGTCTCTTTCTTGC | | | | | |
| NCU00232T0 | 1 | 5 | NCU00232T0\_input.txt | NCU00232T0\_output.txt | 0.215487 GGATCTTGGACTTGGGAGGC | TCAAGAATACGTCGGGTGCC | 0.279770 GGATCTTGGACTTGGGAGGC | CCTAGAACCACTCCGTTCCG | 0.281362 GAGCTAGTGAACACCGAGGG | AGGATAAGAACCGTGCTGCC | 0.492323 GAACACCGAGGGATCTACCG | AGGATAAGAACCGTGCTGCC | 0.557113 GTCTTCGAGACCGAGTCTGG | TCAAGAATACGTCGGGTGCC | | | | | |
| NCU00233T0 | 3 | 5 | NCU00233T0\_input.txt | NCU00233T0\_output.txt | 0.408066 TGAGGTTTTTCGCGTTGTCG | GGCTCAGTCACCATAGCTCC | 0.429407 TCGGTTGCTAACTGGAAGGG | GGCTCAGTCACCATAGCTCC | 0.489638 GAGGTTTTTCGCGTTGTCGG | GGCTCAGTCACCATAGCTCC | 0.616999 CGTTGTCGGTTGCTAACTGG | GGCTCAGTCACCATAGCTCC | 0.639086 TGTCGGGGTGAGGTTTTTCG | GGCTCAGTCACCATAGCTCC | | | | | |
| NCU00234T0 | 2 | 5 | NCU00234T0\_input.txt | NCU00234T0\_output.txt | 0.127301 GTTGGTGTGGCACTTCAACC | GTCAATTGCGATGACGGTCG | 0.355143 TTGACAGGCCGAGTCTTTCC | GAGCCAGCAGGGATATGTCG | 0.357595 TCCCTCATTTCTGTCTGCGG | GGTTCCATTACCTGGGGTCG | 0.357983 TTGACAGGCCGAGTCTTTCC | AAGGGCGTGGTTCCATTACC | 0.422547 GTTGGTGTGGCACTTCAACC | GAGCCAGCAGGGATATGTCG | | | | | |
| NCU00236T0 | 1 | 5 | NCU00236T0\_input.txt | NCU00236T0\_output.txt | 0.779347 GAACTCCGTCCCATCTTCCC | TGACTGAAGGCTCTTGCACG | 0.926979 TGTGCTTCCATCTGACCTGG | TGACTGAAGGCTCTTGCACG | 0.962306 ATTCATACGACTTGCGACGC | CACAAGGAACTGACAAGCGC | 0.994583 GTGCTTCCATCTGACCTGGG | TGACTGAAGGCTCTTGCACG | 1.018354 AGACCCCTGAGAAGAAACGG | CACAAGGAACTGACAAGCGC | | | | | |
| NCU00237T0 | 2 | 5 | NCU00237T0\_input.txt | NCU00237T0\_output.txt | 0.143021 TCTTTGTGGTCATGGGAGGC | TGAGATGTACGCCTGCATCC | 0.145625 TCTTTGTGGTCATGGGAGGC | GGCAATGACTGACCCTCTCC | 0.217238 TCTTTGTGGTCATGGGAGGC | ATGGCACTGAGATGTACGCC | 0.283968 CCAACAACACCAACACCAGC | TGAGATGTACGCCTGCATCC | 0.286573 CCAACAACACCAACACCAGC | GGCAATGACTGACCCTCTCC | | | | | |
| NCU00238T0 | 2 | 5 | NCU00238T0\_input.txt | NCU00238T0\_output.txt | 0.415206 TGGCACACAATGTTGAACGG | AGGAGGTGGAGGAGTGATCC | 0.637227 GGACGACGGAGGAGAAAAGG | AGAAGACGGAGAAGAAGGCG | 0.637227 GAGGAAAAGGACGACGGAGG | AGAAGACGGAGAAGAAGGCG | 0.637227 GAGGAGGAAAAGGACGACGG | AGAAGACGGAGAAGAAGGCG | 0.637227 GGAGGAGGAAAAGGACGACG | AGAAGACGGAGAAGAAGGCG | | | | | |
| NCU00239T1 | 4 | 5 | NCU00239T1\_input.txt | NCU00239T1\_output.txt | 0.142857 TTCGTTATGTGAGGACCGGC | TAAGACCACCACAAGTCCGC | 0.285210 TCATAGGGTCATTGTGCCGG | GACGATGGAGGCAAGGTAGG | 0.291437 TCATAGGGTCATTGTGCCGG | CGTTGCTTTTCACCGAGACG | 0.426798 TCGTACTGGGAAGCATTGGC | TAAGACCACCACAAGTCCGC | 0.497500 TCGTACTGGGAAGCATTGGC | GACGATGGAGGCAAGGTAGG | | | | | |
| NCU00239T0 | 4 | 5 | NCU00239T0\_input.txt | NCU00239T0\_output.txt | 0.142857 TTCGTTATGTGAGGACCGGC | TAAGACCACCACAAGTCCGC | 0.285210 TCATAGGGTCATTGTGCCGG | GACGATGGAGGCAAGGTAGG | 0.291437 TCATAGGGTCATTGTGCCGG | CGTTGCTTTTCACCGAGACG | 0.426798 TCGTACTGGGAAGCATTGGC | TAAGACCACCACAAGTCCGC | 0.497500 TCGTACTGGGAAGCATTGGC | GACGATGGAGGCAAGGTAGG | | | | | |
| NCU00240T0 | 8 | 5 | NCU00240T0\_input.txt | NCU00240T0\_output.txt | 0.145750 CCACTGACATGCACGTTTGG | CGGTGATGAGGGTGATGAGG | 0.147781 CAACGACACCACAGCTTTCG | CGGTGATGAGGGTGATGAGG | 0.219230 CCACTGACATGCACGTTTGG | GGTGATGAGGGATGTGTCGG | 0.219230 CCACTGACATGCACGTTTGG | GGGTGATGAGGGATGTGTCG | 0.221260 CAACGACACCACAGCTTTCG | GGTGATGAGGGATGTGTCGG | | | | | |
| NCU00241T0 | 2 | 5 | NCU00241T0\_input.txt | NCU00241T0\_output.txt | 0.278542 TCTGGATCGGAGGAGGAAGG | TGGACGCTGTCGACTAATGG | 0.279047 TCTGTCTGGATCGGAGGAGG | TGGACGCTGTCGACTAATGG | 0.279047 TCCTCTGTCTGGATCGGAGG | TGGACGCTGTCGACTAATGG | 0.351567 AACAGGAACAGGTTCAGGGC | TGGACGCTGTCGACTAATGG | 0.571811 ATCGGAGGAGGAAGGAAGGG | TGGACGCTGTCGACTAATGG | | | | | |
| NCU00242T0 | 2 | 5 | NCU00242T0\_input.txt | NCU00242T0\_output.txt | 0.417416 AATCATGGAAGGACGACCCG | CTGCTTTTCTTCTGTGCCGG | 0.428192 AATCATGGAAGGACGACCCG | TCCAGTTTACAGCGTGGTGG | 0.492141 ATCTTCGGCTGCTTGTTTGC | TCCAGTTTACAGCGTGGTGG | 0.556657 GCCGTTGCCGTTGTATATGG | TCCAGTTTACAGCGTGGTGG | 0.564547 AATCATGGAAGGACGACCCG | GCTGCTTTTCTTCTGTGCCG | | | | | |
| NCU00243T0 | 4 | 5 | NCU00243T0\_input.txt | NCU00243T0\_output.txt | 0.498260 GAACCGGCTACTCACTCTGG | TGAACATTGGATGGCAGGGG | 0.623852 TTCACGTTGAACTTGACGCG | TGAACATTGGATGGCAGGGG | 0.636467 GAACCGGCTACTCACTCTGG | GAATCCTTCCTCGCTGGTGG | 0.762059 TTCACGTTGAACTTGACGCG | GAATCCTTCCTCGCTGGTGG | 0.850007 GGGAACATCGCCGAAAATGC | TGAACATTGGATGGCAGGGG | | | | | |
| NCU00244T1 | 4 | 5 | NCU00244T1\_input.txt | NCU00244T1\_output.txt | 0.506657 TATCGAATATGCACCCGGGC | AGGTGAAGGGGCAGTTTTGG | 0.772828 GTTCACATGCCTGCACTTGC | GAGGAAGGAGTGATGGGTGC | 0.773295 CCAGAGAATCGTCCTCCTCG | GAGGAAGGAGTGATGGGTGC | 0.837946 GTTCACATGCCTGCACTTGC | TTGATGGCGTGAGGACTACG | 0.869261 CCTACATCTTCCAGAGGGCC | GAGGAAGGAGTGATGGGTGC | | | | | |
| NCU00244T0 | 3 | 5 | NCU00244T0\_input.txt | NCU00244T0\_output.txt | 0.506657 TATCGAATATGCACCCGGGC | AGGTGAAGGGGCAGTTTTGG | 0.772828 GTTCACATGCCTGCACTTGC | GAGGAAGGAGTGATGGGTGC | 0.773295 CCAGAGAATCGTCCTCCTCG | GAGGAAGGAGTGATGGGTGC | 0.837946 GTTCACATGCCTGCACTTGC | TTGATGGCGTGAGGACTACG | 0.869261 CCTACATCTTCCAGAGGGCC | GAGGAAGGAGTGATGGGTGC | | | | | |
| NCU00245T0 | 2 | 5 | NCU00245T0\_input.txt | NCU00245T0\_output.txt | 0.139176 GATGGTGACTGGTGGGAAGG | TGAGCCCGTACATTCATCGG | 0.211735 GATGGTGACTGGTGGGAAGG | AGTAGCAGGCACGAATGAGG | 0.211735 GATGGTGACTGGTGGGAAGG | AAGCGATGGTCAGTAGCAGG | 0.490013 GATGGTGACTGGTGGGAAGG | CTGTGGGCGCTTCATTTTCG | 0.564910 GACCTGAGGCAGAAGATGGG | TTTGAAACGGCGTCGATTGC | | | | | |
| NCU00246T1 | 7 | 5 | NCU00246T1\_input.txt | NCU00246T1\_output.txt | 0.215357 TATTGGCTGCAGTCTGAGCC | ATAGCGGTCTCAAAACGGGG | 0.429179 TATTGGCTGCAGTCTGAGCC | TAATAGCCATCTCTGCGGCC | 0.506166 AGTGACAGAAGTGGAAGGCC | ATAGCGGTCTCAAAACGGGG | 0.562469 GATGATCTAGTACCGCCGCG | ATAGCGGTCTCAAAACGGGG | 0.574078 AGTGACAGAAGTGGAAGGCC | TCGAAGGTGCCAGTAGATGC | | | | | |
| NCU00246T0 | 8 | 5 | NCU00246T0\_input.txt | NCU00246T0\_output.txt | 0.215357 TATTGGCTGCAGTCTGAGCC | ATAGCGGTCTCAAAACGGGG | 0.429179 TATTGGCTGCAGTCTGAGCC | TAATAGCCATCTCTGCGGCC | 0.506166 AGTGACAGAAGTGGAAGGCC | ATAGCGGTCTCAAAACGGGG | 0.562469 GATGATCTAGTACCGCCGCG | ATAGCGGTCTCAAAACGGGG | 0.574078 AGTGACAGAAGTGGAAGGCC | TCGAAGGTGCCAGTAGATGC | | | | | |
| NCU00247T0 | 1 | 5 | NCU00247T0\_input.txt | NCU00247T0\_output.txt | 0.140497 GTTGGTATCGGGAGCTCAGG | ACAAGAGCCTCCTTCGTTGG | 0.214172 GTTGGTATCGGGAGCTCAGG | CCTTGCTCATGTTTTCGCCC | 0.214172 GTTGGTATCGGGAGCTCAGG | GGGCCTTGCTCATGTTTTCG | 0.288167 CAGCCTGGGACTTATCAGCC | CCTTGCTCATGTTTTCGCCC | 0.288167 CAGCCTGGGACTTATCAGCC | GGGCCTTGCTCATGTTTTCG | | | | | |
| NCU00248T0 | 1 | 5 | NCU00248T0\_input.txt | NCU00248T0\_output.txt | 0.074814 CGCCGCCTACTTATCAGAGG | TAAACTCCCGTTGGCACTCC | 0.145793 CGCCGCCTACTTATCAGAGG | TCCCCCGATTCAATCAAGCC | 0.214820 GGTAGGAGGAACGACATGGC | TAAACTCCCGTTGGCACTCC | 0.218155 GGTAGGAGGAACGACATGGC | CTCTGATAAGTAGGCGGCGG | 0.218155 GGTAGGAGGAACGACATGGC | CCTCTGATAAGTAGGCGGCG | | | | | |
| NCU00249T0 | 1 | 5 | NCU00249T0\_input.txt | NCU00249T0\_output.txt | 0.071712 AGTCTCTAATGGATGCGCCG | TTCCCCTTGGGTTCATTCGG | 0.144074 GGTGCCGGAGTCTCTAATGG | TTCCCCTTGGGTTCATTCGG | 0.208977 GGAAGAGAGGATGACGCTGG | CCATTAGAGACTCCGGCACC | 0.218132 GAGAGGATGACGCTGGTTCC | TTCCCCTTGGGTTCATTCGG | 0.284136 GAGAGGATGACGCTGGTTCC | CCATTAGAGACTCCGGCACC | | | | | |
| NCU00250T0 | 2 | 5 | NCU00250T0\_input.txt | NCU00250T0\_output.txt | 0.271273 TTCCACACCATCTTCTCCGC | CTTTTGTCCCGAAGTCGTGC | 0.283023 TTCCACACCATCTTCTCCGC | TTCTTCCTGCCTACACTCGC | 0.469769 GTCGGCGACTTGTTCAAAGG | CTTTTGTCCCGAAGTCGTGC | 0.481519 GTCGGCGACTTGTTCAAAGG | TTCTTCCTGCCTACACTCGC | 0.556555 GTCGGCGACTTGTTCAAAGG | AGTCATCGGCCCATATTCGG | | | | | |
| NCU00251T0 | 3 | 5 | NCU00251T0\_input.txt | NCU00251T0\_output.txt | 0.074479 AGCTGTTTAGGCAGTGACCC | GGGTGACAGAAGTGGTACGG | 0.285947 AGCTGTTTAGGCAGTGACCC | CTGGATAGACGGCGTTCAGG | 0.558610 AGCTGTTTAGGCAGTGACCC | GGCGTTCAGGCTTACTTTGG | 0.645639 TTCGGCCCAAGCTGTTTAGG | GGGTGACAGAAGTGGTACGG | 0.712397 AGCTGTTTAGGCAGTGACCC | GTGGTACGGAATGGTGGTGG | | | | | |
| NCU00252T0 | 4 | 5 | NCU00252T0\_input.txt | NCU00252T0\_output.txt | 0.561684 ATGTAGACGCGGAAGAGAGC | GACGAGGTAGGAACACGAGC | 0.561720 ATGTAGACGCGGAAGAGAGC | GACGACGAGGTAGGAACACG | 0.563477 TGCTAAGGATGTAGACGCGG | GACGAGGTAGGAACACGAGC | 0.563513 TGCTAAGGATGTAGACGCGG | GACGACGAGGTAGGAACACG | 0.925498 TGCGTGCATAGAGATGACGG | TGGATGAACGTGTGGCATCC | | | | | |
| NCU00253T0 | 3 | 5 | NCU00253T0\_input.txt | NCU00253T0\_output.txt | 1.237501 GACGTTGAGTAAGACGGTGC | GACAGATGGGAAGACGAGCC | 1.520628 ACAGCGAGTGATATACAGGGC | GACAGATGGGAAGACGAGCC | 1.590977 GACGTTGAGTAAGACGGTGC | TGACAGATGGGAAGACGAGC | 1.734528 ACAGCGAGTGATATACAGGGC | AATGATCACAGGTCGTGGCC | 1.780259 TGACGTTGAGTAAGACGGTGC | GACAGATGGGAAGACGAGCC | | | | | |
| NCU00254T0 | 3 | 5 | NCU00254T0\_input.txt | NCU00254T0\_output.txt | 0.665513 CTTGCTTCGAACATCGCTCG | TGTTGTTGTTGTCGTCGTCG | 0.680076 GCTTCAAGTCCAGGCTTTGC | TGTTGTTGTTGTCGTCGTCG | 0.750998 TTTGTTGTCTCCTTCCCCCG | TGTTGTTGTTGTCGTCGTCG | 1.083644 CTTGCTTCGAACATCGCTCG | TTGTTGTTGTTGTCGTCGTCG | 1.098207 GCTTCAAGTCCAGGCTTTGC | TTGTTGTTGTTGTCGTCGTCG | | | | | |
| NCU00257T0 | 2 | 5 | NCU00257T0\_input.txt | NCU00257T0\_output.txt | 0.348009 AGCAACATTAGGAGCGAGGG | GGCTTGTCGAACTTGATGCC | 0.358215 AGCAACATTAGGAGCGAGGG | CGGCTTGTCGAACTTGATGC | 0.425635 AGCAACATTAGGAGCGAGGG | TTCGGTAGAGCTCCAGTTGC | 0.489430 TGTTTGAGGTGCCGAGTAGC | GGCTTGTCGAACTTGATGCC | 0.499636 TGTTTGAGGTGCCGAGTAGC | CGGCTTGTCGAACTTGATGC | | | | | |
| NCU00258T0 | 3 | 5 | NCU00258T0\_input.txt | NCU00258T0\_output.txt | 0.777077 CAATAGCCTTCTTGCCGTGG | CCGCTATCAAACGAATGCGG | 0.777077 CAATAGCCTTCTTGCCGTGG | CCCGCTATCAAACGAATGCG | 0.991292 AAACTTACCGCTGCTGGACG | CCGCTATCAAACGAATGCGG | 0.991292 AAACTTACCGCTGCTGGACG | CCCGCTATCAAACGAATGCG | 1.053589 CAATAGCCTTCTTGCCGTGG | TCGTGGTCACTTTGCTCTCG | | | | | |
| NCU00259T0 | 2 | 5 | NCU00259T0\_input.txt | NCU00259T0\_output.txt | 0.565145 ACATCTCTACACGGTTCGCG | ACTCGTCGCATTTTGCTTGC | 0.632159 ACATCTCTACACGGTTCGCG | TTTGCTTGCTGATCGATGGC | 0.784755 CCATTTGTCTACCTTGGCGC | AGGAAGCTAAGGCCAAAGGG | 0.837757 CCATTTGTCTACCTTGGCGC | ACTCGTCGCATTTTGCTTGC | 0.839348 CCATTTGTCTACCTTGGCGC | CGGAGAGAGGAAGCTAAGGC | | | | | |
| NCU00260T0 | 3 | 5 | NCU00260T0\_input.txt | NCU00260T0\_output.txt | 0.197898 ACTAGACACTTATGGCGCCG | GACGATGCCGACTTTCTTGC | 0.206885 GTTACTCCTCTCACGTCCGC | CGAGATCACGTAGACGGTGG | 0.274586 GTTACTCCTCTCACGTCCGC | GACGATGCCGACTTTCTTGC | 0.417721 GTTACTCCTCTCACGTCCGC | GCTTTGGCGAACAAGAGACC | 0.423252 ACTAGACACTTATGGCGCCG | CGAACAAGAGACCCAGGTGG | | | | | |
| NCU00261T0 | 6 | 5 | NCU00261T0\_input.txt | NCU00261T0\_output.txt | 0.560044 CAAGGAGAAGAGGCACCTGG | GGGGTTACGTGGACAATTGC | 0.567315 TCAAGGGGCTTCTCACAACG | GTTGTCTGAACTGCCGTTGC | 0.770774 TAGATGGTGGGCATATCGCG | GGGGTTACGTGGACAATTGC | 0.774709 TCAAGGGGCTTCTCACAACG | GGGGTTACGTGGACAATTGC | 0.776048 CCTCAAGGAGAAGAGGCACC | GGGGTTACGTGGACAATTGC | | | | | |
| NCU00262T1 | 3 | 5 | NCU00262T1\_input.txt | NCU00262T1\_output.txt | 0.696867 TTGTGGTTGTGGTTTTGGCG | GCATGCATTAGCCCACTACG | 0.767199 TGGATGGGTGGTGTTTTCCC | GCATGCATTAGCCCACTACG | 1.040035 GGACGGTCATGTTGAATGGC | GCATGCATTAGCCCACTACG | 1.047142 CATGTTGAATGGCCGCTTGG | GCATGCATTAGCCCACTACG | 1.338042 TTGTGGTTGTGGTTTTGGCG | CATGCATTAGCCCACTACGG | | | | | |
| NCU00262T0 | 2 | 5 | NCU00262T0\_input.txt | NCU00262T0\_output.txt | 0.985063 CAGGGGGAGGCTTATAACCG | GCATGCATTAGCCCACTACG | 1.355312 AGGGGGAGGCTTATAACCGG | GCATGCATTAGCCCACTACG | 1.626239 CAGGGGGAGGCTTATAACCG | CATGCATTAGCCCACTACGG | 1.650356 CAGGGGGAGGCTTATAACCG | GCGATTGTTTTGATGATGCGC | 1.785682 ATGATCGATCAGGGGGAGGC | GCATGCATTAGCCCACTACG | | | | | |
| NCU00263T0 | 2 | 5 | NCU00263T0\_input.txt | NCU00263T0\_output.txt | 1.845988 GCCCACTTTAGACGAGGAGG | AATGATGAGCGGGCTTTGGC | 1.855129 GCCCACTTTAGACGAGGAGG | TAATGATGAGCGGGCTTTGG | 1.991995 GGACCCGAATATCAGAGCCC | AATGATGAGCGGGCTTTGGC | 1.991995 GGGACCCGAATATCAGAGCC | AATGATGAGCGGGCTTTGGC | 2.001135 GGACCCGAATATCAGAGCCC | TAATGATGAGCGGGCTTTGG | | | | | |
| NCU00264T0 | 2 | 5 | NCU00264T0\_input.txt | NCU00264T0\_output.txt | 0.073263 TTCAGATTGACACTCCCGCC | AACAGGGAGCAGTAGTTGGC | 0.073263 TTCAGATTGACACTCCCGCC | AAGTCTTTGGGAAGCCTCGG | 0.489376 TTCAGATTGACACTCCCGCC | CTGCAAATCATGGTGACGGG | 0.490824 CGAGACGAACACTACCGTGG | AACAGGGAGCAGTAGTTGGC | 0.576985 TTCAGATTGACACTCCCGCC | AGGCAAGTCTTTGGGAAGCC | | | | | |
| NCU00265T0 | 1 | 5 | NCU00265T0\_input.txt | NCU00265T0\_output.txt | 0.559415 CAACGACTGAGAGTCTCCGC | TGATACAATCAGGCCCGTCG | 0.571636 CAACGACTGAGAGTCTCCGC | ACCACCAAGGAATTTCCCCC | 0.573784 GAATGCACTAAGGGCTTGGC | ACCACCAAGGAATTTCCCCC | 0.714353 AATGAGCCTGGTGTCAAGGC | TGATACAATCAGGCCCGTCG | 0.726574 AATGAGCCTGGTGTCAAGGC | ACCACCAAGGAATTTCCCCC | | | | | |
| NCU00267T0 | 2 | 5 | NCU00267T0\_input.txt | NCU00267T0\_output.txt | 0.565216 TGAGATGGTGGTTGCGATGG | GAAGACTGTGGAGGAGCTCG | 0.690988 CTGTTGAGATGGTGGTTGCG | GAAGACTGTGGAGGAGCTCG | 0.701730 CTTCATCTTTGGGGTGCTGC | GAAGACTGTGGAGGAGCTCG | 1.008714 TGAGATGGTGGTTGCGATGG | GGGGAAGAAGACTGTGGAGG | 1.134486 CTGTTGAGATGGTGGTTGCG | GGGGAAGAAGACTGTGGAGG | | | | | |
| NCU00268T0 | 3 | 5 | NCU00268T0\_input.txt | NCU00268T0\_output.txt | 0.292978 TTTCCCCTAAGAGGAAGCGC | GCTAGTCGAGTCGATGAGGC | 0.500209 TTTCCCCTAAGAGGAAGCGC | TACCCACGCACACATACAGG | 0.717294 TTTCCCCTAAGAGGAAGCGC | ATGGAGCGCCTCAAAGATGG | 0.833929 CAAGTTAGAGGCTGCAGTGC | CGGTCTGTAGGCTATGCTGG | 0.841420 TTTCCCCTAAGAGGAAGCGC | GGCTAGTCGAGTCGATGAGG | | | | | |
| NCU00269T0 | 3 | 5 | NCU00269T0\_input.txt | NCU00269T0\_output.txt | 0.559840 CATTCTGCGAACCAAACCCC | ACGGTGGTTCTTGTAGTCGG | 0.559840 CCATTCTGCGAACCAAACCC | ACGGTGGTTCTTGTAGTCGG | 0.704724 GCCATTCTGCGAACCAAACC | ACGGTGGTTCTTGTAGTCGG | 0.765371 CATTCTGCGAACCAAACCCC | GTTGATCTCACGGCCAAACC | 0.765371 CCATTCTGCGAACCAAACCC | GTTGATCTCACGGCCAAACC | | | | | |
| NCU00270T0 | 2 | 5 | NCU00270T0\_input.txt | NCU00270T0\_output.txt | 0.701315 ACCATGGTGGATGCTAAGCC | CAACCTGCCAGTCAAAACGC | 0.830500 ACCATGGTGGATGCTAAGCC | GCTCTTGATTCACGGTCTGC | 0.844908 ACCATGGTGGATGCTAAGCC | ACAACCTGCCAGTCAAAACG | 1.060256 TTGTTGTGTCCCTCTGTGCC | CAACCTGCCAGTCAAAACGC | 1.060256 TGGCTGTCTTGTTGTGTCCC | CAACCTGCCAGTCAAAACGC | | | | | |
| NCU00271T0 | 3 | 5 | NCU00271T0\_input.txt | NCU00271T0\_output.txt | 0.631210 GACGAGGATGGTCAACAGGG | TCTTTCGTACGTTCCTCCCG | 0.981615 CGACGAGGATGGTCAACAGG | TCTTTCGTACGTTCCTCCCG | 1.057606 TTGGTTACACCGATGCAAGC | CCGTCTTTGGCTTTCATCGC | 1.146570 ATGCAAGCAAGTGGGTAGGC | CCGTCTTTGGCTTTCATCGC | 1.176471 GACGAGGATGGTCAACAGGG | TTCTTTCGTACGTTCCTCCCG | | | | | |
| NCU00272T0 | 2 | 5 | NCU00272T0\_input.txt | NCU00272T0\_output.txt | 0.780804 AACAAACGTCTCCTCTCGCC | TGGAACGGGATGCATACACG | 0.786196 AACAAACGTCTCCTCTCGCC | TCTGGGTGGGTTTTGGAACG | 0.983644 AGGCATCGATGGACAAGTCG | ACGGGATGCATACACGAACG | 1.038523 GTTCAAGTCAAGACAGCCGG | GAGAGCGTCACTGAGTGAGG | 1.124184 AACAAACGTCTCCTCTCGCC | ACGGGATGCATACACGAACG | | | | | |
| NCU00273T0 | 3 | 5 | NCU00273T0\_input.txt | NCU00273T0\_output.txt | 1.899863 GTCTCCACCACCCACATTGG | AGAAGAAGGAGGAGGAGGAGG | 2.333307 GTATCGCCCAGACATCCTCG | TCTGTCTGACAAGCATTTTGGC | 2.397360 GTCTCCACCACCCACATTGG | GAAGAAGGAGGAGGAGGAGG | 2.403459 GCTATACTCGGCGATCCACC | TCTGTCTGACAAGCATTTTGGC | 2.475701 CCACCACCCACATTGGAACC | AGAAGAAGGAGGAGGAGGAGG | | | | | |
| NCU00274T0 | 4 | 5 | NCU00274T0\_input.txt | NCU00274T0\_output.txt | 0.926465 TGACGAGAGGAATTTGGCGG | TTCTAGCCCTGGCAAGTACG | 0.986014 GTAGAGGCAGAGGATGAGCG | CCATCAAGCATAAGCAGCCC | 1.068067 CAGAATGTAAGGGTGGGGGC | CCATCAAGCATAAGCAGCCC | 1.351292 GGTAGAGGCAGAGGATGAGC | CCATCAAGCATAAGCAGCCC | 1.497767 TAGAGGCAGAGGATGAGCGG | CCATCAAGCATAAGCAGCCC | | | | | |
| NCU00275T0 | 3 | 5 | NCU00275T0\_input.txt | NCU00275T0\_output.txt | 1.501872 GAGGAACAGGAACACCAGGG | GTTTGGCCTGTCTCTTTAGCG | 1.644026 AACAGGAACACCAGGGTTCG | GTTTGGCCTGTCTCTTTAGCG | 1.714395 AGTTGGAGTTCGGATTCGGG | GTTTGGCCTGTCTCTTTAGCG | 1.845285 GAGGAACAGGAACACCAGGG | TTTGGCCTGTCTCTTTAGCG | 1.987440 AACAGGAACACCAGGGTTCG | TTTGGCCTGTCTCTTTAGCG | | | | | |
| NCU00276T0 | 1 | 5 | NCU00276T0\_input.txt | NCU00276T0\_output.txt | 0.774194 AGACAACGACGACTCCAAGC | CTCTCTCTCGCTCACCAAGG | 1.060493 CCAAAGAGCAAAAGGAGCCC | CTCTCTCTCGCTCACCAAGG | 1.168649 ACTTGTATCTTGGGGGCAGC | CTACACAACTCCTCCCTACGC | 1.214118 ACTTGTATCTTGGGGGCAGC | TACACAACTCCTCCCTACGC | 1.277484 CCAAAGAGCAAAAGGAGCCC | TCTTTTCATCACCCGTCGCC | | | | | |
| NCU00277T0 | 2 | 5 | NCU00277T0\_input.txt | NCU00277T0\_output.txt | 0.664258 GCGTTGTTCGATAGTCACGG | CGTCGTTCATTAGCAGCTGC | 0.969824 TCTTCTGCTGGTTCGTTCGC | CGTCGTTCATTAGCAGCTGC | 1.062220 GGGATCCTCTCTCTTGACGG | CGTCGTTCATTAGCAGCTGC | 1.082798 GTGCGTTGTTCGATAGTCACG | CGTCGTTCATTAGCAGCTGC | 1.218047 GTCGTTTCTTCTGCTGGTTCG | CGTCGTTCATTAGCAGCTGC | | | | | |
| NCU00278T0 | 2 | 5 | NCU00278T0\_input.txt | NCU00278T0\_output.txt | 0.210850 GCCATCAAGTTTCCATGGCG | GGGTACTGAACTATCCGGCG | 0.279181 TCATGTCGCTCAACCTCAGG | GGGTACTGAACTATCCGGCG | 0.281103 TCATGTCGCTCAACCTCAGG | GGGCGGAACTGATAGTAGGC | 0.356278 TCTCACTCTCGTCTCACCCC | GGGCGGAACTGATAGTAGGC | 0.483834 GCCATCAAGTTTCCATGGCG | GCGTGTATCGAAGCCATTGG | | | | | |
| NCU00279T0 | 1 | 5 | NCU00279T0\_input.txt | NCU00279T0\_output.txt | 0.211045 AAGATCAAGAAGGCGGGTGG | GGACTCGCCACTTCTAAGGG | 0.211045 AAGATCAAGAAGGCGGGTGG | GGGACTCGCCACTTCTAAGG | 0.212204 GAAGGGGATTGAGGAACGGG | CCGCACTCCTCTCCAATACC | 0.214193 AAGATCAAGAAGGCGGGTGG | TCATAGCCTCAACTGCGTCG | 0.281029 GGTATTGGAGAGGAGTGCGG | GGACTCGCCACTTCTAAGGG | | | | | |
| NCU00280T0 | 1 | 5 | NCU00280T0\_input.txt | NCU00280T0\_output.txt | 0.285742 CAGCACTCTGGTCTGGATCC | CAAGGGACAAGAGTAGCGGG | 0.422902 CTACCTCGGCATCTTCCTCG | TTGACCAGTCTTCCCTTGGC | 0.429100 AAAGCCAATCAACAACGCCG | TTGACCAGTCTTCCCTTGGC | 0.431608 AGCAGAAGAAGCCTTGGTCG | TTGACCAGTCTTCCCTTGGC | 0.434186 ACTCTGGTCTGGATCCAGGG | CAAGGGACAAGAGTAGCGGG | | | | | |
| NCU00281T0 | 1 | 5 | NCU00281T0\_input.txt | NCU00281T0\_output.txt | 0.147116 CGCTTTACGTCAAGTGCACC | GGTCTCCCACTACAGTTGGC | 0.205682 CTTGTTGCGATGCTTGGAGG | AATCCTCCCACAGCAAGACG | 0.357656 TCAAAAGGAGTGAGTGGCCG | GTAACATTGGACCTCCCCGG | 0.429351 TCAAAAGGAGTGAGTGGCCG | TAGATTGCGGCTACCCTTGC | 0.432964 TTGGGCTGGTTAGGTAACGG | GTAACATTGGACCTCCCCGG | | | | | |
| NCU00282T0 | 3 | 5 | NCU00282T0\_input.txt | NCU00282T0\_output.txt | 0.704028 CTTGGAGAGAAGTCGACCGG | TCACTCAGGGAATGACTCGC | 0.850053 AGGGTCGACGTTCTCTTTGG | TCACTCAGGGAATGACTCGC | 1.057436 TAGGAAGTCGAGTTCGTGGC | TCACTCAGGGAATGACTCGC | 1.134132 TCGACGTTCTCTTTGGGAGG | TCACTCAGGGAATGACTCGC | 1.202198 GGGTCGACGTTCTCTTTGGG | TCACTCAGGGAATGACTCGC | | | | | |
| NCU00282T1 | 2 | 5 | NCU00282T1\_input.txt | NCU00282T1\_output.txt | 0.367448 AGGTGTCATCCAGAGAGGGG | TGTGAATTGTGAAGTGCGCG | 0.662971 TCCAGAGAGGGGACATTGGG | TGTGAATTGTGAAGTGCGCG | 0.785088 CGAGGTGTCATCCAGAGAGG | TGTGAATTGTGAAGTGCGCG | 1.051833 AGGTGTCATCCAGAGAGGGG | GTGAATTGTGAAGTGCGCGG | 1.160238 GAGGTGTCATCCAGAGAGGG | TGTGAATTGTGAAGTGCGCG | | | | | |
| NCU00283T0 | 3 | 5 | NCU00283T0\_input.txt | NCU00283T0\_output.txt | 0.569346 ATCGTCGTTGTATAGCGGGC | CAAGTGTCTTGGGAGGGTGG | 0.640097 ATCGTCGTTGTATAGCGGGC | ATTCACCCTCTCGTGGTTGC | 0.918693 GCAGACGGGGATATTCTCGC | CAAGTGTCTTGGGAGGGTGG | 0.924886 TGCAGACGGGGATATTCTCG | CAAGTGTCTTGGGAGGGTGG | 1.087067 ATCGTCGTTGTATAGCGGGC | TAAGTGAGGTGTTGGGTGGG | | | | | |
| NCU00284T0 | 1 | 5 | NCU00284T0\_input.txt | NCU00284T0\_output.txt | 0.566951 TCATCCATTTAGACGGCCGG | CATGAACCAGTCTCGAGGGC | 0.571065 ACCATCAACAGACAGACGGC | TTGTTCCAGCACTCCTTCCG | 0.621596 CATCAGACGATTGTGTGCCG | TTGTTCCAGCACTCCTTCCG | 0.632175 TCATCCATTTAGACGGCCGG | TCCGTCATGAACCAGTCTCG | 0.694661 CATCAGACGATTGTGTGCCG | TGCTCACCCAATAGCCAACC | | | | | |
| NCU00285T0 | 5 | 5 | NCU00285T0\_input.txt | NCU00285T0\_output.txt | 0.509015 GGAATACCTTCAGCCCGAGG | AGAACCAAAGTCCAGGAGGC | 0.581166 CCAGGAATACCTTCAGCCCG | AGAACCAAAGTCCAGGAGGC | 0.712974 CTTTCCAACGGTGTTCAGCC | AGAACCAAAGTCCAGGAGGC | 0.712974 CCTTTCCAACGGTGTTCAGC | AGAACCAAAGTCCAGGAGGC | 0.929685 CATCAAGCCCTTTCCAACGG | AGAACCAAAGTCCAGGAGGC | | | | | |
| NCU00285T1 | 2 | 5 | NCU00285T1\_input.txt | NCU00285T1\_output.txt | 0.280176 GCAAGCCGATGGTCTTTTCC | GCATCCTTCAACCTTGCAGC | 0.285855 CTCTTCCTCCTCCTTTCGGC | GCATCCTTCAACCTTGCAGC | 0.340903 GCAAGCCGATGGTCTTTTCC | CATCCTTCAACCTTGCAGCG | 0.346583 CTCTTCCTCCTCCTTTCGGC | CATCCTTCAACCTTGCAGCG | 0.360687 TCTTTTCCACTGCCCATGGG | GCATCCTTCAACCTTGCAGC | | | | | |
| NCU00287T0 | 1 | 5 | NCU00287T0\_input.txt | NCU00287T0\_output.txt | 0.211782 TCTGCTGTTCCATCCGTAGC | AGCCAAGTTCTCACTCTGGC | 0.354587 TGCCAGAGTGAGAACTTGGC | AAAAGGTGTGGAAAAGCGGC | 0.355145 GGTGTTGGACTCGAGGATCC | CATCACGGCCTTGAACATGC | 0.358838 TCCTCTGGCATGTTCAAGGC | AGCCAAGTTCTCACTCTGGC | 0.360269 TTGCCGATGGAGGATGAAGG | CATCACGGCCTTGAACATGC | | | | | |
| NCU00288T0 | 3 | 5 | NCU00288T0\_input.txt | NCU00288T0\_output.txt | 0.215867 CTCCTCAACCACCCAAGACC | TGAGGTTGCAGTTCAAGGGG | 0.360599 CTCCTCAACCACCCAAGACC | CAGTTCAAGGGGTCCCATGG | 0.499763 CTCAACCACCCAAGACCTCG | TGAGGTTGCAGTTCAAGGGG | 0.644495 CTCAACCACCCAAGACCTCG | CAGTTCAAGGGGTCCCATGG | 0.982140 CTCCTCAACCACCCAAGACC | CTGCCCACGTTGCAAAATGG | | | | | |
| NCU00289T0 | 4 | 5 | NCU00289T0\_input.txt | NCU00289T0\_output.txt | 0.133727 ATTTGCGTATCGTGTTGCCG | AGTCAAGCTGGGAAAGAGGC | 0.144906 GAGGTGGACAAGATGGAGGC | AGTCAAGCTGGGAAAGAGGC | 0.207079 CGCACTTTATCACCCATGCG | ATTGATCGCCTGAGTGGACG | 0.207121 CGCACTTTATCACCCATGCG | GGAAGAGAGGGTTACGACGC | 0.286559 GAGGTGGACAAGATGGAGGC | ATTGATCGCCTGAGTGGACG | | | | | |
| NCU00289T1 | 3 | 5 | NCU00289T1\_input.txt | NCU00289T1\_output.txt | 0.357282 ATGACTGACGTTGGCCTTCC | ATCTTGTCCACCTCTGCTGC | 0.424051 ATTTGCTGTGATGCATGGCC | GGTCTTCGTCCTGGAACTCC | 0.565790 ATTTGCTGTGATGCATGGCC | CGTCCTGGAACTCCTTGTCG | 0.568562 ATGACTGACGTTGGCCTTCC | GGTCTTCGTCCTGGAACTCC | 0.569366 TCATGGAGGAAAGTTGGCGG | GGTCTTCGTCCTGGAACTCC | | | | | |
| NCU00290T0 | 4 | 5 | NCU00290T0\_input.txt | NCU00290T0\_output.txt | 0.141173 GTCTGGGTGAGACTGGATGC | GAGATGATCAGAGCCGTGGG | 0.141173 GTCTGGGTGAGACTGGATGC | GGAGATGATCAGAGCCGTGG | 0.208988 CATGGTCGGTACTGTCTGGG | GAGATGATCAGAGCCGTGGG | 0.208988 CATGGTCGGTACTGTCTGGG | GGAGATGATCAGAGCCGTGG | 0.211153 GTCTGGGTGAGACTGGATGC | AGTAATCTTCGAGCCAGGCG | | | | | |
| NCU00291T0 | 2 | 5 | NCU00291T0\_input.txt | NCU00291T0\_output.txt | 0.344432 ATGGATGCATGGGAAAGGGG | ATGTGGCATATGCGCAATCG | 0.616570 ATGGATGCATGGGAAAGGGG | ACGCAGGAACGAAAATCACG | 0.633647 AGGAACTGGATGCTATCGGC | ATGTGGCATATGCGCAATCG | 0.785411 ATGGATGCATGGGAAAGGGG | GGTAATACCGAGGCTCTGGG | 0.785411 ATGGATGCATGGGAAAGGGG | GGGTAATACCGAGGCTCTGG | | | | | |
| NCU00292T0 | 2 | 5 | NCU00292T0\_input.txt | NCU00292T0\_output.txt | 0.070014 TCTCAACCCTCCTCTCCTCG | AAGGAGCAGGACTGTGAAGC | 0.070285 TCTCAACCCTCCTCTCCTCG | GTTGGAAAGGAGGAGGACGG | 0.072897 CTCCCAACACCCTTGACTCC | AAGGAGCAGGACTGTGAAGC | 0.073168 CTCCCAACACCCTTGACTCC | GTTGGAAAGGAGGAGGACGG | 0.270179 TCTCAACCCTCCTCTCCTCG | CGGGAGAGTAGAGCGATTCG | | | | | |
| NCU00293T0 | 2 | 5 | NCU00293T0\_input.txt | NCU00293T0\_output.txt | 0.286293 TACAACGGAGAGGTTTGGGC | TTGGTGCCGGTTTGACTAGG | 0.642412 TACAACGGAGAGGTTTGGGC | ACTTTTCTTGGAGAGGCGCC | 0.989699 TACAACGGAGAGGTTTGGGC | GTGCCGGTTTGACTAGGTGG | 0.990085 GTTGTGGTGGTGATGGTTGC | TTGTGTGTAATGGCAGTGGC | 1.011813 TACAACGGAGAGGTTTGGGC | AGGGGTGGTGGACTATGTCG | | | | | |
| NCU00294T0 | 3 | 5 | NCU00294T0\_input.txt | NCU00294T0\_output.txt | 0.151157 CAGAGAAACGCTTGTCACGC | GTCTTTTGCCTCTTGTGCCG | 0.151157 CAGAGAAACGCTTGTCACGC | CGTCTTTTGCCTCTTGTGCC | 0.209076 GAATGGTGCCAGAGAAACGC | GTCTTTTGCCTCTTGTGCCG | 0.209076 GAATGGTGCCAGAGAAACGC | CGTCTTTTGCCTCTTGTGCC | 0.483936 CGAATGGTGCCAGAGAAACG | GTCTTTTGCCTCTTGTGCCG | | | | | |
| NCU00295T0 | 2 | 5 | NCU00295T0\_input.txt | NCU00295T0\_output.txt | 0.629567 CTAGCGGTTCCTTGTTTGGC | TCAAGGGGTGGAGTTTGTCG | 0.629584 CTAGCGGTTCCTTGTTTGGC | GGTATGGTTGGGAGGACACG | 1.046552 CTAGCGGTTCCTTGTTTGGC | TGTAGCTAGCTGTTACGCGC | 1.171094 GGATTGACATCAAGGTGCAGC | TGGCTTCAGCAAGAGAAGGG | 1.241621 GGATTGACATCAAGGTGCAGC | TCAAGGGGTGGAGTTTGTCG | | | | | |
| NCU00296T0 | 1 | 5 | NCU00296T0\_input.txt | NCU00296T0\_output.txt | 0.431022 TCCTTCGTCATTGGCGTAGG | GAAGATCCCAACACCTCCCC | 0.644762 TCCTTCGTCATTGGCGTAGG | TCGCATCCCCAAGAAGATCC | 0.706960 GGTGTATTTTGCAGGGCTGG | TCCTACGCCAATGACGAAGG | 0.710851 TCCTTCGTCATTGGCGTAGG | CGCATCCCCAAGAAGATCCC | 0.788292 GGTGTATTTTGCAGGGCTGG | GAAGATCCCAACACCTCCCC | | | | | |
| NCU00297T0 | 1 | 5 | NCU00297T0\_input.txt | NCU00297T0\_output.txt | 0.355759 TGTAGGGTTCTTCGGCTTGG | AATCCGACGATGATGGACGG | 0.490155 CGTCTGTAGGGTTCTTCGGC | AATCCGACGATGATGGACGG | 0.490155 GCGTCTGTAGGGTTCTTCGG | AATCCGACGATGATGGACGG | 0.558792 AGCGTCTGTAGGGTTCTTCG | AATCCGACGATGATGGACGG | 0.637026 ACTTTGACCGGGACTTTGGG | CCGAAGAACCCTACAGACGC | | | | | |
| NCU00298T0 | 2 | 5 | NCU00298T0\_input.txt | NCU00298T0\_output.txt | 0.863829 ATCTTGAGGCTCAGGAGTGC | GAGGGGGAAGCTTTCTGTGG | 0.992706 ATCTTGAGGCTCAGGAGTGC | GCTGATTGCGTTTACAGGGG | 0.992706 ATCTTGAGGCTCAGGAGTGC | GGCTGATTGCGTTTACAGGG | 0.992706 ATCTTGAGGCTCAGGAGTGC | GGGCTGATTGCGTTTACAGG | 1.014320 AGGATGGAGGCAGTGAGTCC | GAGGGGAGTGGGAAATGTGG | | | | | |
| NCU00299T0 | 2 | 5 | NCU00299T0\_input.txt | NCU00299T0\_output.txt | 0.569316 AATGTCGAGTACAAGGGCCG | GTGGATGGCGTCTTGAGAGG | 0.574796 TACCCTGATCATTGGCACCG | TGGGGTGCGTATAAAAGGCC | 0.641158 TACCCTGATCATTGGCACCG | GTGGATGGCGTCTTGAGAGG | 0.782012 TACCCTGATCATTGGCACCG | TGAAGTCGGCGAAGGAAACC | 0.850847 ACTACGATCTGGCTCAACGG | TGGGGTGCGTATAAAAGGCC | | | | | |
| NCU00300T0 | 2 | 5 | NCU00300T0\_input.txt | NCU00300T0\_output.txt | 0.282109 TGAGGATTCATGAGCGGTCG | TCTCCGACCAAATGATCGCC | 0.429293 CCCTGAGGATTCATGAGCGG | TCTCCGACCAAATGATCGCC | 0.558841 TGAGGATTCATGAGCGGTCG | GACCAAATGATCGCCCAACC | 0.628472 CACTTCAATTGAGAGCCGCC | TCTCCGACCAAATGATCGCC | 0.706025 CCCTGAGGATTCATGAGCGG | GACCAAATGATCGCCCAACC | | | | | |
| NCU00301T0 | 2 | 5 | NCU00301T0\_input.txt | NCU00301T0\_output.txt | 0.359278 GTGAAGACGCTGCCAAAAGG | CAGGCTTGCACACACAATGG | 0.478139 GCGACTGAGAAACGTTGACG | CAGGCTTGCACACACAATGG | 0.497102 GTGAAGACGCTGCCAAAAGG | CATGGCCTCAACGGAAATGG | 0.560156 GTGTTGGATGGAGTTTGGCG | CAGGCTTGCACACACAATGG | 0.560156 CGTGTTGGATGGAGTTTGGC | CAGGCTTGCACACACAATGG | | | | | |
| NCU00302T0 | 2 | 5 | NCU00302T0\_input.txt | NCU00302T0\_output.txt | 0.289586 TGAAAACCCTCAGCACCTCC | AGACGAGGATGACATTGGCG | 0.427327 TGAAAACCCTCAGCACCTCC | TTTGCGAAAGTTGGATGGCC | 0.785501 AAACCACATGCCAGTCGAGG | AGACGAGGATGACATTGGCG | 0.848201 TGAAAACCCTCAGCACCTCC | CGACGAGGGCTTCTTCAAGG | 0.860275 AGCTCCAAAAACCACATGCC | AGACGAGGATGACATTGGCG | | | | | |
| NCU00303T0 | 3 | 5 | NCU00303T0\_input.txt | NCU00303T0\_output.txt | 0.066771 CATCTTATCACCCTCCGCCC | TGAGATGAGCGAGCGATTCC | 0.074713 CATCTTATCACCCTCCGCCC | TTCTGCGCAGTTGAATTGGC | 0.134186 CATCTTATCACCCTCCGCCC | GACGACATCGGCCATTTTGG | 1.079915 TCATCTTATCACCCTCCGCC | TGAGATGAGCGAGCGATTCC | 1.087857 TCATCTTATCACCCTCCGCC | TTCTGCGCAGTTGAATTGGC | | | | | |
| NCU00304T0 | 2 | 5 | NCU00304T0\_input.txt | NCU00304T0\_output.txt | 0.357068 GCTGGGGTACTCATGACTGG | ACCAGTCACGGTATGATGGC | 0.480687 GCTGGGGTACTCATGACTGG | CTGAACACATACATCCGCGC | 0.499190 ACTGCCTTTCTCTTCGCTGG | ACCAGTCACGGTATGATGGC | 0.560583 CTCGACGGAGGTGAAGATCC | ACCAGTCACGGTATGATGGC | 0.579593 GCTGGGGTACTCATGACTGG | GTATGATGGCCCCTGTACCC | | | | | |
| NCU00305T0 | 2 | 5 | NCU00305T0\_input.txt | NCU00305T0\_output.txt | 0.417343 CACGTTTCAACACCATGGCC | GATCGGTGGTGGATTTTGCG | 0.419426 ACAAACCAGTAGCGACCTCC | GATCGGTGGTGGATTTTGCG | 0.481960 CGGGAGATTTGCATTCAGCC | GATCGGTGGTGGATTTTGCG | 0.482318 TGCACGTTTCAACACCATGG | GATCGGTGGTGGATTTTGCG | 0.489991 CAAACCAGTAGCGACCTCCC | GATCGGTGGTGGATTTTGCG | | | | | |
| NCU00306T0 | 2 | 5 | NCU00306T0\_input.txt | NCU00306T0\_output.txt | 1.289040 TTTGTCAGTAACCAGGGGGC | GAAAGGTGGTTGATGAGCCC | 1.289040 TTTGTCAGTAACCAGGGGGC | GGAAAGGTGGTTGATGAGCC | 1.289040 TTTGTCAGTAACCAGGGGGC | GGGAAAGGTGGTTGATGAGC | 1.435017 TGGTAAACAGCCATAGCCCC | GAAAGGTGGTTGATGAGCCC | 1.435017 TGGTAAACAGCCATAGCCCC | GGAAAGGTGGTTGATGAGCC | | | | | |
| NCU00307T0 | 3 | 5 | NCU00307T0\_input.txt | NCU00307T0\_output.txt | 0.219312 ACTGGGAAGGCCTTGATTGG | ACGGAGCTTCGCTATTCACC | 0.358181 CAAGACGGGTATGAGCTGGG | ACGGAGCTTCGCTATTCACC | 0.358181 CCAAGACGGGTATGAGCTGG | ACGGAGCTTCGCTATTCACC | 0.413992 CGACGGTTTGAGAAAGTGGC | ACGGAGCTTCGCTATTCACC | 0.770766 GACGGTTTGAGAAAGTGGCC | ACGGAGCTTCGCTATTCACC | | | | | |
| NCU00308T0 | 1 | 5 | NCU00308T0\_input.txt | NCU00308T0\_output.txt | 0.142058 TTCTTTGAGGATGGCGGACC | TAAACACCACCACCACCACC | 0.214539 TTCTTTGAGGATGGCGGACC | CAGCATGTCCCTCTACCACG | 0.215044 ATGCGAGACTTGAGACTGCC | TAAACACCACCACCACCACC | 0.285727 GGCTAGTGCGTAGACTTGGG | TAAACACCACCACCACCACC | 0.285727 GGGCTAGTGCGTAGACTTGG | TAAACACCACCACCACCACC | | | | | |
| NCU00309T0 | 2 | 5 | NCU00309T0\_input.txt | NCU00309T0\_output.txt | 1.225449 CACTCAACGCTTACCAACGC | GGTTGGGATTCTTGATGCCC | 1.503793 CCTCTCTGACCACTCAACGC | GGTTGGGATTCTTGATGCCC | 1.576497 TTCATCGGAACAGGATGCGG | GGTTGGGATTCTTGATGCCC | 1.588381 TCCACCCATCCATTCATCGG | GGTTGGGATTCTTGATGCCC | 1.588927 CACTCAACGCTTACCAACGC | GGATTCTTGATGCCCAACACC | | | | | |
| NCU00310T0 | 1 | 5 | NCU00310T0\_input.txt | NCU00310T0\_output.txt | 0.212152 CGGTAGACTCCATCAGTGGC | AGCTGTTTACGGTCCTACGC | 0.287752 AGAGCAATTGTCTCGGTCCG | TATCACTCAACCAGCTCGCG | 0.425216 CGGTAGACTCCATCAGTGGC | CAGGAAAGGCCTCACAGTCC | 0.493440 ATTCGCCGATTCCTAGACCG | TATCACTCAACCAGCTCGCG | 0.499695 AGCTAAAAGGGTCCCGTTGG | CTGAGGAATCGGTCTGGTCC | | | | | |
| NCU00311T1 | 4 | 5 | NCU00311T1\_input.txt | NCU00311T1\_output.txt | 0.494955 GCGTGAATTTGGCCTTCTCC | TGAAAGCCCAGATCCCAAGC | 0.546685 GCGTGAATTTGGCCTTCTCC | TTGCGATACGGAATTTGCGG | 0.716802 ACCCGAGTGTCTCTTGTTGG | TGAAAGCCCAGATCCCAAGC | 0.768531 ACCCGAGTGTCTCTTGTTGG | TTGCGATACGGAATTTGCGG | 0.771526 CGTGAATTTGGCCTTCTCCG | TGAAAGCCCAGATCCCAAGC | | | | | |
| NCU00311T0 | 5 | 5 | NCU00311T0\_input.txt | NCU00311T0\_output.txt | 0.494955 GCGTGAATTTGGCCTTCTCC | TGAAAGCCCAGATCCCAAGC | 0.546685 GCGTGAATTTGGCCTTCTCC | TTGCGATACGGAATTTGCGG | 0.716802 ACCCGAGTGTCTCTTGTTGG | TGAAAGCCCAGATCCCAAGC | 0.768531 ACCCGAGTGTCTCTTGTTGG | TTGCGATACGGAATTTGCGG | 0.771526 CGTGAATTTGGCCTTCTCCG | TGAAAGCCCAGATCCCAAGC | | | | | |
| NCU00312T0 | 2 | 5 | NCU00312T0\_input.txt | NCU00312T0\_output.txt | 1.384190 GAAAATCTCCAGTCCCCTCCC | CGGGTGATGTGACAAATGCC | 1.459859 AATCCATGCCGTCTACGCC | GTGACGTCGAGTTTTGCTGG | 1.459859 AATCCATGCCGTCTACGCC | CTGGTGACGTCGAGTTTTGC | 1.470947 AAAATCTCCAGTCCCCTCCC | CGGGTGATGTGACAAATGCC | 1.540109 GAAAATCTCCAGTCCCCTCCC | ATGGTAGGGGTGGTTGTTCG | | | | | |
| NCU00313T0 | 1 | 5 | NCU00313T0\_input.txt | NCU00313T0\_output.txt | 0.281692 GGGCGATAAAATGCAGCTGG | AACACCTCCAGATTCCTCGC | 0.287764 GTGAAAGCGAGCAGAATGCC | TGCTCCCTCTTCACAACACC | 0.351861 TGGACATGAACCTCAGCACG | CCAGCTGCATTTTATCGCCC | 0.357973 AGATTCTGAAAGCCGAGGCC | AACACCTCCAGATTCCTCGC | 0.429268 TGGACATGAACCTCAGCACG | TGCTCCCTCTTCACAACACC | | | | | |
| NCU00314T0 | 1 | 5 | NCU00314T0\_input.txt | NCU00314T0\_output.txt | 0.135709 TCGATCTAAACGGCTCTGGC | CCTCGAGACTCATCATGGCC | 0.136667 TCGATCTAAACGGCTCTGGC | TCATCATGGCCACACCTTCC | 0.208301 GAGTGGTGACCGTATGGACC | CCTCGAGACTCATCATGGCC | 0.209259 GAGTGGTGACCGTATGGACC | TCATCATGGCCACACCTTCC | 0.212170 TCGATCTAAACGGCTCTGGC | CGCTCGATAATCCCTCGACC | | | | | |
| NCU00315T0 | 4 | 5 | NCU00315T0\_input.txt | NCU00315T0\_output.txt | 0.974069 GTGGTCTTGTTGCGAATCGG | TCATTGCGATGTTTTCCCGG | 1.047019 GAGAGCATGACGATGGTTGC | TTCAATGGTTTGCCCTGTGC | 1.265648 CGTGGAGAGCATGACGATGG | TTCAATGGTTTGCCCTGTGC | 1.338124 GTGGTCTTGTTGCGAATCGG | TGCACTTACCAAGGCAGTGC | 1.367125 GTGGTCTTGTTGCGAATCGG | TTCATTGCGATGTTTTCCCGG | | | | | |
| NCU00316T0 | 4 | 5 | NCU00316T0\_input.txt | NCU00316T0\_output.txt | 0.619480 AATCGACTTGGACAATGCGC | TTCTGCTGCATTTGATGGCG | 0.623791 AATCGACTTGGACAATGCGC | CCAAGTAGACGCTCCCTTCC | 0.906568 GATGGTGGCAATCGACTTGG | TTCTGCTGCATTTGATGGCG | 1.277964 GGATTGCAGGCCTACTTTGG | TTCTGCTGCATTTGATGGCG | 1.473869 TGAACAAACTCAAAGCGCCC | CATCTTTCAGCCGAACCTGG | | | | | |
| NCU00317T0 | 5 | 5 | NCU00317T0\_input.txt | NCU00317T0\_output.txt | 0.341361 CGGCATTGATCGAGAAAGCC | TCCTCACGGGAGAATTTGCC | 0.341361 CCGGCATTGATCGAGAAAGC | TCCTCACGGGAGAATTTGCC | 0.404299 CGGCATTGATCGAGAAAGCC | GGTACCGAGTGTCACTAGCG | 0.404299 CGGCATTGATCGAGAAAGCC | CGGTACCGAGTGTCACTAGC | 0.404299 CCGGCATTGATCGAGAAAGC | GGTACCGAGTGTCACTAGCG | | | | | |
| NCU00317T1 | 4 | 5 | NCU00317T1\_input.txt | NCU00317T1\_output.txt | 0.341361 CGGCATTGATCGAGAAAGCC | TCCTCACGGGAGAATTTGCC | 0.341361 CCGGCATTGATCGAGAAAGC | TCCTCACGGGAGAATTTGCC | 0.404299 CGGCATTGATCGAGAAAGCC | GGTACCGAGTGTCACTAGCG | 0.404299 CGGCATTGATCGAGAAAGCC | CGGTACCGAGTGTCACTAGC | 0.404299 CCGGCATTGATCGAGAAAGC | GGTACCGAGTGTCACTAGCG | | | | | |
| NCU00318T0 | 3 | 5 | NCU00318T0\_input.txt | NCU00318T0\_output.txt | 1.984609 TTGAAGAATGCAAGCACGGC | AGTATGGGACGCGACAGTGG | 2.167291 TTGAAGAATGCAAGCACGGC | GATCTTGAAGTATGGGACGCG | 2.259341 CTTGCCCCAAAACGTCTTCC | AGTATGGGACGCGACAGTGG | 2.263085 TTGAAGAATGCAAGCACGGC | AGATCTTGAAGTATGGGACGCG | 2.264324 TTGAAGAATGCAAGCACGGC | ATCTTGAAGTATGGGACGCG | | | | | |
| NCU00319T0 | 3 | 5 | NCU00319T0\_input.txt | NCU00319T0\_output.txt | 0.342855 GCCTTGGTAGACAGAGGAGC | CAACCGCTAGTTTCAAGGCG | 0.356034 GCCTTGGTAGACAGAGGAGC | CTTCCTCAGCCGTACTCTGC | 0.498209 GCCTTGGTAGACAGAGGAGC | ATATATGCCCGTCCAACCGC | 0.698646 GCCTTGGTAGACAGAGGAGC | CCAACCGCTAGTTTCAAGGC | 0.705138 CTCTTGGCTGATTTGTGGCC | TCAGGCAGCTTCGATACACC | | | | | |
| NCU00320T2 | 6 | 5 | NCU00320T2\_input.txt | NCU00320T2\_output.txt | 0.211810 GCAAGTACTTCCCTCCTCCG | TCAAGCAACATACCGGACCC | 0.283644 GCAAGTACTTCCCTCCTCCG | CGGACCCATCTTCTTCCACC | 0.356241 TCTGTGCGGCAAGTACTTCC | TCAAGCAACATACCGGACCC | 0.361210 AAGTACTTCCCTCCTCCGGG | TCAAGCAACATACCGGACCC | 0.419084 GCAAGTACTTCCCTCCTCCG | ACCTCGTCTACCTTCAACGC | | | | | |
| NCU00320T0 | 6 | 5 | NCU00320T0\_input.txt | NCU00320T0\_output.txt | 0.217435 GTGGAAGAAGATGGGTCCGG | AAACACATCAACGATGCCGC | 0.217435 GGTGGAAGAAGATGGGTCCG | AAACACATCAACGATGCCGC | 0.284334 GTGGAAGAAGATGGGTCCGG | TCTCCATGAAAGCTCCTCGC | 0.284334 GGTGGAAGAAGATGGGTCCG | TCTCCATGAAAGCTCCTCGC | 0.426095 GTGGAAGAAGATGGGTCCGG | TGAACTCCTCTGCCTTGTCG | | | | | |
| NCU00320T1 | 7 | 5 | NCU00320T1\_input.txt | NCU00320T1\_output.txt | 0.217435 GTGGAAGAAGATGGGTCCGG | AAACACATCAACGATGCCGC | 0.217435 GGTGGAAGAAGATGGGTCCG | AAACACATCAACGATGCCGC | 0.284334 GTGGAAGAAGATGGGTCCGG | TCTCCATGAAAGCTCCTCGC | 0.284334 GGTGGAAGAAGATGGGTCCG | TCTCCATGAAAGCTCCTCGC | 0.426095 GTGGAAGAAGATGGGTCCGG | TGAACTCCTCTGCCTTGTCG | | | | | |
| NCU00321T0 | 1 | 5 | NCU00321T0\_input.txt | NCU00321T0\_output.txt | 0.430884 GAGAGATGGGAAGTGTCGCC | ATTCACCAAGCCTCAACCCC | 0.505183 ATTACGAGGGGAGAGTGGGG | CCATCCATCCCCAGTGAACC | 0.577732 ATTACGAGGGGAGAGTGGGG | TCCCATCGCATAACCGAACC | 0.646756 GAGAGATGGGAAGTGTCGCC | ACTCTCCCCTCGTAATCGGG | 0.727607 GAGAGATGGGAAGTGTCGCC | CAAGAATCCCCACTCTCCCC | | | | | |
| NCU00322T0 | 2 | 5 | NCU00322T0\_input.txt | NCU00322T0\_output.txt | 0.358384 TCGATGGCAAGAGAGAAGCG | GTTTCGACGCTTTGCTACGG | 1.010433 TCGATGGCAAGAGAGAAGCG | AGGAGTTAATCTGACCGGGC | 1.054563 TTATATCGGAGAGGCGCAGG | CAGAATGGGTGTTTCGACGC | 1.067876 TTATATCGGAGAGGCGCAGG | GTTTCGACGCTTTGCTACGG | 1.081602 TGGTATTAGGCCAGCGATGC | AGGAGTTAATCTGACCGGGC | | | | | |
| NCU00324T0 | 3 | 5 | NCU00324T0\_input.txt | NCU00324T0\_output.txt | 0.415225 ATAACCGTTCTCCTACGCCG | TCAAAGCGAAGGCAAAAGGC | 0.422758 ATAACCGTTCTCCTACGCCG | AAAGGTCCGCATTTTCGTGC | 0.503181 TTTGATGAGGGCGGGATTCG | AAAGGTCCGCATTTTCGTGC | 0.647562 TGGGTTCGTTTGATGAGGGC | AAAGGTCCGCATTTTCGTGC | 0.838188 ATAACCGTTCTCCTACGCCG | CCGCATTTTCGTGCTTCTGG | | | | | |
| NCU00325T0 | 1 | 5 | NCU00325T0\_input.txt | NCU00325T0\_output.txt | 0.278885 ACCTACAATCAAGAGCCGCC | GATCAGGTTTTTGAGGCGGC | 0.283244 AAACTGCCGCCTCAAAAACC | GACGCATGAGAGAGGACACC | 0.285027 ACCTACAATCAAGAGCCGCC | GGGTATCCGAACCAGTCACC | 0.285217 AGCAAATTGATACCCGGGGG | AGGCGGCTCTTGATTGTAGG | 0.285373 AGCAAATTGATACCCGGGGG | CGACAATGGTGGCTGAATGC | | | | | |
| NCU00326T0 | 3 | 5 | NCU00326T0\_input.txt | NCU00326T0\_output.txt | 0.485352 CGTTGACGATGACTGGTTGC | TGGTGAAAGCTGATGGACGG | 0.838880 GTTGACGATGACTGGTTGCC | TGGTGAAAGCTGATGGACGG | 0.901503 CGTTGACGATGACTGGTTGC | GCTGATGGACGGCGATATCC | 0.992727 TACTAAGTGGGCGTTTGGCG | TGGTGAAAGCTGATGGACGG | 1.034845 CGTTGACGATGACTGGTTGC | GGCGATATCCAGAGCTTTGC | | | | | |
| NCU00326T1 | 3 | 5 | NCU00326T1\_input.txt | NCU00326T1\_output.txt | 0.485352 CGTTGACGATGACTGGTTGC | TGGTGAAAGCTGATGGACGG | 0.838880 GTTGACGATGACTGGTTGCC | TGGTGAAAGCTGATGGACGG | 0.901503 CGTTGACGATGACTGGTTGC | GCTGATGGACGGCGATATCC | 0.992727 TACTAAGTGGGCGTTTGGCG | TGGTGAAAGCTGATGGACGG | 1.034845 CGTTGACGATGACTGGTTGC | GGCGATATCCAGAGCTTTGC | | | | | |
| NCU00327T0 | 3 | 5 | NCU00327T0\_input.txt | NCU00327T0\_output.txt | 0.141944 TCCTTCGTTTCCATCCGTCG | TGACACGGTACAGCAGAACC | 0.432443 TCCTTCGTTTCCATCCGTCG | CCCCTAGTGTTGAAAGCCCC | 0.497641 TCCTTCGTTTCCATCCGTCG | CAGGTCTGACACGGTACAGC | 0.513418 TCCTTCGTTTCCATCCGTCG | ACCCCTAGTGTTGAAAGCCC | 0.778617 TCCTTCGTTTCCATCCGTCG | GACACGGTACAGCAGAACCC | | | | | |
| NCU00329T0 | 3 | 5 | NCU00329T0\_input.txt | NCU00329T0\_output.txt | 0.339417 GCTGACACGTTGGTTGTACG | ACGGGAAAGAGCTTGGATCG | 0.352044 TGTGGCTCTTGTCTCTACGC | ACGGGAAAGAGCTTGGATCG | 0.483476 GCTGACACGTTGGTTGTACG | ATAAACCCGGAGCTGACACC | 0.566854 GTGGCTCTTGTCTCTACGCC | ACGGGAAAGAGCTTGGATCG | 0.619573 GCTGACACGTTGGTTGTACG | AGCGAGTATAAACCCGGAGC | | | | | |
| NCU00330T0 | 1 | 5 | NCU00330T0\_input.txt | NCU00330T0\_output.txt | 0.215593 ATCATGAGGGATGTGACGGC | ACCGCAGTACAAGATCCAGC | 0.286637 ATCATGAGGGATGTGACGGC | CATTGCGTCACACCATCAGC | 0.286671 ATCATGAGGGATGTGACGGC | GATCCAGCAAACTCGCAACG | 0.340530 ATCATGAGGGATGTGACGGC | ATAGCGCGTTTGATCATGCG | 0.341259 TGCTGGATCTTGTACTGCGG | ATAGCGCGTTTGATCATGCG | | | | | |
| NCU00331T0 | 3 | 5 | NCU00331T0\_input.txt | NCU00331T0\_output.txt | 0.354626 TGTGCTGAACTCCGTCTTCC | CAATGCGATAGAGGAGCCCC | 0.428069 TGTGCTGAACTCCGTCTTCC | ACAATGCGATAGAGGAGCCC | 0.429689 GAACTCCGTCTTCCAGAGCC | CAATGCGATAGAGGAGCCCC | 0.500206 ATATGCTCACGACCGGTTGG | CAATGCGATAGAGGAGCCCC | 0.503132 GAACTCCGTCTTCCAGAGCC | ACAATGCGATAGAGGAGCCC | | | | | |
| NCU00332T0 | 3 | 5 | NCU00332T0\_input.txt | NCU00332T0\_output.txt | 0.729781 GCATACGATCGGTAGAGGCC | TTTGGGCTGCTAGGAAGAGG | 0.729781 GGCATACGATCGGTAGAGGC | TTTGGGCTGCTAGGAAGAGG | 0.914215 GCATACGATCGGTAGAGGCC | CAAGCGCTTGAAAGGGTAGG | 0.914215 GGCATACGATCGGTAGAGGC | CAAGCGCTTGAAAGGGTAGG | 1.063678 GCATACGATCGGTAGAGGCC | AAGACAAGCGCTTGAAAGGG | | | | | |
| NCU00333T1 | 3 | 5 | NCU00333T1\_input.txt | NCU00333T1\_output.txt | 1.579973 CAGCTTCCGGAAACTATTGCC | CAGGTACATGTCGGGAAGGG | 1.866278 CAGCTTCCGGAAACTATTGCC | CATGTCGGGAAGGGTGATGG | 1.908493 TTTCACACGCCCTTGTCAGC | CATGTCGGGAAGGGTGATGG | 1.929505 AGCTTCCGGAAACTATTGCC | CAGGTACATGTCGGGAAGGG | 1.939093 CAGCTTCCGGAAACTATTGCC | AGCTTCAGGTACATGTCGGG | | | | | |
| NCU00333T0 | 2 | 5 | NCU00333T0\_input.txt | NCU00333T0\_output.txt | 1.579973 CAGCTTCCGGAAACTATTGCC | CAGGTACATGTCGGGAAGGG | 1.866278 CAGCTTCCGGAAACTATTGCC | CATGTCGGGAAGGGTGATGG | 1.908493 TTTCACACGCCCTTGTCAGC | CATGTCGGGAAGGGTGATGG | 1.929505 AGCTTCCGGAAACTATTGCC | CAGGTACATGTCGGGAAGGG | 1.939093 CAGCTTCCGGAAACTATTGCC | AGCTTCAGGTACATGTCGGG | | | | | |
| NCU00334T0 | 2 | 5 | NCU00334T0\_input.txt | NCU00334T0\_output.txt | 0.364351 CAAGCGATACATTGGCGACG | TTTGCTTCTACGCCACTCCC | 0.433392 TCTGGGCATGTTGACTGAGG | GTCCCCAAGTCCTCATCTGC | 0.497513 TGACGGTTCTGCTTCTCTGG | TCCTCATCTGCCGCTAAAGC | 0.505006 TCTGGGCATGTTGACTGAGG | TCCTCATCTGCCGCTAAAGC | 0.569544 GACGGTTCTGCTTCTCTGGG | TCCTCATCTGCCGCTAAAGC | | | | | |
| NCU00335T0 | 2 | 5 | NCU00335T0\_input.txt | NCU00335T0\_output.txt | 0.284610 CCACAGCTCACAGACCTACC | AACTTGAGCTGGGTGTAGGC | 0.357997 CCCATCTCGCCCTCTATTGC | AACTTGAGCTGGGTGTAGGC | 0.359849 ATGACCACAGCTCACAGACC | AACTTGAGCTGGGTGTAGGC | 0.705587 CCACAGCTCACAGACCTACC | CAGAGGTAAGAGCAGGTCCG | 0.716433 CCCATCTCGCCCTCTATTGC | TAGGAGACGCTGGTGGTAGG | | | | | |
| NCU00336T0 | 2 | 5 | NCU00336T0\_input.txt | NCU00336T0\_output.txt | 0.442724 AAACTTGGAGGAGGAGGTGC | GCCATTCATTAGCGAGCTGC | 0.579585 ATGTTTTCGGAGAGTGCCCC | ACAGGAGATGGATTGTGGGC | 0.644110 ATGTTTTCGGAGAGTGCCCC | TTGTGGGCATTGAGGAGACG | 0.659160 AAACTTGGAGGAGGAGGTGC | ACAGGAGATGGATTGTGGGC | 0.723684 AAACTTGGAGGAGGAGGTGC | TTGTGGGCATTGAGGAGACG | | | | | |
| NCU00337T0 | 1 | 5 | NCU00337T0\_input.txt | NCU00337T0\_output.txt | 0.357830 GGAAGATCAGCTCGCTCTGG | ATGTCTCCTCAACAGCTCGC | 0.359923 GACAGTGGAGGGTAGCAACC | TCCGACATCACCCTTTCACC | 0.490582 TCAGCTCGCTCTGGAATACG | ATGTCTCCTCAACAGCTCGC | 0.498765 TCACGAAGCAGCTCATGTCC | ATGTCTCCTCAACAGCTCGC | 0.568445 TGCTCGTCAAGTGTCTCAGC | CCAGAGCGAGCTGATCTTCC | | | | | |
| NCU00338T0 | 2 | 5 | NCU00338T0\_input.txt | NCU00338T0\_output.txt | 0.501022 TGGGAATGGATCTTGACGGC | CCTTCCGGGTGTACCAATGG | 0.503151 ATGGATCTTGACGGCATCCC | CCTTCCGGGTGTACCAATGG | 0.616159 CTCAACAACGGAAGCCTTCG | GCCTGTGTCGAGATGTAGGG | 0.855077 TCAGTCCCAAAACCTGCTGC | GGTAGGTGCCGGAGTATTCG | 0.906760 CTCAACAACGGAAGCCTTCG | CCTTCCGGGTGTACCAATGG | | | | | |
| NCU00339T0 | 3 | 5 | NCU00339T0\_input.txt | NCU00339T0\_output.txt | 0.352255 GAGATTCTGGGACGTCGTCG | TCGGCCTGTCATGGTATTCG | 0.777765 ATTGTCGAGGCGGTCTTTGG | TCGGCCTGTCATGGTATTCG | 0.825838 GAAAGCCGTCATTGTCGAGG | TCGGCCTGTCATGGTATTCG | 0.909154 GAGATTCTGGGACGTCGTCG | AACTAACACACGGCAACAGC | 0.992856 TTTGGCGGCGATGGATATGG | TCGGCCTGTCATGGTATTCG | | | | | |
| NCU00340T0 | 4 | 5 | NCU00340T0\_input.txt | NCU00340T0\_output.txt | 1.654327 AAGTGTGCTGAGGTGTACGG | ACTTCCCTTTGTGTCATGCC | 2.078265 TCTGGCAGTATGAGCAGACG | ACTTCCCTTTGTGTCATGCC | 2.139922 AAGTGTGCTGAGGTGTACGG | GAACGAACTTTTGTTGTCCGC | 2.147364 AAGTGTGCTGAGGTGTACGG | TTGTGTCATGCCTCTTAGCC | 2.211018 GCAACTGAAGTGTGCTGAGG | ACTTCCCTTTGTGTCATGCC | | | | | |
| NCU00343T0 | 3 | 5 | NCU00343T0\_input.txt | NCU00343T0\_output.txt | 0.284588 TGCTGCTCATCTGTCTGACC | ACAAGAAGGCTGCTGAGTCC | 0.286103 GATCATTCCCCTCAGTCCGC | ACAAGAAGGCTGCTGAGTCC | 0.289295 ATGTCATTCCTCGACCAGCC | TTGTCTGGTGGTGGACATGG | 0.358860 TGCTGCTCATCTGTCTGACC | TTGTCTGGTGGTGGACATGG | 0.360376 GATCATTCCCCTCAGTCCGC | TTGTCTGGTGGTGGACATGG | | | | | |
| NCU00344T0 | 6 | 5 | NCU00344T0\_input.txt | NCU00344T0\_output.txt | 0.137441 AGCATCTCTTCGCTGTAGCC | TGGACGACTTTGACTGGAGC | 0.142103 AGCATCTCTTCGCTGTAGCC | TCTTGGTGGATTGGCAGACC | 0.352152 AGCATCTCTTCGCTGTAGCC | CAACGGCTCGAATGGAATGC | 0.488461 AGCATTTGATGACGTTGCGC | TGGACGACTTTGACTGGAGC | 0.499628 GGTCTGGATTTCGAGGAGGG | TGGACGACTTTGACTGGAGC | | | | | |
| NCU00344T1 | 6 | 5 | NCU00344T1\_input.txt | NCU00344T1\_output.txt | 0.067203 AGATTGGCTTTGGAGGGAGC | TGGACGACTTTGACTGGAGC | 0.137441 AGCATCTCTTCGCTGTAGCC | TGGACGACTTTGACTGGAGC | 0.142103 AGCATCTCTTCGCTGTAGCC | TCTTGGTGGATTGGCAGACC | 0.281913 AGATTGGCTTTGGAGGGAGC | CAACGGCTCGAATGGAATGC | 0.352152 AGCATCTCTTCGCTGTAGCC | CAACGGCTCGAATGGAATGC | | | | | |
| NCU00345T0 | 2 | 5 | NCU00345T0\_input.txt | NCU00345T0\_output.txt | 0.279777 CATTCCACACCTCAGGTCCC | AAGATTTCTTTGGCAGCGGC | 0.279777 CCATTCCACACCTCAGGTCC | AAGATTTCTTTGGCAGCGGC | 0.357052 CATTCCACACCTCAGGTCCC | GACTCAAGGGGACACAGACC | 0.357052 CCATTCCACACCTCAGGTCC | GACTCAAGGGGACACAGACC | 0.358030 CATTCCACACCTCAGGTCCC | CTTGTCTGGTGACTGGAGGG | | | | | |
| NCU00346T0 | 4 | 5 | NCU00346T0\_input.txt | NCU00346T0\_output.txt | 0.537742 GCATTTCGTACAGCAGACCG | CTCACTGCTGCCATCAAACG | 0.619511 GCATTTCGTACAGCAGACCG | AACGATCTGATGCTCTCCGC | 0.894868 GGCATTTCGTACAGCAGACC | CTCACTGCTGCCATCAAACG | 0.970464 TTACTACGCCGGCATGAACG | CTCACTGCTGCCATCAAACG | 0.970683 TTACTACGCCGGCATGAACG | GCTGCTGTTGCTACTCTTGC | | | | | |
| NCU00347T0 | 2 | 5 | NCU00347T0\_input.txt | NCU00347T0\_output.txt | 0.359521 GAGTTGAACTCGGACGAGGG | TGAGGGTCTGAGTTTTGGCG | 0.359521 GGAGTTGAACTCGGACGAGG | TGAGGGTCTGAGTTTTGGCG | 0.571805 CAGTGGATACCATGGGTCCG | ATTTGGAGGATGAGCGCAGG | 0.644115 GCAGTGGATACCATGGGTCC | ATTTGGAGGATGAGCGCAGG | 0.714712 AGATGTCGATGCGGATGTGG | ATTTGGAGGATGAGCGCAGG | | | | | |
| NCU00348T0 | 3 | 5 | NCU00348T0\_input.txt | NCU00348T0\_output.txt | 1.465173 GTTCTGGTAGTTGCCGAACG | AGGCGTTGACTAACATGGCC | 1.740431 TAGAACGCGTCCGAGATGC | AGGCGTTGACTAACATGGCC | 1.746688 CGCACTTGTTCTGGTAGTTGC | AGGCGTTGACTAACATGGCC | 1.772010 ACACCACGCACTTGTTCTGG | AGGCGTTGACTAACATGGCC | 1.877880 GTAGAACGCGTCCGAGATGC | AGGCGTTGACTAACATGGCC | | | | | |
| NCU00349T0 | 3 | 5 | NCU00349T0\_input.txt | NCU00349T0\_output.txt | 0.149207 TAGTCTGCGAACTGTCAGCG | TGGGTTGATGGTGGTGATGG | 0.280966 TAGTCTGCGAACTGTCAGCG | GACTTCATTCTTTGGCGGCC | 0.545952 GAGCGGATGGTAGGTTGTCC | CTTCGCGGACTTGTTCATCG | 0.572494 TAGTCTGCGAACTGTCAGCG | GGCGGGATCTCAGACTTTGG | 0.580819 GAGCGGATGGTAGGTTGTCC | TTCTTTGGTGAGCTCCCAGG | | | | | |
| NCU00350T0 | 2 | 5 | NCU00350T0\_input.txt | NCU00350T0\_output.txt | 0.499491 GACCCTCAAAGATGCCTCGG | TTGTGGAACTAGGCTGCTGG | 0.575783 AGACCCTCAAAGATGCCTCG | TTGTGGAACTAGGCTGCTGG | 0.634797 GGCAGCAACAACACCTTAGG | TTGTGGAACTAGGCTGCTGG | 0.707223 ACCATTCAGACGCGTAGTGG | GCTGCTGGTTGTGGAAAAGC | 0.713660 GACCCTCAAAGATGCCTCGG | AACTAGGCTGCTGGTTGTGG | | | | | |
| NCU00351T0 | 3 | 5 | NCU00351T0\_input.txt | NCU00351T0\_output.txt | 0.141702 GAGCATAGGGGTTCTGAGCC | CCCATGTCTACTCATCGCCC | 0.143191 ATACTTTTGCCTTGCAGCGC | CCCATGTCTACTCATCGCCC | 0.283403 GAGCATAGGGGTTCTGAGCC | TCTACTCATCGCCCAACAGC | 0.284892 ATACTTTTGCCTTGCAGCGC | TCTACTCATCGCCCAACAGC | 0.288993 GAGCATAGGGGTTCTGAGCC | ATCCATCCCTTCTTCACCGC | | | | | |
| NCU00352T0 | 3 | 5 | NCU00352T0\_input.txt | NCU00352T0\_output.txt | 0.207673 GCTGGCAATCCTCTGTTTGC | CCTGCGAAAAGGCATAACCG | 0.264673 CGTGGAAGTTTAGCAAGCGG | CCTGCGAAAAGGCATAACCG | 0.277078 CGTGGAAGTTTAGCAAGCGG | CACGATGCTGTGTTGTGTCG | 0.418779 GAGCCCGATCTTCTGGATCC | CCTGCGAAAAGGCATAACCG | 0.418975 GATCCAGATGCCTACGCTGG | CCTGCGAAAAGGCATAACCG | | | | | |
| NCU00353T0 | 5 | 5 | NCU00353T0\_input.txt | NCU00353T0\_output.txt | 0.834763 AGTGCCCGTTCATCTTGTCG | TGAAAACGCGTCGGTATTGC | 1.047144 TCCACCGGAAAACTTGCTCG | TGAAAACGCGTCGGTATTGC | 1.246321 CACCGGAAAACTTGCTCGC | TGAAAACGCGTCGGTATTGC | 1.255422 AGTGCCCGTTCATCTTGTCG | GAAGTCCCCACGTGAAAACG | 1.416391 AGTGCCCGTTCATCTTGTCG | AGACATCACATCCGCCAAGC | | | | | |
| NCU00354T0 | 2 | 5 | NCU00354T0\_input.txt | NCU00354T0\_output.txt | 0.215952 AGATGAACGTCCTGCCATCC | TTCTCTCTTTCTGCAGGGCG | 0.428255 AACTTCTCGCACGACCTTCC | ACGCCGTTCTGATTCTGAGG | 0.499474 CGATAGCCCAGACTGGAAGG | ACGCCGTTCTGATTCTGAGG | 0.581120 AGATGAACGTCCTGCCATCC | AGAGCTCAAAGGTGGGTTCC | 0.638793 TTTTGTCGAAGAGCGGACCC | TTCTCTCTTTCTGCAGGGCG | | | | | |
| NCU00355T0 | 3 | 5 | NCU00355T0\_input.txt | NCU00355T0\_output.txt | 0.134942 CAAGGACAAGCAAACACCGG | CGGAGTGAATAAGGTCGGGG | 0.134942 CCAAGGACAAGCAAACACCG | CGGAGTGAATAAGGTCGGGG | 0.145615 CAAGCAAACACCGGTCTTCG | CGGAGTGAATAAGGTCGGGG | 0.207780 CAAGGACAAGCAAACACCGG | TGAATAAGGTCGGGGAAGCG | 0.207780 CCAAGGACAAGCAAACACCG | TGAATAAGGTCGGGGAAGCG | | | | | |
| NCU00356T0 | 2 | 5 | NCU00356T0\_input.txt | NCU00356T0\_output.txt | 0.345312 CAGACGAGGAGTATGAGCCG | TGAAAGTAGGGAGCAGCAGC | 0.488320 CAGACGAGGAGTATGAGCCG | ATAGCGGATTGGGAGTTGGC | 0.488375 CAGACGAGGAGTATGAGCCG | AGTTGGCGAGGATCCATTCG | 0.492231 AGACATACGAAGACCAGGCG | TGAAAGTAGGGAGCAGCAGC | 0.554296 CAGACGAGGAGTATGAGCCG | CGGAGAGGATAGCGGATTGG | | | | | |
| NCU00357T0 | 2 | 5 | NCU00357T0\_input.txt | NCU00357T0\_output.txt | 2.333718 GGTATTCGAGTCCCCGATCG | TGCCACGAGTTCATTACAGG | 2.403581 AAGTTCACGTGCTGAGGTCG | TGAACCTGCATCATGTCAACG | 2.485100 GCGGAAAAGAGACCTGGAGG | TGCCACGAGTTCATTACAGG | 2.690944 AAGTTCACGTGCTGAGGTCG | TGCCACGAGTTCATTACAGG | 2.765087 GCAGCAGTGGTGACAGTAGG | TGCCACGAGTTCATTACAGG | | | | | |
| NCU00358T0 | 3 | 5 | NCU00358T0\_input.txt | NCU00358T0\_output.txt | 0.118706 GTCGTCTCGCAACTCAATGC | CGAAGCGATTTGTGTAGGCG | 0.204046 CGTAGGGCAGATTTCCAGGG | CGAAGCGATTTGTGTAGGCG | 0.215339 CGTAGGGCAGATTTCCAGGG | GGAGGAGTTTTGGTCGGAGG | 0.467421 GTCGTCTCGCAACTCAATGC | GAAGCGATTTGTGTAGGCGG | 0.552762 CGTAGGGCAGATTTCCAGGG | GAAGCGATTTGTGTAGGCGG | | | | | |
| NCU00359T0 | 2 | 5 | NCU00359T0\_input.txt | NCU00359T0\_output.txt | 0.542620 GATGACTGTGTACGGGAGGC | CATTGAGTTGCGAGACGACG | 0.647756 GATGACTGTGTACGGGAGGC | GGTCTTCCTGGGGGTTATCG | 0.691626 GATGACTGTGTACGGGAGGC | TTGCATTGAGTTGCGAGACG | 0.911988 GATGACTGTGTACGGGAGGC | ATTGAGTTGCGAGACGACGG | 0.981599 CGATATAGCTGGGCAAACGC | GGTCTTCCTGGGGGTTATCG | | | | | |
| NCU00360T0 | 2 | 5 | NCU00360T0\_input.txt | NCU00360T0\_output.txt | 0.841050 CGCCGCAAAGAAGCTTATCG | GGACGACATAGTCAGCACCG | 0.846126 CGCCGCAAAGAAGCTTATCG | TGGACGACATAGTCAGCACC | 1.053485 AAGAAGCTTATCGTCGCCGG | GGACGACATAGTCAGCACCG | 1.058560 AAGAAGCTTATCGTCGCCGG | TGGACGACATAGTCAGCACC | 1.381482 AGCTTCCATCATGTCGTCCG | GGATACGGAAGACCAGTTCGG | | | | | |
| NCU00361T0 | 1 | 5 | NCU00361T0\_input.txt | NCU00361T0\_output.txt | 0.407628 GAGGCTGATAGAACCGGTGG | AACAAGAAAACGACGTCGCC | 0.615004 CTCCGAGGCTGATAGAACCG | AACAAGAAAACGACGTCGCC | 0.693455 AGGACTGAGATGTTGCTGCG | AACAAGAAAACGACGTCGCC | 0.712741 TTGCTGCGAGGATGACTTGG | CAGTCCTCGTCAACTCCACC | 0.851151 AGGACTGAGATGTTGCTGCG | CGTGACCGATTCCAAGGAGG | | | | | |
| NCU00362T0 | 1 | 5 | NCU00362T0\_input.txt | NCU00362T0\_output.txt | 0.215215 ATGAGCAGGATGGTGGAAGC | ACCAAAATGCAAGGTTGGCC | 0.432169 AGGAAGTATTGGGGCCAACC | GCTTTGGCTAGCGTTACTGC | 0.441837 ATGAGCAGGATGGTGGAAGC | AGGTTGGCCCCAATACTTCC | 0.634521 AAAGGTCGCCCAGTTGAAGG | GCTTTGGCTAGCGTTACTGC | 0.654181 ATTTGAGATACCCGCGGACC | TTCCACCATCCTGCTCATCC | | | | | |
| NCU00363T0 | 3 | 5 | NCU00363T0\_input.txt | NCU00363T0\_output.txt | 0.502994 AGGTTCCGGGTAAGAGGAGG | GCAGGTCAGAGAGTGATGGG | 0.504487 AGGTTCCGGGTAAGAGGAGG | GATCAGAGTTGTCGGAGGGC | 0.638064 GTAAGAGGAGGCGGTTCTGC | GCAGGTCAGAGAGTGATGGG | 0.639557 GTAAGAGGAGGCGGTTCTGC | GATCAGAGTTGTCGGAGGGC | 0.651438 AGGTTCCGGGTAAGAGGAGG | AGGTCAGAGAGTGATGGGGG | | | | | |
| NCU00364T0 | 2 | 5 | NCU00364T0\_input.txt | NCU00364T0\_output.txt | 0.793102 GTCTTTGAGGAGGAGTCGCC | AACCTTGCCCCAAATTTCGG | 1.048450 TGATAAGCGAAGGAACGGCG | ACAACGTCGATCCACTCTGG | 1.079433 GAAGGGACAGGCAAGGATGG | AACCTTGCCCCAAATTTCGG | 1.180109 GTCTTTGAGGAGGAGTCGCC | AAACCTTGCCCCAAATTTCGG | 1.187764 GTCTTTGAGGAGGAGTCGCC | CTTGCCCCAAATTTCGGCC | | | | | |
| NCU00365T0 | 2 | 5 | NCU00365T0\_input.txt | NCU00365T0\_output.txt | 0.072585 TTTCCGGTCAACCATCTCCG | TTTACCACCCATCAGTCCGC | 0.206660 TACCATCCCGCAACTATCGC | CTGAGGAGGGTACTGAACGC | 0.210472 TTTCCGGTCAACCATCTCCG | CTGAGGAGGGTACTGAACGC | 0.428815 TACCATCCCGCAACTATCGC | TAGACTTCGCCTGAGGAGGG | 0.432627 TTTCCGGTCAACCATCTCCG | TAGACTTCGCCTGAGGAGGG | | | | | |
| NCU00366T0 | 1 | 5 | NCU00366T0\_input.txt | NCU00366T0\_output.txt | 0.069047 GAGGTCATCACCAAGTGGGG | TTCCTCCTCCTCAGACTCGG | 0.069047 GGAGGTCATCACCAAGTGGG | TTCCTCCTCCTCAGACTCGG | 0.497793 AAATGCGTGAAAGGGTTGCG | GTTTCTGGCATCGCTTGAGC | 0.643122 CACCAAGTGGGGTAGCAAGG | TTCCTCCTCCTCAGACTCGG | 0.652449 TCATCACCAAGTGGGGTAGC | TTCCTCCTCCTCAGACTCGG | | | | | |
| NCU00367T0 | 1 | 5 | NCU00367T0\_input.txt | NCU00367T0\_output.txt | 0.407140 GCTCGGCTTCATCTTCAAGC | TCGAGCACTTGCAAAAAGCC | 0.413548 GCTCGGCTTCATCTTCAAGC | CCAGCAAAATGCCCTCAACC | 0.482421 GCCGTAACTCTTGCCAATGG | TCGAGCACTTGCAAAAAGCC | 0.484253 CAGCTGCCTTCTCCTCTTCC | GCTTGAAGATGAAGCCGAGC | 0.487591 GCTCGGCTTCATCTTCAAGC | CGACAAACTCGAGCACTTGC | | | | | |
| NCU00368T0 | 1 | 5 | NCU00368T0\_input.txt | NCU00368T0\_output.txt | 0.144261 ACGCTCCTGTTCTTTCCTGG | TTCGCGGAATCAGACTCACC | 0.287765 AAGGTGAGTCTGATTCCGCG | CCTTGAGAACGAGCGAGAGG | 0.352710 TTCAAGAACCTCGAGCTCGG | TTCGCGGAATCAGACTCACC | 0.358104 AAGGTGAGTCTGATTCCGCG | CCGTTATCAGAAGGCCGAGG | 0.428444 ACGACGCTCCTGTTCTTTCC | TTCGCGGAATCAGACTCACC | | | | | |
| NCU00369T0 | 2 | 5 | NCU00369T0\_input.txt | NCU00369T0\_output.txt | 0.418440 TTGGTTCAGACACATTGCGC | TGGTACGGCACATCACATCC | 0.489577 TTGGTTCAGACACATTGCGC | GGCACATCACATCCTCCTCC | 0.774579 TTGGTTCAGACACATTGCGC | TACGGCACATCACATCCTCC | 0.850843 TTGTGTGCAATTGCCTGAGG | TGCTCTAACACTCCATCGGC | 0.924481 TTGTGTGCAATTGCCTGAGG | AACACTCCATCGGCAGTACC | | | | | |
| NCU00370T0 | 3 | 5 | NCU00370T0\_input.txt | NCU00370T0\_output.txt | 0.428470 CTGGACGATATAACCGGGCG | CATCGCAAGCAGATGGATGC | 0.709960 GCTGGACGATATAACCGGGC | CATCGCAAGCAGATGGATGC | 0.717624 TGCTGGACGATATAACCGGG | CATCGCAAGCAGATGGATGC | 0.906770 CTGGACGATATAACCGGGCG | TCTTGCTTGACATCGCAAGC | 0.992109 CTGGACGATATAACCGGGCG | GATATCGGCCCCTTCACAGC | | | | | |
| NCU00370T1 | 4 | 5 | NCU00370T1\_input.txt | NCU00370T1\_output.txt | 0.206280 GTCGCCAAGGTTGTCAATCG | CATCGCAAGCAGATGGATGC | 0.684580 GTCGCCAAGGTTGTCAATCG | TCTTGCTTGACATCGCAAGC | 0.766120 CAATCGTGAACCTGTTGCGC | CATCGCAAGCAGATGGATGC | 0.769919 GTCGCCAAGGTTGTCAATCG | GATATCGGCCCCTTCACAGC | 0.905447 TCAATCGTGAACCTGTTGCG | CATCGCAAGCAGATGGATGC | | | | | |
| NCU00371T0 | 2 | 5 | NCU00371T0\_input.txt | NCU00371T0\_output.txt | 0.710335 ACACGAAGCAGATAGACCGC | AGTTCGAAGCGACTTAGGGG | 0.719037 TGGGATCTCGAATTGGCTCC | TACATGACTGTTGGCTCCGG | 0.850431 GGATCTCGAATTGGCTCCGG | TACATGACTGTTGGCTCCGG | 0.850431 GGGATCTCGAATTGGCTCCG | TACATGACTGTTGGCTCCGG | 0.915869 GTATCTTCGGGGGAGTGACG | AGTTCGAAGCGACTTAGGGG | | | | | |
| NCU00372T0 | 4 | 5 | NCU00372T0\_input.txt | NCU00372T0\_output.txt | 0.500356 ATTGAGCAAGAAGGCCGAGG | ATGATGGCCTTGCTCTCACC | 0.630248 ATTGAGCAAGAAGGCCGAGG | CGATTCGATGATGGCCTTGC | 1.110173 TTGAGCAAGAAGGCCGAGG | ATGATGGCCTTGCTCTCACC | 1.240065 TTGAGCAAGAAGGCCGAGG | CGATTCGATGATGGCCTTGC | 1.241219 TATTGAGCAAGAAGGCCGAGG | ATGATGGCCTTGCTCTCACC | | | | | |
| NCU00373T0 | 1 | 5 | NCU00373T0\_input.txt | NCU00373T0\_output.txt | 0.278202 ATAGTCGTACCCGTCGTTGC | TGCATGCAGCTGTAGGTAGG | 0.353383 GCTCGTGAGGGAGAAGAAGG | TGCATGCAGCTGTAGGTAGG | 0.633190 ATAGTCGTACCCGTCGTTGC | GCATGCAGCTGTAGGTAGGG | 0.707479 ACAAGGTGAGCAAGGTGACG | CTTCTTCTCCCTCACGAGCC | 0.707479 ACAAGGTGAGCAAGGTGACG | CCTTCTTCTCCCTCACGAGC | | | | | |
| NCU00375T0 | 6 | 5 | NCU00375T0\_input.txt | NCU00375T0\_output.txt | 0.557595 CGATATCTTCGGAGCTCGGC | TGACTGGATACACATCGGCG | 0.629002 CGATATCTTCGGAGCTCGGC | CAGTCGTCCTCCCAGTTAGC | 0.634429 CGATATCTTCGGAGCTCGGC | GTCCCGTGAGAACAGATCGG | 0.702206 CGATATCTTCGGAGCTCGGC | GCTTCAGATTCGCGTTCACG | 0.721281 ATCTTGACGGGGTTTCCTCC | TGACTGGATACACATCGGCG | | | | | |
| NCU00376T1 | 5 | 5 | NCU00376T1\_input.txt | NCU00376T1\_output.txt | 1.980980 GGCGGTAGCTTGTGAAAAACG | CAAAAGAAGCTTGGCGTGGG | 1.987374 ATGTCTCATTTGGCGGTAGC | CAAAAGAAGCTTGGCGTGGG | 1.989280 GCGGTAGCTTGTGAAAAACG | CAAAAGAAGCTTGGCGTGGG | 2.047415 GGCGGTAGCTTGTGAAAAACG | ACAAAAGAAGCTTGGCGTGG | 2.053809 ATGTCTCATTTGGCGGTAGC | ACAAAAGAAGCTTGGCGTGG | | | | | |
| NCU00376T0 | 3 | 5 | NCU00376T0\_input.txt | NCU00376T0\_output.txt | 1.980980 GGCGGTAGCTTGTGAAAAACG | CAAAAGAAGCTTGGCGTGGG | 1.987374 ATGTCTCATTTGGCGGTAGC | CAAAAGAAGCTTGGCGTGGG | 1.989280 GCGGTAGCTTGTGAAAAACG | CAAAAGAAGCTTGGCGTGGG | 2.047415 GGCGGTAGCTTGTGAAAAACG | ACAAAAGAAGCTTGGCGTGG | 2.053809 ATGTCTCATTTGGCGGTAGC | ACAAAAGAAGCTTGGCGTGG | | | | | |
| NCU00377T0 | 2 | 5 | NCU00377T0\_input.txt | NCU00377T0\_output.txt | 0.419853 TCGTAGCGAACTCCATCACG | ATGCGTTGGTTGAGAGTCCC | 0.500714 CTCTGGCTGATTTCCGAGGG | ATGCGTTGGTTGAGAGTCCC | 0.500714 CCTCTGGCTGATTTCCGAGG | ATGCGTTGGTTGAGAGTCCC | 0.556743 TCGTAGCGAACTCCATCACG | CAAGGTGGTGAGATCGTCGG | 0.565708 AAGCAAATACCTCGGAGGCC | CAAGGTGGTGAGATCGTCGG | | | | | |
| NCU00378T0 | 2 | 5 | NCU00378T0\_input.txt | NCU00378T0\_output.txt | 0.136400 CAACGGCAAGAAGTGGATGC | GCGACTTCCATGATTCGTGC | 0.420140 CAACGGCAAGAAGTGGATGC | CGAGCAGATCTCCTCTTCGG | 0.420140 CAACGGCAAGAAGTGGATGC | CCGAGCAGATCTCCTCTTCG | 0.762189 CAACGGCAAGAAGTGGATGC | CGACTTCCATGATTCGTGCC | 0.919628 CAACGGCAAGAAGTGGATGC | ATACTGCGGCGTCAACATCC | | | | | |
| NCU00379T0 | 2 | 5 | NCU00379T0\_input.txt | NCU00379T0\_output.txt | 0.151955 CACGAGACGCAGAAAAAGGC | ATTGCGAGAACGTTGTGTGC | 0.151955 GCACGAGACGCAGAAAAAGG | ATTGCGAGAACGTTGTGTGC | 0.292609 TGGTGAATTGGGAGCTGTGG | ATTGCGAGAACGTTGTGTGC | 0.647984 AAGGAGAAGTTGAAGGCGGC | ATTGCGAGAACGTTGTGTGC | 0.931995 GAATTGGGAGCTGTGGTTGG | ATTGCGAGAACGTTGTGTGC | | | | | |
| NCU00380T0 | 3 | 5 | NCU00380T0\_input.txt | NCU00380T0\_output.txt | 0.558312 CAGGTGCTGAAGAGAGTGGG | GCTACGGGTTTGAGCTTTGG | 1.058544 CAGGTGCTGAAGAGAGTGGG | GTTGGTGGTGTGAGTTGTCC | 1.142291 ACCATTACACCAGGAGCAGG | GCTACGGGTTTGAGCTTTGG | 1.175833 CAGGTGCTGAAGAGAGTGGG | GGAACCGGGAGAAGAGAATCC | 1.184887 CAGGTGCTGAAGAGAGTGGG | GGTGTGAGTTGTCCATGTCG | | | | | |
| NCU00382T0 | 1 | 5 | NCU00382T0\_input.txt | NCU00382T0\_output.txt | 0.074474 AAGTTGCCCAAGAGGTAGCC | ATTTTTGCCTTTTCCCGCCC | 0.361519 TGGGTGTATCAAGGCAAGGC | ACAACCTACTCAGACGCAGC | 0.656768 AAGTTGCCCAAGAGGTAGCC | TTTCCTTCCATCCAGTCCCG | 0.701312 GCCCCTGATTATCAACGTGC | ACAACCTACTCAGACGCAGC | 0.705875 TGGGTGTATCAAGGCAAGGC | CTACTCAGACGCAGCCTACC | | | | | |
| NCU00384T0 | 2 | 5 | NCU00384T0\_input.txt | NCU00384T0\_output.txt | 0.427094 GCCAAAAGCAGGAAAGGTCG | ATGGGTAGCGTATGGTGTCG | 0.558340 GCCAAAAGCAGGAAAGGTCG | CGCGCCTCAAGTGAAATTCG | 0.567037 GTCTTGTCGGGGGAAGATCG | ATGGGTAGCGTATGGTGTCG | 0.698283 GTCTTGTCGGGGGAAGATCG | CGCGCCTCAAGTGAAATTCG | 0.705409 AGGAAAGGTCGGTCTTGTCG | ATGGGTAGCGTATGGTGTCG | | | | | |
| NCU00385T0 | 5 | 5 | NCU00385T0\_input.txt | NCU00385T0\_output.txt | 0.352273 AGGCCCAGAAGATTGTCAGC | CTGTGCACACTCAAAGCAGC | 0.353710 AGGCCCAGAAGATTGTCAGC | CGATGGCCGATCACTAGTGG | 0.635981 AGGCCCAGAAGATTGTCAGC | GATGGCCGATCACTAGTGGC | 0.924004 AAGATTGTCAGCGGTGGTGG | CTGTGCACACTCAAAGCAGC | 0.925441 AAGATTGTCAGCGGTGGTGG | CGATGGCCGATCACTAGTGG | | | | | |
| NCU00386T0 | 2 | 5 | NCU00386T0\_input.txt | NCU00386T0\_output.txt | 0.288304 ACCAGCAGTAACACCATCCG | TTACTGACCTGGCATTCCGG | 0.497375 ACCAGCAGTAACACCATCCG | CTACTTCTTGAGGGCCTGCG | 0.498550 ACCAGATCCGAAACCTGACG | TTACTGACCTGGCATTCCGG | 0.647502 TACCATGAAGCCACCCATGC | TTACTGACCTGGCATTCCGG | 0.715749 CCCATGCCTACGGAGAAACC | TTACTGACCTGGCATTCCGG | | | | | |
| NCU00387T0 | 2 | 5 | NCU00387T0\_input.txt | NCU00387T0\_output.txt | 0.420477 CATGTGGATCGTGCATTGGG | AGTACTTCTTCAACCCGGCG | 0.420477 CCATGTGGATCGTGCATTGG | AGTACTTCTTCAACCCGGCG | 0.645225 ATCGTGCATTGGGTTTTGGG | AGTACTTCTTCAACCCGGCG | 1.042761 CGTGCATTGGGTTTTGGGG | AGTACTTCTTCAACCCGGCG | 1.112978 TGTTTGGGTTGAGGGATCTGG | AGTACTTCTTCAACCCGGCG | | | | | |
| NCU00388T0 | 3 | 5 | NCU00388T0\_input.txt | NCU00388T0\_output.txt | 0.217181 GCCTGGGAGAAATTTGCTGC | TGCAAAGCCATGATTTCGGC | 0.217181 GCTGCCTGGGAGAAATTTGC | TGCAAAGCCATGATTTCGGC | 0.218195 GCCTGGGAGAAATTTGCTGC | TCCACTTTTCCAAGGGTCGG | 0.218195 GCTGCCTGGGAGAAATTTGC | TCCACTTTTCCAAGGGTCGG | 0.219532 CTACCGTGTGACGCAAAAGC | TGCAAAGCCATGATTTCGGC | | | | | |
| NCU00389T0 | 6 | 5 | NCU00389T0\_input.txt | NCU00389T0\_output.txt | 0.484692 CAAGTCCGTCAAGCATCAGC | TCAGTGTACGTGCGTTACCC | 0.542935 CAAGTCCGTCAAGCATCAGC | TATCGGTCAGTGTACGTGCG | 0.714150 AAGTCCGTCAAGCATCAGCC | TCAGTGTACGTGCGTTACCC | 0.772394 AAGTCCGTCAAGCATCAGCC | TATCGGTCAGTGTACGTGCG | 0.815708 CAAGTCCGTCAAGCATCAGC | CATGAAAGTCTGCGTACGGC | | | | | |
| NCU00391T0 | 2 | 5 | NCU00391T0\_input.txt | NCU00391T0\_output.txt | 0.640914 TCCATTGACTGTACAGCCCG | GCTACACCAACTCTCCAGGC | 0.643135 TGGGAAAGAAGCTGGGAAGC | GCTACACCAACTCTCCAGGC | 0.917105 GAAAGAAGCTGGGAAGCTGC | GCTACACCAACTCTCCAGGC | 0.946586 ATGATTTCAGGAGCCAGGGG | GCTACACCAACTCTCCAGGC | 1.057091 GCCCGAATGATTTCAGGAGC | GCTACACCAACTCTCCAGGC | | | | | |
| NCU00392T0 | 2 | 5 | NCU00392T0\_input.txt | NCU00392T0\_output.txt | 0.147105 TCCAGACAACATCACAGCCC | CGCCCTTGACTTTGTTACGC | 0.147105 TCCAGACAACATCACAGCCC | GCGCCCTTGACTTTGTTACG | 0.147677 AGACAACATCACAGCCCAGG | CGCCCTTGACTTTGTTACGC | 0.147677 AGACAACATCACAGCCCAGG | GCGCCCTTGACTTTGTTACG | 0.218988 CTATGGATCATCCGACGGCC | CGCCCTTGACTTTGTTACGC | | | | | |
| NCU00395T0 | 2 | 5 | NCU00395T0\_input.txt | NCU00395T0\_output.txt | 0.361123 GACTTTTTGGCAGCAGCTCC | TAGTAGGTGTTGGCAACCGG | 0.567919 GAGCAAAGCTTGGACAAGGC | GTACGGGCTGGATGTTACGG | 0.567919 GAGCAAAGCTTGGACAAGGC | GGTACGGGCTGGATGTTACG | 0.768466 CGAGCAAAGCTTGGACAAGG | GTACGGGCTGGATGTTACGG | 0.768466 CGAGCAAAGCTTGGACAAGG | GGTACGGGCTGGATGTTACG | | | | | |
| NCU00396T0 | 4 | 5 | NCU00396T0\_input.txt | NCU00396T0\_output.txt | 0.543884 ATGGTGACTTGTGCGAGAGG | ATGTCTGTGAAAACGACGCG | 0.615546 AACTTGATCGCTCGGTGAGG | ATGTCTGTGAAAACGACGCG | 0.650729 ATGGTGACTTGTGCGAGAGG | AGTTTAGAAGGCAGACCGGG | 0.707696 CTCGGTGAGGGAAATCGAGC | AAACCACTTACCACCACCGG | 0.831604 AATCGAGCGCAAGATTTCGG | AAACCACTTACCACCACCGG | | | | | |
| NCU00396T1 | 4 | 5 | NCU00396T1\_input.txt | NCU00396T1\_output.txt | 0.543884 ATGGTGACTTGTGCGAGAGG | ATGTCTGTGAAAACGACGCG | 0.615546 AACTTGATCGCTCGGTGAGG | ATGTCTGTGAAAACGACGCG | 0.650729 ATGGTGACTTGTGCGAGAGG | AGTTTAGAAGGCAGACCGGG | 0.707696 CTCGGTGAGGGAAATCGAGC | AAACCACTTACCACCACCGG | 0.831604 AATCGAGCGCAAGATTTCGG | AAACCACTTACCACCACCGG | | | | | |
| NCU00397T0 | 4 | 5 | NCU00397T0\_input.txt | NCU00397T0\_output.txt | 0.360732 GTTAACCGTGGTCTCTCCGG | AAGATCTTGGGGTTGTGGGC | 0.424590 CAATGCCCTGAAGCTCTTGC | AAGATCTTGGGGTTGTGGGC | 0.440223 GTTAACCGTGGTCTCTCCGG | TGGTTATTGACAGGCTGGGG | 0.504081 CAATGCCCTGAAGCTCTTGC | TGGTTATTGACAGGCTGGGG | 0.556746 GTTAACCGTGGTCTCTCCGG | CGTGCTGGTTATTGACAGGC | | | | | |
| NCU00398T1 | 2 | 5 | NCU00398T1\_input.txt | NCU00398T1\_output.txt | 0.428586 TTGTTGGAAAAACGCCAGGC | ACACTATTCGCCCTCGATGC | 0.550742 TTGTTGGAAAAACGCCAGGC | CAGAGCGCTTTCATCGAACC | 0.568251 AAGTCTGTCTGGAGTTCGCC | ACACTATTCGCCCTCGATGC | 0.690407 AAGTCTGTCTGGAGTTCGCC | CAGAGCGCTTTCATCGAACC | 0.780655 TTGTTGGAAAAACGCCAGGC | TATTCGCCCTCGATGCTACC | | | | | |
| NCU00398T0 | 1 | 5 | NCU00398T0\_input.txt | NCU00398T0\_output.txt | 0.215950 TCAGAGCAACACTAGCAGCC | ATCCTGATGCATGGGACTGC | 0.514156 TCAGAGCAACACTAGCAGCC | AGTCCATCCTGATGCATGGG | 0.636104 AACTCACCGTCAACTACCCG | TTGCTCTGACTGTCTTCCGC | 0.639282 CAACACTAGCAGCCCTACGG | ATCCTGATGCATGGGACTGC | 0.646657 ACTAGCAGCCCTACGGTAGG | ATCCTGATGCATGGGACTGC | | | | | |
| NCU00399T0 | 2 | 5 | NCU00399T0\_input.txt | NCU00399T0\_output.txt | 0.217072 ACCCGGCTCTCTTGTAAACG | TACGGATGCTAAGCCCAACC | 0.286976 ATTGGCCGTGATGACTGAGG | TACGGATGCTAAGCCCAACC | 0.631250 CAAGGGCTACGTTCATTGGC | TACGGATGCTAAGCCCAACC | 0.859428 AAGGGCTACGTTCATTGGCC | TACGGATGCTAAGCCCAACC | 1.106914 ATTGGCCGTGATGACTGAGG | CGCTGAGGGTTCAACATACG | | | | | |
| NCU00400T0 | 1 | 5 | NCU00400T0\_input.txt | NCU00400T0\_output.txt | 0.145506 CCAATTTCGACCACCAAGCG | TTCAGCAGATGACAGCCAGG | 0.214439 TTGCTCTTCTCGACATCCGG | GTGAATGGAATCGATGGCGC | 0.283445 TTGCTCTTCTCGACATCCGG | CTCTAGCAGCGTCCGATAGC | 0.345333 TGAGATAGCTGGTATCGCGC | AGCCCTTTTTCGTTGTTGCC | 0.356416 CCAATTTCGACCACCAAGCG | TCGAGGCAAGAACAGCTACC | | | | | |
| NCU00401T0 | 2 | 5 | NCU00401T0\_input.txt | NCU00401T0\_output.txt | 0.558171 CGGGACTGTTAACCGAGAGC | AGAACGAGAATACCGGACGC | 0.922268 AACCGAGAGCAAACCCAACC | AGAACGAGAATACCGGACGC | 1.290790 AAAGAGGGAGAAGAGAGGCG | AGAACGAGAATACCGGACGC | 1.328301 ACCGAGAGCAAACCCAACC | AGAACGAGAATACCGGACGC | 1.644017 TAACCGAGAGCAAACCCAACC | AGAACGAGAATACCGGACGC | | | | | |
| NCU00401T1 | 3 | 5 | NCU00401T1\_input.txt | NCU00401T1\_output.txt | 0.135323 TAGGGTTGTGCAACGGTACC | AGAACGAGAATACCGGACGC | 0.136911 TAGCTAGGGTTGTGCAACGG | AGAACGAGAATACCGGACGC | 0.208284 ACTGGCATGAAGTAGTCGCC | AGAACGAGAATACCGGACGC | 0.420046 TCAGCTCGAGGTTGAACAGC | AGAACGAGAATACCGGACGC | 0.555151 GTAGTCGCCACAGTAGGTCC | AGAACGAGAATACCGGACGC | | | | | |
| NCU00403T0 | 5 | 5 | NCU00403T0\_input.txt | NCU00403T0\_output.txt | 1.180377 GTGTCTGACAACCCAACATGC | GTCCAGTAGGCTCACCATGG | 1.496613 TGTCTGACAACCCAACATGC | GTCCAGTAGGCTCACCATGG | 1.616356 GTGTCTGACAACCCAACATGC | TCCTGTCCAGTAGGCTCACC | 1.932592 TGTCTGACAACCCAACATGC | TCCTGTCCAGTAGGCTCACC | 2.068236 GTGTCTGACAACCCAACATGC | TGCGGAAAGAGAACAGCTAGG | | | | | |
| NCU00403T1 | 5 | 5 | NCU00403T1\_input.txt | NCU00403T1\_output.txt | 1.180377 GTGTCTGACAACCCAACATGC | GTCCAGTAGGCTCACCATGG | 1.496613 TGTCTGACAACCCAACATGC | GTCCAGTAGGCTCACCATGG | 1.616356 GTGTCTGACAACCCAACATGC | TCCTGTCCAGTAGGCTCACC | 1.932592 TGTCTGACAACCCAACATGC | TCCTGTCCAGTAGGCTCACC | 2.068236 GTGTCTGACAACCCAACATGC | TGCGGAAAGAGAACAGCTAGG | | | | | |
| NCU00403T2 | 5 | 5 | NCU00403T2\_input.txt | NCU00403T2\_output.txt | 1.180377 GTGTCTGACAACCCAACATGC | GTCCAGTAGGCTCACCATGG | 1.496613 TGTCTGACAACCCAACATGC | GTCCAGTAGGCTCACCATGG | 1.616356 GTGTCTGACAACCCAACATGC | TCCTGTCCAGTAGGCTCACC | 1.932592 TGTCTGACAACCCAACATGC | TCCTGTCCAGTAGGCTCACC | 2.068236 GTGTCTGACAACCCAACATGC | TGCGGAAAGAGAACAGCTAGG | | | | | |
| NCU00404T0 | 4 | 5 | NCU00404T0\_input.txt | NCU00404T0\_output.txt | 0.148418 GGTTGTGCAATGTCACGACC | TCATCGGCAAGAGAAGCTGG | 0.218029 GGTTGTGCAATGTCACGACC | AAGAGAAGCTGGAGATGCCG | 0.356614 GTGTGCATGGATTTAGCGGC | TCATCGGCAAGAGAAGCTGG | 0.366689 GGTTGTGCAATGTCACGACC | TCTTGGCTTCCTCCTTGAGC | 0.426225 GTGTGCATGGATTTAGCGGC | AAGAGAAGCTGGAGATGCCG | | | | | |
| NCU00405T0 | 2 | 5 | NCU00405T0\_input.txt | NCU00405T0\_output.txt | 0.215829 CCTCGAGCACAACTACTGGG | TCGGCCTTGCTGGAAATAGG | 0.216206 TCAACATCCGCGAGTACACC | TCGGCCTTGCTGGAAATAGG | 0.358511 TGGTGATGGCAAGGTTGAGG | TCGGCCTTGCTGGAAATAGG | 0.494728 CCTCGAGCACAACTACTGGG | GCTGGAGATACCGACAGACC | 0.495105 TCAACATCCGCGAGTACACC | GCTGGAGATACCGACAGACC | | | | | |
| NCU00406T0 | 5 | 5 | NCU00406T0\_input.txt | NCU00406T0\_output.txt | 0.568321 TCTGCTTGGAGGTGAGTTGC | GTCAACTTGGCGCAGAAACC | 0.919785 TCTGCTTGGAGGTGAGTTGC | TAGCTTCGGTCAACTTGGCG | 1.193073 ACTTGTACAGACATGCCGCG | AAATATCGACCTTGGGGCCG | 1.331898 ACTTGTACAGACATGCCGCG | GTCAACTTGGCGCAGAAACC | 1.332806 ACTTGTACAGACATGCCGCG | ACTCCTTCTGCTTGACGACC | | | | | |
| NCU00407T0 | 1 | 5 | NCU00407T0\_input.txt | NCU00407T0\_output.txt | 0.213741 ATGTGAGGCAAGAAGGGAGC | GGACTCCAAGCCTCGATTCC | 0.353432 ATGTGAGGCAAGAAGGGAGC | CTATAGGCGGGACTCCAAGC | 0.361970 TGGAGTCCCGCCTATAGAGG | GTATTTCCTCCTCGTCCGCC | 0.365949 ATGTGAGGCAAGAAGGGAGC | TGTCCCCAACATCCATCACC | 0.425011 ATGTGAGGCAAGAAGGGAGC | TCCACACTCTCAGCAATCGC | | | | | |
| NCU00408T0 | 3 | 5 | NCU00408T0\_input.txt | NCU00408T0\_output.txt | 0.279587 TCTTCCGAATCCATCACGCC | ACAACAGAAAACACTGCCGC | 0.348718 CGAATCCATCACGCCAAAGC | ACAACAGAAAACACTGCCGC | 0.561490 GACACCCTTCTGCCTCATCG | ACAACAGAAAACACTGCCGC | 0.617380 TCACGCCAAAGCAATTCTCG | ACAACAGAAAACACTGCCGC | 0.991186 CCAATTGACACCCTTCTGCC | ACAACAGAAAACACTGCCGC | | | | | |
| NCU00409T1 | 4 | 5 | NCU00409T1\_input.txt | NCU00409T1\_output.txt | 0.285951 GGGTTGAGTTCTCGAAGGGG | CTACCTCTCCAATCGGCAGC | 0.567137 AGTTCTCGAAGGGGACAACG | CTACCTCTCCAATCGGCAGC | 0.571221 TGCCATGGGTTGAGTTCTCG | CTACCTCTCCAATCGGCAGC | 0.775418 AACGCCGAAATCCACATTGG | CTACCTCTCCAATCGGCAGC | 0.922546 GTTAGGGTAGGTAGAGGCGC | CTACCTCTCCAATCGGCAGC | | | | | |
| NCU00409T0 | 4 | 5 | NCU00409T0\_input.txt | NCU00409T0\_output.txt | 0.211704 GACGTCTAGGTAGGCTTCGC | TCGGCCATGGGTTGAATAGG | 0.218831 GGGTTGAGTTCTCGAAGGGG | TCGGCCATGGGTTGAATAGG | 0.285951 GGGTTGAGTTCTCGAAGGGG | CTACCTCTCCAATCGGCAGC | 0.499060 GACGTCTAGGTAGGCTTCGC | GGCCATGGGTTGAATAGGGG | 0.500016 AGTTCTCGAAGGGGACAACG | TCGGCCATGGGTTGAATAGG | | | | | |
| NCU00410T0 | 6 | 5 | NCU00410T0\_input.txt | NCU00410T0\_output.txt | 0.975428 CTTGATGTTCGAGGCAGTGC | CCTAGGACCGAATTCCACGC | 0.975428 CTTGATGTTCGAGGCAGTGC | GCCTAGGACCGAATTCCACG | 2.109095 GACACGCGACTTGATGTTCG | GCGCTCTCAAATAGTGCCC | 2.349030 TTGCTGGGTGGAGGTAATGG | GCGCTCTCAAATAGTGCCC | 2.463188 CTTGATGTTCGAGGCAGTGC | GCGCTCTCAAATAGTGCCC | | | | | |
| NCU00411T0 | 3 | 5 | NCU00411T0\_input.txt | NCU00411T0\_output.txt | 0.290626 GATTACGGCGAGGGATCAGG | ACAGAAGGAGAGGGACCAGG | 0.501587 TGCAGATACGTGGTGGAAGG | ACAGAAGGAGAGGGACCAGG | 0.630724 GCAATGCAGATACGTGGTGG | ACAGAAGGAGAGGGACCAGG | 0.642671 GATTACGGCGAGGGATCAGG | CCAGTTGTTGACCCCGTACC | 0.643070 TGGTGTTGATTACGGCGAGG | ACAGAAGGAGAGGGACCAGG | | | | | |
| NCU00412T0 | 4 | 5 | NCU00412T0\_input.txt | NCU00412T0\_output.txt | 0.768779 CTCCAGCAAGAGGGTTCTGG | GGTGAAGCAATTAGACCGGC | 0.918492 AATAGCTTCTTCGAGGGCGC | TCGTTACCCTGAAGCGAACG | 0.983943 TGGCATACCCGTTGTACTGG | GGTGAAGCAATTAGACCGGC | 1.008613 CTCCAGCAAGAGGGTTCTGG | TCGAAGGGGAGCTTCAGAGG | 1.042462 CTCCAGCAAGAGGGTTCTGG | CGGGTGAAGCAATTAGACCG | | | | | |
| NCU00413T0 | 4 | 5 | NCU00413T0\_input.txt | NCU00413T0\_output.txt | 2.160118 GCACAAGTTCGCTGTCAAGC | ACCTGGAGATGGTAGAAGCC | 2.276483 GCACAAGTTCGCTGTCAAGC | ATGAGACCCGCCTTCTGACC | 2.313496 TCTACAGAGGCTTCTCGTGC | ACCTGGAGATGGTAGAAGCC | 2.429862 TCTACAGAGGCTTCTCGTGC | ATGAGACCCGCCTTCTGACC | 2.479611 CCAAGACTCGTGGTGTTGCC | CAATCCCAGAAACCCCAGCC | | | | | |
| NCU00414T0 | 3 | 5 | NCU00414T0\_input.txt | NCU00414T0\_output.txt | 0.060683 CGCTAAGCTCTCCATCCAGG | TGCGCTGAACAACAAGAACG | 0.136435 CGCTAAGCTCTCCATCCAGG | ACGCCTCTTCTCCTATTGCG | 0.283566 CGCTAAGCTCTCCATCCAGG | ACAACCAACGCCTCTTCTCC | 0.339870 CGAGGGCAAGTCTATCGAGG | TGCGCTGAACAACAAGAACG | 0.344558 CGTCGAGGGCAAGTCTATCG | TGCGCTGAACAACAAGAACG | | | | | |
| NCU00415T0 | 4 | 5 | NCU00415T0\_input.txt | NCU00415T0\_output.txt | 0.838042 TTCGGTAGGCATTGGTGACC | TAGCAGATTGACAGACGCGC | 1.187611 GGCATTTCGGTAGGCATTGG | TAGCAGATTGACAGACGCGC | 1.542958 GTAGGCATTGGTGACCATGC | TAGCAGATTGACAGACGCGC | 1.664833 TTCGGTAGGCATTGGTGACC | TTAGCAGATTGACAGACGCG | 1.850214 TTCGGTAGGCATTGGTGACC | TGCCTCTGAATGTGTCAATGC | | | | | |
| NCU00416T0 | 2 | 5 | NCU00416T0\_input.txt | NCU00416T0\_output.txt | 0.342292 TCTACTTGGGCTGCACATCG | CAATGAGATGCATCCGTCGC | 0.356400 TCTACTTGGGCTGCACATCG | GATGCATCCGTCGCTTTTGG | 0.549041 CAACAGCATCCACACCAACC | CAATGAGATGCATCCGTCGC | 0.563150 CAACAGCATCCACACCAACC | GATGCATCCGTCGCTTTTGG | 0.777504 TCTACTTGGGCTGCACATCG | AATACCGTGCAGTGTCACCG | | | | | |
| NCU00417T0 | 2 | 5 | NCU00417T0\_input.txt | NCU00417T0\_output.txt | 1.073658 CCTCAATGTCGGCAAAACCC | TGGTTTGCCCAAGCCTTACC | 1.273892 CATTTTGAGGTCGTCGCTGG | TGGTTTGCCCAAGCCTTACC | 1.273892 CTGGCATTTTGAGGTCGTCG | TGGTTTGCCCAAGCCTTACC | 1.302867 TCGCAATTGTTCGGTTGAAGG | ACGCTTACTGTTGAGGGAGC | 1.439154 ATGTCGGCAAAACCCTCTGG | TGGTTTGCCCAAGCCTTACC | | | | | |
| NCU00418T0 | 5 | 5 | NCU00418T0\_input.txt | NCU00418T0\_output.txt | 0.413035 AACCATGAACTTTTGGCGCC | GGATGTTCATCTGGCTTGCG | 0.415585 GTGGAGCCAAGAGACTTCCC | GGATGTTCATCTGGCTTGCG | 0.503697 TATCGATGGCTTCCTGCAGG | CATGGGTAACGGCGTTTTGG | 0.631304 CCAAGAGACTTCCCTCCAGC | GGATGTTCATCTGGCTTGCG | 0.688972 CTCTCCACGCGTCTATCAGG | GGATGTTCATCTGGCTTGCG | | | | | |
| NCU00419T0 | 5 | 5 | NCU00419T0\_input.txt | NCU00419T0\_output.txt | 0.142459 AATTCAAGCCCCAAAACGCC | TTACCTGCAGGAAGCCATCG | 0.355439 ACAAGAGGCTTAGGCAGACG | TTACCTGCAGGAAGCCATCG | 0.672470 CTTATCCGAAACGACGTCGC | TTACCTGCAGGAAGCCATCG | 0.838877 CAAAACGCCGTTACCCATGC | TTACCTGCAGGAAGCCATCG | 1.177681 ACCAATTCAAGCCCCAAAACG | TTACCTGCAGGAAGCCATCG | | | | | |
| NCU00420T1 | 3 | 5 | NCU00420T1\_input.txt | NCU00420T1\_output.txt | 0.356659 ACAAATCCTCTCCACCCAGC | GTGGTTGTTGTTGGACGTCG | 0.772908 CAAATCCTCTCCACCCAGCG | GTGGTTGTTGTTGGACGTCG | 0.834560 GCTGCACACAAATCCTCTCC | GTGGTTGTTGTTGGACGTCG | 1.195777 GCACACAAATCCTCTCCACC | GTGGTTGTTGTTGGACGTCG | 1.209874 ACAAATCCTCTCCACCCAGC | CAAGTCCACTGGTGTCACGG | | | | | |
| NCU00420T0 | 3 | 5 | NCU00420T0\_input.txt | NCU00420T0\_output.txt | 0.356659 ACAAATCCTCTCCACCCAGC | GTGGTTGTTGTTGGACGTCG | 0.772908 CAAATCCTCTCCACCCAGCG | GTGGTTGTTGTTGGACGTCG | 0.834560 GCTGCACACAAATCCTCTCC | GTGGTTGTTGTTGGACGTCG | 1.195777 GCACACAAATCCTCTCCACC | GTGGTTGTTGTTGGACGTCG | 1.209874 ACAAATCCTCTCCACCCAGC | CAAGTCCACTGGTGTCACGG | | | | | |
| NCU00421T0 | 2 | 5 | NCU00421T0\_input.txt | NCU00421T0\_output.txt | 0.480848 CGTTCCGGAAGCATTGATCG | CTGACTTGACGCTACTCGGG | 0.480848 CGTTCCGGAAGCATTGATCG | CCTGACTTGACGCTACTCGG | 0.759712 CGTTCCGGAAGCATTGATCG | GACGCTACTCGGGTTCAAGG | 0.810328 CGTTCCGGAAGCATTGATCG | CACTCAACAAACCGAGCTCG | 0.896484 CGTTCCGGAAGCATTGATCG | CAAGGCGCACTCAACAAACC | | | | | |
| NCU00422T0 | 4 | 5 | NCU00422T0\_input.txt | NCU00422T0\_output.txt | 0.429894 GGTGACAAGGACAACTCCCC | AACTTGGAGAGATCGAGCGC | 0.562873 GGTGACAAGGACAACTCCCC | GTTCTTGCTGCCAGTGTTCC | 0.651594 TGGTGACAAGGACAACTCCC | AACTTGGAGAGATCGAGCGC | 0.712731 GGTGACAAGGACAACTCCCC | CTCACCCTCCTTCTTAGCGG | 0.781762 TGGTGTCATTGAGTGCGTCC | AACTTGGAGAGATCGAGCGC | | | | | |
| NCU00422T1 | 4 | 5 | NCU00422T1\_input.txt | NCU00422T1\_output.txt | 0.429894 GGTGACAAGGACAACTCCCC | AACTTGGAGAGATCGAGCGC | 0.562873 GGTGACAAGGACAACTCCCC | GTTCTTGCTGCCAGTGTTCC | 0.651594 TGGTGACAAGGACAACTCCC | AACTTGGAGAGATCGAGCGC | 0.712731 GGTGACAAGGACAACTCCCC | CTCACCCTCCTTCTTAGCGG | 0.781762 TGGTGTCATTGAGTGCGTCC | AACTTGGAGAGATCGAGCGC | | | | | |
| NCU00423T0 | 6 | 5 | NCU00423T0\_input.txt | NCU00423T0\_output.txt | 0.350415 GCACAAGCGGCGTTAATACC | CAGCATGCTCCTGATTTGGC | 0.354252 GCACAAGCGGCGTTAATACC | AATTGATCCTGGGAGGCAGC | 0.413335 AGTTTACAACCTTGCTGCGC | CAGCATGCTCCTGATTTGGC | 0.507872 GCACAAGCGGCGTTAATACC | ACCAATTGATCCTGGGAGGC | 0.843022 GTCCGTGAAAATCGCAAGGG | AAGGTGATCAGAGTTGCCGC | | | | | |
| NCU00424T0 | 3 | 5 | NCU00424T0\_input.txt | NCU00424T0\_output.txt | 0.608886 CTTCGCTGCCTTCAACATCG | CTTTTGTCTGCTTCCGTCGG | 1.014553 CTTCGCTGCCTTCAACATCG | CGGTGTATGTAACGGAAGCG | 1.043602 TGATCTCGGCAAAGGATCGC | CTTTTGTCTGCTTCCGTCGG | 1.048958 GAAGAGGGCGGGAATGATGG | CTTTTGTCTGCTTCCGTCGG | 1.449269 TGATCTCGGCAAAGGATCGC | CGGTGTATGTAACGGAAGCG | | | | | |
| NCU00425T0 | 2 | 5 | NCU00425T0\_input.txt | NCU00425T0\_output.txt | 0.699553 CAACATGATGCTGATGGCCG | TGAACACCATCGACTTCGCC | 0.746343 CAACATGATGCTGATGGCCG | CTGAACACCATCGACTTCGC | 0.834003 CAACATGATGCTGATGGCCG | CTTCGCCAGTGGTCTGATCG | 0.848364 ACGCTCAGATGGGTTATGGC | TGAACACCATCGACTTCGCC | 0.848366 TCAGATGGGTTATGGCAGCG | TGAACACCATCGACTTCGCC | | | | | |
| NCU00426T0 | 2 | 5 | NCU00426T0\_input.txt | NCU00426T0\_output.txt | 0.266130 CAAGGGAGTTTGGTGCTTGC | TTGGACGCACGAATGAAACG | 0.412689 AGGCGTCAGTCATCATTCGG | TTGGACGCACGAATGAAACG | 0.444229 GTTCTGCTGAGGTTGTTGCG | GAGGATGGGGATGATGTGGG | 0.444229 GTTCTGCTGAGGTTGTTGCG | GGAGGATGGGGATGATGTGG | 0.477496 TTGACTGCTAGAGCGTGACC | TTGGACGCACGAATGAAACG | | | | | |
| NCU00427T0 | 2 | 5 | NCU00427T0\_input.txt | NCU00427T0\_output.txt | 0.288125 TGGTGCCAAGTGGAAGAAGG | CTTGGAAGCGAAACATGGGC | 0.479483 TGGTGCCAAGTGGAAGAAGG | CGTTGGTGTGTTGTCTGACG | 0.545485 CACTGGAGTGGAAGGAGACG | CGTTGGTGTGTTGTCTGACG | 0.552709 TGGTGCCAAGTGGAAGAAGG | GTGCATTTACTCGGAAGCGG | 0.565106 TGGTGCCAAGTGGAAGAAGG | TTTCTTTTTCCGGGCGTTGG | | | | | |
| NCU00428T0 | 2 | 5 | NCU00428T0\_input.txt | NCU00428T0\_output.txt | 0.634174 GACAGGACCACCACTTGTCG | TCAGCAGGCACATATAGCGG | 0.703238 TGGCGTAAAGTCTTGGCTCG | TCAGCAGGCACATATAGCGG | 0.839072 GACAGGACCACCACTTGTCG | GTAGATCTAGGCTCGCGTGG | 0.848265 TGGCGTAAAGTCTTGGCTCG | ATCTCCTCTCCGTCATCCCC | 0.908136 TGGCGTAAAGTCTTGGCTCG | GTAGATCTAGGCTCGCGTGG | | | | | |
| NCU00430T0 | 2 | 5 | NCU00430T0\_input.txt | NCU00430T0\_output.txt | 0.351317 GGAGGCTCTCAAGGAGAAGC | TGAGAACCTCGCCATCTTCG | 0.408438 TGTTCTGGCTGTGGAATCCC | CCTCGCCATCTTCGTTTTCG | 0.424692 CTAGGAGAAGTGAACCGGCC | CGTTTGGTTGGTTTGGACGG | 0.548109 GGAGGCTCTCAAGGAGAAGC | CCTCGCCATCTTCGTTTTCG | 0.569167 TCAGGCTGGAGCTATGATGC | TGAGAACCTCGCCATCTTCG | | | | | |
| NCU00431T0 | 2 | 5 | NCU00431T0\_input.txt | NCU00431T0\_output.txt | 0.287570 GATTCCATACCTGTCGGGCC | GTCGGTGAAGGTCTCTTGGG | 0.287570 GATTCCATACCTGTCGGGCC | GGTCGGTGAAGGTCTCTTGG | 0.495257 GGTTCAGCTTACCGAGGTCG | GTCGGTGAAGGTCTCTTGGG | 0.495257 GGTTCAGCTTACCGAGGTCG | GGTCGGTGAAGGTCTCTTGG | 0.637487 CGATTCCATACCTGTCGGGC | GTCGGTGAAGGTCTCTTGGG | | | | | |
| NCU00432T1 | 5 | 5 | NCU00432T1\_input.txt | NCU00432T1\_output.txt | 1.587986 TTGAGACTGGTACACTGGCG | GCACTCTTTCTTTCCCTTGCC | 2.273637 TTGAGACTGGTACACTGGCG | TGCACTCTTTCTTTCCCTTGC | 2.337684 ATTGAGACTGGTACACTGGCG | GCACTCTTTCTTTCCCTTGCC | 2.877265 TTGAGACTGGTACACTGGCG | ATGCACTCTTTCTTTCCCTTGC | 3.023335 ATTGAGACTGGTACACTGGCG | TGCACTCTTTCTTTCCCTTGC | | | | | |
| NCU00432T0 | 5 | 5 | NCU00432T0\_input.txt | NCU00432T0\_output.txt | 1.588764 CACCGGACTGCGTATCTAGC | GCACTCTTTCTTTCCCTTGCC | 2.274415 CACCGGACTGCGTATCTAGC | TGCACTCTTTCTTTCCCTTGC | 2.878043 CACCGGACTGCGTATCTAGC | ATGCACTCTTTCTTTCCCTTGC | 3.031834 CACCGGACTGCGTATCTAGC | GCACTCTTTCTTTCCCTTGC | 3.053857 ACCGGACTGCGTATCTAGCG | GCACTCTTTCTTTCCCTTGCC | | | | | |
| NCU00434T0 | 2 | 5 | NCU00434T0\_input.txt | NCU00434T0\_output.txt | 0.865021 TCCATCATGTTCCAGTCCCG | TTGGCCTCTCAACTAACCGG | 1.001852 TCCATCATGTTCCAGTCCCG | GCCTCTCAACTAACCGGAGC | 1.222418 TTCAGGTCCAGTCCGAGTCC | TTGGCCTCTCAACTAACCGG | 1.356517 GATGCTTCAGGTCCAGTCCG | TCTCAACTAACCGGAGCTGG | 1.359248 TTCAGGTCCAGTCCGAGTCC | GCCTCTCAACTAACCGGAGC | | | | | |
| NCU00435T0 | 4 | 5 | NCU00435T0\_input.txt | NCU00435T0\_output.txt | 0.064739 AGGGTAGGTTCATTGTGCGG | CATCCCGCTTTGTTATGCCG | 0.420829 AGGGTAGGTTCATTGTGCGG | CCATCCCGCTTTGTTATGCC | 0.420829 AGGGTAGGTTCATTGTGCGG | CCCATCCCGCTTTGTTATGC | 0.556865 ATTGTGCGGTTCATGCTTCC | CATCCCGCTTTGTTATGCCG | 0.912955 ATTGTGCGGTTCATGCTTCC | CCATCCCGCTTTGTTATGCC | | | | | |
| NCU00436T0 | 2 | 5 | NCU00436T0\_input.txt | NCU00436T0\_output.txt | 0.778750 ATTCCTGGTTTGATTGCGGC | TCTACAGAACGCTGAAGGCC | 0.847727 TGGTACAAGTGTGCTCAGCG | CTTCTCCTTCAGACGGTGGG | 0.850801 CAAAAGGAGGAAAAGGCCGG | CTTCTCCTTCAGACGGTGGG | 0.919865 ATTCCTGGTTTGATTGCGGC | AGAGTTTCGTTACGGCGTCC | 0.989968 ATTCCTGGTTTGATTGCGGC | TGAACGTAGTCGATGGCAGC | | | | | |
| NCU00437T0 | 1 | 5 | NCU00437T0\_input.txt | NCU00437T0\_output.txt | 0.065534 CAAGGGTTTGTGAAGCGTGG | TCCTTCAAGTCAACCTCCGC | 0.068311 TACTGGGGAGAAGGAGGAGC | ACCCTTTACGCCTCTTCAGC | 0.074963 GAGCAAGGGTTTGTGAAGCG | TCCTTCAAGTCAACCTCCGC | 0.209853 TACTGGGGAGAAGGAGGAGC | GCCTCTTCAGCTTCCTCTCC | 0.212283 GAAGGAGGAGCTGGTTACGG | ACCCTTTACGCCTCTTCAGC | | | | | |
| NCU00438T0 | 5 | 5 | NCU00438T0\_input.txt | NCU00438T0\_output.txt | 0.069449 CGTCTCCTCAACCTTCCACC | AGAGAAGGAGAGGCTCAGGG | 0.211217 CAAGACGATGGAAGGGGAGG | AGAGAAGGAGAGGCTCAGGG | 0.640939 CGTCTCCTCAACCTTCCACC | TTGCTCGCTTGTGAGAAGGG | 0.647465 CGTCTCCTCAACCTTCCACC | AGGGCTTTGATGTGGTCTGC | 0.717877 AACCGTCTCCTCAACCTTCC | AGAGAAGGAGAGGCTCAGGG | | | | | |
| NCU00439T0 | 3 | 5 | NCU00439T0\_input.txt | NCU00439T0\_output.txt | 0.135312 CATTGCTACCGTTGCACTCG | GAGCACCGCAAAAGTTCAGG | 0.191699 CATTGCTACCGTTGCACTCG | GCGCATAGAAACAACGGACC | 0.192221 CATTGCTACCGTTGCACTCG | CCTGCGCATAGAAACAACGG | 0.202459 CATTGCTACCGTTGCACTCG | ATGCCATGTCCGTAACCAGG | 0.432261 ATCTGAAGTGGAAGCTGGCG | GAGCACCGCAAAAGTTCAGG | | | | | |
| NCU00440T0 | 4 | 5 | NCU00440T0\_input.txt | NCU00440T0\_output.txt | 0.210347 GTTTGTGCACAGGCTCTTGG | GACTTCGTCCTGTCTACGCC | 0.404895 GTTTGTGCACAGGCTCTTGG | GTCTACGCCAAATCGCAAGG | 0.490763 GTTTGTGCACAGGCTCTTGG | TCCTGTCTACGCCAAATCGC | 0.493667 GGTCTGGGATCTTACTCGCG | AAGCACTCGATAGGCTTGGC | 0.493937 GTTTGTGCACAGGCTCTTGG | AAGCACTCGATAGGCTTGGC | | | | | |
| NCU00441T0 | 3 | 5 | NCU00441T0\_input.txt | NCU00441T0\_output.txt | 0.146036 GAAAGTGGGACCAGAGACGG | TTGTCGAACTCCATCTCGGC | 0.147546 GGATAGCATGGACGAGGACG | TCGATTGCTGCTGAGACAGG | 0.209871 CATGGACGAGGACGAGAAGG | TCGATTGCTGCTGAGACAGG | 0.287447 AGATGGAGGGAGAGTAGGCG | TCGATTGCTGCTGAGACAGG | 0.356960 GAAAGTGGGACCAGAGACGG | AATCATCTCGATCAGCCCCG | | | | | |
| NCU00442T0 | 3 | 5 | NCU00442T0\_input.txt | NCU00442T0\_output.txt | 0.072034 CATCCTCCATCCTCATCCGC | AAAGAGGAACGCTGGAGTGG | 0.072034 GCATCCTCCATCCTCATCCG | AAAGAGGAACGCTGGAGTGG | 0.277790 CATCCTCCATCCTCATCCGC | GGCTTTGGAAAGAGGAACGC | 0.277790 GCATCCTCCATCCTCATCCG | GGCTTTGGAAAGAGGAACGC | 0.278594 CATCCTCCATCCTCATCCGC | CTTGCGGCTTTGGAAAGAGG | | | | | |
| NCU00443T1 | 4 | 5 | NCU00443T1\_input.txt | NCU00443T1\_output.txt | 0.430785 ACCACGAGAATGGCAAGACC | GGCATCCTTGAAAGCGTTGG | 0.495180 TCAAGTTCGTCAAGGAGGCC | CTCACCCTCGGAAACATCGG | 0.639507 ACCACGAGAATGGCAAGACC | GTTGTCGGAGTTGGCAAAGC | 0.776610 ACCACGAGAATGGCAAGACC | CATCCTTGAAAGCGTTGGCG | 0.781937 ACCACGAGAATGGCAAGACC | CTCACCCTCGGAAACATCGG | | | | | |
| NCU00443T0 | 3 | 5 | NCU00443T0\_input.txt | NCU00443T0\_output.txt | 0.430785 ACCACGAGAATGGCAAGACC | GGCATCCTTGAAAGCGTTGG | 0.495180 TCAAGTTCGTCAAGGAGGCC | CTCACCCTCGGAAACATCGG | 0.639507 ACCACGAGAATGGCAAGACC | GTTGTCGGAGTTGGCAAAGC | 0.776610 ACCACGAGAATGGCAAGACC | CATCCTTGAAAGCGTTGGCG | 0.781937 ACCACGAGAATGGCAAGACC | CTCACCCTCGGAAACATCGG | | | | | |
| NCU00444T0 | 5 | 5 | NCU00444T0\_input.txt | NCU00444T0\_output.txt | 0.578373 CTATAGCGGGCGAGTTCTCG | ACTGGGTATGGTATGGTGCG | 0.856875 GGACATGTCTATAGCGGGCG | ACTGGGTATGGTATGGTGCG | 0.923756 CTATAGCGGGCGAGTTCTCG | TATGGTATGGTGCGCACTGC | 0.946547 CTATAGCGGGCGAGTTCTCG | AGAGGGTGGTGGTGTACTGG | 0.994851 CTATAGCGGGCGAGTTCTCG | CTGGGTATGGTATGGTGCGC | | | | | |
| NCU00445T0 | 3 | 5 | NCU00445T0\_input.txt | NCU00445T0\_output.txt | 0.684597 ACGCAACCGCTTTATTCACG | TTGTTTGTAGGATTGCCGCG | 0.698693 CAACCGCTTTATTCACGCCC | TTGTTTGTAGGATTGCCGCG | 0.914878 GTCGCCCAATCCAAATCACC | GGGTAAGTTAGACGTGGCGG | 0.954527 CTCGAAACTTTGACGAGCCG | TTGTTTGTAGGATTGCCGCG | 0.975334 GTCGCCCAATCCAAATCACC | TTGTTTGTAGGATTGCCGCG | | | | | |
| NCU00446T0 | 2 | 5 | NCU00446T0\_input.txt | NCU00446T0\_output.txt | 0.357148 GCAGTCTGCTGGTTCAATGC | AGAGACACTGGAATGACCGC | 0.497970 GTTACCGTTACAGGGGGTCC | AGAGACACTGGAATGACCGC | 0.691417 GCAGTCTGCTGGTTCAATGC | GGCGGATAACGTTCTCATGC | 0.772598 CAGTCTGCTGGTTCAATGCC | AGAGACACTGGAATGACCGC | 0.842789 CACAGACCATAGTAGCGCCG | AGAGACACTGGAATGACCGC | | | | | |
| NCU00447T0 | 2 | 5 | NCU00447T0\_input.txt | NCU00447T0\_output.txt | 0.214813 CTCTTGGAGGCTGCTGTACC | GGGTTGGTAATCCTTCGGGG | 0.287079 CTCTTGGAGGCTGCTGTACC | CTTTCAGGACGCGTAGGAGG | 0.341753 CAGTTTTCAACCTCGTCGCC | GGGTTGGTAATCCTTCGGGG | 0.341753 CCAGTTTTCAACCTCGTCGC | GGGTTGGTAATCCTTCGGGG | 0.414019 CAGTTTTCAACCTCGTCGCC | CTTTCAGGACGCGTAGGAGG | | | | | |
| NCU00448T0 | 3 | 5 | NCU00448T0\_input.txt | NCU00448T0\_output.txt | 0.867283 CTGGAGGAAGCTCGATGACG | TTCGAGGAATCTTGGGTGGG | 0.939756 TTGGCCCAGAGTAAGTTCGC | TTCGAGGAATCTTGGGTGGG | 1.138933 TTGGCCCAGAGTAAGTTCGC | TGTTGTTGGGGACAGCTTCG | 1.290239 AAGTGGTGAGACGCATGAGC | TTCGAGGAATCTTGGGTGGG | 1.387152 TTGGCCCAGAGTAAGTTCGC | ACAGCTTCGAGGAATCTTGGG | | | | | |
| NCU00449T0 | 2 | 5 | NCU00449T0\_input.txt | NCU00449T0\_output.txt | 0.275528 GTGAAGTCATTGAAGCGGCC | ATGGGAATGGAAAGAGCGGG | 0.275528 GGTGAAGTCATTGAAGCGGC | ATGGGAATGGAAAGAGCGGG | 0.344998 GTGAAGTCATTGAAGCGGCC | GAGCGGGAGTGAGGTTTAGG | 0.344998 GGTGAAGTCATTGAAGCGGC | GAGCGGGAGTGAGGTTTAGG | 0.496760 CCACTTGTTGAGGTCCGAGG | GAGCGGGAGTGAGGTTTAGG | | | | | |
| NCU00450T1 | 5 | 5 | NCU00450T1\_input.txt | NCU00450T1\_output.txt | 0.923445 AGGCGTTAGTGATCGATGGC | GGAACGTTGCTTCCTCTCCC | 0.924251 ATGGCGTCGATGTATTGGGG | GGAACGTTGCTTCCTCTCCC | 1.169244 GCACAGTCAACGATGAAGGC | TCCTGCTGATCAATATCGCCC | 1.239145 GCACAGTCAACGATGAAGGC | CCGGGGGAAGATCATTACAGG | 1.240779 GCACAGTCAACGATGAAGGC | CTGCTGATCAATATCGCCCG | | | | | |
| NCU00450T0 | 4 | 5 | NCU00450T0\_input.txt | NCU00450T0\_output.txt | 0.194400 GCACAGTCAACGATGAAGGC | GGTTATCATCACGCAACGGC | 0.467571 CACAGTCAACGATGAAGGCG | GGTTATCATCACGCAACGGC | 0.467571 CGCACAGTCAACGATGAAGG | GGTTATCATCACGCAACGGC | 0.632520 GCACAGTCAACGATGAAGGC | TGACCCGGATCTGATCAAGC | 0.905691 CACAGTCAACGATGAAGGCG | TGACCCGGATCTGATCAAGC | | | | | |
| NCU00451T0 | 1 | 5 | NCU00451T0\_input.txt | NCU00451T0\_output.txt | 0.355395 AAGTGCATGGAGAAGGCTGG | TGGCCCAGATTCCCAATTCC | 0.364007 ATCAAGGACATCCAGGTGCC | GATTTCCTCAGGGGTCCAGC | 0.366590 ATCAAGGACATCCAGGTGCC | TCAGCTCTCTCTTGAACGCG | 0.369843 AGGAATTGGGAATCTGGGCC | GATTTCCTCAGGGGTCCAGC | 0.372427 AGGAATTGGGAATCTGGGCC | TCAGCTCTCTCTTGAACGCG | | | | | |
| NCU00453T0 | 5 | 5 | NCU00453T0\_input.txt | NCU00453T0\_output.txt | 0.145129 GGAGTTTTTCGGCAGCTTGG | TTGGTGGTCAGTTGAGTCCG | 0.218354 GGAGTTTTTCGGCAGCTTGG | GTGCTTAAGTGTGATGCCGC | 0.579290 ATCCCAAGATGGAGAGCTGC | TTGGTGGTCAGTTGAGTCCG | 0.652515 ATCCCAAGATGGAGAGCTGC | GTGCTTAAGTGTGATGCCGC | 0.693732 GTTGTTGGCATTTTGGCTGC | TTGGTGGTCAGTTGAGTCCG | | | | | |
| NCU00454T0 | 1 | 5 | NCU00454T0\_input.txt | NCU00454T0\_output.txt | 0.149732 GGGTATAGCCAGGGTTCTGC | GCAATATGCTGCAGTACGCC | 0.209169 TGGAATGGCGTGGTGATACC | TCCGCTGATAAGTACGGTGC | 0.216184 TGGAATGGCGTGGTGATACC | GCAGAACCCTGGCTATACCC | 0.278208 GGCCAGGACCATTTCATTGC | TCCGCTGATAAGTACGGTGC | 0.280888 GCCAGGACCATTTCATTGCG | TCCGCTGATAAGTACGGTGC | | | | | |
| NCU00455T0 | 3 | 5 | NCU00455T0\_input.txt | NCU00455T0\_output.txt | 0.357450 GGGCATTAACAGTGTTGGCC | CGTCTCTCAAACGCAACTGC | 0.361603 AACGAGATTGTGGGAGAGGC | CGTCTCTCAAACGCAACTGC | 0.631583 GGGCATTAACAGTGTTGGCC | CTCAAACGCAACTGCTCAGC | 0.633428 TGACTTCCGCTAACTCCACG | TAATGTCGTTGAGGGTGGCG | 0.635736 AACGAGATTGTGGGAGAGGC | CTCAAACGCAACTGCTCAGC | | | | | |
| NCU00456T0 | 3 | 5 | NCU00456T0\_input.txt | NCU00456T0\_output.txt | 1.213869 ATGCTGGCGTAGAAGTTGGG | TTTGAGTTGTTGGGTGGTGC | 1.300205 TAGAAGTTGGGGAGGAGGGC | TGCGCTATAACTAGCCTGGG | 1.441571 TAGAAGTTGGGGAGGAGGGC | TTTGAGTTGTTGGGTGGTGC | 1.477287 ATGCTGGCGTAGAAGTTGGG | GTGCAGGTGACGGAAATACC | 1.531264 ATGCTGGCGTAGAAGTTGGG | ACGGAAATACCTGATGGGACG | | | | | |
| NCU00457T0 | 6 | 5 | NCU00457T0\_input.txt | NCU00457T0\_output.txt | 0.996962 GAGATGAGCGGTGAAAGGGG | TGTTTCTCGGGACAACTGCC | 1.160301 GACATTCACGACATCGCAGC | ACCGTCGAGTCCATCAAATCC | 1.312857 TCCTTGAACTCGGCATAGGC | ACCGTCGAGTCCATCAAATCC | 1.341870 CGAGATGAGCGGTGAAAGGG | TGTTTCTCGGGACAACTGCC | 1.341870 CCGAGATGAGCGGTGAAAGG | TGTTTCTCGGGACAACTGCC | | | | | |
| NCU00458T1 | 5 | 5 | NCU00458T1\_input.txt | NCU00458T1\_output.txt | 0.635174 CCGATTCTTCCCCTTCGAGC | CCCGGTAGGATGTTTCTCGG | 0.641960 CCGATTCTTCCCCTTCGAGC | CAGTGAAAAGACGACTGCGC | 0.641960 CCGATTCTTCCCCTTCGAGC | GCAGTGAAAAGACGACTGCG | 0.773622 TAACTCTCTGGCAAACGGCG | CCCGGTAGGATGTTTCTCGG | 0.780408 TAACTCTCTGGCAAACGGCG | CAGTGAAAAGACGACTGCGC | | | | | |
| NCU00458T0 | 4 | 5 | NCU00458T0\_input.txt | NCU00458T0\_output.txt | 0.635174 CCGATTCTTCCCCTTCGAGC | CCCGGTAGGATGTTTCTCGG | 0.641960 CCGATTCTTCCCCTTCGAGC | CAGTGAAAAGACGACTGCGC | 0.641960 CCGATTCTTCCCCTTCGAGC | GCAGTGAAAAGACGACTGCG | 0.773622 TAACTCTCTGGCAAACGGCG | CCCGGTAGGATGTTTCTCGG | 0.780408 TAACTCTCTGGCAAACGGCG | CAGTGAAAAGACGACTGCGC | | | | | |
| NCU00459T0 | 2 | 5 | NCU00459T0\_input.txt | NCU00459T0\_output.txt | 0.193852 GAGTAACGTACCTCAGCCGG | GAGTGTCGAGCATGAAACGC | 0.202177 GCCCTTCAAATCAAGCAGCC | GAGTGTCGAGCATGAAACGC | 0.268935 GAGTAACGTACCTCAGCCGG | AAGCATGCCATGTTCGAACG | 0.279302 GAGTAACGTACCTCAGCCGG | GGTCAAGCATGCCATGTTCG | 0.344782 CTATCCCCCATTCAGCCAGC | GAGTGTCGAGCATGAAACGC | | | | | |
| NCU00459T1 | 2 | 5 | NCU00459T1\_input.txt | NCU00459T1\_output.txt | 0.202177 GCCCTTCAAATCAAGCAGCC | GAGTGTCGAGCATGAAACGC | 0.277260 GCCCTTCAAATCAAGCAGCC | AAGCATGCCATGTTCGAACG | 0.287628 GCCCTTCAAATCAAGCAGCC | GGTCAAGCATGCCATGTTCG | 0.344782 CTATCCCCCATTCAGCCAGC | GAGTGTCGAGCATGAAACGC | 0.347032 CCCCTTCAAGGAGTACTGCC | GAGTGTCGAGCATGAAACGC | | | | | |
| NCU00461T0 | 4 | 5 | NCU00461T0\_input.txt | NCU00461T0\_output.txt | 0.195352 AAGTAGACGTCGTCGATGCC | CCGACATCAAACACGCATCC | 0.200913 CAGAGTCTCGGGAATCCAGC | CCGACATCAAACACGCATCC | 0.206665 GACCGTCAACCAGAGTCTCG | CCGACATCAAACACGCATCC | 0.487025 AAGTAGACGTCGTCGATGCC | CCTTCACTCTTGGTCGACCG | 0.487467 AGAAAGCAGCAACTTTGGCC | CCGACATCAAACACGCATCC | | | | | |
| NCU00462T0 | 6 | 5 | NCU00462T0\_input.txt | NCU00462T0\_output.txt | 0.142653 TGATCGGGATGTTTCTGGCC | AAGGCCCACAATCAGGTACG | 0.277195 GATTGTGGGCCTTTTCTCGC | TTGTCAGGTGGGAGTCAACG | 0.279368 GATTGTGGGCCTTTTCTCGC | CAGGTGGGAGTCAACGATCC | 0.357509 TGATCGGGATGTTTCTGGCC | TTGGACTCTTGCGTTCTGGG | 0.415266 TGTCACCAACGAATTTGCCG | TTGTCAGGTGGGAGTCAACG | | | | | |
| NCU00463T0 | 3 | 5 | NCU00463T0\_input.txt | NCU00463T0\_output.txt | 0.490641 AAGCCTCTTGAAGAGTCGCC | GAATTTGGGTTTCGACCGCG | 0.490641 AAGCCTCTTGAAGAGTCGCC | CGAATTTGGGTTTCGACCGC | 0.830985 GTCTGCTTTTTATCGCCCCG | CGAATTTGGGTTTCGACCGC | 0.906589 AAGCCTCTTGAAGAGTCGCC | ACGAATTTGGGTTTCGACCG | 1.106319 GTCTGCTTTTTATCGCCCCG | CGGTGCTGATGATGTTGTCC | | | | | |
| NCU00464T0 | 5 | 5 | NCU00464T0\_input.txt | NCU00464T0\_output.txt | 0.216318 TCTTGTTGGAGCCATAGCCG | AAGACGTTGGCCTCTCATCG | 0.279176 AACGTACTCAGCAGCGTAGG | AAGACGTTGGCCTCTCATCG | 0.353242 TCTTGTTGGAGCCATAGCCG | ACATACAGACAACGGACGGG | 0.357937 TCTTGTTGGAGCCATAGCCG | TGATGAGGAAGGCCAAGACG | 0.420795 AACGTACTCAGCAGCGTAGG | TGATGAGGAAGGCCAAGACG | | | | | |
| NCU00465T0 | 4 | 5 | NCU00465T0\_input.txt | NCU00465T0\_output.txt | 0.484444 CCGGATGGTGAATCAATGCG | TGAGCGACCATTAGTGAGGC | 0.625391 GCCTTGAACTTGACTTCGGC | GTATTGCCCATCAAGCAGGC | 0.695523 CCGGATGGTGAATCAATGCG | GTATTGCCCATCAAGCAGGC | 0.847149 AAGAATCTCGTACGCCTGGG | GTATTGCCCATCAAGCAGGC | 0.975189 GCCTTGAACTTGACTTCGGC | TACCCGGCGATATGATAGCG | | | | | |
| NCU00465T1 | 5 | 5 | NCU00465T1\_input.txt | NCU00465T1\_output.txt | 0.484444 CCGGATGGTGAATCAATGCG | TGAGCGACCATTAGTGAGGC | 0.625391 GCCTTGAACTTGACTTCGGC | GTATTGCCCATCAAGCAGGC | 0.695523 CCGGATGGTGAATCAATGCG | GTATTGCCCATCAAGCAGGC | 0.847149 AAGAATCTCGTACGCCTGGG | GTATTGCCCATCAAGCAGGC | 0.975189 GCCTTGAACTTGACTTCGGC | TACCCGGCGATATGATAGCG | | | | | |
| NCU00466T0 | 5 | 5 | NCU00466T0\_input.txt | NCU00466T0\_output.txt | 0.073422 AATATCGTCGATGACCGGGC | TCTTTGTCAACGAGGAGGCC | 0.148308 AATATCGTCGATGACCGGGC | CCGACGTGAGCAAATTTGGG | 0.197162 CGGAAGCGTGATGGTTTTCC | AGGCGTTGTATAGCTCGACG | 0.203024 CGGAAGCGTGATGGTTTTCC | TCTTTGTCAACGAGGAGGCC | 0.276457 CGGAAGCGTGATGGTTTTCC | ATAGCTCGACGTGTTTGGGG | | | | | |
| NCU00467T0 | 2 | 5 | NCU00467T0\_input.txt | NCU00467T0\_output.txt | 0.564354 GAAAGCTTGATTGCGGCAGG | CAACTAGGGACTGGCCATGG | 0.778798 AAGCTGCAAGTACGCTGAGG | CAACTAGGGACTGGCCATGG | 0.848998 GAAAGCTTGATTGCGGCAGG | ACAACAGCTACAGCCATCCG | 0.994990 GAAAGCTTGATTGCGGCAGG | GAGACAACTAGGGACTGGCC | 1.058238 AAAGTAGCAGTGTCCGTCGC | ACAACAGCTACAGCCATCCG | | | | | |
| NCU00468T1 | 4 | 5 | NCU00468T1\_input.txt | NCU00468T1\_output.txt | 1.064788 ACACACATGGACCGTACTGG | AACGGGCATCTTGATGGACG | 1.135544 CACACATGGACCGTACTGGG | AACGGGCATCTTGATGGACG | 1.390042 ACACACATGGACCGTACTGG | GGGTATAACACGGCTACCTGG | 1.460798 CACACATGGACCGTACTGGG | GGGTATAACACGGCTACCTGG | 1.473743 ACACACATGGACCGTACTGG | ACGGGCATCTTGATGGACG | | | | | |
| NCU00468T0 | 4 | 5 | NCU00468T0\_input.txt | NCU00468T0\_output.txt | 1.064788 ACACACATGGACCGTACTGG | AACGGGCATCTTGATGGACG | 1.135544 CACACATGGACCGTACTGGG | AACGGGCATCTTGATGGACG | 1.390042 ACACACATGGACCGTACTGG | GGGTATAACACGGCTACCTGG | 1.460798 CACACATGGACCGTACTGGG | GGGTATAACACGGCTACCTGG | 1.473743 ACACACATGGACCGTACTGG | ACGGGCATCTTGATGGACG | | | | | |
| NCU00469T0 | 1 | 5 | NCU00469T0\_input.txt | NCU00469T0\_output.txt | 0.572729 ACCCCTTTAAGCACTGCTCG | AACCCAGCAGCTTTCTGAGG | 0.646595 ACCCCTTTAAGCACTGCTCG | AACATCCACTCCAGAACCCG | 0.646663 TGGGTGGCAATACAGATGCC | AACCCAGCAGCTTTCTGAGG | 0.646927 TGGGTGGCAATACAGATGCC | AGCAGTGCTTAAAGGGGTGG | 0.701253 CTGTCTGGCTCATTACCGGG | ACAACGCTAGAGTCGAGTGG | | | | | |
| NCU00470T1 | 7 | 5 | NCU00470T1\_input.txt | NCU00470T1\_output.txt | 0.358183 CTGTCTGGCTCATTACCGGG | TTAATGGACTGAGCGAGGCG | 0.696427 CGGTCTTGCCAGTAAACTGC | TTAATGGACTGAGCGAGGCG | 0.703487 CTGTCTGGCTCATTACCGGG | GTTTTCCCGGAAATGTCCCG | 0.717168 TCAGAAAGCTGCTGGGTTGG | TTAATGGACTGAGCGAGGCG | 0.821601 CTGCTAATTCGCAAGGACGG | GTCTTCTTTGTGGCCGATGC | | | | | |
| NCU00470T0 | 5 | 5 | NCU00470T0\_input.txt | NCU00470T0\_output.txt | 0.289619 CATGAGTTGTCGCAGTGTGC | TTAATGGACTGAGCGAGGCG | 0.347238 GGTGGCTTTGATTCGAACGG | TTAATGGACTGAGCGAGGCG | 0.486112 CGGTAGAGAATGAGACGCGG | GTCTTCTTTGTGGCCGATGC | 0.496214 CGGTAGAGAATGAGACGCGG | TTAATGGACTGAGCGAGGCG | 0.546563 ACGGTAGAGAATGAGACGCG | GTCTTCTTTGTGGCCGATGC | | | | | |
| NCU00471T0 | 4 | 5 | NCU00471T0\_input.txt | NCU00471T0\_output.txt | 0.357354 TGCTGACCTGATGAACGTGG | AGCCTTTAGTACGTGTGGGC | 0.428531 TGCTGACCTGATGAACGTGG | ACGTGTGGGCCACTTATAGC | 0.428676 TGCTGACCTGATGAACGTGG | TTCAGAGAGCCAGAATGCCG | 0.480540 CAAGAATCTGAACCACCGCG | AGCCTTTAGTACGTGTGGGC | 0.551717 CAAGAATCTGAACCACCGCG | ACGTGTGGGCCACTTATAGC | | | | | |
| NCU00472T0 | 4 | 5 | NCU00472T0\_input.txt | NCU00472T0\_output.txt | 0.272314 TGATCGCCTTGGTTCTGTCC | GAGGTTGTGAACTTGCGAGC | 0.288348 CCGTAAGCAACACAATGGCC | AACACCCTCGAAACACCAGG | 0.345245 CCGTAAGCAACACAATGGCC | GAGGTTGTGAACTTGCGAGC | 0.353408 TGATCGCCTTGGTTCTGTCC | GTTGTGAACTTGCGAGCACC | 0.426339 CCGTAAGCAACACAATGGCC | GTTGTGAACTTGCGAGCACC | | | | | |
| NCU00473T0 | 1 | 5 | NCU00473T0\_input.txt | NCU00473T0\_output.txt | 0.060756 GAAGAAGCTCATGCGCATGG | CCGGCTCTCGTGTAATACCC | 0.136402 TCGGTTGAATGGGATCACGG | AATCAGCGCGACATTCATGC | 0.140144 ATGAATGTCGCGCTGATTGC | TACCTTCACCCCAAAGCACC | 0.279019 ATGAATGTCGCGCTGATTGC | CAGGCCAGTCTACCTTCACC | 0.346638 GAAGAAGCTCATGCGCATGG | CGGCTCTCGTGTAATACCCG | | | | | |
| NCU00474T0 | 2 | 5 | NCU00474T0\_input.txt | NCU00474T0\_output.txt | 0.346328 GTCTGGTCCGAATGTCTCCC | GTGGCTACGATTTTGGTGGC | 0.349473 CGTCTGGTCCGAATGTCTCC | GTGGCTACGATTTTGGTGGC | 0.696100 TAATGCGCAAGAAACCTGCC | GTGGCTACGATTTTGGTGGC | 0.787885 GTCTGGTCCGAATGTCTCCC | AAGCCTTCCAAATCGGGTGG | 0.791030 CGTCTGGTCCGAATGTCTCC | AAGCCTTCCAAATCGGGTGG | | | | | |
| NCU00475T0 | 4 | 5 | NCU00475T0\_input.txt | NCU00475T0\_output.txt | 0.142801 TCACCATCATGCAGAACCCC | AAGATCGTCACGGAGCTTGG | 0.212171 CACCTCTGAGGAGCTTGAGC | GGATCTGAGAGTCCTTGCCG | 0.213902 TTTTATGCAGCGTTGACGGC | GGATCTGAGAGTCCTTGCCG | 0.216848 CACCTCTGAGGAGCTTGAGC | AAGATCGTCACGGAGCTTGG | 0.287687 CACTGCCTACAAGATCCCCG | AAGATCGTCACGGAGCTTGG | | | | | |
| NCU00476T0 | 1 | 5 | NCU00476T0\_input.txt | NCU00476T0\_output.txt | 0.622202 TCTTGTAGCAGGCAACCTCG | GACAACTACACAACCACCGC | 0.637054 TCTTGTAGCAGGCAACCTCG | CACAACCACCGCCAAAATGG | 0.851146 TCTTGTAGCAGGCAACCTCG | GAAAAGCCAGTCCAGCATCC | 0.853747 TCTTGTAGCAGGCAACCTCG | ACAACTACACAACCACCGCC | 0.893966 TCTTGTAGCAGGCAACCTCG | CGACAACTACACAACCACCG | | | | | |
| NCU00477T0 | 2 | 5 | NCU00477T0\_input.txt | NCU00477T0\_output.txt | 0.410543 GCTCGACAACTTCAACCTGC | CCGAGCTCAAGGAAGAGACC | 0.483917 AAGGTCAAGCAGTACAGCGG | GTTCCAAGCGTACTCGTTGC | 0.495387 AAGGTCAAGCAGTACAGCGG | CCGAGCTCAAGGAAGAGACC | 0.558382 GCTCGACAACTTCAACCTGC | TGTCAATGGAAGAAGGGCCG | 0.643227 AAGGTCAAGCAGTACAGCGG | TGTCAATGGAAGAAGGGCCG | | | | | |
| NCU00478T0 | 2 | 5 | NCU00478T0\_input.txt | NCU00478T0\_output.txt | 1.063499 GTTTCATCCACCGAACCTGC | TTGGCTTGGTTTGGCTAGGG | 1.337351 GTTTCATCCACCGAACCTGC | GGTTTGGCTAGGGCTCATCG | 1.388519 ACCGAACACTGGTAGAAGCC | CTAGCTGCTTAGACTTCCCGG | 1.432810 ACCGAACACTGGTAGAAGCC | TAGCTGCTTAGACTTCCCGG | 1.490141 GTTTCATCCACCGAACCTGC | TGCATGGTTCCTTCTTTGGC | | | | | |
| NCU00478T1 | 3 | 5 | NCU00478T1\_input.txt | NCU00478T1\_output.txt | 0.720861 CGAGGAGACTGCTAAAGGGC | TTGGCTTGGTTTGGCTAGGG | 0.720861 GCGAGGAGACTGCTAAAGGG | TTGGCTTGGTTTGGCTAGGG | 0.720861 GGCGAGGAGACTGCTAAAGG | TTGGCTTGGTTTGGCTAGGG | 0.720997 GAGGAGACTGCTAAAGGGCC | TTGGCTTGGTTTGGCTAGGG | 0.785670 TTTCGAGACGCTCAAGGAGG | TTGGCTTGGTTTGGCTAGGG | | | | | |
| NCU00480T0 | 5 | 5 | NCU00480T0\_input.txt | NCU00480T0\_output.txt | 0.205754 GCCTTCTTGCCATAAGTGCG | ATTGGAAGATCAGGCTGGCC | 0.423838 GTTTTTGCCTCGTGGAACGG | ATTGGAAGATCAGGCTGGCC | 0.485281 TGTTTTTGCCTCGTGGAACG | ATTGGAAGATCAGGCTGGCC | 0.638953 GCCTTCTTGCCATAAGTGCG | GAGGTCCTGGAAAGAGAGGC | 0.645369 AATCTTGCTCAGGGACCTCG | ATTGGAAGATCAGGCTGGCC | | | | | |
| NCU00481T0 | 4 | 5 | NCU00481T0\_input.txt | NCU00481T0\_output.txt | 0.695653 TGGAAGCCTGAGTCTTCTGC | CAGATGATGACTGTGCACGC | 0.972237 GGGGTCCCGTTTGTTTAAGC | CAGATGATGACTGTGCACGC | 1.003351 TTGTGGATCCATTCCTGGGG | CAGATGATGACTGTGCACGC | 1.366188 TGGATCCATTCCTGGGGTCC | CAGATGATGACTGTGCACGC | 1.390065 GCTCGTTGTGGATCCATTCC | CAGATGATGACTGTGCACGC | | | | | |
| NCU00483T0 | 2 | 5 | NCU00483T0\_input.txt | NCU00483T0\_output.txt | 3.008486 CGCACGTCCCAAAAGAATGG | ACATCTCAATTGCTCTCGTCC | 3.076773 TAATGCGAGGAGGAGTTGGC | ACATCTCAATTGCTCTCGTCC | 3.145578 CGTTGTGATGATGTCGCACG | ACATCTCAATTGCTCTCGTCC | 3.196525 GAAGATTTAGCGCGTGGTCG | ACATCTCAATTGCTCTCGTCC | 3.268978 GGTCGTTGTGATGATGTCGC | ACATCTCAATTGCTCTCGTCC | | | | | |
| NCU00484T0 | 3 | 5 | NCU00484T0\_input.txt | NCU00484T0\_output.txt | 0.142741 ATGACAGCCTCAACCATGGG | GGGACTCGAGGTGTTTACGG | 0.500738 GGCCGCCATAGAGCTTATCC | GGGACTCGAGGTGTTTACGG | 0.920359 ATGACAGCCTCAACCATGGG | AGGGACTCGAGGTGTTTACG | 0.929836 ACAAGCAGCCTGTCAAGTGG | GGGACTCGAGGTGTTTACGG | 0.999487 TGTCAAGTGGGTGCATGAGC | GGGACTCGAGGTGTTTACGG | | | | | |
| NCU00485T0 | 3 | 5 | NCU00485T0\_input.txt | NCU00485T0\_output.txt | 0.413016 TTCTTATCCAGCACAGCCGG | TTTGCAACCTAACGACGTGC | 0.789912 TTCTTATCCAGCACAGCCGG | ACATTGATGGGAGCAGCACC | 1.177203 TTCTTATCCAGCACAGCCGG | ATTTGCAACCTAACGACGTGC | 1.315142 GTTCTTATCCAGCACAGCCG | TTTGCAACCTAACGACGTGC | 1.351722 TTCTTATCCAGCACAGCCGG | ACGTGCCCTCCATTGAAAGC | | | | | |
| NCU00486T0 | 3 | 5 | NCU00486T0\_input.txt | NCU00486T0\_output.txt | 0.214739 CATTACCACCCCTGGAGTCG | CGACCCCTAACTTCCACACC | 0.218273 CATTACCACCCCTGGAGTCG | AACCCTTCCAGCATCCTTGG | 0.359758 AAAGACGCCATTACCACCCC | CGACCCCTAACTTCCACACC | 0.363292 AAAGACGCCATTACCACCCC | AACCCTTCCAGCATCCTTGG | 0.572420 CATTACCACCCCTGGAGTCG | AATCAGAACATGGCGACCCC | | | | | |
| NCU00487T0 | 2 | 5 | NCU00487T0\_input.txt | NCU00487T0\_output.txt | 0.479706 CAATGTCCGCCGATAACACG | GCACAATCTTGTTCTCGGCG | 0.566780 AAACCACCAACAATGTCCGC | GCACAATCTTGTTCTCGGCG | 0.784288 GAGTCCAAGCTTTTGTCGCG | AGGCGTGGTTGGAGATTTCG | 0.784288 CGAGTCCAAGCTTTTGTCGC | AGGCGTGGTTGGAGATTTCG | 0.850456 GAGTCCAAGCTTTTGTCGCG | ATTTCGCGTGTGTAGAGGGC | | | | | |
| NCU00488T0 | 7 | 5 | NCU00488T0\_input.txt | NCU00488T0\_output.txt | 0.490618 CTGGGAACTCGTCCTTGAGC | GCTCCAGTAACTACGACCCG | 0.628830 GAGAAAGGAGGTCGATGCCG | GCTCCAGTAACTACGACCCG | 0.637334 TGAGAAAGGAGGTCGATGCC | GCTCCAGTAACTACGACCCG | 0.710199 CTGGGAACTCGTCCTTGAGC | CCCATCAGAGGATAGCGTGG | 0.781331 CTGGGAACTCGTCCTTGAGC | CAGAGGATAGCGTGGAAGGG | | | | | |
| NCU00489T0 | 2 | 5 | NCU00489T0\_input.txt | NCU00489T0\_output.txt | 0.285717 AACACCGCGAGAAGGTATCC | GACGAACTTTCTGCGCTTGG | 0.500401 GCGAGAAGGTATCCCACTCC | GACGAACTTTCTGCGCTTGG | 0.775435 GCCCTCAGGACCTTCATAGC | AAGAACTCGTTCAGCTCGGC | 0.842865 AACACCGCGAGAAGGTATCC | AAGAACTCGTTCAGCTCGGC | 0.900893 GCCCTCAGGACCTTCATAGC | GCTGGAAGAACTCGTTCAGC | | | | | |
| NCU00490T0 | 8 | 5 | NCU00490T0\_input.txt | NCU00490T0\_output.txt | 0.421292 GATCATGAACCCGGCAAAGC | TTGGTTCCAAGTACGAGCGG | 0.712933 GATCATGAACCCGGCAAAGC | TTTCCGAGTCCTAACTGCCC | 0.713071 AAAGCCACATACCAGAGCCG | TTGGTTCCAAGTACGAGCGG | 0.773463 GCAAAGCCACATACCAGAGC | TTGGTTCCAAGTACGAGCGG | 0.930159 GACCGATATCGAACCCACGG | TTTCCGAGTCCTAACTGCCC | | | | | |
| NCU00490T1 | 8 | 5 | NCU00490T1\_input.txt | NCU00490T1\_output.txt | 0.421292 GATCATGAACCCGGCAAAGC | TTGGTTCCAAGTACGAGCGG | 0.712933 GATCATGAACCCGGCAAAGC | TTTCCGAGTCCTAACTGCCC | 0.713071 AAAGCCACATACCAGAGCCG | TTGGTTCCAAGTACGAGCGG | 0.773463 GCAAAGCCACATACCAGAGC | TTGGTTCCAAGTACGAGCGG | 0.930159 GACCGATATCGAACCCACGG | TTTCCGAGTCCTAACTGCCC | | | | | |
| NCU00491T0 | 2 | 5 | NCU00491T0\_input.txt | NCU00491T0\_output.txt | 0.205846 CCCGTACCTGTTAGTCAGCC | TCGTCTGATACAGCCATCGC | 0.211805 CCCGTACCTGTTAGTCAGCC | TCCCAAGTGACTGTGATGGC | 0.360369 AGCCAGTCAAGAACCCATCC | TCGTCTGATACAGCCATCGC | 0.366328 AGCCAGTCAAGAACCCATCC | TCCCAAGTGACTGTGATGGC | 0.420590 TCACAAGAGGCGTGGATTCG | TCGTCTGATACAGCCATCGC | | | | | |
| NCU00492T0 | 3 | 5 | NCU00492T0\_input.txt | NCU00492T0\_output.txt | 0.288574 CACCTTCTTCGGTCTGGTCC | ATGAACCTGGACACAAGGCC | 0.359766 CTCCTGAGCACCTTCTTCGG | ATGAACCTGGACACAAGGCC | 0.570638 ATATTCGTCCGCGTTAGGGC | ATGAACCTGGACACAAGGCC | 0.641813 GAGCACCTTCTTCGGTCTGG | ATGAACCTGGACACAAGGCC | 0.705962 CGTCGTCAGTGTACTGCTCC | ATGAACCTGGACACAAGGCC | | | | | |
| NCU00493T0 | 4 | 5 | NCU00493T0\_input.txt | NCU00493T0\_output.txt | 0.491367 TCTTCCCGATGCAAGTACGG | ATGATGATCGAGACGCCTGG | 0.979834 TCTTCCCGATGCAAGTACGG | CAATGAGGAACTTGTCGGCC | 1.123773 GATAGGCACCGTAAGCGAGG | CAATGAGGAACTTGTCGGCC | 1.182704 GCCTCAGGTGTCTAGCTTCG | TCTTCACCTTCATCCACCAGC | 1.316667 GAACGAGTTCTTCCCGATGC | ATGATGATCGAGACGCCTGG | | | | | |
| NCU00494T0 | 7 | 5 | NCU00494T0\_input.txt | NCU00494T0\_output.txt | 0.341180 GGCATCGTCGAGCATTTAGC | GGTTGGGTGACTGATACCCG | 0.546379 GTCTTTGCTCGTGCTAAGCC | TGGTTTTGCTCGCTTGTTGG | 0.693658 GTCTTTGCTCGTGCTAAGCC | ACATACGGCACTTCTGGACC | 0.913393 GGCATCGTCGAGCATTTAGC | GGGTGACTGATACCCGATCC | 1.060110 GTCTTTGCTCGTGCTAAGCC | AACAGACCCTAGCAGGAAGC | | | | | |
| NCU00495T0 | 2 | 5 | NCU00495T0\_input.txt | NCU00495T0\_output.txt | 1.171701 CGGTATATGGAGCGGATCGG | CGATGTCGGTGGAGTTGACG | 1.250064 ATACGGTCATGAGCAAGGCC | CGATGTCGGTGGAGTTGACG | 1.524409 GTAAGCCAATCTGGTGCAGC | CGATGTCGGTGGAGTTGACG | 1.531329 GTATATGGAGCGGATCGGGG | CGATGTCGGTGGAGTTGACG | 1.531329 GGTATATGGAGCGGATCGGG | CGATGTCGGTGGAGTTGACG | | | | | |
| NCU00496T0 | 3 | 5 | NCU00496T0\_input.txt | NCU00496T0\_output.txt | 0.215515 CACATCCCCTCCTTACGTGG | AGAAGGAGTTGAAGGGCAGC | 0.286493 CACATCCCCTCCTTACGTGG | CAGAGACAAAGGGAGCGAGG | 0.286493 CACCACATCCCCTCCTTACG | CAGAGACAAAGGGAGCGAGG | 0.421704 CACATCCCCTCCTTACGTGG | CAAAGATTGGACAACCGGGC | 0.421704 CACCACATCCCCTCCTTACG | CAAAGATTGGACAACCGGGC | | | | | |
| NCU00497T1 | 5 | 5 | NCU00497T1\_input.txt | NCU00497T1\_output.txt | 0.352660 AAGGTATGCATGATGGGGCC | GACGTAGAGTGGCCAAGAGG | 0.495271 TGACAGGTCGAAGGCAATGG | GACGTAGAGTGGCCAAGAGG | 0.635174 AAGGTATGCATGATGGGGCC | TCTCATTCCGAGGAGAACGC | 0.763903 CAACCTGACAGCTGTTCAGC | GACGTAGAGTGGCCAAGAGG | 1.046706 AAGGTATGCATGATGGGGCC | CTCATTCCGAGGAGAACGCG | | | | | |
| NCU00497T0 | 3 | 5 | NCU00497T0\_input.txt | NCU00497T0\_output.txt | 0.427436 TGAAAAGAGAGGAACGGGCC | TGAATCGCCCCTTGAACTCG | 0.428456 TGACAACGTGGATGAGCTCC | TGAATCGCCCCTTGAACTCG | 0.499338 GGATGAGCTCCCATGACTGG | TGAATCGCCCCTTGAACTCG | 0.544947 TGAAAAGAGAGGAACGGGCC | CGTTCCAGCAACAGAAGACG | 0.545967 TGACAACGTGGATGAGCTCC | CGTTCCAGCAACAGAAGACG | | | | | |
| NCU00497T2 | 4 | 5 | NCU00497T2\_input.txt | NCU00497T2\_output.txt | 0.428456 TGACAACGTGGATGAGCTCC | TGAATCGCCCCTTGAACTCG | 0.499338 GGATGAGCTCCCATGACTGG | TGAATCGCCCCTTGAACTCG | 0.544947 TGAAAAGAGAGGAACGGGCC | CGTTCCAGCAACAGAAGACG | 0.545967 TGACAACGTGGATGAGCTCC | CGTTCCAGCAACAGAAGACG | 0.550996 TGAAAAGAGAGGAACGGGCC | CCAGCAACAGAAGACGAAGC | | | | | |
| NCU00498T0 | 3 | 5 | NCU00498T0\_input.txt | NCU00498T0\_output.txt | 0.502341 AAGAAGGAGAAGCATGCCGG | ACTGGTATGACTGCGAACGG | 0.724915 TCCCAACTCAGAGGAGCTCC | ACTGGTATGACTGCGAACGG | 0.936814 AAGAAGGAGAAGCATGCCGG | TCTGCTGAGGGTATTGCTGG | 0.983862 GTTCGAGGTTTTGAGGCAGG | ACTGGTATGACTGCGAACGG | 0.997990 TTCGAGGTTTTGAGGCAGGC | ACTGGTATGACTGCGAACGG | | | | | |
| NCU00499T0 | 3 | 5 | NCU00499T0\_input.txt | NCU00499T0\_output.txt | 0.135588 ATCAGCTGATTGCCCAGTCC | CCTCGTAGCTCTGCTTCTCG | 0.429635 ATCAGCTGATTGCCCAGTCC | TCCATGTCTCTTTGCAGGGC | 0.640310 ATCAGCTGATTGCCCAGTCC | TTTGCAGGGCATTCATCTGC | 0.643733 ATCAGCTGATTGCCCAGTCC | GGGCATTCATCTGCTCCTCC | 0.698055 ATCAGCTGATTGCCCAGTCC | CTCGTAGCTCTGCTTCTCGC | | | | | |
| NCU00500T0 | 5 | 5 | NCU00500T0\_input.txt | NCU00500T0\_output.txt | 0.708700 AATTGTCAGAACTCCGCGGG | CCTACCTGCCTGTCCTTTGG | 0.774148 AATTGTCAGAACTCCGCGGG | GCGTTGCATACCCATGAACC | 0.916851 GCTTGACTTACCAACCTGGC | CCTACCTGCCTGTCCTTTGG | 0.924158 AATTGTCAGAACTCCGCGGG | ACAAGCCGACTCCAAGATCC | 0.982299 GCTTGACTTACCAACCTGGC | GCGTTGCATACCCATGAACC | | | | | |
| NCU00501T0 | 3 | 5 | NCU00501T0\_input.txt | NCU00501T0\_output.txt | 0.345763 CATTACCACCCCAACGTTGC | ATGAACTCGATGACCGGAGC | 0.634349 CATTACCACCCCAACGTTGC | CCGGAGCCTTCTTCACTTCC | 0.666879 AGAGGACCCTATGGAGACCC | ATGAACTCGATGACCGGAGC | 0.788305 TGACCCTTTCCTTCCTGACG | ATGAACTCGATGACCGGAGC | 0.796256 ACAGGCTTACAACCTGGAGG | ATGAACTCGATGACCGGAGC | | | | | |
| NCU00502T0 | 4 | 5 | NCU00502T0\_input.txt | NCU00502T0\_output.txt | 0.203391 AGCTCCTTCTGAACCTTGGC | CACAAGAACGCGGAACATCC | 0.216669 AGCTCCTTCTGAACCTTGGC | GAAGCGTTGTAAGCTTCCGC | 0.274983 GAGTCGAGGACAGTCTTGGC | CACAAGAACGCGGAACATCC | 0.288261 GAGTCGAGGACAGTCTTGGC | GAAGCGTTGTAAGCTTCCGC | 0.558405 TTGGCGATAACAGTCTGGGC | CACAAGAACGCGGAACATCC | | | | | |
| NCU00503T0 | 6 | 5 | NCU00503T0\_input.txt | NCU00503T0\_output.txt | 0.803864 ATGCCAGATCCATCCAGACC | TGACGAGGAAGGAGAGGAGG | 0.861180 TGTTAGTATACGCCCAGGCC | TGTGCAGACTGAGGTGTTCC | 1.198732 TGTTAGTATACGCCCAGGCC | GCTACAAAAGCGTGAGGAGC | 1.214133 ATGCCAGATCCATCCAGACC | GCTACAAAAGCGTGAGGAGC | 1.244187 TGTGTTAGTATACGCCCAGGC | TGTGCAGACTGAGGTGTTCC | | | | | |
| NCU00504T0 | 1 | 5 | NCU00504T0\_input.txt | NCU00504T0\_output.txt | 0.424456 CTAGAATCAGAGGCGCTGGG | AAAACCAATCACCCAACCGC | 0.427580 CTAGAATCAGAGGCGCTGGG | CACCTCCCATGTGCCTTACC | 0.498687 AAGCGGGATGAGAGAAAGGC | AAAACCAATCACCCAACCGC | 0.501811 AAGCGGGATGAGAGAAAGGC | CACCTCCCATGTGCCTTACC | 0.734953 AAGGGGAGAAGGGACTAGGG | AAAACCAATCACCCAACCGC | | | | | |
| NCU00505T0 | 3 | 5 | NCU00505T0\_input.txt | NCU00505T0\_output.txt | 0.986125 CGTCAAGATGACCCCCAAGG | CTTGTTCTTGGGCTTCACGG | 0.986125 CGTCAAGATGACCCCCAAGG | CCTTGTTCTTGGGCTTCACG | 1.182350 AACGGCACAATTGTACACGG | CTTGTTCTTGGGCTTCACGG | 1.182350 AACGGCACAATTGTACACGG | CCTTGTTCTTGGGCTTCACG | 1.262446 TCGCAACGTCAAGATGACCC | CTTGTTCTTGGGCTTCACGG | | | | | |
| NCU00505T1 | 3 | 5 | NCU00505T1\_input.txt | NCU00505T1\_output.txt | 0.986125 CGTCAAGATGACCCCCAAGG | CTTGTTCTTGGGCTTCACGG | 0.986125 CGTCAAGATGACCCCCAAGG | CCTTGTTCTTGGGCTTCACG | 1.182350 AACGGCACAATTGTACACGG | CTTGTTCTTGGGCTTCACGG | 1.182350 AACGGCACAATTGTACACGG | CCTTGTTCTTGGGCTTCACG | 1.262446 TCGCAACGTCAAGATGACCC | CTTGTTCTTGGGCTTCACGG | | | | | |
| NCU00506T0 | 2 | 5 | NCU00506T0\_input.txt | NCU00506T0\_output.txt | 1.018683 AACAAGATCACCCTCTGCCG | GTCGATTCGTGTTCAGGACG | 1.042835 AACAAGATCACCCTCTGCCG | TTCGTGTTCAGGACGATGCG | 1.090376 TCCTGTGGCATCGATTGACC | GTCGATTCGTGTTCAGGACG | 1.114528 TCCTGTGGCATCGATTGACC | TTCGTGTTCAGGACGATGCG | 1.161931 GACAATTTCGTGCGTCGTCC | GTCGATTCGTGTTCAGGACG | | | | | |
| NCU00509T0 | 4 | 5 | NCU00509T0\_input.txt | NCU00509T0\_output.txt | 0.429419 GCTTGCCCTCTTACTACGGG | ACTTCTCCCTTTGGTGACGC | 0.500572 TATGGCTATCCTGCATCGGC | ACTTCTCCCTTTGGTGACGC | 0.500733 GCTTGCCCTCTTACTACGGG | ACGCAGGAGGTTGCTAAAGG | 0.502992 AACTCACACCACCCGTTACC | GGGCTACCTCCCATTGTTGG | 0.567740 GCTTGCCCTCTTACTACGGG | TGGCAGTTTGGACATGTTGC | | | | | |
| NCU00510T0 | 2 | 5 | NCU00510T0\_input.txt | NCU00510T0\_output.txt | 0.348405 CGTTTTGTGGATGGTCTGCC | GACCTGACTGGCTGATTCCC | 0.492287 CGTTTTGTGGATGGTCTGCC | ACCAAGGACTCATGTTGGCC | 0.494699 GCGTTTTGTGGATGGTCTGC | GACCTGACTGGCTGATTCCC | 0.559221 CGTTTTGTGGATGGTCTGCC | CCCGTAATCGAGCTCTGAGC | 0.638581 GCGTTTTGTGGATGGTCTGC | ACCAAGGACTCATGTTGGCC | | | | | |
| NCU00512T0 | 1 | 5 | NCU00512T0\_input.txt | NCU00512T0\_output.txt | 0.287960 TTGGTTTGCGGTGCATATGC | AATCTCTCGCATCAGGCACC | 0.417242 ATCGGAGTTGACCAAGGTGC | CGACGACGAGACTCTTCTGG | 0.417933 ATCGGAGTTGACCAAGGTGC | ACAACACAACAACTGCGACG | 0.500714 ATCGGAGTTGACCAAGGTGC | AATCTCTCGCATCAGGCACC | 0.500837 ATCGGAGTTGACCAAGGTGC | GACGACGAGACTCTTCTGGC | | | | | |
| NCU00513T0 | 3 | 5 | NCU00513T0\_input.txt | NCU00513T0\_output.txt | 0.202186 GGGCGACATTGATTCATGGC | GGTGCACGTTGATGGATTGG | 0.486994 GGGCGACATTGATTCATGGC | GCCGATTGTGGACATTTCCC | 0.486994 GGGCGACATTGATTCATGGC | GGCCGATTGTGGACATTTCC | 0.487831 GGGCGACATTGATTCATGGC | GTGCACGTTGATGGATTGGC | 0.488277 GGCGACATTGATTCATGGCG | GGTGCACGTTGATGGATTGG | | | | | |
| NCU00515T2 | 4 | 5 | NCU00515T2\_input.txt | NCU00515T2\_output.txt | 0.706927 GGCGTCATGGGAGTAGAACG | TTCCCGATGATGACTGTCGC | 0.722860 AGATAGTGGGCACTTCAGGC | TTCCCGATGATGACTGTCGC | 0.842119 GGCGTCATGGGAGTAGAACG | ACGAGACTGACCCTTTCACG | 0.858052 AGATAGTGGGCACTTCAGGC | ACGAGACTGACCCTTTCACG | 0.990727 GATAGTGGGCACTTCAGGCG | TTCCCGATGATGACTGTCGC | | | | | |
| NCU00515T1 | 3 | 5 | NCU00515T1\_input.txt | NCU00515T1\_output.txt | 0.706927 GGCGTCATGGGAGTAGAACG | TTCCCGATGATGACTGTCGC | 0.722860 AGATAGTGGGCACTTCAGGC | TTCCCGATGATGACTGTCGC | 0.842119 GGCGTCATGGGAGTAGAACG | ACGAGACTGACCCTTTCACG | 0.858052 AGATAGTGGGCACTTCAGGC | ACGAGACTGACCCTTTCACG | 0.990727 GATAGTGGGCACTTCAGGCG | TTCCCGATGATGACTGTCGC | | | | | |
| NCU00515T0 | 5 | 5 | NCU00515T0\_input.txt | NCU00515T0\_output.txt | 0.218291 ACCAGCGACAGTCATCATCG | GGGCTCAAAGGCAAAAGACG | 0.641885 ACCAGCGACAGTCATCATCG | GATCAGGGAAAGCAGGGTCG | 0.779118 ACCAGCGACAGTCATCATCG | TCGGATGTGTATGATGGCGC | 0.843593 ACCAGCGACAGTCATCATCG | GGCTCAAAGGCAAAAGACGC | 0.847661 ACCAGCGACAGTCATCATCG | AAGGCAAAAGACGCTTCACC | | | | | |
| NCU00516T0 | 1 | 5 | NCU00516T0\_input.txt | NCU00516T0\_output.txt | 0.147288 CATGCAGTGAAGTGAACGGC | GTCTTGGGAGTCAGGGAACG | 0.213993 CATCCCCACTCTCAGTCAGC | TTCGGATGAGGACCACTTGC | 0.218563 CATCCCCACTCTCAGTCAGC | CTTGCCGTTCACTTCACTGC | 0.271273 GACAAAGAAAGACGTGGGCG | TTCGGATGAGGACCACTTGC | 0.272582 GACAAAGAAAGACGTGGGCG | TCACTGCATGTCGTGGTACC | | | | | |
| NCU00517T0 | 1 | 5 | NCU00517T0\_input.txt | NCU00517T0\_output.txt | 0.282333 GTTCGGTTCCATGCGTATGC | TTACCATGCACCCTACTGGC | 0.285655 GCGGGACCTAGACAAGATCG | TTACCATGCACCCTACTGGC | 0.348708 GTTCGGTTCCATGCGTATGC | CAACTCTCAGTCACCCCTGC | 0.349582 GTTCGGTTCCATGCGTATGC | GTCCAACTCTCAGTCACCCC | 0.352030 GCGGGACCTAGACAAGATCG | CAACTCTCAGTCACCCCTGC | | | | | |
| NCU00518T0 | 3 | 5 | NCU00518T0\_input.txt | NCU00518T0\_output.txt | 0.357985 GTGGTGGCTTCTTACCCTGG | AACCGGTCATGGACATCACC | 0.931677 GTGGTGGCTTCTTACCCTGG | ATCACCCCAAGTCTTTGCCG | 0.996300 GCAGTGGTGGCTTCTTACCC | AACCGGTCATGGACATCACC | 0.996300 GGCAGTGGTGGCTTCTTACC | AACCGGTCATGGACATCACC | 1.047095 GTGGTGGCTTCTTACCCTGG | TCGAAATCATCCGCAAACCG | | | | | |
| NCU00519T0 | 3 | 5 | NCU00519T0\_input.txt | NCU00519T0\_output.txt | 0.565214 CGAATTTTGGCGACGATGGG | TCTGTACCTTGTGTGGCTCG | 0.783389 CGAATTTTGGCGACGATGGG | TATGTCACTGACCCTTCGCC | 0.933151 CGAATTTTGGCGACGATGGG | TCCTCTGTACCTTGTGTGGC | 1.065165 ATTTTGGCGACGATGGGAGG | TCTGTACCTTGTGTGGCTCG | 1.178610 GCGAATTTTGGCGACGATGG | TCTGTACCTTGTGTGGCTCG | | | | | |
| NCU00520T0 | 2 | 5 | NCU00520T0\_input.txt | NCU00520T0\_output.txt | 0.280909 ATGCAGCAGGAGAAAAACGC | TCAACTACGTCAACTCCGCC | 0.578877 TCTTGGGTAGGTCGATTGGC | TCAACTACGTCAACTCCGCC | 0.775217 GCAAGAGCAAATGCAGCAGG | TCAACTACGTCAACTCCGCC | 0.919170 TTCTTGCTGTCAGACCGTGC | TCAACTACGTCAACTCCGCC | 1.117740 ACCGTGCCTGATTCTCTGC | TCAACTACGTCAACTCCGCC | | | | | |
| NCU00521T0 | 4 | 5 | NCU00521T0\_input.txt | NCU00521T0\_output.txt | 0.490646 GTTGCGCGACAGGTATTTCC | TCCATCATGGAGGATGACGC | 0.492140 GTTGCGCGACAGGTATTTCC | ATCATGGAGGATGACGCAGG | 0.559213 GTTGCGCGACAGGTATTTCC | TGGCTTTTGGGCGTTTTTGG | 0.573327 ATCGCTTCTTTCTCGACGGG | TCCATCATGGAGGATGACGC | 0.574820 ATCGCTTCTTTCTCGACGGG | ATCATGGAGGATGACGCAGG | | | | | |
| NCU00522T0 | 2 | 5 | NCU00522T0\_input.txt | NCU00522T0\_output.txt | 0.194910 GCTTCTCTTTCGGTTCACGC | GTTAGGATGGCCTCGAGACG | 0.279981 CTATGCCGAGAACCCAGACG | GTTAGGATGGCCTCGAGACG | 0.352098 CTATGCCGAGAACCCAGACG | TCAGGAAGACGATCATGGCG | 0.423369 TCCACCACCTTTCGCTATGC | GTTAGGATGGCCTCGAGACG | 0.425397 GGCCTTCCCTAACAAGGAGC | GTTAGGATGGCCTCGAGACG | | | | | |
| NCU00523T0 | 2 | 5 | NCU00523T0\_input.txt | NCU00523T0\_output.txt | 0.483822 GAGGTTACAGTGGACGTCGG | GTAGTCGAGACTCTGACGGC | 0.569988 TTACATCATCCACTGGCGGG | TCGTTCCTGCAATCTGTCGG | 0.699642 GAGGTTACAGTGGACGTCGG | GAGACTCTGACGGCGAATCG | 0.921326 GAGGTTACAGTGGACGTCGG | AACTCAGACGATCTCGGTGG | 0.969012 TGAGGTTACAGTGGACGTCG | GTAGTCGAGACTCTGACGGC | | | | | |
| NCU00524T0 | 1 | 5 | NCU00524T0\_input.txt | NCU00524T0\_output.txt | 0.136576 TCGTATGAGTCTGGCCATGC | CCGACTAACATACCCGGAGC | 0.140294 TCGTATGAGTCTGGCCATGC | ACATACCCGGAGCAACAAGG | 0.210699 GGGAGGATCAGGGATTTGGC | CCGACTAACATACCCGGAGC | 0.214417 GGGAGGATCAGGGATTTGGC | ACATACCCGGAGCAACAAGG | 0.354267 GGGGTGAGTGGTCATGTTCC | CCGACTAACATACCCGGAGC | | | | | |
| NCU00525T0 | 1 | 5 | NCU00525T0\_input.txt | NCU00525T0\_output.txt | 0.217321 TCTTGGTCGATTTCGGTCGG | ATGAGGCGTCTTAACACCGG | 0.218973 CAAAGGTGACTCGCTTTCGC | ATGAGGCGTCTTAACACCGG | 0.218973 GCAAAGGTGACTCGCTTTCG | ATGAGGCGTCTTAACACCGG | 0.291516 AGCAACATGGGCTTCCTAGG | ACCTACCCTTCCAAATGGCG | 0.363076 TCGCCATTTGGAAGGGTAGG | ATGAGGCGTCTTAACACCGG | | | | | |
| NCU00526T0 | 3 | 5 | NCU00526T0\_input.txt | NCU00526T0\_output.txt | 0.212097 TTTCCCAGAGGTGTCGAACG | AGCGGACAATCGTCTTACCG | 0.347255 TTTCCCAGAGGTGTCGAACG | ACAACCTCGGTGTCTCTTCG | 0.439139 ATTAACAAGGGGCCTGGACC | AAAAAGCTGGCAACGAACCC | 0.493447 AGAGGTGTCGAACGTGAAGG | AGCGGACAATCGTCTTACCG | 0.514164 ATTAACAAGGGGCCTGGACC | AGCGGACAATCGTCTTACCG | | | | | |
| NCU00527T0 | 5 | 5 | NCU00527T0\_input.txt | NCU00527T0\_output.txt | 0.570354 ATTGACCTACGGGCATGTCC | GGCACGATAATAGGCCTCGG | 0.570354 ATTGACCTACGGGCATGTCC | GGGCACGATAATAGGCCTCG | 0.854321 ATTGGGCCAGTACATGCTCG | GGCACGATAATAGGCCTCGG | 0.854321 ATTGGGCCAGTACATGCTCG | GGGCACGATAATAGGCCTCG | 0.855408 TTTCAGATATGGTGCCGGCC | GGCACGATAATAGGCCTCGG | | | | | |
| NCU00528T0 | 3 | 5 | NCU00528T0\_input.txt | NCU00528T0\_output.txt | 0.493674 ACCTGCGCCACTATTGATGG | CAATGCGATTACGGCTTGGG | 0.572222 ACCTGCGCCACTATTGATGG | AATTCTTGGTAGACGCGGGG | 0.695674 GAGTTTGGACGGGTTGTTGG | CAATGCGATTACGGCTTGGG | 0.774222 GAGTTTGGACGGGTTGTTGG | AATTCTTGGTAGACGCGGGG | 0.776555 CAACTGGCTGTCACTCGAGG | AATTCTTGGTAGACGCGGGG | | | | | |
| NCU00529T0 | 3 | 5 | NCU00529T0\_input.txt | NCU00529T0\_output.txt | 0.286331 AAGCATGCCGTAAGTACCCC | TAGGCTGACGGTAGGATCCC | 0.429204 TCTCATGAAACCGGCTCAGG | TAGGCTGACGGTAGGATCCC | 0.436957 AAGCATGCCGTAAGTACCCC | AGCTGTCCAAGGGTGATTCC | 0.569094 TCGTACACAGACACCCAACC | TAGGCTGACGGTAGGATCCC | 0.579830 TCTCATGAAACCGGCTCAGG | AGCTGTCCAAGGGTGATTCC | | | | | |
| NCU00530T0 | 4 | 5 | NCU00530T0\_input.txt | NCU00530T0\_output.txt | 0.417035 CCTCTGGGATCTGCTGTACG | TACGCCTATTCGTCTTCGCC | 0.708854 CCTCTGGGATCTGCTGTACG | AACTGGGACGAGAACCATGC | 0.769003 CCTCTGGGATCTGCTGTACG | ATCGCCATTTTCATGTCCGC | 0.845870 CCTCTGGGATCTGCTGTACG | TATTCGTCTTCGCCCTGTCC | 0.919317 CCTCTGGGATCTGCTGTACG | ATTTTCATGTCCGCCCAAGC | | | | | |
| NCU00531T0 | 3 | 5 | NCU00531T0\_input.txt | NCU00531T0\_output.txt | 0.205485 CTTTATCGAGTTTGCCCGCG | CAATAACGCGTGGAACAGCC | 0.271279 CTTTATCGAGTTTGCCCGCG | TGTTCCTCGAGAAGAGCAGC | 0.335278 CTTTATCGAGTTTGCCCGCG | GCCAGAGTGAAGTGTGTTGC | 0.344962 CCAGAGCATTCAACATGGCG | TGTTCCTCGAGAAGAGCAGC | 0.355186 CGCGTTCTCCTTTTCCTTGC | TGTTCCTCGAGAAGAGCAGC | | | | | |
| NCU00532T0 | 3 | 5 | NCU00532T0\_input.txt | NCU00532T0\_output.txt | 0.649537 CTAACTCTGCCTCCAGGTGG | TGAGGTTGGTTTGAGCCTCC | 0.729454 ACCCTAACTCTGCCTCCAGG | TGAGGTTGGTTTGAGCCTCC | 1.001169 CTGACCCATTCCCAAACTGC | TGAGGTTGGTTTGAGCCTCC | 1.004511 CTAACTCTGCCTCCAGGTGG | TCCAGTTCCACCACAACAGC | 1.084428 ACCCTAACTCTGCCTCCAGG | TCCAGTTCCACCACAACAGC | | | | | |
| NCU00533T0 | 3 | 5 | NCU00533T0\_input.txt | NCU00533T0\_output.txt | 0.714633 ACCCAAGGAACCGTAAGTGC | TGACCGATCGAATCCTTGGG | 0.788979 ATGAGACACCCAAGGAACCG | TGACCGATCGAATCCTTGGG | 0.989009 ACCCAAGGAACCGTAAGTGC | GCTTGGTCATGCATGTTGGC | 1.060065 ATCAACTCCCTCCACAACGC | GCTTGGTCATGCATGTTGGC | 1.063355 ATGAGACACCCAAGGAACCG | GCTTGGTCATGCATGTTGGC | | | | | |
| NCU00534T0 | 3 | 5 | NCU00534T0\_input.txt | NCU00534T0\_output.txt | 0.291907 ACCTTTCTCCCGTAATGGCC | AGACTCCCAGCTTTTTCGGG | 0.652206 TTCTGACCCTCATCAGGTGC | AGACTCCCAGCTTTTTCGGG | 0.654695 TCTCCCCCAGTCTGTAAGCC | AGACTCCCAGCTTTTTCGGG | 0.725237 TCCTGCCCAGAGATATCGGG | AGCAGGATTCTAGCCTTGGC | 0.774589 CAGTCTGTAAGCCGCACTCC | AGACTCCCAGCTTTTTCGGG | | | | | |
| NCU00535T0 | 2 | 5 | NCU00535T0\_input.txt | NCU00535T0\_output.txt | 0.781762 CTGTGCCAATGAAGCTTGCC | TGGCATTGTGGTGAAAAGCC | 0.913627 TAACGCTGTGCCAATGAAGC | TGGCATTGTGGTGAAAAGCC | 1.191920 CTGTGCCAATGAAGCTTGCC | AAAAGCCAGGTATGTTCGCG | 1.323785 TAACGCTGTGCCAATGAAGC | AAAAGCCAGGTATGTTCGCG | 1.404822 GCTGTGCCAATGAAGCTTGC | TGGCATTGTGGTGAAAAGCC | | | | | |
| NCU00536T0 | 1 | 5 | NCU00536T0\_input.txt | NCU00536T0\_output.txt | 0.284737 TGCTTCCCAATCGACTAGCC | GGCTTGGTGTTCCTATCCCC | 0.351391 GCACATCCACCTTTGCTTCC | GGCTTGGTGTTCCTATCCCC | 0.553285 GCCGAGATTTCACGAAACCC | GGCTTGGTGTTCCTATCCCC | 0.568016 TGCTTCCCAATCGACTAGCC | GTGGTGTCACTCGATGGTCC | 0.634234 CCACCTTTGCTTCCCAATCG | GGCTTGGTGTTCCTATCCCC | | | | | |
| NCU00537T0 | 2 | 5 | NCU00537T0\_input.txt | NCU00537T0\_output.txt | 0.549714 CTGACGAGAAAAGCGAAGGC | ACACAGAGGGTTTGCTGAGG | 0.748132 CTGACGAGAAAAGCGAAGGC | CGCGGATTACTTCATTGGCC | 0.748132 CTGACGAGAAAAGCGAAGGC | GGCGCGGATTACTTCATTGG | 0.757391 CTGACGAGAAAAGCGAAGGC | GCGCGGATTACTTCATTGGC | 0.769808 AAGAATAGACCAAGGGCGCG | CGCGGATTACTTCATTGGCC | | | | | |
| NCU00538T0 | 4 | 5 | NCU00538T0\_input.txt | NCU00538T0\_output.txt | 1.230245 GATCCCGAGCTCCAACTAGC | AGTGCTGGGAGAACTGGAGG | 1.672000 ACACTGGCACTGGAGAATACC | CTGGCCTCAGAAGCTTGAGG | 1.677212 ACACTGGCACTGGAGAATACC | TGAAGTGCTGGGAGAACTGG | 2.422567 CACTGGCACTGGAGAATACC | CTGGCCTCAGAAGCTTGAGG | 2.427779 CACTGGCACTGGAGAATACC | TGAAGTGCTGGGAGAACTGG | | | | | |
| NCU00541T1 | 6 | 5 | NCU00541T1\_input.txt | NCU00541T1\_output.txt | 0.768103 ACGTTGGGCAGCATAGAAGG | GTACATCTTGCGGCTCTTGC | 0.832548 GGGCAGCATAGAAGGTTTGC | GTACATCTTGCGGCTCTTGC | 1.258388 GGTAGTTCCTAAGCTCCCGC | CTTGCGGCTCTTGCATTGG | 1.349606 TTTGCAAGTTGGAGGGCTGG | GCAAAGAGGTGGTTCGAACG | 1.350210 AGGTTTGCAAGTTGGAGGGC | GCAAAGAGGTGGTTCGAACG | | | | | |
| NCU00541T0 | 6 | 5 | NCU00541T0\_input.txt | NCU00541T0\_output.txt | 0.768103 ACGTTGGGCAGCATAGAAGG | GTACATCTTGCGGCTCTTGC | 0.832548 GGGCAGCATAGAAGGTTTGC | GTACATCTTGCGGCTCTTGC | 1.258388 GGTAGTTCCTAAGCTCCCGC | CTTGCGGCTCTTGCATTGG | 1.349606 TTTGCAAGTTGGAGGGCTGG | GCAAAGAGGTGGTTCGAACG | 1.350210 AGGTTTGCAAGTTGGAGGGC | GCAAAGAGGTGGTTCGAACG | | | | | |
| NCU00542T0 | 1 | 5 | NCU00542T0\_input.txt | NCU00542T0\_output.txt | 0.215334 ATTTGGCGTATGTGAGGGGG | ATTCCGTCAGCGACTCTTGG | 0.216752 AAGTTGAATCTCGGCGAGGG | ATTCCGTCAGCGACTCTTGG | 0.427227 ATTTGGCGTATGTGAGGGGG | GTTCACACCCTCTTCGTCCC | 0.628576 AAGAGTCGCTGACGGAATGG | CGGGTTCGTCATTTGTTCCC | 0.698979 ACAGTTGAGAGTTGGGTGGC | CGGGTTCGTCATTTGTTCCC | | | | | |
| NCU00545T0 | 3 | 5 | NCU00545T0\_input.txt | NCU00545T0\_output.txt | 0.288707 TTACTCTTGCCGCCTGATCG | CTGCTTTCGTTGGTGATGGC | 0.288707 TTACTCTTGCCGCCTGATCG | GCTGCTTTCGTTGGTGATGG | 0.352818 TTACTCTTGCCGCCTGATCG | ACCGATTTCTCAGAGTCCGC | 0.499010 TTACTCTTGCCGCCTGATCG | TTTCTCAGAGTCCGCACAGC | 0.622979 TTACTCTTGCCGCCTGATCG | CTTTCGTTGGTGATGGCTCG | | | | | |
| NCU00547T0 | 3 | 5 | NCU00547T0\_input.txt | NCU00547T0\_output.txt | 0.497791 ATAAATGGGGCCGAAGACCG | GATGATACCTCCACCTCCGC | 0.497791 ATAAATGGGGCCGAAGACCG | GCGATGATACCTCCACCTCC | 0.502003 ATAAATGGGGCCGAAGACCG | ATGGCTGCGATGATACCTCC | 0.567882 TCCGTATGTGTGGCTCTTGG | GATGATACCTCCACCTCCGC | 0.567882 TCCGTATGTGTGGCTCTTGG | GCGATGATACCTCCACCTCC | | | | | |
| NCU00548T0 | 5 | 5 | NCU00548T0\_input.txt | NCU00548T0\_output.txt | 0.277824 GTCTCTTCCTTGAGCTCCCG | AGTCGCGAACATCTCCATCC | 0.499735 AAAGCTTGCGCAACATAGCC | TCAAGAATACCCGGCACACG | 0.565220 GTCTCTTCCTTGAGCTCCCG | TCAAGAATACCCGGCACACG | 0.644006 AAAGCTTGCGCAACATAGCC | AGAGCTTTCCCTCTCTTCGC | 0.709490 GTCTCTTCCTTGAGCTCCCG | AGAGCTTTCCCTCTCTTCGC | | | | | |
| NCU00549T0 | 1 | 5 | NCU00549T0\_input.txt | NCU00549T0\_output.txt | 0.222765 ACAAAGGGACAGAACCACCC | TCAGAACCCCATTTGTCCGG | 0.298755 ACAAAGGGACAGAACCACCC | TAACCCCTCCACCCAAAAGC | 0.353459 TGCGGAATGTTTTTCTCGGC | TAACCCCTCCACCCAAAAGC | 0.365111 ACAAAGGGACAGAACCACCC | CAGCTCCTCCATCAGAACCC | 0.426756 GTGTGGAGAAGAGTGCGAGG | TCAGAACCCCATTTGTCCGG | | | | | |
| NCU00550T1 | 5 | 5 | NCU00550T1\_input.txt | NCU00550T1\_output.txt | 0.198013 GTGAACAATGCGACGAGACG | GCACCTTTGACCTATCCCCC | 0.259471 GTGAACAATGCGACGAGACG | CGATGAACCGGGTCTTTTGC | 0.269412 GTGAACAATGCGACGAGACG | AAGACCATCGATGAACCGGG | 0.412114 GTGAACAATGCGACGAGACG | TGAAGGAGCTTGCGAAGACC | 0.487855 CATCCATACCTCTCTGCCCG | CGATGAACCGGGTCTTTTGC | | | | | |
| NCU00550T0 | 4 | 5 | NCU00550T0\_input.txt | NCU00550T0\_output.txt | 0.198013 GTGAACAATGCGACGAGACG | GCACCTTTGACCTATCCCCC | 0.259471 GTGAACAATGCGACGAGACG | CGATGAACCGGGTCTTTTGC | 0.269412 GTGAACAATGCGACGAGACG | AAGACCATCGATGAACCGGG | 0.412114 GTGAACAATGCGACGAGACG | TGAAGGAGCTTGCGAAGACC | 0.487855 CATCCATACCTCTCTGCCCG | CGATGAACCGGGTCTTTTGC | | | | | |
| NCU00551T0 | 3 | 5 | NCU00551T0\_input.txt | NCU00551T0\_output.txt | 0.144717 TTCAGATGGGTCAGTTCGGC | AAGGCCGATCGTGTGTTAGG | 0.287418 CTGAGGCCTCGTTCAGATGG | AAGGCCGATCGTGTGTTAGG | 0.288585 TTCAGATGGGTCAGTTCGGC | ATCGTGTGTTAGGAAGGGGC | 0.363006 TTCAGATGGGTCAGTTCGGC | TAGGAAGGGGCTGAGAAGGG | 0.429627 TAGCCTTGTCGAACTTGGCC | AAGGCCGATCGTGTGTTAGG | | | | | |
| NCU00552T0 | 3 | 5 | NCU00552T0\_input.txt | NCU00552T0\_output.txt | 0.500043 TGTGTGGATGAGACTGCAGC | ATGGGGTTCATCATGGCTCG | 0.637034 GACGTCTACTCCGGCTTTGG | ATGGGGTTCATCATGGCTCG | 0.777074 GGTAGCCAGCTTTTGTGTGG | ATGGGGTTCATCATGGCTCG | 1.058775 AACTGTGACGTCTACTCCGG | ATGGGGTTCATCATGGCTCG | 1.258622 TGTGGATGAGACTGCAGCG | ATGGGGTTCATCATGGCTCG | | | | | |
| NCU00553T0 | 7 | 5 | NCU00553T0\_input.txt | NCU00553T0\_output.txt | 0.316727 GCACTACGCGTCGTTTATCG | GCTCGAGTTGTTGATGACGC | 0.343194 ATTCTTCCGCGATCTTCCCG | GCTCGAGTTGTTGATGACGC | 0.483653 CCCCGAGAACTTCTTCCACG | GCTCGAGTTGTTGATGACGC | 0.586534 GCACTACGCGTCGTTTATCG | CTCGAGTTGTTGATGACGCG | 0.586534 GCACTACGCGTCGTTTATCG | CGCTCGAGTTGTTGATGACG | | | | | |
| NCU00554T0 | 3 | 5 | NCU00554T0\_input.txt | NCU00554T0\_output.txt | 0.426460 TGACCGTGATCTCTGCAAGG | AGCCTCAGCGTTAAGGATGG | 0.497440 TCTTTGACGAGGCAGACAGG | AGCCTCAGCGTTAAGGATGG | 0.638942 CAAGTTTGTTGCCCTGAGCC | ATCAACGCTCCACACCAAGG | 0.639987 GACCGTGATCTCTGCAAGGG | AGCCTCAGCGTTAAGGATGG | 0.852644 ATACACTGTCAGCGTTGGCC | AGCCTCAGCGTTAAGGATGG | | | | | |
| NCU00556T0 | 1 | 5 | NCU00556T0\_input.txt | NCU00556T0\_output.txt | 0.357143 CGCTCTTACCCTCCGAATGG | AAACATCGCCTACCAGCTCC | 0.357227 AATACGCATACGAACCGGGG | AAACATCGCCTACCAGCTCC | 0.359545 CGCTCTTACCCTCCGAATGG | CACTGTGCCTCAAACATCGC | 0.359629 AATACGCATACGAACCGGGG | CACTGTGCCTCAAACATCGC | 0.489455 CCCTCCGAATGGCATTTTCG | AAACATCGCCTACCAGCTCC | | | | | |
| NCU00557T0 | 4 | 5 | NCU00557T0\_input.txt | NCU00557T0\_output.txt | 0.147109 TTGAGATGAACACCCGACCG | CGACGGACACTTCCACTACC | 0.498471 CCACTTCTGGAGGTTACGGG | CGAGGTAGGAGCATCCAACG | 0.640335 CACTTCTGGAGGTTACGGGC | CGAGGTAGGAGCATCCAACG | 1.134594 CCACTTCTGGAGGTTACGGG | CTAGCGAGGTAGGAGCATCC | 1.250556 CCACTTCTGGAGGTTACGGG | GGCTACAGCTCTTACCTAGCG | | | | | |
| NCU00558T0 | 7 | 5 | NCU00558T0\_input.txt | NCU00558T0\_output.txt | 1.454215 GAGGGGAGTGGTAATGTGCC | AGAGATCCAGCAATCCATCGG | 1.641760 GAGGGGAGTGGTAATGTGCC | GCAATCCATCGGTCATTGTGC | 1.677317 ACATTTACTGGGTTCGGGGG | AGAGATCCAGCAATCCATCGG | 1.864862 ACATTTACTGGGTTCGGGGG | GCAATCCATCGGTCATTGTGC | 1.913034 GAGGGGAGTGGTAATGTGCC | GAGATCCAGCAATCCATCGG | | | | | |
| NCU00559T0 | 2 | 5 | NCU00559T0\_input.txt | NCU00559T0\_output.txt | 0.682592 CTTACTCGAATCTGCTGCGC | GTGGAAGTGGATGGGAGACG | 0.692534 TAACTGTTTTGACAGCGCCG | GTGGAAGTGGATGGGAGACG | 0.848757 GAAACCAAGGGCGACATTCC | AACAGTCCTTCCAATCCCGC | 0.897000 CTTACTCGAATCTGCTGCGC | AACAGTCCTTCCAATCCCGC | 0.906942 TAACTGTTTTGACAGCGCCG | AACAGTCCTTCCAATCCCGC | | | | | |
| NCU00560T0 | 2 | 5 | NCU00560T0\_input.txt | NCU00560T0\_output.txt | 0.571419 GCCTCTTCCTCCCAAGAACC | TTTTCTGAACCCGGTCTGGC | 0.716396 CAGATGTGCCTCTTCCTCCC | TTTTCTGAACCCGGTCTGGC | 0.716396 CCAGATGTGCCTCTTCCTCC | TTTTCTGAACCCGGTCTGGC | 1.107464 GCCTCTTCCTCCCAAGAACC | TTTCTGAACCCGGTCTGGC | 1.252441 CAGATGTGCCTCTTCCTCCC | TTTCTGAACCCGGTCTGGC | | | | | |
| NCU00561T0 | 2 | 5 | NCU00561T0\_input.txt | NCU00561T0\_output.txt | 0.504947 TGATCTGGGCTGGTTATCGC | TGAGCCTGAGGATGGAGAGG | 0.655218 TGATCTGGGCTGGTTATCGC | GATGGAGAGGGGTGGAAAGG | 0.715016 TGATCTGGGCTGGTTATCGC | GAAAGGGTGGAAGGGGAACG | 0.948042 ATTCCGGCTATCAGACAGCG | AGCTAAAAGAGAGGCCAGGG | 1.022072 TGATCTGGGCTGGTTATCGC | AGCTAAAAGAGAGGCCAGGG | | | | | |
| NCU00562T0 | 1 | 5 | NCU00562T0\_input.txt | NCU00562T0\_output.txt | 0.282514 ATTGAGGAAGCCGAGTTGGG | GAGCTTTCGTCTCGCAATGC | 0.288770 CTTGTTTGAACTCCCACGCG | GAGCTTTCGTCTCGCAATGC | 0.575510 ATTGAGGAAGCCGAGTTGGG | GAACCTAACCCCCTTCTCCG | 0.581766 CTTGTTTGAACTCCCACGCG | GAACCTAACCCCCTTCTCCG | 0.636884 AAAGAATCGGGTGGTCGTCG | GAGCTTTCGTCTCGCAATGC | | | | | |
| NCU00563T0 | 4 | 5 | NCU00563T0\_input.txt | NCU00563T0\_output.txt | 0.207832 GGGCGAAGATCGGACATAGG | CAAGCTCCATGACAAAGGCG | 0.210631 GAACGCTCTTGTTGTGCTGG | CAAGCTCCATGACAAAGGCG | 0.419161 ATCGGACATAGGCTGAACGC | CAAGCTCCATGACAAAGGCG | 0.501813 GGGCGAAGATCGGACATAGG | TTCATGATGTAGGCACGCCC | 0.504612 GAACGCTCTTGTTGTGCTGG | TTCATGATGTAGGCACGCCC | | | | | |
| NCU00564T0 | 3 | 5 | NCU00564T0\_input.txt | NCU00564T0\_output.txt | 0.418328 GGGTGGTGTTGCGAATAACG | AGCTTTTCAGTGGGCAGAGG | 0.427117 TGGTGTAGGCATGCATCTGG | GAATCTCTCTCCCGATGCCC | 0.427117 TGGTGTAGGCATGCATCTGG | GGAATCTCTCTCCCGATGCC | 0.427117 TGGTGTAGGCATGCATCTGG | GGGAATCTCTCTCCCGATGC | 0.487302 GGGTGGTGTTGCGAATAACG | GAATCTCTCTCCCGATGCCC | | | | | |
| NCU00565T0 | 2 | 5 | NCU00565T0\_input.txt | NCU00565T0\_output.txt | 0.069218 AGACTGAGGAGGAGCTCTGG | AGATGTCTCTTGGTTGGGCG | 0.076361 TCTGGGTGAGACTGAGGAGG | AGATGTCTCTTGGTTGGGCG | 0.131408 AGACTGAGGAGGAGCTCTGG | TGATGTACTTCTCGACCGCG | 0.138552 TCTGGGTGAGACTGAGGAGG | TGATGTACTTCTCGACCGCG | 0.206915 AGACTGAGGAGGAGCTCTGG | ACTAGCGCAGTACAGGAAGC | | | | | |
| NCU00566T0 | 4 | 5 | NCU00566T0\_input.txt | NCU00566T0\_output.txt | 1.133182 CATTCACGAGCAGCTTAGCG | CCCTATCACCCACAGACAGG | 1.597009 GCCCACAATCAAGCAGATGC | AGACGGAATCATGACCCATCC | 1.838032 AGGTCAAACATACCTGCTGGG | CCCTATCACCCACAGACAGG | 1.922344 CATTCACGAGCAGCTTAGCG | TCCCTATCACCCACAGACAGG | 1.994526 GCCCACAATCAAGCAGATGC | CGGAATCATGACCCATCCACC | | | | | |
| NCU00567T1 | 3 | 5 | NCU00567T1\_input.txt | NCU00567T1\_output.txt | 0.213759 GGATATGAGGATGGGCTGGC | CCTGATGCTCTCTCCAACCG | 0.213759 GGATATGAGGATGGGCTGGC | AGTCACTCGATGCCTGATGC | 0.287609 ACTGGTATGGCGTTGTACCG | CCTGATGCTCTCTCCAACCG | 0.287609 ACTGGTATGGCGTTGTACCG | AGTCACTCGATGCCTGATGC | 0.354397 GGATATGAGGATGGGCTGGC | CGATGCCTGATGCTCTCTCC | | | | | |
| NCU00567T0 | 2 | 5 | NCU00567T0\_input.txt | NCU00567T0\_output.txt | 0.218707 TGACTCCCAAATCCCTTGCC | CCTGATGCTCTCTCCAACCG | 0.218707 TGACTCCCAAATCCCTTGCC | AGTCACTCGATGCCTGATGC | 0.359345 TGACTCCCAAATCCCTTGCC | CGATGCCTGATGCTCTCTCC | 0.503092 TGACTCCCAAATCCCTTGCC | GCCTGATGCTCTCTCCAACC | 0.504855 AGGACCCTAAGCTGACTCCC | CCTGATGCTCTCTCCAACCG | | | | | |
| NCU00568T0 | 2 | 5 | NCU00568T0\_input.txt | NCU00568T0\_output.txt | 0.425732 AGGTACCGCTGTTCTTTGGG | TACAAGTCGAAGCGAAGGGC | 0.497292 AGGTACCGCTGTTCTTTGGG | CAATGTCTCCGAGTCCCAGC | 0.502495 AGGTACCGCTGTTCTTTGGG | ATACCAGCTTGATTGGGGCG | 0.765897 ATGTGCTACGCTGCTTTTGG | GGGGCGTCAACCTTTTTAGC | 0.912498 ATGTGCTACGCTGCTTTTGG | TACAAGTCGAAGCGAAGGGC | | | | | |
| NCU00569T0 | 2 | 5 | NCU00569T0\_input.txt | NCU00569T0\_output.txt | 0.144867 AAGTAGACGTGAGCAGTGGC | TGTTGTCTCTCCACCTGTGC | 0.341592 GTTGGACGAAAGCCTCAACG | TGTTGTCTCTCCACCTGTGC | 0.426666 ACCAACAATTCCTCGAGGGC | AGAAAAACCCAACGCCAAGC | 0.429254 ACCAACAATTCCTCGAGGGC | TGTTGTCTCTCCACCTGTGC | 0.505081 AAGTAGACGTGAGCAGTGGC | CCCATCAGTCTGCAATCCCC | | | | | |
| NCU00570T0 | 3 | 5 | NCU00570T0\_input.txt | NCU00570T0\_output.txt | 0.143379 CCTGTCCTTACGAACACCCC | TGTTGTCTCTCCACCTGTGC | 0.211747 GACAAGCCCATCTACTCCCG | TGTTGTCTCTCCACCTGTGC | 0.211747 CGACAAGCCCATCTACTCCC | TGTTGTCTCTCCACCTGTGC | 0.213717 GACAGCGGAACTTGCAATGG | AGAAAAACCCAACGCCAAGC | 0.216305 GACAGCGGAACTTGCAATGG | TGTTGTCTCTCCACCTGTGC | | | | | |
| NCU00571T0 | 2 | 5 | NCU00571T0\_input.txt | NCU00571T0\_output.txt | 0.350138 ATGAAGGACGGGATGGAAGC | TTGCAGCATGTAAAGCGAGC | 0.633241 CTAACGGGCTGGTTAGGAGG | TTGCAGCATGTAAAGCGAGC | 0.834086 CGTACCTCTACCTAACGGGC | TTGCAGCATGTAAAGCGAGC | 0.855557 ACCTCTACCTAACGGGCTGG | TTGCAGCATGTAAAGCGAGC | 1.171447 TGGTGGTTGTATGAAGGACGG | TTGCAGCATGTAAAGCGAGC | | | | | |
| NCU00572T0 | 2 | 5 | NCU00572T0\_input.txt | NCU00572T0\_output.txt | 0.201366 GGCGTGAATGGAAATGGAGC | GTTGTAGCCGTCTACGAGGG | 0.201366 GGCGTGAATGGAAATGGAGC | GGTTGTAGCCGTCTACGAGG | 0.279219 CAAGGCAGAGTATGGGGACG | GTTGTAGCCGTCTACGAGGG | 0.279219 CAAGGCAGAGTATGGGGACG | GGTTGTAGCCGTCTACGAGG | 0.286921 CAAGGCAGAGTATGGGGACG | AATTCGGCGAGGGTTACTGG | | | | | |
| NCU00573T0 | 4 | 5 | NCU00573T0\_input.txt | NCU00573T0\_output.txt | 0.199543 TCCTTCTTCCGTCAAGTGGC | CTTCTCAAGGTTCTTGCGCG | 0.267673 TCACTCGATGGCAAGGATCG | CTTCTCAAGGTTCTTGCGCG | 0.343728 AAGGTTAAGATCGGCGAGCC | CTTCTCAAGGTTCTTGCGCG | 0.478634 TATCCTGTATGCCTGCGACG | CTTCTCAAGGTTCTTGCGCG | 0.553973 TCCTTCTTCCGTCAAGTGGC | CCTTCTCAAGGTTCTTGCGC | | | | | |
| NCU00574T0 | 3 | 5 | NCU00574T0\_input.txt | NCU00574T0\_output.txt | 0.337893 ATCAACCGATAGACACCCGC | CTCGATGTCTCCGCTACTCG | 0.342932 ATCACATCCATCGGAAGCCC | CTCGATGTCTCCGCTACTCG | 0.483824 TGATGTCCCTTTGCTCCTCG | CTCGATGTCTCCGCTACTCG | 0.761154 AGCAATGTCATTGAACCGGC | CTCGATGTCTCCGCTACTCG | 0.770049 TTTGCTCCTCGAACCAACCC | CTCGATGTCTCCGCTACTCG | | | | | |
| NCU00575T0 | 5 | 5 | NCU00575T0\_input.txt | NCU00575T0\_output.txt | 0.491899 CAGAGGAGGTTAGCCATCGG | ATGATGGCATTTCGCAACCG | 0.495802 TGTGGGAATCTGGCTCATGC | ATGATGGCATTTCGCAACCG | 0.565249 CAGAGGAGGTTAGCCATCGG | TTCGAGTCCTCAGATGCTGC | 0.569151 TGTGGGAATCTGGCTCATGC | TTCGAGTCCTCAGATGCTGC | 0.710702 CCTTTTCAGTGCCATTGGGG | ATGATGGCATTTCGCAACCG | | | | | |
| NCU00576T0 | 1 | 5 | NCU00576T0\_input.txt | NCU00576T0\_output.txt | 0.641397 TACCTTCCGTCTTCTGCTGC | GTCCGGGATTTACCAACCCC | 0.721679 TACCTTCCGTCTTCTGCTGC | TACCAACCCCGATTATGCCC | 0.868771 TACCTTCCGTCTTCTGCTGC | AGTCCGGGATTTACCAACCC | 0.906592 TACCTTCCGTCTTCTGCTGC | GAATGCCATCTCGTTGGAGC | 1.003893 TCCTAGTGCTGCTTTTGGGC | GTCCGGGATTTACCAACCCC | | | | | |
| NCU00577T0 | 2 | 5 | NCU00577T0\_input.txt | NCU00577T0\_output.txt | 0.215874 AGTTGTAGTGGGCTTCAGGC | AGCTTTCTGCCGTGATAGGG | 0.356388 TCCATCCACGGCGATATTCG | AAATGAGCGCAACAACGTGG | 0.357573 TCCATCCACGGCGATATTCG | AACGTGGCAGCTCATTTTGC | 0.361817 AGTTGTAGTGGGCTTCAGGC | AAGATGAGGTTGACCGTGGG | 0.363561 TCCATCCACGGCGATATTCG | AAGATGAGGTTGACCGTGGG | | | | | |
| NCU00578T0 | 3 | 5 | NCU00578T0\_input.txt | NCU00578T0\_output.txt | 0.140655 CGCTGTTGAAACCACCATGG | CAGGGTGGATCTCGTCTTCG | 0.370534 CGCTGTTGAAACCACCATGG | TATGTTTCAGGCCAGGGTGG | 0.627160 ATCAAAAATGGCCACCGACG | CAGGGTGGATCTCGTCTTCG | 0.717304 CGCTGTTGAAACCACCATGG | ACGGTGGAGTCGTAGTACCC | 0.768946 CGCTGTTGAAACCACCATGG | GACGGTGGAGTCGTAGTACC | | | | | |
| NCU00579T1 | 3 | 5 | NCU00579T1\_input.txt | NCU00579T1\_output.txt | 0.207888 ACGAGAACGGCATAAGGACC | GCTAGATCCAAGAGGTCGGC | 0.208301 ACGAGAACGGCATAAGGACC | CAGCGGCTAGATCCAAGAGG | 0.349918 ACGAGAACGGCATAAGGACC | TGGGCTCGTTGAAGGATACG | 0.425813 ATGGATGCGGACAACAGACC | GCTAGATCCAAGAGGTCGGC | 0.426226 ATGGATGCGGACAACAGACC | CAGCGGCTAGATCCAAGAGG | | | | | |
| NCU00579T0 | 3 | 5 | NCU00579T0\_input.txt | NCU00579T0\_output.txt | 0.207888 ACGAGAACGGCATAAGGACC | GCTAGATCCAAGAGGTCGGC | 0.208301 ACGAGAACGGCATAAGGACC | CAGCGGCTAGATCCAAGAGG | 0.349918 ACGAGAACGGCATAAGGACC | TGGGCTCGTTGAAGGATACG | 0.425813 ATGGATGCGGACAACAGACC | GCTAGATCCAAGAGGTCGGC | 0.426226 ATGGATGCGGACAACAGACC | CAGCGGCTAGATCCAAGAGG | | | | | |
| NCU00580T0 | 2 | 5 | NCU00580T0\_input.txt | NCU00580T0\_output.txt | 1.178384 TCAGCCGTGGTGGAATTCC | TATGGCTCAGTGGTTCTCGC | 1.481690 GTCAGCCGTGGTGGAATTCC | TATGGCTCAGTGGTTCTCGC | 2.081472 TCAGCCGTGGTGGAATTCC | ATGGCTCAGTGGTTCTCGC | 2.205087 TCAGCCGTGGTGGAATTCC | CCTATGGCTCAGTGGTTCTCG | 2.384778 GTCAGCCGTGGTGGAATTCC | ATGGCTCAGTGGTTCTCGC | | | | | |
| NCU00581T0 | 2 | 5 | NCU00581T0\_input.txt | NCU00581T0\_output.txt | 0.215039 AACGGGACAAGACAAGTGGG | ATGTCAACCTTCTGGAGCCG | 0.216416 ATGATTTCGGGAGGAACGGG | ATGTCAACCTTCTGGAGCCG | 0.216633 AACGGGACAAGACAAGTGGG | AACCTTCTGGAGCCGTTACG | 0.218011 ATGATTTCGGGAGGAACGGG | AACCTTCTGGAGCCGTTACG | 0.281456 GCACAATCATGCAAGTCCGG | GGGCCGTATGAAACATTGGC | | | | | |
| NCU00582T0 | 2 | 5 | NCU00582T0\_input.txt | NCU00582T0\_output.txt | 0.642334 TGGATGACAAGCCACGAAGG | TCTTCCTTGCCTTCTCACGC | 1.354357 TGGATGACAAGCCACGAAGG | TGAAGACATCGGGGAGATCG | 1.418821 GCCAAGTTGGAGCTGTATGG | TCTTCCTTGCCTTCTCACGC | 1.456352 TGGATGACAAGCCACGAAGG | GTAGGTGGTGAAGACATCGGG | 1.456352 TGGATGACAAGCCACGAAGG | GGTAGGTGGTGAAGACATCGG | | | | | |
| NCU00583T0 | 4 | 5 | NCU00583T0\_input.txt | NCU00583T0\_output.txt | 0.078507 CGTCTCATCCGATCAGAGCC | AAGGAAGGAAGGAAGCTGGC | 0.136914 GTGAGTCTCTCGCTCCTTCG | AAGGAAGGAAGGAAGCTGGC | 0.277240 GTGAGTCTCTCGCTCCTTCG | GAAACATGTGCGAAGGAGCG | 0.355322 CCAAGGGAAGGAAGGACTGC | TCCAGTAGTAGTCCAGGGGC | 0.358616 CATCCGATCAGAGCCTCAGC | AAGGAAGGAAGGAAGCTGGC | | | | | |
| NCU00584T0 | 1 | 5 | NCU00584T0\_input.txt | NCU00584T0\_output.txt | 0.562275 AGATCTTCCTGGATGCGTCG | AGTCCGCTAACCGTCAATCC | 0.618942 CAAACCAAGGAGCGATGACG | AGTCCGCTAACCGTCAATCC | 0.705715 TGATGCGAAGACGGGTAAGG | GATCAGCCAGTCCGCTAACC | 0.712304 ATTGACGGTTAGCGGACTGG | AGAAACCGGAGAAGCAGTGC | 0.712304 ATTGACGGTTAGCGGACTGG | AGCACGCTTCTGGAGAAACC | | | | | |
| NCU00585T0 | 3 | 5 | NCU00585T0\_input.txt | NCU00585T0\_output.txt | 1.103189 GGCTTCTGGTGGGTAAGACC | TCGAGACTGAACACATTCCCG | 1.319761 TGTTGAGAATGGGGTAGGCG | TCGAGACTGAACACATTCCCG | 1.385536 GGCTTCTGGTGGGTAAGACC | CGAGACTGAACACATTCCCG | 1.449636 TGTTGAGAATGGGGTAGGCG | GAAAAGCTGATGACACGTGGG | 1.490355 TGTTGAGAATGGGGTAGGCG | AAAAGCTGATGACACGTGGG | | | | | |
| NCU00586T0 | 5 | 5 | NCU00586T0\_input.txt | NCU00586T0\_output.txt | 0.862021 AGGAGGATGAGGGAGATGGC | GCTTTGGGCGTTCAATGTCC | 0.916337 GTATGTCTGTCGAAGGGGCG | GCTTTGGGCGTTCAATGTCC | 1.054794 TGTCCATTGTGAGACGTCGC | GCTTTGGGCGTTCAATGTCC | 1.133291 GCCGGAGAGGATGACAAACC | GCTTTGGGCGTTCAATGTCC | 1.136655 AGGAGGATGAGGGAGATGGC | GGCGTTCAATGTCCTATGCC | | | | | |
| NCU00586T1 | 4 | 5 | NCU00586T1\_input.txt | NCU00586T1\_output.txt | 0.862021 AGGAGGATGAGGGAGATGGC | GCTTTGGGCGTTCAATGTCC | 0.916337 GTATGTCTGTCGAAGGGGCG | GCTTTGGGCGTTCAATGTCC | 1.054794 TGTCCATTGTGAGACGTCGC | GCTTTGGGCGTTCAATGTCC | 1.133291 GCCGGAGAGGATGACAAACC | GCTTTGGGCGTTCAATGTCC | 1.136655 AGGAGGATGAGGGAGATGGC | GGCGTTCAATGTCCTATGCC | | | | | |
| NCU00586T2 | 4 | 5 | NCU00586T2\_input.txt | NCU00586T2\_output.txt | 0.862021 AGGAGGATGAGGGAGATGGC | GCTTTGGGCGTTCAATGTCC | 0.916337 GTATGTCTGTCGAAGGGGCG | GCTTTGGGCGTTCAATGTCC | 1.054794 TGTCCATTGTGAGACGTCGC | GCTTTGGGCGTTCAATGTCC | 1.133291 GCCGGAGAGGATGACAAACC | GCTTTGGGCGTTCAATGTCC | 1.136655 AGGAGGATGAGGGAGATGGC | GGCGTTCAATGTCCTATGCC | | | | | |
| NCU00587T1 | 3 | 5 | NCU00587T1\_input.txt | NCU00587T1\_output.txt | 0.477121 GTTACATGGCACCAGAACGC | GACAAAGTCCTGTGCTGTGC | 1.123003 GTGTGCCATGGGTCGATACC | GACAAAGTCCTGTGCTGTGC | 1.145393 ATACCCATACCCGCCTGAGG | GACAAAGTCCTGTGCTGTGC | 1.156821 ATACCCATACCCGCCTGAGG | TTCGGCTTCCACTTCTTCCG | 1.271525 GTTACATGGCACCAGAACGC | ACAAAGTCCTGTGCTGTGCC | | | | | |
| NCU00587T0 | 3 | 5 | NCU00587T0\_input.txt | NCU00587T0\_output.txt | 0.477121 GTTACATGGCACCAGAACGC | GACAAAGTCCTGTGCTGTGC | 1.123003 GTGTGCCATGGGTCGATACC | GACAAAGTCCTGTGCTGTGC | 1.145393 ATACCCATACCCGCCTGAGG | GACAAAGTCCTGTGCTGTGC | 1.156821 ATACCCATACCCGCCTGAGG | TTCGGCTTCCACTTCTTCCG | 1.271525 GTTACATGGCACCAGAACGC | ACAAAGTCCTGTGCTGTGCC | | | | | |
| NCU00589T0 | 3 | 5 | NCU00589T0\_input.txt | NCU00589T0\_output.txt | 1.651527 CCGAGACCATGTACGAGACG | TCAGTGTCTAAACCTCCTGGC | 1.846390 CCGAGACCATGTACGAGACG | AGCAATGGGTTAAAAGGCTCG | 1.900693 TGACGCCCAGTTCAATCAGC | TTAACGAAGCGGACAACGGG | 1.997787 GTTGCCAAATACGCCCATGG | AGCAATGGGTTAAAAGGCTCG | 2.001952 CCGAGACCATGTACGAGACG | ACCTCCTGGCTAGATGTATGC | | | | | |
| NCU00590T0 | 2 | 5 | NCU00590T0\_input.txt | NCU00590T0\_output.txt | 0.287543 TTGTCATGACCACCAACCCC | GAGAAGAGGAGCGGTGTTCC | 0.433027 ACACCTACCTCCCCTCTTCG | GAGAAGAGGAGCGGTGTTCC | 0.570769 TTGTCATGACCACCAACCCC | GAGGTTGAAGAGAAGGGCCG | 0.620220 GAACCCGAACGTTTCTCTGC | GAGAAGAGGAGCGGTGTTCC | 0.716253 ACACCTACCTCCCCTCTTCG | GAGGTTGAAGAGAAGGGCCG | | | | | |
| NCU00591T1 | 4 | 5 | NCU00591T1\_input.txt | NCU00591T1\_output.txt | 0.127307 CAGCTTCTTGATTACGCCGC | GAAGAGACTGAGGAGAGCGC | 0.279614 TCTTGATTACGCCGCTCTGG | GAAGAGACTGAGGAGAGCGC | 0.403655 CAGCTTCTTGATTACGCCGC | CGAAGAGACTGAGGAGAGCG | 0.414005 CAGCTTCTTGATTACGCCGC | TGAGGAGAGCGCTTAGATGC | 0.479113 GCCAGCTTCTTGATTACGCC | GAAGAGACTGAGGAGAGCGC | | | | | |
| NCU00591T0 | 3 | 5 | NCU00591T0\_input.txt | NCU00591T0\_output.txt | 0.127307 CAGCTTCTTGATTACGCCGC | GAAGAGACTGAGGAGAGCGC | 0.279614 TCTTGATTACGCCGCTCTGG | GAAGAGACTGAGGAGAGCGC | 0.403655 CAGCTTCTTGATTACGCCGC | CGAAGAGACTGAGGAGAGCG | 0.414005 CAGCTTCTTGATTACGCCGC | TGAGGAGAGCGCTTAGATGC | 0.479113 GCCAGCTTCTTGATTACGCC | GAAGAGACTGAGGAGAGCGC | | | | | |
| NCU00592T0 | 1 | 5 | NCU00592T0\_input.txt | NCU00592T0\_output.txt | 0.411504 ACCGATCTGGACAAAGCTCG | CTTCTTATTCTGCTGGCGCG | 0.422639 GGACCACAAAAATGCGGAGG | TCTCATCCGCCTCTCTCTCC | 0.488284 ACCGATCTGGACAAAGCTCG | CTGCAATCTTCATGTCCGCC | 0.491015 GCAAAGGAAGATGAGCAGCC | AGCTTTGTCCAGATCGGTGG | 0.493453 GCTGAATAAAGGTGGCAGGC | AGCTTTGTCCAGATCGGTGG | | | | | |
| NCU00593T0 | 2 | 5 | NCU00593T0\_input.txt | NCU00593T0\_output.txt | 1.363880 TAAGGCTGAAGCTCGAGTGG | GGCTAGTTCTACACTGGGGG | 1.363880 TCTGGTAAGGCTGAAGCTCG | GGCTAGTTCTACACTGGGGG | 1.502467 TAAGGCTGAAGCTCGAGTGG | AAGAGTCGAGGTGTCTTGGG | 1.502467 TCTGGTAAGGCTGAAGCTCG | AAGAGTCGAGGTGTCTTGGG | 1.739903 TAAGGCTGAAGCTCGAGTGG | GTGGCTAGTTCTACACTGGGG | | | | | |
| NCU00594T0 | 3 | 5 | NCU00594T0\_input.txt | NCU00594T0\_output.txt | 0.415808 TCAAACAAAGGCGGCAAACC | CACAAGCCAGAAGAAGCACG | 0.778497 TCTTTTCTGTCGATGGCGGG | CGTGACAACCTCTCTGGACG | 0.785346 TCTTTTCTGTCGATGGCGGG | TGGATTGGCCTTCATCCACG | 0.835130 GGTGCTGCTCAAACAAAGGC | CACAAGCCAGAAGAAGCACG | 0.846585 TCAAACAAAGGCGGCAAACC | TAGCGCAGACACTCACAAGC | | | | | |
| NCU00595T0 | 6 | 5 | NCU00595T0\_input.txt | NCU00595T0\_output.txt | 0.485199 CTGTCATGCTCACCACATGC | ACAGAGAGCAGCAGTTCACC | 0.492467 CACTCCTCTGATGGTCGTCC | ACAGAGAGCAGCAGTTCACC | 0.823607 CTGTCATGCTCACCACATGC | GATTCGCAACAGAGAGCAGC | 0.830875 CACTCCTCTGATGGTCGTCC | GATTCGCAACAGAGAGCAGC | 0.845897 ATTTGCCCACTCTTTAGCGC | ACAGAGAGCAGCAGTTCACC | | | | | |
| NCU00596T0 | 2 | 5 | NCU00596T0\_input.txt | NCU00596T0\_output.txt | 0.642157 ATTCCTGCGTCAGTGTCACC | GTCTTCACCTCCCCGAAACC | 0.717581 ATTCCTGCGTCAGTGTCACC | AAGTTGTCTTCACCTCCCCG | 0.781933 ATTCCTGCGTCAGTGTCACC | TATGGTCAACGTGATGCCGG | 0.787942 TGGTTTTGTCGACCTTGGGG | GTCTTCACCTCCCCGAAACC | 0.863366 TGGTTTTGTCGACCTTGGGG | AAGTTGTCTTCACCTCCCCG | | | | | |
| NCU00597T0 | 3 | 5 | NCU00597T0\_input.txt | NCU00597T0\_output.txt | 0.216722 TTGACACTGGAGGCTCAACC | GGACAGGTTCTGGTGATCCG | 0.346083 TTGACACTGGAGGCTCAACC | GCCTTGCGAACTGTGTAACC | 0.430256 TTGACACTGGAGGCTCAACC | ACTCTGGGTGAACACATCGG | 0.497234 TTGACACTGGAGGCTCAACC | AACACATCGGAGAGTGTCGC | 0.563185 TTGACACTGGAGGCTCAACC | TTCTGGTGATCCGTACTCGC | | | | | |
| NCU00598T1 | 3 | 5 | NCU00598T1\_input.txt | NCU00598T1\_output.txt | 0.129022 GCTGTCGACGTCAATCTTGC | GGCTTCTTTTGCCCCTTTGG | 0.142334 AATGAGGAAGGTCGGCATGG | GGCTTCTTTTGCCCCTTTGG | 0.189640 GCTGTCGACGTCAATCTTGC | CGCTCACGCATTCTTTCTGG | 0.206548 GCAAAATGAGGAAGGTCGGC | GGCTTCTTTTGCCCCTTTGG | 0.272884 GCTGTCGACGTCAATCTTGC | GCTCACGCATTCTTTCTGGC | | | | | |
| NCU00598T0 | 2 | 5 | NCU00598T0\_input.txt | NCU00598T0\_output.txt | 0.063296 AATCGCGCGATGATGTTTGG | GGCTTCTTTTGCCCCTTTGG | 0.123913 AATCGCGCGATGATGTTTGG | CGCTCACGCATTCTTTCTGG | 0.136164 AATCGCGCGATGATGTTTGG | TAGCATGCTGGAGTTCACGG | 0.144121 ACAGTCCGCAACTACGATGG | GGCTTCTTTTGCCCCTTTGG | 0.146847 GTGATAGAAGCAATCGCGCG | GGCTTCTTTTGCCCCTTTGG | | | | | |
| NCU00599T0 | 1 | 5 | NCU00599T0\_input.txt | NCU00599T0\_output.txt | 0.215116 TGAATCGCCGGGACTTAAGG | AACGTGTGGTCCGTGTATCC | 0.355675 TGAATCGCCGGGACTTAAGG | TTCGTGTAAATAGGGGGCCG | 0.427792 GGTTTTGAAATCTGCCCGCC | AACGTGTGGTCCGTGTATCC | 0.568350 GGTTTTGAAATCTGCCCGCC | TTCGTGTAAATAGGGGGCCG | 0.636384 ATCTTCGTCGCAGAGAAGGG | TTCGTGTAAATAGGGGGCCG | | | | | |
| NCU00600T0 | 5 | 5 | NCU00600T0\_input.txt | NCU00600T0\_output.txt | 1.361179 AGTACACCGCAAGTCAGACG | CGGAAGTCGAGCTTTCAATCG | 1.593479 AGTACACCGCAAGTCAGACG | TTTTGTCGCGGAAGTCGAGC | 1.710764 AGTACACCGCAAGTCAGACG | TCGAGTAACTGAGCATCTCCG | 1.835330 GTTTTCGAGCGAGTCCTTGC | TCGAGTAACTGAGCATCTCCG | 1.942919 GTCAAATTCTTCCTGGCCGG | TCCCTGTATCATGGTCTGCC | | | | | |
| NCU00602T0 | 1 | 5 | NCU00602T0\_input.txt | NCU00602T0\_output.txt | 0.270677 CTCGGGTTTCTGATCGAGGG | ACACGACATGACTACGACCG | 0.351181 CTCGGGTTTCTGATCGAGGG | GACATGACTACGACCGACCG | 0.351181 CTCGGGTTTCTGATCGAGGG | CGACATGACTACGACCGACC | 0.647002 TCTTTGCCACCTCTGCTTGG | CTACGGAACTTGGTCGGTCC | 0.794403 AGGATGCCATCTTTGCCACC | CTACGGAACTTGGTCGGTCC | | | | | |
| NCU00603T0 | 2 | 5 | NCU00603T0\_input.txt | NCU00603T0\_output.txt | 0.284511 TATACGCGGCCTTTTCTCCC | AAGCCAAGCACAACAACTGC | 0.284532 TATACGCGGCCTTTTCTCCC | GACAGACAGACGGAGATGGC | 0.287575 GCCCATGCTTTCTTCCTTGC | AAGCCAAGCACAACAACTGC | 0.287596 GCCCATGCTTTCTTCCTTGC | GACAGACAGACGGAGATGGC | 0.287599 GCCCATGCTTTCTTCCTTGC | GGGTCGCTTCCTACGTTACC | | | | | |
| NCU00604T0 | 2 | 5 | NCU00604T0\_input.txt | NCU00604T0\_output.txt | 0.056494 CTTGCACGATGAAGGATGCG | CAACCAGACAACAAGCCACG | 0.277677 GCTTGCACGATGAAGGATGC | CAACCAGACAACAAGCCACG | 0.400910 CTTGCACGATGAAGGATGCG | CGTAGCGGAATCCAACTTGC | 0.540145 CTTGCACGATGAAGGATGCG | CAGACAACAAGCCACGTAGC | 0.622093 GCTTGCACGATGAAGGATGC | CGTAGCGGAATCCAACTTGC | | | | | |
| NCU00605T0 | 3 | 5 | NCU00605T0\_input.txt | NCU00605T0\_output.txt | 0.212929 GACTGAGAAGATGGGCGAGG | TCGTACATCTTGTCACGGCC | 0.357817 CTCGACTGAGAAGATGGGCG | TCGTACATCTTGTCACGGCC | 0.500122 TTCAAGAAGGGCGATGTCGG | TCGTACATCTTGTCACGGCC | 0.500785 GGAGGAGTTCAAGAAGGGCG | TCGTACATCTTGTCACGGCC | 0.641425 TTCAAGAAGGGCGATGTCGG | TTCACTGGCCTGAACGAAGG | | | | | |
| NCU00606T0 | 2 | 5 | NCU00606T0\_input.txt | NCU00606T0\_output.txt | 0.292622 AAGATACGGTTCAGGTGGCC | CTTTGAACCGGGCAAGAACG | 0.642695 AGAACAGTCTTGCCCTGAGC | GTCTCTGGAGGTTGTTGGGC | 0.644257 AAGATACGGTTCAGGTGGCC | AACCGGGCAAGAACGATACC | 0.800912 AGAACAGTCTTGCCCTGAGC | TGTCTCTGGAGGTTGTTGGG | 1.013999 AAGATACGGTTCAGGTGGCC | CCCCTCTAGAGTCCATTGCC | | | | | |
| NCU00607T0 | 2 | 5 | NCU00607T0\_input.txt | NCU00607T0\_output.txt | 0.565848 AAGAACCCTAACACCCACGC | GTTGGCCTCCAAGAACTTGC | 0.570757 AAGAACCCTAACACCCACGC | AACCCAACTTCGTCCTACGC | 0.787968 AAGAACCCTAACACCCACGC | CATCCATCCCCCGCTTTACC | 0.911085 AAGAACCCTAACACCCACGC | CACTCGACTCCCATCTCTCG | 0.919131 AAGAACCCTAACACCCACGC | GGAATGCAATACGGGCTTCC | | | | | |
| NCU00608T0 | 2 | 5 | NCU00608T0\_input.txt | NCU00608T0\_output.txt | 1.555433 ATAGACCGAGATGCCAGACG | ACGGAAGCGAAGTGTTGAGG | 1.556489 ATAGACCGAGATGCCAGACG | GAGATTCGGGAAGGCATCGG | 1.628823 ATAGACCGAGATGCCAGACG | GGTCACCATGTTGGGAGACG | 1.701889 TAGACCGAGATGCCAGACGG | ACGGAAGCGAAGTGTTGAGG | 1.702945 TAGACCGAGATGCCAGACGG | GAGATTCGGGAAGGCATCGG | | | | | |
| NCU00609T0 | 3 | 5 | NCU00609T0\_input.txt | NCU00609T0\_output.txt | 0.214457 TCGTTCTCCCCATCACAAGC | TCGAGGCTAGGAAATGTGCC | 0.215409 TCGTTCTCCCCATCACAAGC | TGAAGGCCGCTGGATTATGG | 0.293428 TCGTTCTCCCCATCACAAGC | AGGCTAGGAAATGTGCCTGG | 0.423227 TCGTTCTCCCCATCACAAGC | GCTGGATTATGGTGCCAAGC | 0.432200 TCACAAGCTTCTCTCCTGGC | AAGTTGTAGAGCGTCGAGGC | | | | | |
| NCU00611T0 | 3 | 5 | NCU00611T0\_input.txt | NCU00611T0\_output.txt | 0.487131 GTCTCAGGACAAGCTGGAGG | CAGTCATGGTTTGGTCACGC | 0.488756 TGGCAAGTCTCAGGACAAGC | CAGTCATGGTTTGGTCACGC | 0.687279 CATGGCGTCATGTTCAGTGG | CAGTCATGGTTTGGTCACGC | 0.788828 GTCTCAGGACAAGCTGGAGG | TTGTAGATCTGGTTCCGCCC | 1.024555 ATCTCGCATTCGTCCAGACC | ACGCGACTCATTATTGACGC | | | | | |
| NCU00614T0 | 2 | 5 | NCU00614T0\_input.txt | NCU00614T0\_output.txt | 0.797621 GCTTGGTGTTCGCTTATGGC | TTCAGTGGGAGCCATACACC | 0.859123 GCTTGGTGTTCGCTTATGGC | GTGGGAGCCATACACCAACC | 0.923162 GGTGTTCGCTTATGGCATCG | TTCAGTGGGAGCCATACACC | 0.984665 GGTGTTCGCTTATGGCATCG | GTGGGAGCCATACACCAACC | 1.069908 CGGCAGTTGTGGAGAGTACC | GTGGGAGCCATACACCAACC | | | | | |
| NCU00616T0 | 3 | 5 | NCU00616T0\_input.txt | NCU00616T0\_output.txt | 0.278127 CTCGTTACTCCTCGCAGACC | ACAGCACTGACTTGGTCACC | 0.278165 CTCGTTACTCCTCGCAGACC | ATAACCACGGACTGCGATGG | 0.287698 CTGTCGGTAGTTGAGGCTGG | ATAACCACGGACTGCGATGG | 0.349607 TTCAGCTCGTTACTCCTCGC | ACAGCACTGACTTGGTCACC | 0.349645 TTCAGCTCGTTACTCCTCGC | ATAACCACGGACTGCGATGG | | | | | |
| NCU00616T1 | 2 | 5 | NCU00616T1\_input.txt | NCU00616T1\_output.txt | 0.278127 CTCGTTACTCCTCGCAGACC | ACAGCACTGACTTGGTCACC | 0.278165 CTCGTTACTCCTCGCAGACC | ATAACCACGGACTGCGATGG | 0.287698 CTGTCGGTAGTTGAGGCTGG | ATAACCACGGACTGCGATGG | 0.349607 TTCAGCTCGTTACTCCTCGC | ACAGCACTGACTTGGTCACC | 0.349645 TTCAGCTCGTTACTCCTCGC | ATAACCACGGACTGCGATGG | | | | | |
| NCU00616T2 | 2 | 5 | NCU00616T2\_input.txt | NCU00616T2\_output.txt | 0.278127 CTCGTTACTCCTCGCAGACC | ACAGCACTGACTTGGTCACC | 0.278165 CTCGTTACTCCTCGCAGACC | ATAACCACGGACTGCGATGG | 0.287698 CTGTCGGTAGTTGAGGCTGG | ATAACCACGGACTGCGATGG | 0.349607 TTCAGCTCGTTACTCCTCGC | ACAGCACTGACTTGGTCACC | 0.349645 TTCAGCTCGTTACTCCTCGC | ATAACCACGGACTGCGATGG | | | | | |
| NCU00617T0 | 2 | 5 | NCU00617T0\_input.txt | NCU00617T0\_output.txt | 0.218672 CTATCGACGACCCTAACGCC | TCTGCTGGCGGTAAATCTCC | 0.218672 CCTATCGACGACCCTAACGC | TCTGCTGGCGGTAAATCTCC | 0.219654 CTATCGACGACCCTAACGCC | CTCCTTCATCCTCTGCTGGC | 0.219654 CTATCGACGACCCTAACGCC | GCTCCTTCATCCTCTGCTGG | 0.219654 CCTATCGACGACCCTAACGC | CTCCTTCATCCTCTGCTGGC | | | | | |
| NCU00618T0 | 3 | 5 | NCU00618T0\_input.txt | NCU00618T0\_output.txt | 0.204659 ATATCGCCAGGGATCGATGC | CGCACCGCAATGTTATCTGG | 0.425300 ATATCGCCAGGGATCGATGC | GCACCGCAATGTTATCTGGC | 0.481369 GGCATACCTTGAGCTTGTGC | CGCACCGCAATGTTATCTGG | 0.626541 AGTATCATGTCGCGGCTAGG | CGCACCGCAATGTTATCTGG | 0.702010 GGCATACCTTGAGCTTGTGC | GCACCGCAATGTTATCTGGC | | | | | |
| NCU00619T0 | 1 | 5 | NCU00619T0\_input.txt | NCU00619T0\_output.txt | 0.139784 ATCTTGCCGGTCTTGTAGCC | TCAAGACGACACAGACCAGC | 0.421521 ACTTGATCGGATAGTGGGCG | TCAAGACGACACAGACCAGC | 0.498194 ACTTGATCGGATAGTGGGCG | TTCCGAACAGCGTCAGATCC | 0.647640 ATCTTGCCGGTCTTGTAGCC | TCCGCTAAATGGTCCAGTCC | 0.650666 AGGACTGGACCATTTAGCGG | TTCCGAACAGCGTCAGATCC | | | | | |
| NCU00620T0 | 2 | 5 | NCU00620T0\_input.txt | NCU00620T0\_output.txt | 0.140525 TTCGAGGACCAAGACGATGC | GGTAGTAGCCACGGGAATCG | 0.283877 TTCGAGGACCAAGACGATGC | GTCTTGGTAGTAGCCACGGG | 0.287949 TTCGAGGACCAAGACGATGC | CGTCTTGGTAGTAGCCACGG | 0.287949 TTCGAGGACCAAGACGATGC | CCGTCTTGGTAGTAGCCACG | 0.419969 TTCGAGGACCAAGACGATGC | ATCATTCGAGAGGTACGGCG | | | | | |
| NCU00621T0 | 3 | 5 | NCU00621T0\_input.txt | NCU00621T0\_output.txt | 0.201200 CAAGGACGTACTCAGGACGG | CGTTCCCACAAGCAAACAGG | 0.201200 CCAAGGACGTACTCAGGACG | CGTTCCCACAAGCAAACAGG | 0.212009 TATTCGTATGGACCAGCCGC | TAGCAGAAATAGGCGTGCCC | 0.212049 TATTCGTATGGACCAGCCGC | GAAAGCTGGAGTAGGGCTCG | 0.212186 TATTCGTATGGACCAGCCGC | GGAGAAAGCTGGAGTAGGGC | | | | | |
| NCU00622T0 | 4 | 5 | NCU00622T0\_input.txt | NCU00622T0\_output.txt | 2.332861 GTATGGCCATACCTACCCGC | TCGATAATCTGACGTGGATGGG | 2.577186 GTATGGCCATACCTACCCGC | ACTATGCCATGAACGAAGACG | 2.647150 GTATGGCCATACCTACCCGC | CGATAATCTGACGTGGATGGGG | 2.713870 GTATGGCCATACCTACCCGC | CGATAATCTGACGTGGATGGG | 2.780873 GTATGGCCATACCTACCCGC | AGACGTCTTGATGTTTGATGGC | | | | | |
| NCU00623T0 | 3 | 5 | NCU00623T0\_input.txt | NCU00623T0\_output.txt | 0.147998 TCTGGTGATTGACTGGGTGC | CTTTGTTGAGACGCGGTTCG | 0.417110 TCTGGTGATTGACTGGGTGC | TGTTGCCTTTGTTGAGACGC | 0.495448 TCTGGTGATTGACTGGGTGC | GAGAGCCTTTTCGTCGGAGG | 0.497379 GTGTGATCTCTACGCCTCCC | CTTTGTTGAGACGCGGTTCG | 0.497379 GGTGTGATCTCTACGCCTCC | CTTTGTTGAGACGCGGTTCG | | | | | |
| NCU00624T0 | 3 | 5 | NCU00624T0\_input.txt | NCU00624T0\_output.txt | 0.144148 ATCGGCTGGACTGTCTTTGG | ATCTCTTTGCCCGACCTTCG | 0.215134 ATCGGCTGGACTGTCTTTGG | GAGGAACTGTAGCCTCTGCG | 0.287267 GATTGTGAATGGCGGCAAGG | ATCTCTTTGCCCGACCTTCG | 0.352699 ATCGGCTGGACTGTCTTTGG | GGCGTTGTTGTTGTTCTCGG | 0.352699 ATCGGCTGGACTGTCTTTGG | GGGCGTTGTTGTTGTTCTCG | | | | | |
| NCU00627T0 | 5 | 5 | NCU00627T0\_input.txt | NCU00627T0\_output.txt | 0.415322 GATGTCGATCTCGGTCTCGG | GTCATCCAGAACCTCGGACG | 0.483923 GATGTCGATCTCGGTCTCGG | ACGAGCAGTCATCCAGAACC | 0.556958 GATGTCGATCTCGGTCTCGG | AGGCAGCTCAGGTTTGTACG | 0.621833 GTCGATTTCGGTGTCCTTGC | GTCATCCAGAACCTCGGACG | 0.690435 GTCGATTTCGGTGTCCTTGC | ACGAGCAGTCATCCAGAACC | | | | | |
| NCU00628T0 | 1 | 5 | NCU00628T0\_input.txt | NCU00628T0\_output.txt | 0.273463 CGGTTGGTTTGCAAGACTGG | TAGCGACAAGTCCTTGACCG | 0.281152 CGGTTGGTTTGCAAGACTGG | AAGTCCTTGACCGATCCAGC | 0.631850 GGTTTGCAAGACTGGTTGGG | TAGCGACAAGTCCTTGACCG | 0.639538 GGTTTGCAAGACTGGTTGGG | AAGTCCTTGACCGATCCAGC | 0.778242 ATCGGTCAAGGACTTGTCGC | CAAGTAGGCCGGCTAGTACC | | | | | |
| NCU00629T0 | 3 | 5 | NCU00629T0\_input.txt | NCU00629T0\_output.txt | 0.216612 ATGAGGACGATGGTCTTGCG | CTGCTCCTGCTCCATATCCG | 0.288822 ATGAGGACGATGGTCTTGCG | TACCATAAGGCGGAATGGGC | 0.356041 CTCAGCGATGAGGACGATGG | CTGCTCCTGCTCCATATCCG | 0.360982 GGGTAGTGATGCGGGTATCG | TTCGGGGCTGTTGAGATTGG | 0.428252 CTCAGCGATGAGGACGATGG | TACCATAAGGCGGAATGGGC | | | | | |
| NCU00630T0 | 4 | 5 | NCU00630T0\_input.txt | NCU00630T0\_output.txt | 0.139240 TTCACCGTCTTCACACCTCG | GAATGAAGACGAGGGGGTGG | 0.276663 GACGTTCCAGGAAGCAATGC | GAATGAAGACGAGGGGGTGG | 0.344628 TTCACCGTCTTCACACCTCG | GCTGATGCCGATGGATTTGG | 0.369645 TACTACCTGGAGCTGGGAGG | GAATGAAGACGAGGGGGTGG | 0.429307 ACCATGACGTTCCAGGAAGC | GAATGAAGACGAGGGGGTGG | | | | | |
| NCU00631T0 | 2 | 5 | NCU00631T0\_input.txt | NCU00631T0\_output.txt | 0.344665 GACAACGGCTTCGACTTTGG | CTTGTGTTGTGCGGTATGCC | 0.488038 CGACTTTGGCCCATTGATCG | CTTGTGTTGTGCGGTATGCC | 0.501708 TAACGGCATTGACCCCTTCG | CTTGTGTTGTGCGGTATGCC | 0.558321 CTTCAATGCCAGCACCTTCG | CGAGGTTGGAGTCAAGGTCG | 0.613541 CTTCAATGCCAGCACCTTCG | ATGTTGAAGTAGCTTGCGCG | | | | | |
| NCU00632T0 | 2 | 5 | NCU00632T0\_input.txt | NCU00632T0\_output.txt | 0.500007 CAAGGAGTCGTGTACCACCC | TTGTCCCGTTATTAGGCGCC | 0.933068 TAGCCGGCTCGAAGTTTTCC | GAGGTGGTAAGAGGGGAAGC | 1.039692 CAAGGAGTCGTGTACCACCC | GTCATTATGGAGCCCTACCGG | 1.132027 GCAAGGAGTCGTGTACCACC | TTGTCCCGTTATTAGGCGCC | 1.182100 CAAGGAGTCGTGTACCACCC | TGTCCCGTTATTAGGCGCC | | | | | |
| NCU00633T0 | 1 | 5 | NCU00633T0\_input.txt | NCU00633T0\_output.txt | 0.130204 GATGAGGAGACAGCGATGGG | CCGTATTCTTGTGCTTGCCG | 0.130204 GGATGAGGAGACAGCGATGG | CCGTATTCTTGTGCTTGCCG | 0.212391 GATGAGGAGACAGCGATGGG | GTATTCTTGTGCTTGCCGCC | 0.212391 GGATGAGGAGACAGCGATGG | GTATTCTTGTGCTTGCCGCC | 0.280413 GATGAGGAGACAGCGATGGG | TAACTACCCGGTCAAAGCGG | | | | | |
| NCU00634T0 | 3 | 5 | NCU00634T0\_input.txt | NCU00634T0\_output.txt | 0.921997 AACCGATCTGGATATCCGCC | GAGTTACGCCCGGTAGATCC | 0.976617 TCCGCCATTTTTCTTGTCGG | TCGGCATCGTGAGTAGTTCG | 1.191774 TCCGCCATTTTTCTTGTCGG | GAGTTACGCCCGGTAGATCC | 1.311799 CTCGGTTCAAACATACGCGC | GCAGAAATCTCCAACATCGGC | 1.311799 GCTCGGTTCAAACATACGCG | GCAGAAATCTCCAACATCGGC | | | | | |
| NCU00635T0 | 4 | 5 | NCU00635T0\_input.txt | NCU00635T0\_output.txt | 1.199909 CGGGCAAGCTCACATGTAGG | CCCTACCTTCTTGACAGCCG | 1.412145 CGGGCAAGCTCACATGTAGG | TACCTTCTTGACAGCCGTGG | 1.605754 GGTTAGTCTTTGAACCGGCG | CCACGGGAAATTGGAAGAGC | 1.613888 CTTAGCCTCACCGAAGACGC | CCACGGGAAATTGGAAGAGC | 1.620666 TCTTAGCCTCACCGAAGACG | CCACGGGAAATTGGAAGAGC | | | | | |
| NCU00636T0 | 4 | 5 | NCU00636T0\_input.txt | NCU00636T0\_output.txt | 0.491130 GGAGGTCTTCTCGTCGATGG | CACTCGCAGTCTACCCTTGG | 0.499877 CTTGTAGCCAGGAACGGACC | CACTCGCAGTCTACCCTTGG | 0.564836 TCTTCTCGTCGATGGAAGGC | CACTCGCAGTCTACCCTTGG | 0.565609 GCTTGGAGGTCTTCTCGTCG | CACTCGCAGTCTACCCTTGG | 0.699995 GGAGGTCTTCTCGTCGATGG | ACAACACTCGCAGTCTACCC | | | | | |
| NCU00636T1 | 5 | 5 | NCU00636T1\_input.txt | NCU00636T1\_output.txt | 0.144625 ATGGAAGCGTTGACTGAGGG | CACTCGCAGTCTACCCTTGG | 0.353489 ATGGAAGCGTTGACTGAGGG | ACAACACTCGCAGTCTACCC | 0.772335 ATGGAAGCGTTGACTGAGGG | GTGAGTTCACATCGACCCGG | 0.780781 ATGGAAGCGTTGACTGAGGG | GACCCGGGTCTGAATGAAGC | 0.935730 ATGGAAGCGTTGACTGAGGG | ACCATGCCATTGCTTCTTGG | | | | | |
| NCU00638T0 | 1 | 5 | NCU00638T0\_input.txt | NCU00638T0\_output.txt | 0.145743 TGCATCGATCAAGGGGTAGC | TTCCAGGTTCCCATTTCCGG | 0.146619 TTATTGGGAACGGGGTTGGG | TGCTACCCCTTGATCGATGC | 0.147614 TTATTGGGAACGGGGTTGGG | TGCTTGCTACCCCTTGATCG | 0.147886 GCATTGGCGTTGGGTTTAGG | TTCCAGGTTCCCATTTCCGG | 0.217103 TCGATCAAGGGGTAGCAAGC | TTCCAGGTTCCCATTTCCGG | | | | | |
| NCU00640T0 | 2 | 5 | NCU00640T0\_input.txt | NCU00640T0\_output.txt | 0.356878 ACTCAGTCCTGTTTCCACGC | GTTCTCCCTCCCATTGCTCC | 0.848188 GGGCGCAACTCTCACTAAGG | GTTCTCCCTCCCATTGCTCC | 0.849052 GGGCGCAACTCTCACTAAGG | TTTGGTTCCTGAGTCACCCG | 1.058007 CTCAGTCCTGTTTCCACGCC | GTTCTCCCTCCCATTGCTCC | 1.067331 TCCACGCCAATTTGGAGAGC | GTTCTCCCTCCCATTGCTCC | | | | | |
| NCU00641T0 | 2 | 5 | NCU00641T0\_input.txt | NCU00641T0\_output.txt | 0.201032 GCTACGAGAAGTACGACCGG | AGTTGTCGAGATCCTTCGCC | 0.280602 CGGCTCCTTCATCCTTACCC | AGTTGTCGAGATCCTTCGCC | 0.280602 CCGGCTCCTTCATCCTTACC | AGTTGTCGAGATCCTTCGCC | 0.352753 CTTCATCCTTACCCCTGCCG | AGTTGTCGAGATCCTTCGCC | 0.357439 CCTTCATCCTTACCCCTGCC | AGTTGTCGAGATCCTTCGCC | | | | | |
| NCU00642T0 | 7 | 5 | NCU00642T0\_input.txt | NCU00642T0\_output.txt | 0.343205 ACAGTGGAAGACGAACTCCG | GTTGTGGTGTTGAAGTCGGC | 0.715175 AAGTGGACATCAACAGGCCC | GGTGCCCAGTAGTGGTATGC | 0.975322 ACAGTGGAAGACGAACTCCG | GGTTGTGGTGTTGAAGTCGG | 0.998036 TGGTGGTGAAGGAGAAACGC | GGTGCCCAGTAGTGGTATGC | 1.006420 AAGTGGACATCAACAGGCCC | TGGAAGCCGATCTGATGAGG | | | | | |
| NCU00643T0 | 2 | 5 | NCU00643T0\_input.txt | NCU00643T0\_output.txt | 0.284040 CTGCCTAGACCTTTAGCCGG | AAAAGGGTTCACGGGTCTGG | 0.427662 AATGTCGCGATGGAGTCTCG | AAAAGGGTTCACGGGTCTGG | 0.499801 CTGCCTAGACCTTTAGCCGG | TGCCATCAAGGTGCAGATCC | 0.500299 TTAAGTTCCTGACTGCCCGC | AAAAGGGTTCACGGGTCTGG | 0.505633 CTGCCTAGACCTTTAGCCGG | AGAGGAAAAGGGTTCACGGG | | | | | |
| NCU00644T0 | 3 | 5 | NCU00644T0\_input.txt | NCU00644T0\_output.txt | 2.316346 AGATGACGAAGGGGGTCTGG | CGGAGAGGTCACAAAAGGAGG | 2.322910 AGATGACGAAGGGGGTCTGG | AGACGGAGAGGTCACAAAAGG | 2.492158 GAAGACAATGCGGCCAAGC | CGGAGAGGTCACAAAAGGAGG | 2.498722 GAAGACAATGCGGCCAAGC | AGACGGAGAGGTCACAAAAGG | 2.588184 AGATGACGAAGGGGGTCTGG | ACCGAACCCGATTTTTCAACC | | | | | |
| NCU00645T0 | 5 | 5 | NCU00645T0\_input.txt | NCU00645T0\_output.txt | 0.287937 GTCGAGGTTACGGACAAGGG | TTGGATGATTCCGGATGGCC | 0.349554 CCTGTGTCTCGACGGTAAGG | TTGGATGATTCCGGATGGCC | 0.559016 GACCTCGGACTACTTACGGC | TTGGATGATTCCGGATGGCC | 0.635304 CTGTGTCTCGACGGTAAGGC | TTGGATGATTCCGGATGGCC | 0.636761 GTCGAGGTTACGGACAAGGG | GTCGTCGTATTCTGCCCAGG | | | | | |
| NCU00646T1 | 8 | 5 | NCU00646T1\_input.txt | NCU00646T1\_output.txt | 0.139570 CTCGGTTATACCCCAGACGC | AATGACCTCCCTTAACGCCG | 0.277261 CTCGGTTATACCCCAGACGC | CCAAAGTGGCTTCATGCTGG | 0.423382 CTCGGTTATACCCCAGACGC | TTCATGCTGGTCTTCTCGCC | 0.548429 GCTCATGAACGCGAGATTGG | CCAAAGTGGCTTCATGCTGG | 0.568835 GAGGAACCTCATCAGCCACG | AATGACCTCCCTTAACGCCG | | | | | |
| NCU00646T0 | 8 | 5 | NCU00646T0\_input.txt | NCU00646T0\_output.txt | 0.139570 CTCGGTTATACCCCAGACGC | AATGACCTCCCTTAACGCCG | 0.277261 CTCGGTTATACCCCAGACGC | CCAAAGTGGCTTCATGCTGG | 0.423382 CTCGGTTATACCCCAGACGC | TTCATGCTGGTCTTCTCGCC | 0.548429 GCTCATGAACGCGAGATTGG | CCAAAGTGGCTTCATGCTGG | 0.568835 GAGGAACCTCATCAGCCACG | AATGACCTCCCTTAACGCCG | | | | | |
| NCU00648T0 | 6 | 5 | NCU00648T0\_input.txt | NCU00648T0\_output.txt | 0.431121 ACGGTTCTAGCCAACTCACG | GGTTAGGTTGAGGAAGGCGG | 0.431121 ACGGTTCTAGCCAACTCACG | CCGGTTAGGTTGAGGAAGGC | 0.500732 AACTCACGCATCTACTGGGC | GGTTAGGTTGAGGAAGGCGG | 0.500732 AACTCACGCATCTACTGGGC | CCGGTTAGGTTGAGGAAGGC | 0.501960 CTCACTCCTCTGCTGTGTCG | GGTTAGGTTGAGGAAGGCGG | | | | | |
| NCU00649T0 | 2 | 5 | NCU00649T0\_input.txt | NCU00649T0\_output.txt | 0.145424 TGTCATCAAGCTGAGCTCGG | ACTCAAGACTCATGCCGTGG | 0.287472 TGTCATCAAGCTGAGCTCGG | ATCTTGCCGACTCATCACCC | 0.287679 TTGTCCACAAGGCTACTGGC | ACTCAAGACTCATGCCGTGG | 0.429727 TTGTCCACAAGGCTACTGGC | ATCTTGCCGACTCATCACCC | 0.478745 TGTCATCAAGCTGAGCTCGG | CGCTCCAATCCTTTTCGACG | | | | | |
| NCU00650T0 | 2 | 5 | NCU00650T0\_input.txt | NCU00650T0\_output.txt | 0.281873 GCACTCAAACATCGATGCGG | AAGAAGACACCGAGCACTGG | 0.351328 CAAACATCGATGCGGATGCC | AAGAAGACACCGAGCACTGG | 0.425287 AAAACACCAAACATCGCCCC | AAGAAGACACCGAGCACTGG | 0.502562 GCACTCAAACATCGATGCGG | TACACCATGCTACCTTGGGC | 0.572017 CAAACATCGATGCGGATGCC | TACACCATGCTACCTTGGGC | | | | | |
| NCU00651T0 | 4 | 5 | NCU00651T0\_input.txt | NCU00651T0\_output.txt | 0.781925 TTGCTGGTCGTCTATCTCGC | GCTTTTGGGCTGGAGTAGGG | 0.781925 TTGCTGGTCGTCTATCTCGC | GGCTTTTGGGCTGGAGTAGG | 0.936623 TTGCTGGTCGTCTATCTCGC | ACATCTGATGAGTGAGGGCC | 0.998679 CTGGTCGTCTATCTCGCTGC | GCTTTTGGGCTGGAGTAGGG | 0.998679 CTGGTCGTCTATCTCGCTGC | GGCTTTTGGGCTGGAGTAGG | | | | | |
| NCU00651T1 | 4 | 5 | NCU00651T1\_input.txt | NCU00651T1\_output.txt | 0.134048 TTGCTGGTCGTCTATCTCGC | ATGGGTTGGAAAAGTTGCGC | 0.350802 CTGGTCGTCTATCTCGCTGC | ATGGGTTGGAAAAGTTGCGC | 0.768837 GATTTGGAAGCTCTTCGGCC | ATGGGTTGGAAAAGTTGCGC | 0.781925 TTGCTGGTCGTCTATCTCGC | GCTTTTGGGCTGGAGTAGGG | 0.781925 TTGCTGGTCGTCTATCTCGC | GGCTTTTGGGCTGGAGTAGG | | | | | |
| NCU00651T2 | 4 | 5 | NCU00651T2\_input.txt | NCU00651T2\_output.txt | 0.212490 GCTAGGTGCTTGGAGACTCG | ATGGGTTGGAAAAGTTGCGC | 0.403639 GTCGTAAGCAAGTGATGGCG | ATGGGTTGGAAAAGTTGCGC | 0.403639 CGTCGTAAGCAAGTGATGGC | ATGGGTTGGAAAAGTTGCGC | 0.403639 GCGTCGTAAGCAAGTGATGG | ATGGGTTGGAAAAGTTGCGC | 0.425026 TGCTTGGAGACTCGAATGGC | ATGGGTTGGAAAAGTTGCGC | | | | | |
| NCU00651T3 | 5 | 5 | NCU00651T3\_input.txt | NCU00651T3\_output.txt | 0.143603 CCACGCTTTTCACATGGTCG | ATGGGTTGGAAAAGTTGCGC | 0.352065 TATGCTGTAGATCGCGTGCC | ATGGGTTGGAAAAGTTGCGC | 0.697532 GTGCCACGCTTTTCACATGG | ATGGGTTGGAAAAGTTGCGC | 0.791480 CCACGCTTTTCACATGGTCG | GCTTTTGGGCTGGAGTAGGG | 0.791480 CCACGCTTTTCACATGGTCG | GGCTTTTGGGCTGGAGTAGG | | | | | |
| NCU00652T0 | 3 | 5 | NCU00652T0\_input.txt | NCU00652T0\_output.txt | 0.571521 GTCAAGATGAAGAGGGGGCC | GATAGCTTGGGACCAGGTGC | 0.573139 ATGGTGTATGATCCCGTGGC | GATAGCTTGGGACCAGGTGC | 0.640275 GTCAAGATGAAGAGGGGGCC | TGTCGTCGTTGGAAAAGGGG | 0.646018 GTCAAGATGAAGAGGGGGCC | ACTCTCCTTTCATCCTGCGG | 0.646637 GTCAAGATGAAGAGGGGGCC | AAGGGGTTAAGCAGTGTGCC | | | | | |
| NCU00653T0 | 4 | 5 | NCU00653T0\_input.txt | NCU00653T0\_output.txt | 0.358620 ATATCGGGCGTCCATTCACC | TCTTTCCCAATCTGCCCAGC | 0.697739 CCACATGAGCGTTGTCTTGG | CCGAAATTGCCATTGGAGCC | 0.710413 TGCATCCTGCTCATGCTACG | CCGAAATTGCCATTGGAGCC | 0.781209 CATCCTGCTCATGCTACGGG | CCGAAATTGCCATTGGAGCC | 0.842361 CCACATGAGCGTTGTCTTGG | TCTTTCCCAATCTGCCCAGC | | | | | |
| NCU00654T0 | 1 | 5 | NCU00654T0\_input.txt | NCU00654T0\_output.txt | 0.274474 CAAGTGATGCAGCAGTGTCG | TTACGACAATCACCGGGTCG | 0.410288 CAAGTGATGCAGCAGTGTCG | TCGCCTCTTCAGTTCAGAGC | 0.428221 TCAAGTTGGCTGTCTACGCC | TCCATCTCTTGCTCTGCTGC | 0.491652 TCAAGTTGGCTGTCTACGCC | GCCCGTTTTCCATCTCTTGC | 0.567415 CAAGTGATGCAGCAGTGTCG | AAAGGACAGGAGCTTGGACC | | | | | |
| NCU00655T0 | 2 | 5 | NCU00655T0\_input.txt | NCU00655T0\_output.txt | 2.268617 ACCGAGCCAGTCTACTAGAGG | AGCAGTGATGACGATTGAGGG | 2.427961 ACCGAGCCAGTCTACTAGAGG | GCAGTGATGACGATTGAGGG | 2.458585 ACCTAGCTTCAGACCGAGCC | AGCAGTGATGACGATTGAGGG | 2.470076 CTACCTAGCTTCAGACCGAGC | AGCAGTGATGACGATTGAGGG | 2.525146 TACCTAGCTTCAGACCGAGC | AGCAGTGATGACGATTGAGGG | | | | | |
| NCU00655T1 | 2 | 5 | NCU00655T1\_input.txt | NCU00655T1\_output.txt | 2.268617 ACCGAGCCAGTCTACTAGAGG | AGCAGTGATGACGATTGAGGG | 2.427961 ACCGAGCCAGTCTACTAGAGG | GCAGTGATGACGATTGAGGG | 2.458585 ACCTAGCTTCAGACCGAGCC | AGCAGTGATGACGATTGAGGG | 2.470076 CTACCTAGCTTCAGACCGAGC | AGCAGTGATGACGATTGAGGG | 2.525146 TACCTAGCTTCAGACCGAGC | AGCAGTGATGACGATTGAGGG | | | | | |
| NCU00656T0 | 2 | 5 | NCU00656T0\_input.txt | NCU00656T0\_output.txt | 0.206259 CCGACCAGCTCAACTATCCC | GGACCATTTGCGCTCATTCC | 0.214002 CCGACCAGCTCAACTATCCC | TTGTAGGGTGTTTGGACGGG | 0.275836 CTTGAGGGAGAAGAGACGCC | GGACCATTTGCGCTCATTCC | 0.280289 GGAGAAGAGACGCCCATTCC | GGACCATTTGCGCTCATTCC | 0.283580 CTTGAGGGAGAAGAGACGCC | TTGTAGGGTGTTTGGACGGG | | | | | |
| NCU00657T0 | 2 | 5 | NCU00657T0\_input.txt | NCU00657T0\_output.txt | 0.715474 ACAGTGAGTGGGAGCAAAGC | TGTAGCAATACGGGAAGGCC | 0.916384 ACAGTGAGTGGGAGCAAAGC | TGCCGTTTCTTTGTCAGTGC | 1.035303 TCGACTGTGGTTGAAGACGG | ACTAAGCGGCAACTGACCC | 1.051390 TCGACTGTGGTTGAAGACGG | GACTAAGCGGCAACTGACCC | 1.073194 ACAGTGAGTGGGAGCAAAGC | TCGAGGCTTGACTGGATTCC | | | | | |
| NCU00658T0 | 1 | 5 | NCU00658T0\_input.txt | NCU00658T0\_output.txt | 0.287723 GGTGGAACTATACGCCGACC | AAAGCACGACAGTCCCTACG | 0.635385 GGAACTATACGCCGACCAGC | AAAGCACGACAGTCCCTACG | 0.710536 GGTGGAACTATACGCCGACC | TATGAGCGAACCGGAAAGCC | 0.850508 GGTGGAACTATACGCCGACC | AGAGCAAAGCACGACAGTCC | 1.010166 TAGGGACTGTCGTGCTTTGC | TACCCTTCGGATGGGACTGG | | | | | |
| NCU00659T1 | 3 | 5 | NCU00659T1\_input.txt | NCU00659T1\_output.txt | 2.445208 TAAGAGAAGACGGCTCCTCG | GGTGATGGAGTTGTTAGGGGG | 3.015593 ATAAGAGAAGACGGCTCCTCG | GGTGATGGAGTTGTTAGGGGG | 3.249923 AAGAGAAGACGGCTCCTCG | GGTGATGGAGTTGTTAGGGGG | 3.269946 GGGGTACTTGGAAGGTGTGG | TGTATGAGAGTTTCGGACTGC | 3.271831 TGGAGGGGATTTGAAGGTGC | TGTATGAGAGTTTCGGACTGC | | | | | |
| NCU00659T0 | 2 | 5 | NCU00659T0\_input.txt | NCU00659T0\_output.txt | 5.586985 CGAACGAAGACGATGACAAGG | TGGGCTGCTAATAATATGGACG | 6.078584 CGCAAGAAGGGAAGAGTGG | TGTATGAGAGTTTCGGACTGC | 6.166456 CGAACGAAGACGATGACAAGG | ATGGGCTGCTAATAATATGGACG | 6.169774 CGCAAGAAGGGAAGAGTGG | ACTGCTCTCTATGTCTTATGGGC | 6.383952 CGCAAGAAGGGAAGAGTGG | GGGGTTCTTTTGGTGTTTTTCC | | | | | |
| NCU00660T0 | 2 | 5 | NCU00660T0\_input.txt | NCU00660T0\_output.txt | 0.217300 TGTCAGCTAGAATTGGGCGG | TCGTCGTTAGAGGCGTTTCC | 0.273647 TGTCAGCTAGAATTGGGCGG | GGCGATGTTGTCTTTGACGG | 0.286953 TGTCAGCTAGAATTGGGCGG | CGATGTTGTCTTTGACGGCG | 0.422954 TGTCAGCTAGAATTGGGCGG | GTTGGCAAGGAAGTTGGAGC | 0.560487 TGTCAGCTAGAATTGGGCGG | GCGATGTTGTCTTTGACGGC | | | | | |
| NCU00662T0 | 3 | 5 | NCU00662T0\_input.txt | NCU00662T0\_output.txt | 0.554017 TTCTCCCTCGAAAGATGGCG | CACCATTTCCTCAATCGCGG | 0.554017 TTCTCCCTCGAAAGATGGCG | CCACCATTTCCTCAATCGCG | 0.557330 AAAGATGGCGGAGATCCACG | CACCATTTCCTCAATCGCGG | 0.557330 AAAGATGGCGGAGATCCACG | CCACCATTTCCTCAATCGCG | 0.767648 TCGAAAGATGGCGGAGATCC | CACCATTTCCTCAATCGCGG | | | | | |
| NCU00663T0 | 2 | 5 | NCU00663T0\_input.txt | NCU00663T0\_output.txt | 0.647837 ACCGTGACATTTCCGTCTCC | ATGTTCAACCTCTGGCTGGC | 1.208913 ACCGTGACATTTCCGTCTCC | CATGTTCAACCTCTGGCTGG | 1.265850 ACCGTGACATTTCCGTCTCC | TGTTCAACCTCTGGCTGGC | 1.309147 ACCGTGACATTTCCGTCTCC | TCATGTTCAACCTCTGGCTGG | 1.355216 ACCGTGACATTTCCGTCTCC | CATATCGCCGCTACGAAATCG | | | | | |
| NCU00664T0 | 2 | 5 | NCU00664T0\_input.txt | NCU00664T0\_output.txt | 0.072885 AAGTCTAACTGTGGCCAGCC | ACTGGCGACAAGGATTGAGG | 0.141596 GACGAGGCAAGTAGGATCGG | CAACTGCAATGTCGGTGTCG | 0.147563 AAGTCTAACTGTGGCCAGCC | CAACTGCAATGTCGGTGTCG | 0.568539 CCTGAGACGAGGCAAGTAGG | CAACTGCAATGTCGGTGTCG | 0.646694 GATCGGCTGGGGATTGAAGG | CAACTGCAATGTCGGTGTCG | | | | | |
| NCU00665T0 | 1 | 5 | NCU00665T0\_input.txt | NCU00665T0\_output.txt | 0.144148 TGGCCTGCTAACTGTTAGGC | TGTAACACATCCCGTACCGC | 0.145543 TGGCCTGCTAACTGTTAGGC | GATCCCTCAATCCAGCCAGG | 0.412291 TGGCCTGCTAACTGTTAGGC | CGCCAATGTAACACATCCCG | 0.426569 TGTCGTGCCTAACTGCATCC | TGCCTAACAGTTAGCAGGCC | 0.427099 TGGTTGCGCTGAAGATCTCC | TGCCTAACAGTTAGCAGGCC | | | | | |
| NCU00666T0 | 3 | 5 | NCU00666T0\_input.txt | NCU00666T0\_output.txt | 0.498651 GGCATACGAGTACCGATCCG | TTTTGCACTGCATGATGGCG | 0.791986 AATGGTAGGAAGGCACCTCG | TGTGTGACATTGTCCGTCCC | 0.852611 CCGAATGGTAGGAAGGCACC | TTTTGCACTGCATGATGGCG | 0.930641 AATGGTAGGAAGGCACCTCG | TTTTGCACTGCATGATGGCG | 1.055691 ACGAGTACCGATCCGAATGG | TTTTGCACTGCATGATGGCG | | | | | |
| NCU00667T0 | 6 | 5 | NCU00667T0\_input.txt | NCU00667T0\_output.txt | 0.147321 TGTCGGCATGGTTCTGATCC | TCTTTCTGGGATGGTTGCCC | 0.213326 AGTCGAGGATCTTTGTCGGC | TCTTTCTGGGATGGTTGCCC | 0.412408 TGTCGGCATGGTTCTGATCC | CGTTGTTGGAGCGCATATCC | 0.478413 AGTCGAGGATCTTTGTCGGC | CGTTGTTGGAGCGCATATCC | 0.638420 GTCGGTTCTCACCACAGTGG | TTGGTGTTGAGGATGAGGGC | | | | | |
| NCU00668T0 | 3 | 5 | NCU00668T0\_input.txt | NCU00668T0\_output.txt | 0.626545 CACGCTTCGGTGGTATCTCC | GTACACCCGATGCATTGTGC | 1.456572 TCGATAACACGCTTCGGTGG | CGATGTCTACGCTTGTTGGG | 1.465154 GGGTGACGTTTTGCACATCG | TACGCTTGTTGGGTAGCCG | 1.527223 CACGCTTCGGTGGTATCTCC | CGATGTCTACGCTTGTTGGG | 1.536847 TCGATAACACGCTTCGGTGG | TACGCTTGTTGGGTAGCCG | | | | | |
| NCU00669T0 | 3 | 5 | NCU00669T0\_input.txt | NCU00669T0\_output.txt | 0.627616 CGTTCAACAGGGAATTCGCC | AGAGCATGGAGAACTCGAGC | 0.691674 CGTTCAACAGGGAATTCGCC | CTGGATCTCGTCGTGTTCGG | 0.717381 CCATTTGGAGGAGGAAGGGG | GTCCGGGGTAGTAAAGACGG | 0.717381 CCATTTGGAGGAGGAAGGGG | GGTCCGGGGTAGTAAAGACG | 0.719427 CCATTTGGAGGAGGAAGGGG | AGAGCATGGAGAACTCGAGC | | | | | |
| NCU00670T0 | 3 | 5 | NCU00670T0\_input.txt | NCU00670T0\_output.txt | 0.287065 GCACGAGTAGAAGTAGGCGG | CGGTGATCATAGGTACGGCC | 0.287065 GCACGAGTAGAAGTAGGCGG | GGCGGTGATCATAGGTACGG | 0.287065 GCACGAGTAGAAGTAGGCGG | GGGCGGTGATCATAGGTACG | 0.356193 GCACGAGTAGAAGTAGGCGG | CAAGGAAACCTCTAGGGCGG | 0.419615 CACGAGTAGAAGTAGGCGGG | CGGTGATCATAGGTACGGCC | | | | | |
| NCU00671T0 | 5 | 5 | NCU00671T0\_input.txt | NCU00671T0\_output.txt | 0.494344 TGAACGTGTGGAGGAAGACG | CTCCCGCTGAATCAGGTTCC | 0.794354 ATACGGGCGATGATGATGGG | TGGTGCCTTTCCCATTTCCC | 0.923070 AATACCAAGAGCGAGACGGG | CTCCCGCTGAATCAGGTTCC | 1.129336 GAACGTGTGGAGGAAGACGG | CTCCCGCTGAATCAGGTTCC | 1.134540 CAAGAGAGGCAGGGTGAACG | CTCCCGCTGAATCAGGTTCC | | | | | |
| NCU00673T0 | 2 | 5 | NCU00673T0\_input.txt | NCU00673T0\_output.txt | 0.629037 TCGAACTTGGGAACGACAGC | CAAGGTACCGACGTATCCCG | 0.841221 TCGAACTTGGGAACGACAGC | GCATCTTTGTGCACTCCACC | 0.847580 TCGAACTTGGGAACGACAGC | GACGATCCAAGGTACCGACG | 0.912760 TGCCTTTCGTCGAACTTGGG | CAAGGTACCGACGTATCCCG | 0.971376 TTGCCTTTCGTCGAACTTGG | CAAGGTACCGACGTATCCCG | | | | | |
| NCU00674T0 | 1 | 5 | NCU00674T0\_input.txt | NCU00674T0\_output.txt | 0.150202 GAGCATGGAGATAGTCGGCC | TGTGAAAGGGGAGAAAGGCC | 0.152942 GAGCATGGAGATAGTCGGCC | AAAGGGGAGAAAGGCCTTGG | 0.357663 CTGGTGTGGGAGTAAGCACC | ACCAGCCAAGATCTTCACCG | 0.360696 GAGCATGGAGATAGTCGGCC | GAGAAAGGCCTTGGTCAGGG | 0.423830 ATCGTTGGGTTGCAATTGCG | AGAGTGTGCGGTTTATGGGG | | | | | |
| NCU00675T0 | 2 | 5 | NCU00675T0\_input.txt | NCU00675T0\_output.txt | 0.213188 TCTTGTTGTAGCGGATCCGG | CTTTTCGTTGCATGTGGGGG | 0.213188 TCTTGTTGTAGCGGATCCGG | CCTTTTCGTTGCATGTGGGG | 0.714180 TGGTAGAGTCGGGTCTGACG | CTTTTCGTTGCATGTGGGGG | 0.714180 TGGTAGAGTCGGGTCTGACG | CCTTTTCGTTGCATGTGGGG | 0.848374 CTTGTTGTAGCGGATCCGGG | CTTTTCGTTGCATGTGGGGG | | | | | |
| NCU00676T0 | 1 | 5 | NCU00676T0\_input.txt | NCU00676T0\_output.txt | 0.902442 GGAAGACACGCATGATGTCG | ATACAACCTTCCTTCCCCGC | 1.006309 TGTGAGTGCCACCAACAAGG | ATACAACCTTCCTTCCCCGC | 1.019922 TCAGTTTGGAGGTGGACTGG | ATACAACCTTCCTTCCCCGC | 1.146028 CAGTTTGGAGGTGGACTGGC | ATACAACCTTCCTTCCCCGC | 1.328232 AAGGCGGTCAGTTTGGAGG | ATACAACCTTCCTTCCCCGC | | | | | |
| NCU00677T0 | 3 | 5 | NCU00677T0\_input.txt | NCU00677T0\_output.txt | 1.202813 GCATGTCCAAGGGTTCTTGC | TGATGAAAGCGTTGTTGGGG | 1.203103 GCATGTCCAAGGGTTCTTGC | TGGGAAGAAAATGGGTCGCG | 1.208325 TCTTGCTCTTGTCGATGGGG | TGATGAAAGCGTTGTTGGGG | 1.208616 TCTTGCTCTTGTCGATGGGG | TGGGAAGAAAATGGGTCGCG | 1.210007 TAAGACCGCATGTCCAAGGG | TGATGAAAGCGTTGTTGGGG | | | | | |
| NCU00678T0 | 2 | 5 | NCU00678T0\_input.txt | NCU00678T0\_output.txt | 0.072647 TGTTCCGAAAACGGGAGAGG | GGAAGGAGGAACCAACCAGG | 0.079091 AATGCCGTGTTCCGAAAACG | GGAAGGAGGAACCAACCAGG | 0.142666 TGTTCCGAAAACGGGAGAGG | ATCTTAGCACACCGTTCCCG | 0.149110 AATGCCGTGTTCCGAAAACG | ATCTTAGCACACCGTTCCCG | 0.223243 TGGGGAAGTGGTCAAGTTGG | GGAAGGAGGAACCAACCAGG | | | | | |
| NCU00679T0 | 6 | 5 | NCU00679T0\_input.txt | NCU00679T0\_output.txt | 0.216621 ACCCTTTGAGAAACCCGACC | TGGAATCATACTGGAGCCGC | 0.217369 ACCCTTTGAGAAACCCGACC | AATCATACTGGAGCCGCTGG | 0.356704 TTTGAGAAACCCGACCGTCC | TGGAATCATACTGGAGCCGC | 0.357451 TTTGAGAAACCCGACCGTCC | AATCATACTGGAGCCGCTGG | 0.491714 ACCCTTTGAGAAACCCGACC | CAAGTATGGATTGCCCGTGC | | | | | |
| NCU00680T0 | 2 | 5 | NCU00680T0\_input.txt | NCU00680T0\_output.txt | 2.012630 CCTCTTCTCCCCACATTCGG | TCTGGTCTTCATACTGGCTGG | 2.225423 CTGCCGAAACCTCTTCTCCC | TCTGGTCTTCATACTGGCTGG | 2.273182 CCTCTTCTCCCCACATTCGG | TCGTTCTGGTCTTCATACTGGC | 2.293914 CTTCTCCCCACATTCGGTCG | TCTGGTCTTCATACTGGCTGG | 2.411208 CCTCTTCTCCCCACATTCGG | GGCTGGTGATTCAAAGAAAGGG | | | | | |
| NCU00681T0 | 2 | 5 | NCU00681T0\_input.txt | NCU00681T0\_output.txt | 0.758760 GATAGGGGCTTCCACTACGC | CCGTTCCAGGTTGTTATCGC | 0.780908 TTTACCGGTCAGACTCACGG | ATCCTGGCTGATTCCGTTCC | 0.857187 GGTAACGGCTTCACCTTCCC | ATCCTGGCTGATTCCGTTCC | 0.870762 AGGCCTACCTCTGCTTCTCC | ATCCTGGCTGATTCCGTTCC | 1.008718 CCTACCTCTGCTTCTCCACC | ATCCTGGCTGATTCCGTTCC | | | | | |
| NCU00682T0 | 4 | 5 | NCU00682T0\_input.txt | NCU00682T0\_output.txt | 1.198324 ATAAAGGCCACACGACAGCG | GGACCGGATGATGTCCTTGG | 1.309607 GAATAAAGGCCACACGACAGC | GGACCGGATGATGTCCTTGG | 1.344315 AATAAAGGCCACACGACAGC | GGACCGGATGATGTCCTTGG | 1.412680 ATAAAGGCCACACGACAGCG | ACGAGGGGAATAAAACCGGC | 1.523963 GAATAAAGGCCACACGACAGC | ACGAGGGGAATAAAACCGGC | | | | | |
| NCU00684T1 | 1 | 5 | NCU00684T1\_input.txt | NCU00684T1\_output.txt | 0.346232 GGTGGTTGTTACGCGATTGG | TCCGTACCCCCTTATCCTCG | 0.346327 GGTGGTTGTTACGCGATTGG | CGTAACCATTCCGTACCCCC | 0.412829 GAGTTGGTGGTTGTTACGCG | TCCGTACCCCCTTATCCTCG | 0.412923 GAGTTGGTGGTTGTTACGCG | CGTAACCATTCCGTACCCCC | 0.496813 TGAGATCTGAGGAGGGTGGG | CTGATAGGCTGCCCAAGTGG | | | | | |
| NCU00684T0 | 2 | 5 | NCU00684T0\_input.txt | NCU00684T0\_output.txt | 0.358166 CATCAATGTCGCCGTTTGGG | ATGAAGGAGTAAGCCAGCGG | 0.358233 CATCAATGTCGCCGTTTGGG | TTGTCCCGTATTCATCGGCC | 0.423073 CATCAATGTCGCCGTTTGGG | TCGAGTAGTTGTCCAGCTGC | 0.700803 GCGCAAGGTGTAGGAAAAGG | TTGTCCCGTATTCATCGGCC | 0.712251 CATCAATGTCGCCGTTTGGG | GGCCGATGAAGGAGTAAGCC | | | | | |
| NCU00685T0 | 5 | 5 | NCU00685T0\_input.txt | NCU00685T0\_output.txt | 0.288186 GATCCAGGAAGCCCATGACC | TTGACCAATGCGCAATCTGC | 0.422913 GATCCAGGAAGCCCATGACC | CATAGGACGATACCTGCCGG | 0.422913 GATCCAGGAAGCCCATGACC | CCATAGGACGATACCTGCCG | 0.499380 GATCCAGGAAGCCCATGACC | TCTGCTACTCCAACAACCCG | 0.836846 GATCCAGGAAGCCCATGACC | GATACCATGTGCGAGCAACC | | | | | |
| NCU00685T2 | 4 | 5 | NCU00685T2\_input.txt | NCU00685T2\_output.txt | 0.142727 TATGCGTACCTTGATGGCCC | TCACCGAAAGAACCAGAGCC | 0.215339 TATGCGTACCTTGATGGCCC | AGATGATGTTGGTGCCGAGG | 0.215776 TATGCGTACCTTGATGGCCC | AGAACCAGAGCCGATCTTGC | 0.498434 TATGCGTACCTTGATGGCCC | TGCCGAGGTAAATGTCACCG | 0.500829 TATGCGTACCTTGATGGCCC | ACCGGAGATGATGTTGGTGC | | | | | |
| NCU00685T1 | 5 | 5 | NCU00685T1\_input.txt | NCU00685T1\_output.txt | 0.288186 GATCCAGGAAGCCCATGACC | TTGACCAATGCGCAATCTGC | 0.422913 GATCCAGGAAGCCCATGACC | CATAGGACGATACCTGCCGG | 0.422913 GATCCAGGAAGCCCATGACC | CCATAGGACGATACCTGCCG | 0.499380 GATCCAGGAAGCCCATGACC | TCTGCTACTCCAACAACCCG | 0.836846 GATCCAGGAAGCCCATGACC | GATACCATGTGCGAGCAACC | | | | | |
| NCU00687T0 | 4 | 5 | NCU00687T0\_input.txt | NCU00687T0\_output.txt | 0.210164 TCTGTCATCGAGCGTGATGG | GCATTGTTTCCTCCTGCAGC | 0.562738 AGAAGGTCTGTCATCGAGCG | GCATTGTTTCCTCCTGCAGC | 0.637411 TCATCGAGCGTGATGGAAGC | GCATTGTTTCCTCCTGCAGC | 0.643357 AGCCAGAAGGTCTGTCATCG | GCATTGTTTCCTCCTGCAGC | 1.048463 TCTGTCATCGAGCGTGATGG | CCTCGCACATGATTAAGGGC | | | | | |
| NCU00688T0 | 1 | 5 | NCU00688T0\_input.txt | NCU00688T0\_output.txt | 0.205156 GTTGCGGTTGCTGTTTTTGC | ACATCACCCTTCATCGAGCC | 0.215972 GGAGAACATGGGAGTCGAGC | CACCTTCCTCCAACACCTCC | 0.220187 GGAGAACATGGGAGTCGAGC | ATGACCACCATCCCCAAACC | 0.273891 GTTGCGGTTGCTGTTTTTGC | TCGCAAAACAAACCACGTCG | 0.277497 GTTGCGGTTGCTGTTTTTGC | ATGACAACAACCCCTCAGCC | | | | | |
| NCU00689T0 | 3 | 5 | NCU00689T0\_input.txt | NCU00689T0\_output.txt | 0.907597 TTGAACACGCTGAATCTGCC | TGCCATTTGATCAGGGACCC | 1.102024 CGGCAAGGGTAAAAAGGTTCG | TGCCATTTGATCAGGGACCC | 1.103153 CGGCAAGGGTAAAAAGGTTCG | TACTCAACTCGGTGGTTGGC | 1.104255 CGGCAAGGGTAAAAAGGTTCG | ACTCGGTGGTTGGCAATAGG | 1.157136 ATTCCTCTAGTCGGCAAGGG | TGCCATTTGATCAGGGACCC | | | | | |
| NCU00689T1 | 4 | 5 | NCU00689T1\_input.txt | NCU00689T1\_output.txt | 0.907597 TTGAACACGCTGAATCTGCC | TGCCATTTGATCAGGGACCC | 1.102024 CGGCAAGGGTAAAAAGGTTCG | TGCCATTTGATCAGGGACCC | 1.103153 CGGCAAGGGTAAAAAGGTTCG | TACTCAACTCGGTGGTTGGC | 1.104255 CGGCAAGGGTAAAAAGGTTCG | ACTCGGTGGTTGGCAATAGG | 1.157136 ATTCCTCTAGTCGGCAAGGG | TGCCATTTGATCAGGGACCC | | | | | |
| NCU00690T0 | 1 | 5 | NCU00690T0\_input.txt | NCU00690T0\_output.txt | 0.276830 GGATGTTGCGTGCATCTTGG | CGATTCCAAGAAGCGCAAGG | 0.276889 GCATCTTGGAACTTGACGCG | CGATTCCAAGAAGCGCAAGG | 0.484786 TTGGCGCTCTTGCATTTTCG | CGATTCCAAGAAGCGCAAGG | 0.494724 GGATGTTGCGTGCATCTTGG | CATAGGTGCCTCTAGTCGCC | 0.494724 GGATGTTGCGTGCATCTTGG | CCATAGGTGCCTCTAGTCGC | | | | | |
| NCU00691T0 | 2 | 5 | NCU00691T0\_input.txt | NCU00691T0\_output.txt | 0.566229 AAAGTTGGGAGCTGTGGTGG | TCGACGACTTAGGATGTGCG | 0.717149 AAAGTTGGGAGCTGTGGTGG | TAGGATGTGCGAACTGGTGG | 0.722701 TTCAAGGGGGAGGGAGATGG | TCGACGACTTAGGATGTGCG | 0.811862 TTCAAGGGGGAGGGAGATGG | ATCGAGAAGGAGGAGGAGGG | 0.873620 TTCAAGGGGGAGGGAGATGG | TAGGATGTGCGAACTGGTGG | | | | | |
| NCU00692T0 | 2 | 5 | NCU00692T0\_input.txt | NCU00692T0\_output.txt | 0.203905 GATTCCAAGGTCAGCGTTGC | TGACGAGGAAGTTCTTGGCC | 0.276840 GATTCCAAGGTCAGCGTTGC | TGTTCTTGGGGTTCCTGACG | 0.426556 TGAGCGATTCCAAGGTCAGC | TGACGAGGAAGTTCTTGGCC | 0.492749 TGCACTGTCGACGATAAGGC | TGACGAGGAAGTTCTTGGCC | 0.499492 TGAGCGATTCCAAGGTCAGC | TGTTCTTGGGGTTCCTGACG | | | | | |
| NCU00693T0 | 2 | 5 | NCU00693T0\_input.txt | NCU00693T0\_output.txt | 1.468874 TGGTGGGTTGTTTGCTTTGG | CAAAACGCTGAAGCCACACG | 1.532729 AAAGGTACCTGTGCTGTCGC | CAAAACGCTGAAGCCACACG | 1.894261 TGGTGGGTTGTTTGCTTTGG | AAAACGCTGAAGCCACACG | 1.930779 AGAAAGGTACCTGTGCTGTCG | CAAAACGCTGAAGCCACACG | 1.934742 AAGGTACCTGTGCTGTCGC | CAAAACGCTGAAGCCACACG | | | | | |
| NCU00694T0 | 2 | 5 | NCU00694T0\_input.txt | NCU00694T0\_output.txt | 0.217325 CCAATGCCACCGTGAATTCG | GTCTTGACCAGGGACACTCG | 0.287336 GAGCCCAAGTGATGTACCCC | TAATGTGGCGGTCCAGATGG | 0.287336 GGAGCCCAAGTGATGTACCC | TAATGTGGCGGTCCAGATGG | 0.358365 CCAATGCCACCGTGAATTCG | GCTTGTCGTTAATGTGGCGG | 0.359337 CCAATGCCACCGTGAATTCG | TAATGTGGCGGTCCAGATGG | | | | | |
| NCU00695T0 | 1 | 5 | NCU00695T0\_input.txt | NCU00695T0\_output.txt | 0.128669 CGACGACGACTCAACAATGC | TGCCGTCTTGTCATCAGACC | 0.129163 ACAAGACGGCAGAAAGAGGG | GAGAGCGAACAACATGACGC | 0.145750 CCTCTTCTCCAACTGCTCCG | TGCCGTCTTGTCATCAGACC | 0.185049 CGACGACGACTCAACAATGC | GAGAGCGAACAACATGACGC | 0.195281 CGACGACGACTCAACAATGC | CCCGAGATCTTGACTGAGCC | | | | | |
| NCU00696T0 | 2 | 5 | NCU00696T0\_input.txt | NCU00696T0\_output.txt | 0.437150 GGTGTCAGTAGACCGTCAGC | TGGTGGTAGCATGAGGTTGG | 0.503578 GGTGTCAGTAGACCGTCAGC | GGTGGTAGCATGAGGTTGGG | 0.606835 GTCTATCGCCAGACACCTCG | GCGTCGTCTGTTATGATGCC | 0.606835 CTCGTCTATCGCCAGACACC | GCGTCGTCTGTTATGATGCC | 0.633273 GTCTATCGCCAGACACCTCG | TGGAAAGACTGGCTTCAGCG | | | | | |
| NCU00697T0 | 1 | 5 | NCU00697T0\_input.txt | NCU00697T0\_output.txt | 0.075481 ACACGGCGAAACAATTGAGC | ATCAACTCCCTCAAAGCCGG | 0.147933 ACACGGCGAAACAATTGAGC | ACAGCCATCTTGACATCCGG | 0.214062 GGCACCTCTCATTCATCCCC | ATCAACTCCCTCAAAGCCGG | 0.286515 GGCACCTCTCATTCATCCCC | ACAGCCATCTTGACATCCGG | 0.289237 ATCAGAGTCACATTCGGCCC | TCTGCGCTCAATTGTTTCGC | | | | | |
| NCU00698T0 | 1 | 5 | NCU00698T0\_input.txt | NCU00698T0\_output.txt | 0.145474 AGATGTCGACGACAAGCAGG | TGTCATTCACCCGCTTCTCC | 0.286045 GTAGGGATGAAGCTGAGCCG | TGTCATTCACCCGCTTCTCC | 0.428691 CTCTCTGGTAGAATGGGCGC | ATCCTGCTCCTTCGGATTGC | 0.441974 ACTGATGGGTGGAAGTTGGG | TGTCATTCACCCGCTTCTCC | 0.509299 AGATGTCGACGACAAGCAGG | TGTTCATCTCCACCACCACC | | | | | |
| NCU00699T0 | 5 | 5 | NCU00699T0\_input.txt | NCU00699T0\_output.txt | 0.699663 TTTCTCGGGTTGTTTTCGCC | TCGGAGACTTCCCATTTGCC | 0.772933 TTTCTCGGGTTGTTTTCGCC | CCCATTTGCCATTGTCACCG | 1.261294 TTGTCACCACTTGATGTCGC | TCGGAGACTTCCCATTTGCC | 1.304064 ATTTCTCGGGTTGTTTTCGCC | TCGGAGACTTCCCATTTGCC | 1.334564 TTGTCACCACTTGATGTCGC | CCCATTTGCCATTGTCACCG | | | | | |
| NCU00700T0 | 5 | 5 | NCU00700T0\_input.txt | NCU00700T0\_output.txt | 0.143165 TGCGAGATGGACCACATAGC | AGGTAGCCAGCCTGTTTTCC | 0.349033 TGCGAGATGGACCACATAGC | GCGCTCATACCTAGGTAGCC | 0.408583 CATAGACGACAACCTTGCGC | AGGTAGCCAGCCTGTTTTCC | 0.408583 GCATAGACGACAACCTTGCG | AGGTAGCCAGCCTGTTTTCC | 0.429573 AAACTTCTCGATCGGGGTGC | AGGTAGCCAGCCTGTTTTCC | | | | | |
| NCU00700T1 | 4 | 5 | NCU00700T1\_input.txt | NCU00700T1\_output.txt | 0.691453 CGCGTTGTAAAGGACGATGG | AGAAATCAGGGTTGGGTCGC | 0.987915 AGGACGCGTTGTAAAGGACG | AGAAATCAGGGTTGGGTCGC | 1.109644 CGCGTTGTAAAGGACGATGG | CCGATGGAAGGGACAATTGC | 1.326302 CGCGTTGTAAAGGACGATGG | ATCAACTTGGTCTGCTGCCG | 1.395625 CGCGTTGTAAAGGACGATGG | GGTGCGGACAGAAATCAGGG | | | | | |
| NCU00701T0 | 2 | 5 | NCU00701T0\_input.txt | NCU00701T0\_output.txt | 0.852571 GAAGTCCTTCGTCCTCACGG | GGGTCGATGTAGTTGGTGCC | 0.852571 GAAGTCCTTCGTCCTCACGG | GGGGTCGATGTAGTTGGTGC | 1.108632 GAAGTCCTTCGTCCTCACGG | CGATGTAGTTGGTGCCTTCG | 1.138244 CCAGGGCTTTGACATCTCCC | GGGTCGATGTAGTTGGTGCC | 1.138244 CCAGGGCTTTGACATCTCCC | GGGGTCGATGTAGTTGGTGC | | | | | |
| NCU00704T0 | 3 | 5 | NCU00704T0\_input.txt | NCU00704T0\_output.txt | 0.215939 GAATGGTGACCGGACCTAGC | GACCACCTCTTCACTCAGCC | 0.215982 GACCTAGCGTGCTGAAGAGG | GACCACCTCTTCACTCAGCC | 0.428201 GAATGGTGACCGGACCTAGC | TCCAGCTATGAACCACACCG | 0.428244 GACCTAGCGTGCTGAAGAGG | TCCAGCTATGAACCACACCG | 0.765400 GAATGGTGACCGGACCTAGC | TTCTGCAACGTAGCACAAGC | | | | | |
| NCU00705T0 | 2 | 5 | NCU00705T0\_input.txt | NCU00705T0\_output.txt | 0.631264 CGGTGTTCGCATGGTTTACC | CTTGGAGCATCTGAGCCTGG | 0.840981 CACGACCATTTCAACGACGG | GACTGTGGGATGACTTGGGC | 0.847086 CACGACCATTTCAACGACGG | AATGGTGATTTCAGGGCCGG | 0.855408 CACGACCATTTCAACGACGG | TGACTGTGGGATGACTTGGG | 0.894349 CACGACCATTTCAACGACGG | ACGGTCAGAAGTCGATACGG | | | | | |
| NCU00706T0 | 3 | 5 | NCU00706T0\_input.txt | NCU00706T0\_output.txt | 0.689903 GCTTCTTCTGCTTGCAGACG | GTCTCGAGACGTGCTGATGG | 0.714119 TGCCGTGTCTTCTTGATCCC | TAGCTGTTCCATGTGCTGCC | 0.988043 GCTTCTTCTGCTTGCAGACG | GATGGCCAAGGAGCTCATGG | 1.101490 TGCCGTGTCTTCTTGATCCC | CATAGCTGTTCCATGTGCTGC | 1.121464 AAGCTGCTTCTTCTGCTTGC | GTCTCGAGACGTGCTGATGG | | | | | |
| NCU00707T0 | 2 | 5 | NCU00707T0\_input.txt | NCU00707T0\_output.txt | 0.203870 TATGACTGAGAGGCCAACGC | CGCGGAGATGCTTAAAAGGC | 0.213926 TATGACTGAGAGGCCAACGC | TGCTTCCACTGAATCCACCC | 0.557918 TATGACTGAGAGGCCAACGC | GCGGAGATGCTTAAAAGGCC | 0.562618 TATGACTGAGAGGCCAACGC | TTCTTCTTCTCCGTGACGCG | 0.570659 TATGACTGAGAGGCCAACGC | CCCCTGCTTCCACTGAATCC | | | | | |
| NCU00708T0 | 2 | 5 | NCU00708T0\_input.txt | NCU00708T0\_output.txt | 0.506001 AGGAGAAGATGGAGACGGGG | TCTGCTGTCATTACCTGGCG | 0.734034 ATGGGTTGTGAGGCTTAGGG | TCTGCTGTCATTACCTGGCG | 0.858127 AGGAGAAGATGGAGACGGGG | AAAGTCGGGATAGCAGTCGG | 0.926481 GATGGAGACGGGGATATCGG | TCTGCTGTCATTACCTGGCG | 1.086160 ATGGGTTGTGAGGCTTAGGG | AAAGTCGGGATAGCAGTCGG | | | | | |
| NCU00709T0 | 3 | 5 | NCU00709T0\_input.txt | NCU00709T0\_output.txt | 0.291339 GAAGTGGCGTAGGAGAAGGG | AGAGAGGGGATATGGACGGG | 0.365863 AGGTCATCGTCAAAGGAGGC | AGAGAGGGGATATGGACGGG | 0.490803 TTGTGTTGAACTTGACGCCG | AGAGAGGGGATATGGACGGG | 0.713360 GAAGTGGCGTAGGAGAAGGG | GGAGGTGGTGGTTGAGTTGG | 0.858581 TGACCACCACGCATACTTGG | GGAGGTGGTGGTTGAGTTGG | | | | | |
| NCU00710T0 | 3 | 5 | NCU00710T0\_input.txt | NCU00710T0\_output.txt | 0.356249 TCACTAACAGAGGCTGCTGC | GGTAGGTAGCGGCCATAACG | 0.424890 CTGGGATGTCTCATCACCGG | GGTAGGTAGCGGCCATAACG | 0.569250 CAGAGGCTGCTGCTGATAGG | GGTAGGTAGCGGCCATAACG | 0.793022 TCACTAACAGAGGCTGCTGC | AAGAGCTCCGGGTAGGTAGC | 0.835071 CTGGGATGTCTCATCACCGG | CACTGACGCCAGAGTACACG | | | | | |
| NCU00711T0 | 3 | 5 | NCU00711T0\_input.txt | NCU00711T0\_output.txt | 0.150346 GGGCTTTGGGCTTTTCATCG | AGAGGGGCTTGGAAATTGGG | 0.282629 GTCATGACGTTGGGCTTTGG | AGAGGGGCTTGGAAATTGGG | 0.357364 GGGCTTTGGGCTTTTCATCG | TTTTGAAAGACAACGCCGCC | 0.489648 GTCATGACGTTGGGCTTTGG | TTTTGAAAGACAACGCCGCC | 0.498719 TCGGGAGTGTACGTATTGGC | AGAGGGGCTTGGAAATTGGG | | | | | |
| NCU00712T0 | 3 | 5 | NCU00712T0\_input.txt | NCU00712T0\_output.txt | 0.287994 CAAGACCGACGTCGATACCC | GCATCGAGACGGAGATCTGG | 0.295508 TTGCTACTGGCCAAGATCCC | GCATCGAGACGGAGATCTGG | 0.427419 CAAGACCGACGTCGATACCC | TCGAGACGGAGATCTGGAGG | 0.427449 CAAGACCGACGTCGATACCC | AATGGAAGGCATCGAGACGG | 0.434963 TTGCTACTGGCCAAGATCCC | AATGGAAGGCATCGAGACGG | | | | | |
| NCU00713T0 | 2 | 5 | NCU00713T0\_input.txt | NCU00713T0\_output.txt | 0.795335 CAAACGGTCAATGCACGAGG | TGCCTAGTATTCGGCATCCC | 0.850176 ACAACTAATCGGCGCAAACG | TGCCTAGTATTCGGCATCCC | 0.859486 CACTGGACCTCACTCATCGG | TGCCTAGTATTCGGCATCCC | 0.859486 CCACTGGACCTCACTCATCG | TGCCTAGTATTCGGCATCCC | 0.934419 ATCGGCGCACACTATGTACC | TGCCTAGTATTCGGCATCCC | | | | | |
| NCU00714T0 | 3 | 5 | NCU00714T0\_input.txt | NCU00714T0\_output.txt | 0.352922 CTTCAACGCCATGTATGCGG | ATCTTGGTCCTGACGTTGGG | 0.352922 CCTTCAACGCCATGTATGCG | ATCTTGGTCCTGACGTTGGG | 0.432743 TGAGATTGAGCAGCTGGAGC | ATCTTGGTCCTGACGTTGGG | 0.433415 TCGTGAGATTGAGCAGCTGG | ATCTTGGTCCTGACGTTGGG | 0.573563 GCCTTCAACGCCATGTATGC | ATCTTGGTCCTGACGTTGGG | | | | | |
| NCU00715T0 | 3 | 5 | NCU00715T0\_input.txt | NCU00715T0\_output.txt | 0.841386 TTACTGGGCAACGATCTCGC | AAACAAAGCTCACAGGCACG | 0.846423 TTACTGGGCAACGATCTCGC | AGCAAACAAAGCTCACAGGC | 1.379295 TTACTGGGCAACGATCTCGC | AACCCTCCTTACCCTCATGC | 1.516884 ACTTACTGGGCAACGATCTCG | AAACAAAGCTCACAGGCACG | 1.521921 ACTTACTGGGCAACGATCTCG | AGCAAACAAAGCTCACAGGC | | | | | |
| NCU00716T0 | 3 | 5 | NCU00716T0\_input.txt | NCU00716T0\_output.txt | 0.563987 TTCAACTACCAGCTGAGCGG | GAGGATAGACATACGGGCGG | 0.771928 GCCACGGTTATTGTTTGGGG | GAGGATAGACATACGGGCGG | 1.105935 TTCAACTACCAGCTGAGCGG | CATACGGGCGGCATTTATTGG | 1.193239 CCTCGACTTCACCGTCAACC | GAGGATAGACATACGGGCGG | 1.237577 TTCAACTACCAGCTGAGCGG | CTGAGGATAGACATACGGGCG | | | | | |
| NCU00717T0 | 3 | 5 | NCU00717T0\_input.txt | NCU00717T0\_output.txt | 0.073977 GCCAAGTTCAAGCAGCTTCC | TTTGTACTACACGGCCCACC | 0.283522 GCCAAGTTCAAGCAGCTTCC | GTTTGCCAGGACTCCAATGC | 0.359571 GCCAAGTTCAAGCAGCTTCC | TGCTTTCCCTTGAGACGAGC | 0.412028 TTCACGCTGTGTCTTTGTGC | TTTGTACTACACGGCCCACC | 0.579724 GCCAAGTTCAAGCAGCTTCC | AGGACTCCAATGCAGAGAGC | | | | | |
| NCU00718T0 | 2 | 5 | NCU00718T0\_input.txt | NCU00718T0\_output.txt | 0.363876 CAGAGCATCCGTCCTCTTCC | AAGAGGGGGACATTGGATGC | 0.498836 CAGAGCATCCGTCCTCTTCC | GCTGAAGAGGGGGACATTGG | 0.647687 CAGAGCATCCGTCCTCTTCC | CTGTAAGGCTGAAGAGGGGG | 0.721899 CATCCGTCCTCTTCCAGACC | AAGAGGGGGACATTGGATGC | 0.856860 CATCCGTCCTCTTCCAGACC | GCTGAAGAGGGGGACATTGG | | | | | |
| NCU00719T0 | 1 | 5 | NCU00719T0\_input.txt | NCU00719T0\_output.txt | 0.148059 ATAGCTGTCGCCCAGATTGG | GTCAGACTTGCTGCTTGTGC | 0.217374 ATAGCTGTCGCCCAGATTGG | GATCCAAAACGCCGTTGACC | 0.718445 GCATACCTACACCTACGGGG | GTCAGACTTGCTGCTTGTGC | 0.721525 ATGGGAAGCATCAACGGTGG | GTCAGACTTGCTGCTTGTGC | 0.787760 GCATACCTACACCTACGGGG | GATCCAAAACGCCGTTGACC | | | | | |
| NCU00720T0 | 2 | 5 | NCU00720T0\_input.txt | NCU00720T0\_output.txt | 0.277763 GCGTTCGTTCAAGTCCAACC | GTCGATGAGGACGATCTCGG | 0.415174 TAGACCATCTCCTCCCGAGC | GTCGATGAGGACGATCTCGG | 0.462988 CAAGCACGGAAATCGAGAGC | CAGCGCAGTTTTCGTACTCG | 0.556441 AATCGAGAGCCATGCCAAGG | CAGCGCAGTTTTCGTACTCG | 0.607832 CAAGCACGGAAATCGAGAGC | GTCGATGAGGACGATCTCGG | | | | | |
| NCU00721T0 | 3 | 5 | NCU00721T0\_input.txt | NCU00721T0\_output.txt | 0.216343 TTTCGTCGATGCTAGCCTGG | CCTCACCAAAGACCTCGACC | 0.567760 TTTCGTCGATGCTAGCCTGG | CTCACCAAAGACCTCGACCG | 0.579704 TTTCGTCGATGCTAGCCTGG | AGGTAGGCACTGGTGTTTCC | 0.648648 CGGCGAAATGTGCACATACC | AGGTAGGCACTGGTGTTTCC | 0.652564 TCAGAGGAATCACACTGCCC | CCTCACCAAAGACCTCGACC | | | | | |
| NCU00722T1 | 2 | 5 | NCU00722T1\_input.txt | NCU00722T1\_output.txt | 0.144063 CCATCCCAAGCCAAAACAGC | ATGGTTCGATGGCAGGAAGG | 0.144554 CCATCCCAAGCCAAAACAGC | AGACATGGTTCGATGGCAGG | 0.144749 CCATCCCAAGCCAAAACAGC | TGGAGACATGGTTCGATGGC | 0.146643 CCATCCCAAGCCAAAACAGC | ACATAGCAAACGCAGCAAGC | 0.427391 TCAATCGCGATACCCATCCC | ATGGTTCGATGGCAGGAAGG | | | | | |
| NCU00722T0 | 2 | 5 | NCU00722T0\_input.txt | NCU00722T0\_output.txt | 0.218396 CCATCCCAAGCCAAAACAGC | AGCGGAATTGGATCCAGACC | 0.288515 AGCTTTACCGGCTTCATCCC | AGCGGAATTGGATCCAGACC | 0.424006 AGCTTTACCGGCTTCATCCC | CAACCACCAAATCAACCCCG | 0.428176 CCATCCCAAGCCAAAACAGC | TGCGCATAGTCCTGTGTTCC | 0.436097 TTACCGGCTTCATCCCTTGG | AGCGGAATTGGATCCAGACC | | | | | |
| NCU00723T0 | 4 | 5 | NCU00723T0\_input.txt | NCU00723T0\_output.txt | 0.412995 TCGACAGCTTGGTGATAGGC | AGCAAATGTACACAACGCCG | 0.685259 TCGACAGCTTGGTGATAGGC | ATGTACACAACGCCGAAACG | 0.695778 ATCGTTAGCCTGACTGTGCG | AGCAAATGTACACAACGCCG | 0.699283 CTTGGTGATAGGCGGTGTCC | AGCAAATGTACACAACGCCG | 0.836780 TCGACAGCTTGGTGATAGGC | AAAGGTGCCGAAAGACAACG | | | | | |
| NCU00724T0 | 4 | 5 | NCU00724T0\_input.txt | NCU00724T0\_output.txt | 0.563671 TTCAGTACATGGACGACGCG | CCTCCAGATCATCAGTGCCC | 0.570255 GGGCAAGGAGTACACGATGG | CCTCCAGATCATCAGTGCCC | 0.996225 TAGAACAGACACAGAGCGCG | GTTCATAGCCATCCCGCTGG | 1.056979 TAGAACAGACACAGAGCGCG | CAAGTTCATAGCCATCCCGC | 1.098097 GTTCAGTACATGGACGACGC | CCTCCAGATCATCAGTGCCC | | | | | |
| NCU00725T0 | 4 | 5 | NCU00725T0\_input.txt | NCU00725T0\_output.txt | 0.856475 ACAAGAGACCATGGAACGGG | AGATCTGGCGCCATTCTTCG | 0.873453 ACAAGAGACCATGGAACGGG | AAGGGCCTTGTAGATCTGGC | 0.985963 ACAAGAGACCATGGAACGGG | ACTTTTCTGTGAAACGGCGG | 0.991864 ATGCAAGAGATCGCCGATGG | GAGGAGGGTAAGATGCGACC | 0.992822 GAGGCCACCAACAATGAACC | GAGGAGGGTAAGATGCGACC | | | | | |
| NCU00726T0 | 5 | 5 | NCU00726T0\_input.txt | NCU00726T0\_output.txt | 0.854122 AGGGTGAAGTTAATGCGGCC | AAATGGATTTCCTGCGCTGC | 0.907311 GAGGGTGAAGTTAATGCGGC | AAATGGATTTCCTGCGCTGC | 0.918576 TTAATGCGGCCAGACTGAGC | AAATGGATTTCCTGCGCTGC | 1.212118 AGAGCTCCTTGAAGTTGCGG | CACCCACCCTTCTTCACTGC | 1.240422 AGAGCTCCTTGAAGTTGCGG | CTGCATCGTCATCACTGTCG | | | | | |
| NCU00727T0 | 2 | 5 | NCU00727T0\_input.txt | NCU00727T0\_output.txt | 0.696560 TTGGACATGCAGGTCATCCC | CGTCGACCATGGCGTATACC | 0.839175 TTGGACATGCAGGTCATCCC | GCTTGTTCTTGATGGTCCGG | 0.849802 TTGGACATGCAGGTCATCCC | GATGGTCCGGTTCCAGATGC | 1.243807 TTGGACATGCAGGTCATCCC | TATTTCGGAGTTGGGGAGGG | 1.258779 TTGGACATGCAGGTCATCCC | ATGGTCCGGTTCCAGATGC | | | | | |
| NCU00728T0 | 2 | 5 | NCU00728T0\_input.txt | NCU00728T0\_output.txt | 0.697425 TGATTCTACAGGTCGACGGC | TGTAACCCTGCTTCTCGACG | 0.702336 TGATTCTACAGGTCGACGGC | AGCTGTAACCCTGCTTCTCG | 0.834566 GATTCTACAGGTCGACGGCG | TGTAACCCTGCTTCTCGACG | 0.839477 GATTCTACAGGTCGACGGCG | AGCTGTAACCCTGCTTCTCG | 1.321673 AGTGCGGTGGAAGAGATGC | TGTAACCCTGCTTCTCGACG | | | | | |
| NCU00729T0 | 2 | 5 | NCU00729T0\_input.txt | NCU00729T0\_output.txt | 0.498864 CTGATTCTCAGCTCTGCCCC | GGAGTGCCAATGTGTTTGCC | 0.498864 CCTGATTCTCAGCTCTGCCC | GGAGTGCCAATGTGTTTGCC | 0.498951 CTGATTCTCAGCTCTGCCCC | TGAGTTCGAGCTTGGAGTGC | 0.498951 CCTGATTCTCAGCTCTGCCC | TGAGTTCGAGCTTGGAGTGC | 0.503994 CTGATTCTCAGCTCTGCCCC | ACGGGAGTATATGGCATGGC | | | | | |
| NCU00730T0 | 5 | 5 | NCU00730T0\_input.txt | NCU00730T0\_output.txt | 0.352958 GAGTTTTCCAAACGCCACGG | AGCCCGTTGAAGGTGTATGG | 0.356046 GAGTTTTCCAAACGCCACGG | ACTACGGCGTTGAAGACTGG | 0.578922 GAGTTTTCCAAACGCCACGG | TGGCCTGGGACATAATTGGG | 0.691644 CGAGTTTTCCAAACGCCACG | AGCCCGTTGAAGGTGTATGG | 0.694732 CGAGTTTTCCAAACGCCACG | ACTACGGCGTTGAAGACTGG | | | | | |
| NCU00732T0 | 6 | 5 | NCU00732T0\_input.txt | NCU00732T0\_output.txt | 0.066639 TTATCGTCGAGGAAGCGAGC | AAAAGGCGTACGGTCTCTGG | 0.151185 TAAAAGCCCAGGGGTCAACC | AAAAGGCGTACGGTCTCTGG | 0.197162 TTATCGTCGAGGAAGCGAGC | CCAAAACTGGATCGAACGGC | 0.281708 TAAAAGCCCAGGGGTCAACC | CCAAAACTGGATCGAACGGC | 0.412984 TTATCGTCGAGGAAGCGAGC | CACCGACCAACCAAAACTGG | | | | | |
| NCU00733T0 | 1 | 5 | NCU00733T0\_input.txt | NCU00733T0\_output.txt | 0.143569 GAAGACCAGAAGGGACAGCC | GAAAGATGGGTGGAGACGGG | 0.492137 GAAGACCAGAAGGGACAGCC | AACAGACAGGGTCGATAGCG | 0.498126 CGAAGACCAGAAGGGACAGC | GAAAGATGGGTGGAGACGGG | 0.626544 GTTGCTATCTACTCCCGGCG | CTTTGTCGTTGCCACTGACC | 0.647042 GTCCATGTCCGCTATCGACC | ATCAGAACGGTGAAAGGGGG | | | | | |
| NCU00734T0 | 5 | 5 | NCU00734T0\_input.txt | NCU00734T0\_output.txt | 0.843232 TGGGAAGGACCTCTCTCTCG | GAACGAGCAGGAGCAGATGG | 0.991386 TGGGAAGGACCTCTCTCTCG | AAGTGCATGGAACGAGCAGG | 1.048035 TGGGAAGGACCTCTCTCTCG | TCGAAGTGCATGGAACGAGC | 1.061438 CCGGTGTAGGAAAGAAGGGG | GAACGAGCAGGAGCAGATGG | 1.061438 CCCGGTGTAGGAAAGAAGGG | GAACGAGCAGGAGCAGATGG | | | | | |
| NCU00735T0 | 2 | 5 | NCU00735T0\_input.txt | NCU00735T0\_output.txt | 0.762600 AAAACCAACTTGCACTCGCC | GGTGCGCTTGTCCTTTTTCG | 0.890943 TCCACATCTTGAACCCTGCC | CTGACACCACGTTGTTCTCG | 0.955077 AAAACCAACTTGCACTCGCC | CTGACACCACGTTGTTCTCG | 1.181059 AAAACCAACTTGCACTCGCC | CGATGAAGGCGTCTGACACC | 1.187527 TCCACATCTTGAACCCTGCC | TGACACCACGTTGTTCTCGC | | | | | |
| NCU00736T0 | 2 | 5 | NCU00736T0\_input.txt | NCU00736T0\_output.txt | 0.639445 CGTCTTCTGTAAGAGGCCGG | AGGATACAAGTCTTCGGCGG | 0.708342 CCATTTTGGTCTGAGCTGGC | ATTGTGGATATTGCCGGGGG | 0.708342 GCCATTTTGGTCTGAGCTGG | ATTGTGGATATTGCCGGGGG | 0.714311 CCATTTTGGTCTGAGCTGGC | TATTGCCGGGGGAATGAAGG | 0.714311 GCCATTTTGGTCTGAGCTGG | TATTGCCGGGGGAATGAAGG | | | | | |
| NCU00737T0 | 2 | 5 | NCU00737T0\_input.txt | NCU00737T0\_output.txt | 0.501592 GATGGAGATCAAGGAGGGCC | TTCTCGCCGTAGAAATCGGG | 0.509876 AGCACCGTGTTGATCCTAGC | TCTTGTTGAAGGCCCTCTGG | 0.578957 GATGGAGATCAAGGAGGGCC | AACGCTGGAGATCTTCTCGC | 0.719721 AAGGAGGGCCAGTTCTTTGC | AACGCTGGAGATCTTCTCGC | 0.785198 TGCGAGGATCAAGAAGCACC | AGCCATTGTCTGTAGCCAGC | | | | | |
| NCU00738T0 | 1 | 5 | NCU00738T0\_input.txt | NCU00738T0\_output.txt | 0.145710 GAGAGTCTGGCTTCGTGTCC | CTAACCACGGACTCCCAACC | 0.286539 TTCGTGTCCTTGAGCACAGG | CTAACCACGGACTCCCAACC | 0.575670 ACTGAATGGTTGGGAGTCCG | AACCGTCAGTGTCCCAATGG | 0.623027 AGATGACCGCCGGTATTTCC | AAACACATCACGACATGGCG | 0.634800 GAGAGTCTGGCTTCGTGTCC | TTCCTAAACTGCGCAATGGC | | | | | |
| NCU00738T1 | 2 | 5 | NCU00738T1\_input.txt | NCU00738T1\_output.txt | 0.716382 AGATGACCGCCGGTATTTCC | ACCTCCATGCAGCTTTCACC | 0.901704 GGGTGATGATTGCTTCCACG | CAACCGCTACAAGCTTCAGC | 0.912958 GGGTGATGATTGCTTCCACG | ATCCAGACATTTCCGACGGG | 0.985878 GGGTGATGATTGCTTCCACG | TCCGTGTTTTCCACCCATCC | 1.053009 GGGTGATGATTGCTTCCACG | CATTGCACAGAACCCCAACC | | | | | |
| NCU00739T0 | 1 | 5 | NCU00739T0\_input.txt | NCU00739T0\_output.txt | 0.209034 GTATCTCAGAGCGGCTTCCC | TAATGGTACGTTGGGCTCGG | 0.355212 TTCCCTTCTGCACCACTTGG | TAATGGTACGTTGGGCTCGG | 0.489891 CTCCGCCAGTATCTCAGAGC | TAATGGTACGTTGGGCTCGG | 0.628403 GTATCTCAGAGCGGCTTCCC | CGAGGGTGAGATCGATGAGG | 0.704903 GAAATGAGTCCCGAGGAGCG | TAATGGTACGTTGGGCTCGG | | | | | |
| NCU00740T0 | 2 | 5 | NCU00740T0\_input.txt | NCU00740T0\_output.txt | 0.152563 CCGCAAAGTCAATGGAGTGC | GCTTAAGTGCGCGATGTAGC | 0.215769 CCATTGGAGCAAAGCAGAGC | GCTTAAGTGCGCGATGTAGC | 0.217356 TCTCAGCAATCTGACCTCGC | GCTTAAGTGCGCGATGTAGC | 0.429151 CCGCAAAGTCAATGGAGTGC | TCCACAACTTCTTCGCTCGG | 0.492358 CCATTGGAGCAAAGCAGAGC | TCCACAACTTCTTCGCTCGG | | | | | |
| NCU00741T0 | 2 | 5 | NCU00741T0\_input.txt | NCU00741T0\_output.txt | 0.781299 AGAATGGCGAAAGGTGGACG | GATGGAGAAGGGGTTGTCCG | 1.063071 AGAATGGCGAAAGGTGGACG | TTCGGCTTCTGTAACGAGCC | 1.066721 AGAATGGCGAAAGGTGGACG | ATAATGGAAGACGGAGGCGG | 1.185155 ACATGATTGCCTGGGTCGG | GATGGAGAAGGGGTTGTCCG | 1.212695 AGAATGGCGAAAGGTGGACG | AACGAGCCTGATGGAGAAGG | | | | | |
| NCU00742T0 | 1 | 5 | NCU00742T0\_input.txt | NCU00742T0\_output.txt | 0.210667 GCGTTTTACGGCGGTTTAGG | CGCGAGGAAGGATCTTCTCC | 0.276716 GGCTGTTGAACGCATCATCG | GTAAAACGCCCCATGATCGC | 0.286177 GGTGGAGGAGATTCCCAAGC | CACGCATAAAACCGAACCCG | 0.334943 CGATCATGGGGCGTTTTACG | CGCGAGGAAGGATCTTCTCC | 0.358427 GCGTTTTACGGCGGTTTAGG | CACGCATAAAACCGAACCCG | | | | | |
| NCU00743T0 | 4 | 5 | NCU00743T0\_input.txt | NCU00743T0\_output.txt | 1.194049 AGTGTGCGGTTGAAGTCAGG | AGTGATTGCAAGCAAACGCG | 1.309570 TGTGGTGTAGAAGGCGTACG | GATAGAGACAACCGAGGCAGG | 1.309570 TGTGGTGTAGAAGGCGTACG | GGCAGATAGAGACAACCGAGG | 1.332041 GTTGAAGTCAGGGACGAGCC | AGTGATTGCAAGCAAACGCG | 1.332041 GGTTGAAGTCAGGGACGAGC | AGTGATTGCAAGCAAACGCG | | | | | |
| NCU00744T0 | 4 | 5 | NCU00744T0\_input.txt | NCU00744T0\_output.txt | 0.815784 GCAACGTCGACAAGTACTGC | CGACGACGATGATGACGAGG | 1.041618 TCGACAAGTACTGCTGTCGC | CGACGACGATGATGACGAGG | 1.467944 AGATTTGGCACGCATTCAGG | CGACGACGATGATGACGAGG | 1.690268 GCAACGTCGACAAGTACTGC | AGGCGCCTATCCAAATGCC | 1.817465 GCAACGTCGACAAGTACTGC | CAGGCGCCTATCCAAATGC | | | | | |
| NCU00745T0 | 2 | 5 | NCU00745T0\_input.txt | NCU00745T0\_output.txt | 1.051217 TTTGAAGCGGTTGGTTGACC | TTGTCGGACATTTGGTTGCG | 1.198073 GTGGTGCTTGGAGGTAGACG | TCAATTGGATGAAACGCCGG | 1.389190 GCTTGGCCGGACATTTTGG | TTGTCGGACATTTGGTTGCG | 1.404885 TTTGAAGCGGTTGGTTGACC | CGGACATTTGGTTGCGTTGG | 1.415614 CTGAAGGTGGTGCTTGGAGG | TCAATTGGATGAAACGCCGG | | | | | |
| NCU00746T0 | 3 | 5 | NCU00746T0\_input.txt | NCU00746T0\_output.txt | 0.420109 CATGGCCTGTTGAACGAACC | GGAGTGGTGCGCTTTTATGC | 0.494098 ACTGCTGGTCAAATTTGGCG | GGAGTGGTGCGCTTTTATGC | 0.563112 TTTGCTTGTCGGACTTTGGC | GGAGTGGTGCGCTTTTATGC | 0.619908 CATGGCCTGTTGAACGAACC | GACCTGACGATGAGGAGACG | 0.693897 ACTGCTGGTCAAATTTGGCG | GACCTGACGATGAGGAGACG | | | | | |
| NCU00747T0 | 1 | 5 | NCU00747T0\_input.txt | NCU00747T0\_output.txt | 0.140946 CAGTGGGGGTTGTGTATCCC | GGATTTCCTCTTCCCCAGCC | 0.140946 CCAGTGGGGGTTGTGTATCC | GGATTTCCTCTTCCCCAGCC | 0.267493 GAAACTCGCGTCTGTTTCGG | GGATTTCCTCTTCCCCAGCC | 0.269353 GAAACTCGCGTCTGTTTCGG | TTCGATGGAAACTCCTCGGC | 0.285498 GGCTGGGGAAGAGGAAATCC | AGACCTCCCAACAACCAACG | | | | | |
| NCU00748T0 | 1 | 5 | NCU00748T0\_input.txt | NCU00748T0\_output.txt | 0.139316 GGGGGATTCTGAGTGTGTCG | CTTGGTTGCTTCACCACTGC | 0.210297 GAAGGGTATCCGTGTGGTCG | CTTGGTTGCTTCACCACTGC | 0.210788 TAAGGCCCGTCCTATTTGCG | GATCGACCACACGGATACCC | 0.212157 TAAGGCCCGTCCTATTTGCG | CGGATACCCTTCCTTACGGC | 0.268956 TTTGCGATCAAACAGTCGCC | GATCGACCACACGGATACCC | | | | | |
| NCU00749T0 | 2 | 5 | NCU00749T0\_input.txt | NCU00749T0\_output.txt | 0.636330 TGCAAACGAACCAAAGCAGC | GGAATTGCTCTGGTTGCAGC | 0.636704 TGCAAACGAACCAAAGCAGC | ACGGGTCAAATGCGAAATGC | 0.695747 GGACTGACTGCAAACGAACC | GGAATTGCTCTGGTTGCAGC | 0.696121 GGACTGACTGCAAACGAACC | ACGGGTCAAATGCGAAATGC | 0.705027 AACAGCATAAACCGTGTGCC | GGAATTGCTCTGGTTGCAGC | | | | | |
| NCU00751T0 | 3 | 5 | NCU00751T0\_input.txt | NCU00751T0\_output.txt | 0.216324 GAAGGTACCCAATCCGGACG | CTTGACCGTCCAAGACTCCC | 0.216324 GAAGGTACCCAATCCGGACG | CCTTGACCGTCCAAGACTCC | 0.280057 ACACGTGGACCAGTCATACG | CTTGACCGTCCAAGACTCCC | 0.280057 ACACGTGGACCAGTCATACG | CCTTGACCGTCCAAGACTCC | 0.431289 GAAGGTACCCAATCCGGACG | ACCGATGTTCTAGGTTGGCC | | | | | |
| NCU00752T0 | 1 | 5 | NCU00752T0\_input.txt | NCU00752T0\_output.txt | 0.210810 CAAAAGCGATGGTCGAACCG | TGGACGTGAAGTTGTCGAGG | 0.211522 ATGCTTCACCCTCAGCTAGC | TGGACGTGAAGTTGTCGAGG | 0.279025 AGCCAATCCGACCAATACCG | TGAATTCGTACTCGGCGAGG | 0.279193 CAAAAGCGATGGTCGAACCG | TGAATTCGTACTCGGCGAGG | 0.349290 ATCCGACCAATACCGCTTCG | TGAATTCGTACTCGGCGAGG | | | | | |
| NCU00753T0 | 3 | 5 | NCU00753T0\_input.txt | NCU00753T0\_output.txt | 0.358231 CTCTTGAGCTCGACTTCCGG | GTCTGGATCAGGTAACGGGC | 0.637693 CTCTTGAGCTCGACTTCCGG | CGTCGTTGGTCTGGATCAGG | 0.643603 CTCTTGAGCTCGACTTCCGG | GGTCTGGATCAGGTAACGGG | 0.703047 ACTCTTGAGCTCGACTTCCG | GTCTGGATCAGGTAACGGGC | 0.779405 CTCTTGAGCTCGACTTCCGG | GGATCAGGTAACGGGCATCG | | | | | |
| NCU00754T0 | 3 | 5 | NCU00754T0\_input.txt | NCU00754T0\_output.txt | 1.590905 TCCATAGACTTCGGAAAGCGG | TACTTGATGTCGGACGAGCC | 1.821455 GGAGCGAAAACCATCGGACC | TACTTGATGTCGGACGAGCC | 2.026519 CCATAGACTTCGGAAAGCGG | TACTTGATGTCGGACGAGCC | 2.203517 TCCATAGACTTCGGAAAGCGG | ATACTTGATGTCGGACGAGCC | 2.351951 TCCATAGACTTCGGAAAGCGG | ACTTGATGTCGGACGAGCC | | | | | |
| NCU00755T0 | 6 | 5 | NCU00755T0\_input.txt | NCU00755T0\_output.txt | 0.428915 AGTTGCAATTGGTTTGGCCG | CTTCATCAGACCTCAGCCGG | 0.709833 TTCGTTCTCGACTGGATGGG | CTTCATCAGACCTCAGCCGG | 0.768242 TTCGTTCTCGACTGGATGGG | GTTTTCGGCTTCTTGGACCG | 0.790749 AGTTGCAATTGGTTTGGCCG | AGCTTCATCAGACCTCAGCC | 1.016361 TACTACCTGATTGCCCTGCC | CTTCATCAGACCTCAGCCGG | | | | | |
| NCU00756T0 | 3 | 5 | NCU00756T0\_input.txt | NCU00756T0\_output.txt | 0.425370 TCCAAGACGGCGTACATTGG | TGATGCAAGGAGGCTCAAGG | 0.425897 TGGAGGCGATTTCCAAGACG | TGATGCAAGGAGGCTCAAGG | 0.501247 TCCAAGACGGCGTACATTGG | GAGAAAACGCAACCAGCTCG | 0.501774 TGGAGGCGATTTCCAAGACG | GAGAAAACGCAACCAGCTCG | 0.503911 TTGGCCCCAAGTCTCAAACC | TGATGCAAGGAGGCTCAAGG | | | | | |
| NCU00757T0 | 2 | 5 | NCU00757T0\_input.txt | NCU00757T0\_output.txt | 0.069993 AGCTCTAGTGCGGATGATGC | TCCAAACCCGTCATTCGAGG | 0.139192 AGCTCTAGTGCGGATGATGC | TGTCGGTGAAGTGGTTCTGG | 0.359492 AGCTCTAGTGCGGATGATGC | CCTCCACTCAAAGTCCCAGG | 0.423700 AGCTCTAGTGCGGATGATGC | CTCAAAGTCCCAGGTCTCGC | 0.649078 AGCTCTAGTGCGGATGATGC | CCTCCTCCACTCAAAGTCCC | | | | | |
| NCU00758T0 | 5 | 5 | NCU00758T0\_input.txt | NCU00758T0\_output.txt | 0.206599 TTGGTATCTGGCACCACACC | GAACATTCCACAGAAGCCGC | 0.211298 TTGGTATCTGGCACCACACC | CACTCTCGTACTGGGGAAGC | 0.486445 GTGTCTCGCTGCCTATCTCG | GAACATTCCACAGAAGCCGC | 0.713427 TTGGTATCTGGCACCACACC | TCGTCACTCTCGTACTGGGG | 0.792732 TCCCTTTGGTATCTGGCACC | GAACATTCCACAGAAGCCGC | | | | | |
| NCU00759T1 | 2 | 5 | NCU00759T1\_input.txt | NCU00759T1\_output.txt | 0.355950 TTCAAAAGTCGTGGTTGCGC | GTTCGGATGGACTTGGGAGG | 0.356686 GCACAAGCTTGTCCAACACC | GTTCGGATGGACTTGGGAGG | 0.421641 TGTCTGTACCTGTCACAGCG | GTTCGGATGGACTTGGGAGG | 0.427316 TTCAAAAGTCGTGGTTGCGC | TATCCCATGCTTGAAGGGCG | 0.427456 TTCAAAAGTCGTGGTTGCGC | GTGTCCATGGACTCGAGACG | | | | | |
| NCU00759T0 | 2 | 5 | NCU00759T0\_input.txt | NCU00759T0\_output.txt | 0.355950 TTCAAAAGTCGTGGTTGCGC | GTTCGGATGGACTTGGGAGG | 0.356686 GCACAAGCTTGTCCAACACC | GTTCGGATGGACTTGGGAGG | 0.421641 TGTCTGTACCTGTCACAGCG | GTTCGGATGGACTTGGGAGG | 0.427316 TTCAAAAGTCGTGGTTGCGC | TATCCCATGCTTGAAGGGCG | 0.427456 TTCAAAAGTCGTGGTTGCGC | GTGTCCATGGACTCGAGACG | | | | | |
| NCU00760T0 | 5 | 5 | NCU00760T0\_input.txt | NCU00760T0\_output.txt | 0.349478 GGCCGAGACACCAAAATTCG | CAGCTCCATAACCTCCACCC | 0.437398 TGGAGGATGCAGTTTCAGGG | AACCCTACAGCATCACGAGC | 0.503036 GAGGATGCAGTTTCAGGGGG | AACCCTACAGCATCACGAGC | 0.503036 GGAGGATGCAGTTTCAGGGG | AACCCTACAGCATCACGAGC | 0.508371 TGGAGGATGCAGTTTCAGGG | ACCGCTATCCACACATCACG | | | | | |
| NCU00761T1 | 3 | 5 | NCU00761T1\_input.txt | NCU00761T1\_output.txt | 0.427775 GGCCGAGACACCAAAATTCG | TCCATTTGATGAGGGGCTGG | 0.834502 GGCCGAGACACCAAAATTCG | GAGGTTGTTGTTTGGGACGG | 0.921975 GGCCGAGACACCAAAATTCG | AGGTTGTTGTTTGGGACGGG | 0.926063 GGCCGAGACACCAAAATTCG | CATTTGATGAGGGGCTGGGG | 0.926063 GGCCGAGACACCAAAATTCG | CCATTTGATGAGGGGCTGGG | | | | | |
| NCU00761T0 | 2 | 5 | NCU00761T0\_input.txt | NCU00761T0\_output.txt | 0.215436 TTATGCCGACTGCCATCTGG | ATACGTTGAGGTGCTGTGGG | 0.295277 AGTGGAGATGAACTGCCTGC | TCCATTTGATGAGGGGCTGG | 0.367301 GTCCCCGTGGAGAGAATTGG | TCCATTTGATGAGGGGCTGG | 0.438695 TTATGCCGACTGCCATCTGG | TCCATTTGATGAGGGGCTGG | 0.507107 GGCAAAGATCAAACAGGGGC | TCCATTTGATGAGGGGCTGG | | | | | |
| NCU00762T0 | 2 | 5 | NCU00762T0\_input.txt | NCU00762T0\_output.txt | 1.186470 GATCCTTGAGTTCAGGGCCC | GAGTTGCTGTAGGGAAAGCG | 1.445167 GATCCTTGAGTTCAGGGCCC | AGAGTTGCTGTAGGGAAAGCG | 1.700059 TGATCCTTGAGTTCAGGGCC | GAGTTGCTGTAGGGAAAGCG | 1.958755 TGATCCTTGAGTTCAGGGCC | AGAGTTGCTGTAGGGAAAGCG | 2.186893 GATCCTTGAGTTCAGGGCCC | CAGAGTTGCTGTAGGGAAAGC | | | | | |
| NCU00763T0 | 6 | 5 | NCU00763T0\_input.txt | NCU00763T0\_output.txt | 0.073221 TGTGGGGGTCAAGATTCTGC | AGGAAACGGCATTCTGGAGG | 0.148652 TGTGGGGGTCAAGATTCTGC | TTCTGGAGGGTTTGTGGAGC | 0.288289 TGTGGGGGTCAAGATTCTGC | TAATTGCCGGCGATGGTAGG | 0.364316 TCAGCTTCAAGATGTGGGGG | AGGAAACGGCATTCTGGAGG | 0.439747 TCAGCTTCAAGATGTGGGGG | TTCTGGAGGGTTTGTGGAGC | | | | | |
| NCU00764T0 | 1 | 5 | NCU00764T0\_input.txt | NCU00764T0\_output.txt | 0.501793 TGAGCATCTTTTCCAGCCCC | CATGTCGGTTAGGGGTGAGG | 0.501793 TGAGCATCTTTTCCAGCCCC | TAGATGCCGTCCTTTCTGCC | 0.573931 TCTCCTCGATCACCACCTCC | CATGTCGGTTAGGGGTGAGG | 0.575640 TGAGCATCTTTTCCAGCCCC | TTCCCAGCATGTCGGTTAGG | 0.640029 CCAACCTCTCCTCGATCACC | CATGTCGGTTAGGGGTGAGG | | | | | |
| NCU00765T0 | 6 | 5 | NCU00765T0\_input.txt | NCU00765T0\_output.txt | 0.428558 ATCTTCTTCTTCCCCACGCG | AACCGATCACACTCCAACGG | 0.703232 ATCTTCTTCTTCCCCACGCG | CCGGTATAGTAGTGGCGTCC | 0.707660 GTCTGGGTCAATGCGTTTGC | AACCGATCACACTCCAACGG | 0.774143 TGCAAGATGGTCTATGGGCG | CCGGTATAGTAGTGGCGTCC | 0.924128 ACAGTGTCTGGGTCAATGCG | AACCGATCACACTCCAACGG | | | | | |
| NCU00766T0 | 1 | 5 | NCU00766T0\_input.txt | NCU00766T0\_output.txt | 0.495165 CCTCGGTCTTGCTCTCATCC | AACCAAACCCGCAAATCACC | 0.569927 TTGGTGATTTGCGGGTTTGG | TCGGTCACACACTCCAAACC | 0.641630 TTGGTGATTTGCGGGTTTGG | GGTGGGGTGTCTGTCTTTCC | 0.648057 CCTCGGTCTTGCTCTCATCC | TCAAAATGGCCTCCAACCCC | 0.778793 GCTCCTTCTCTAGGGAAGCG | AACCAAACCCGCAAATCACC | | | | | |
| NCU00768T0 | 11 | 5 | NCU00768T0\_input.txt | NCU00768T0\_output.txt | 0.288243 TTATGGCGCTCCTCACTTCG | GAAATGCTCAAGCCAACCGG | 0.288243 TTATGGCGCTCCTCACTTCG | GGAAATGCTCAAGCCAACCG | 0.422746 TTATGGCGCTCCTCACTTCG | CTGGGGACGTTTTGATTGCC | 0.422746 TTATGGCGCTCCTCACTTCG | CCTGGGGACGTTTTGATTGC | 0.426571 TTATGGCGCTCCTCACTTCG | GGGAAATGCTCAAGCCAACC | | | | | |
| NCU00770T0 | 2 | 5 | NCU00770T0\_input.txt | NCU00770T0\_output.txt | 0.145002 TGATAGTCGTGTCGGTTGGC | AATGCAGGAAGGGACTGTCG | 0.217321 TGATAGTCGTGTCGGTTGGC | ACGCCCTAGTGTCAAATCCG | 0.278222 TGATAGTCGTGTCGGTTGGC | TTTGTGTGTGTGACAACGCC | 1.244795 TGATAGTCGTGTCGGTTGGC | ACTTTGTGTGTGTGACAACGC | 1.448089 TGATAGTCGTGTCGGTTGGC | AGGGACTGTCGGTATATGGC | | | | | |
| NCU00771T0 | 2 | 5 | NCU00771T0\_input.txt | NCU00771T0\_output.txt | 0.972907 GAGCTTGATGGTGCTTTCGG | GTAGGCAAGGTCAAAGCTGC | 0.972907 GGAGCTTGATGGTGCTTTCG | GTAGGCAAGGTCAAAGCTGC | 1.240343 GCGGCAGTAAAGACAAGCG | TCGATGGTGGTGAGGAATGC | 1.240343 CGCGGCAGTAAAGACAAGC | TCGATGGTGGTGAGGAATGC | 1.243018 CCGTCAATGGTTCTGCATTGG | TCGATGGTGGTGAGGAATGC | | | | | |
| NCU00772T1 | 3 | 5 | NCU00772T1\_input.txt | NCU00772T1\_output.txt | 0.873983 CTTCTACTTCGTCCTCGGCG | AGCATTGGACTGGGTTAGGG | 1.095766 TTGATAGCTACTGCCTGGCC | AGCATTGGACTGGGTTAGGG | 1.097466 AAAGGATGGCGATCTCCTGG | AGCATTGGACTGGGTTAGGG | 1.227160 CGGAAAGGATGGCGATCTCC | AGCATTGGACTGGGTTAGGG | 1.530001 CTTCTACTTCGTCCTCGGCG | TCAGCATTGGACTGGGTTAGG | | | | | |
| NCU00772T0 | 4 | 5 | NCU00772T0\_input.txt | NCU00772T0\_output.txt | 0.873983 CTTCTACTTCGTCCTCGGCG | AGCATTGGACTGGGTTAGGG | 1.095766 TTGATAGCTACTGCCTGGCC | AGCATTGGACTGGGTTAGGG | 1.097466 AAAGGATGGCGATCTCCTGG | AGCATTGGACTGGGTTAGGG | 1.227160 CGGAAAGGATGGCGATCTCC | AGCATTGGACTGGGTTAGGG | 1.530001 CTTCTACTTCGTCCTCGGCG | TCAGCATTGGACTGGGTTAGG | | | | | |
| NCU00773T0 | 2 | 5 | NCU00773T0\_input.txt | NCU00773T0\_output.txt | 0.574075 TCTGTTTCCCACACTGCACC | GGATACGGATGCGGATCTGG | 0.636915 TCTGTTTCCCACACTGCACC | GACTGCTGAGTAGTGACGGG | 0.851936 TCTGTTTCCCACACTGCACC | GACGTTTGGGGGTCAAAACC | 0.864694 TCTGTTTCCCACACTGCACC | TTTGGGGGTCAAAACCTGGG | 0.919905 TGGTTCGATCGTATCCCACC | GACTGCTGAGTAGTGACGGG | | | | | |
| NCU00774T0 | 2 | 5 | NCU00774T0\_input.txt | NCU00774T0\_output.txt | 0.150080 GCAGACTCAAGCGAAAACCG | ATGAGTGTCGTCGTGTGTCC | 0.150080 CGCAGACTCAAGCGAAAACC | ATGAGTGTCGTCGTGTGTCC | 0.153092 GCAGACTCAAGCGAAAACCG | GTGTCCGAGTTGTTTGACGC | 0.153092 CGCAGACTCAAGCGAAAACC | GTGTCCGAGTTGTTTGACGC | 0.424946 GCAGACTCAAGCGAAAACCG | GACGTCGGAGATATGGTGGG | | | | | |
| NCU00775T0 | 3 | 5 | NCU00775T0\_input.txt | NCU00775T0\_output.txt | 0.571988 TGGTGGGCTTGAAGATGTCG | ATTTGTTGCCAAAGCCCTGC | 0.712385 GAAGATGTCGGACTGGAGGG | ATTTGTTGCCAAAGCCCTGC | 0.920049 GAGGGTGACGGTGTACTTGC | ATTTGTTGCCAAAGCCCTGC | 1.277142 GGTGGGCTTGAAGATGTCGG | ATTTGTTGCCAAAGCCCTGC | 1.330105 TACTTGCCGCCGTACTTGG | ATTTGTTGCCAAAGCCCTGC | | | | | |
| NCU00776T0 | 3 | 5 | NCU00776T0\_input.txt | NCU00776T0\_output.txt | 0.350489 AGTCTAGCTTGGCTTCGTCG | ATCGATGAGGAAGGCTTGCC | 0.350489 ACGAGTCTAGCTTGGCTTCG | ATCGATGAGGAAGGCTTGCC | 0.546560 AGTCTAGCTTGGCTTCGTCG | GCTTGCCACATTACTCTCGC | 0.546560 ACGAGTCTAGCTTGGCTTCG | GCTTGCCACATTACTCTCGC | 0.642288 AGTCTAGCTTGGCTTCGTCG | ATCCTGAGATCACCGAGGGG | | | | | |
| NCU00777T0 | 3 | 5 | NCU00777T0\_input.txt | NCU00777T0\_output.txt | 0.210000 ATCTCACCCTACAGGACCCC | ACTTGAAGTGGAAGGTGGCC | 0.431187 CCCCTTTGGACCTTCCTACG | ACTTGAAGTGGAAGGTGGCC | 0.624138 ATCTCACCCTACAGGACCCC | CGCCAACTTGAAGTGGAAGG | 0.845325 CCCCTTTGGACCTTCCTACG | CGCCAACTTGAAGTGGAAGG | 0.926569 ATCTCACCCTACAGGACCCC | CTTGAAGTGGAAGGTGGCCC | | | | | |
| NCU00778T0 | 4 | 5 | NCU00778T0\_input.txt | NCU00778T0\_output.txt | 0.562406 AGGTGGGAACATTGGATGGC | ATACGATCGTCACTAGCGCC | 0.614613 TCATGTTTTGATAGGCGCGC | ATACGATCGTCACTAGCGCC | 0.627709 ATGGTTGACGAGAACGAGGC | ATACGATCGTCACTAGCGCC | 0.636375 CCAGCCATACACAAATGCCG | TACCTCTCATGACAACGGCC | 0.859980 AGGTGGGAACATTGGATGGC | TACCTCTCATGACAACGGCC | | | | | |
| NCU00779T0 | 1 | 5 | NCU00779T0\_input.txt | NCU00779T0\_output.txt | 0.072962 TCTTGAAGGAGCTGCGAAGG | ACTCTTCCCATGCTTGGTCG | 0.140250 CCCTTCGACGGTATCTGAGC | CATGGGTGCATCCTAGGTCC | 0.209934 CCCTTCGACGGTATCTGAGC | TCCTAGGTCCTCGTCATCCG | 0.210069 AGACTTCGATTCGGACAGCG | GCTCAGATACCGTCGAAGGG | 0.217326 AGACTTCGATTCGGACAGCG | TCACCATGGGCTGAAGAACC | | | | | |
| NCU00780T0 | 1 | 5 | NCU00780T0\_input.txt | NCU00780T0\_output.txt | 0.358140 CTACCAGGATCACAGTGGCC | CAGAGTCTCTCCGAGCAAGC | 0.419719 TTGCGGAGTCTACGTTGAGC | TGCGGCATCGTATCTACACC | 0.423379 GGCTTTCTTCACGGAGGAGG | TGCGGCATCGTATCTACACC | 0.491686 GGTTGGTCAACACAAAGGCC | ATTGTGGAACGTTGCCAACC | 0.491686 GGGTTGGTCAACACAAAGGC | ATTGTGGAACGTTGCCAACC | | | | | |
| NCU00781T0 | 1 | 5 | NCU00781T0\_input.txt | NCU00781T0\_output.txt | 0.148473 CCACAAGACTAAGGCCCTCG | AGTCGTAGCACATATCGCCG | 0.403024 GAACGCTAACATCTGCGACG | CGAGGGCCTTAGTCTTGTGG | 0.433468 GAGGCCACAAGACTAAGGCC | AGTCGTAGCACATATCGCCG | 0.656186 AGAGGCCACAAGACTAAGGC | AGTCGTAGCACATATCGCCG | 0.688019 GAACGCTAACATCTGCGACG | GAGGGCCTTAGTCTTGTGGC | | | | | |
| NCU00782T0 | 4 | 5 | NCU00782T0\_input.txt | NCU00782T0\_output.txt | 0.494974 TGAAGAAGTTCCGCCCTTCC | CATCTGGCTGCCATCAAACC | 0.705930 TGAAGAAGTTCCGCCCTTCC | AAACTCGGAGCGCTTGTACC | 0.782150 TGAAGAAGTTCCGCCCTTCC | CAGCCCGAGAAACCATCTGG | 0.908057 CATGCTGAAGAAGTTCCGCC | CATCTGGCTGCCATCAAACC | 0.914132 TGTCTCGTATGCGGTGTTCG | CATCTGGCTGCCATCAAACC | | | | | |
| NCU00783T0 | 1 | 5 | NCU00783T0\_input.txt | NCU00783T0\_output.txt | 0.288116 GTCCACAGCTGCCTCTTAGG | TGGATTATGCAGTGGGGTCG | 0.414974 ACCCCACTGCATAATCCACG | GACTTTGTTGGTGTCGCAGG | 0.415705 GTCCACAGCTGCCTCTTAGG | GACTTTGTTGGTGTCGCAGG | 0.416938 GTCCACAGCTGCCTCTTAGG | GCTGGAAGCGTGGATTATGC | 0.420361 GTCCACAGCTGCCTCTTAGG | CTCAAGATAGACGGGGAGCG | | | | | |
| NCU00784T0 | 3 | 5 | NCU00784T0\_input.txt | NCU00784T0\_output.txt | 0.625931 GCTGCAACTAAACCAGACGG | TCTAGAAGGCTGCAACGAGC | 0.625931 GGCTGCAACTAAACCAGACG | TCTAGAAGGCTGCAACGAGC | 0.626588 GCTGCAACTAAACCAGACGG | TCGTCTAGAAGGCTGCAACG | 0.626588 GGCTGCAACTAAACCAGACG | TCGTCTAGAAGGCTGCAACG | 0.687877 GCTGCAACTAAACCAGACGG | TTCGTCGTCTAGAAGGCTGC | | | | | |
| NCU00786T0 | 4 | 5 | NCU00786T0\_input.txt | NCU00786T0\_output.txt | 0.280189 TCCGGAGAGAAAGATGCACG | TGCGAGTCATTAGGGATGGC | 0.287329 AGAGGATTCCATGTGTCGGC | AATGGAGGCTCGTACAACGG | 0.495489 TCCGGAGAGAAAGATGCACG | AAGAAGAGCGGCAAGAAGGG | 0.501008 AGAGGATTCCATGTGTCGGC | AAGAAGAGCGGCAAGAAGGG | 0.557291 TACCGTAGCTGTGCTTCTCG | TGCGAGTCATTAGGGATGGC | | | | | |
| NCU00787T0 | 1 | 5 | NCU00787T0\_input.txt | NCU00787T0\_output.txt | 0.071701 ACCTCGAAAACTCGACGACC | CAACAAAAACCAGGCCTCCG | 0.071701 ACCTCGAAAACTCGACGACC | CCTCCGCAACAAAAACCAGG | 0.145521 TGACCCGTACTTTCCCAAGC | CCCTCAAATTCACGCGTTGG | 0.149489 ACCTCGAAAACTCGACGACC | CCCTCAAATTCACGCGTTGG | 0.211061 TAAGGATAAGGCGTTGGGCG | CAACAAAAACCAGGCCTCCG | | | | | |
| NCU00788T0 | 2 | 5 | NCU00788T0\_input.txt | NCU00788T0\_output.txt | 0.280560 CGAGAATCAGGAGACACGGG | CTCGCAGGTAGTGGTCTTCG | 0.280560 CCGAGAATCAGGAGACACGG | CTCGCAGGTAGTGGTCTTCG | 0.286815 AGGCGAAAGATGCCTGTACC | CTCGCAGGTAGTGGTCTTCG | 0.349403 AGGCGAAAGATGCCTGTACC | TATTGCTACGCGTATCCCCG | 0.355150 GAATCAGGAGACACGGGACC | CTCGCAGGTAGTGGTCTTCG | | | | | |
| NCU00789T0 | 3 | 5 | NCU00789T0\_input.txt | NCU00789T0\_output.txt | 0.347955 GAGTTGAGGGAGAGCGATCG | GCGCAAAGTTGGACACTTCC | 0.350475 GAGTTGAGGGAGAGCGATCG | TATAGGGATCCAAAGCGCCG | 0.496989 GAGACTTCCATCGTGGTCGG | GCGCAAAGTTGGACACTTCC | 0.853860 GCCCATCCATCCGAAGAAGG | GCGCAAAGTTGGACACTTCC | 0.856380 GCCCATCCATCCGAAGAAGG | TATAGGGATCCAAAGCGCCG | | | | | |
| NCU00790T0 | 3 | 5 | NCU00790T0\_input.txt | NCU00790T0\_output.txt | 0.066967 TCCATGCGGATAATGAGGGC | GAGCAAAACGTGGGTGAACC | 0.075806 TCCATGCGGATAATGAGGGC | ACAGTCACTTCTTCTCGCGG | 0.209253 ATAATGAGGGCGAAGGTGGC | GAGCAAAACGTGGGTGAACC | 0.215805 TCCATGCGGATAATGAGGGC | GGACATGACCATGGAGACGG | 0.358090 ATAATGAGGGCGAAGGTGGC | GGACATGACCATGGAGACGG | | | | | |
| NCU00792T0 | 4 | 5 | NCU00792T0\_input.txt | NCU00792T0\_output.txt | 0.357700 CACGAGCGACAGTATCCAGG | GTGACTGTCTCCTCTTCGGC | 0.496766 ACGAGTTTAGCACCAGTGGG | TGATTGAGCGAATGGGGACG | 0.567360 CACGAGCGACAGTATCCAGG | TGTTGAAGGAGACACGAGCG | 0.629998 GGGTTCATTGGCGTTCTTCG | TGATTGAGCGAATGGGGACG | 0.640855 CACGAGCGACAGTATCCAGG | CTCTTGGGTAGCAGTGCTGG | | | | | |
| NCU00793T0 | 4 | 5 | NCU00793T0\_input.txt | NCU00793T0\_output.txt | 0.693448 GTAACCCAGGGCGTTAGTGG | CAAAGCAAACAAGCAACGCC | 0.707361 GTAACCCAGGGCGTTAGTGG | GCAAAGCAAACAAGCAACGC | 0.972164 GTAACCCAGGGCGTTAGTGG | CTGCAGCAAAGCAAACAAGC | 1.044733 CCAGGGCGTTAGTGGTAAGC | CAAAGCAAACAAGCAACGCC | 1.058647 CCAGGGCGTTAGTGGTAAGC | GCAAAGCAAACAAGCAACGC | | | | | |
| NCU00794T0 | 4 | 5 | NCU00794T0\_input.txt | NCU00794T0\_output.txt | 0.134027 CTCAAGGCATGGAATGTGCG | GTCATATCACCCCGCTGAGG | 0.136357 CTCAAGGCATGGAATGTGCG | TTTCTCCATGTCCTCACGGC | 0.206863 CTCAAGGCATGGAATGTGCG | CCTTTCCGCCTGATCCTACC | 0.215461 CTACCAATGCTCCTGGTCCG | TTTCTCCATGTCCTCACGGC | 0.277110 CTCAAGGCATGGAATGTGCG | TCACGGCCTTATCCTTTCCG | | | | | |
| NCU00795T0 | 2 | 5 | NCU00795T0\_input.txt | NCU00795T0\_output.txt | 0.210984 CACCTTTGGAAACGCTGTCG | CAAACAAGCGCTCATCTGGG | 0.220449 CACCTTTGGAAACGCTGTCG | TGTTGTAGATCTGCTCGCGG | 0.279269 GGTTGCTGCCTTGATGAACG | CAAACAAGCGCTCATCTGGG | 0.279865 CACCTTTGGAAACGCTGTCG | TAGATCTGCTCGCGGAATCG | 0.280316 CTTTGGAAACGCTGTCGAGC | CAAACAAGCGCTCATCTGGG | | | | | |
| NCU00796T0 | 3 | 5 | NCU00796T0\_input.txt | NCU00796T0\_output.txt | 0.210164 GGTTGAGATGCTGCCTTTGC | GGCTTTCCCATTGTCATCGC | 0.274700 TGCTTCTCCGGAATCTGTCG | GGCTTTCCCATTGTCATCGC | 0.274927 GTCTACAGAGGCCAAGACGG | GGCTTTCCCATTGTCATCGC | 0.288023 GGTTGAGATGCTGCCTTTGC | TGTTGCTGCTTTTGGCTTGG | 0.352559 TGCTTCTCCGGAATCTGTCG | TGTTGCTGCTTTTGGCTTGG | | | | | |
| NCU00797T0 | 1 | 5 | NCU00797T0\_input.txt | NCU00797T0\_output.txt | 0.504963 ACCCCATCAAGATTCACCGG | CTACGGTCAGCATTCCTCCG | 0.565812 ACCCCATCAAGATTCACCGG | CGTCTGATGATTGCATGCCC | 0.573493 ACCCCATCAAGATTCACCGG | GCGTCTGATGATTGCATGCC | 0.712202 ACCCCATCAAGATTCACCGG | CACTTCGGTCTACGGTCAGC | 0.779066 TGGGCTCTGGACAAAAACCC | CGTCTGATGATTGCATGCCC | | | | | |
| NCU00798T0 | 1 | 5 | NCU00798T0\_input.txt | NCU00798T0\_output.txt | 0.644743 ACGCCTCAGTTGTTGTAGGC | GAATCATCGACCATGCGTGC | 0.703391 ATCGGATGTAGTGGTCGTCG | GAATCATCGACCATGCGTGC | 0.842905 GGATGTAGTGGTCGTCGTGC | GAATCATCGACCATGCGTGC | 0.928329 AAGTCGGCAGAGCTTGTTGG | GAATCATCGACCATGCGTGC | 1.058454 TGATCAGAGAATGGACGCGG | TGTGTGATTGCTTCTCCGGC | | | | | |
| NCU00799T0 | 2 | 5 | NCU00799T0\_input.txt | NCU00799T0\_output.txt | 0.138263 ACGAAGATGCGATCCAGACC | CTAGCACTGTTTCCCAGGGG | 0.140082 ACGAAGATGCGATCCAGACC | ACTGGCTAGCACTGTTTCCC | 0.141223 TGGTCCTCTATCCAGACGGG | CTAGCACTGTTTCCCAGGGG | 0.142899 TGGTCCTCTATCCAGACGGG | TGGAGAGGTTGACATGTGGC | 0.143001 AAGAACGGAGAGCAGATGGC | CTAGCACTGTTTCCCAGGGG | | | | | |
| NCU00800T0 | 4 | 5 | NCU00800T0\_input.txt | NCU00800T0\_output.txt | 0.138479 CGTCATGAGATGGAGGTCGG | AATAACTCCAAGGGGCTCGC | 0.138770 CGTCATGAGATGGAGGTCGG | ATTGCGCTATGGAGTGGAGG | 0.144491 CCGTATGGATCCATGGGTCG | AATAACTCCAAGGGGCTCGC | 0.353322 CGTCATGAGATGGAGGTCGG | GGAGTGGAGGTGAAAGCAGG | 0.355589 CGTCATGAGATGGAGGTCGG | TGGCAATAACTCCAAGGGGC | | | | | |
| NCU00801T0 | 3 | 5 | NCU00801T0\_input.txt | NCU00801T0\_output.txt | 0.138049 TACCACCGATGAACATGCCC | TCAAAAAGCCTCAAGTGCGC | 0.205085 TACCACCGATGAACATGCCC | GAGCGTCATGTTGTGATGGC | 0.208764 CAACGACAAACTGAGCGAGC | GAGCGTCATGTTGTGATGGC | 0.286592 TACCACCGATGAACATGCCC | GTCATGTTGTGATGGCTGCG | 0.286592 TACCACCGATGAACATGCCC | CGTCATGTTGTGATGGCTGC | | | | | |
| NCU00802T0 | 2 | 5 | NCU00802T0\_input.txt | NCU00802T0\_output.txt | 1.013844 GCCTTTTTCAGCGTCATCCC | CGATCGTGAGCATCTTGTCG | 1.190677 GCACTGGTTTGGACATGTGC | TTTCCTGTCGTGAATTGCCG | 1.229852 CAGTTTGCGCTGGCTAAACC | CGATCGTGAGCATCTTGTCG | 1.260381 TGGTTTGGACATGTGCTTGC | TTTCCTGTCGTGAATTGCCG | 1.535888 GGTTTGGACATGTGCTTGCG | TTTCCTGTCGTGAATTGCCG | | | | | |
| NCU00803T0 | 4 | 5 | NCU00803T0\_input.txt | NCU00803T0\_output.txt | 0.711251 TCATCGCTTTTTGCTGCTGC | TTCGGGGGAATATGATCGGC | 0.711251 TGCTCATCGCTTTTTGCTGC | TTCGGGGGAATATGATCGGC | 1.133050 TGGAGCCGTTGAAAGAACCG | TGTAAGGAAGGTGCACACGG | 1.206556 TGGAGCCGTTGAAAGAACCG | TTCGGGGGAATATGATCGGC | 1.353268 TGGAGCCGTTGAAAGAACCG | GGAGTAGATGCCCAGGTTCC | | | | | |
| NCU00803T1 | 3 | 5 | NCU00803T1\_input.txt | NCU00803T1\_output.txt | 0.711251 TCATCGCTTTTTGCTGCTGC | TTCGGGGGAATATGATCGGC | 0.711251 TGCTCATCGCTTTTTGCTGC | TTCGGGGGAATATGATCGGC | 1.133050 TGGAGCCGTTGAAAGAACCG | TGTAAGGAAGGTGCACACGG | 1.206556 TGGAGCCGTTGAAAGAACCG | TTCGGGGGAATATGATCGGC | 1.353268 TGGAGCCGTTGAAAGAACCG | GGAGTAGATGCCCAGGTTCC | | | | | |
| NCU00804T0 | 3 | 5 | NCU00804T0\_input.txt | NCU00804T0\_output.txt | 3.109873 GCTCACCACGAATAGATTCAAGC | GTCTTGTCCTGTCGATGGGG | 3.245642 GCTCACCACGAATAGATTCAAGC | ACGTCTTGTCCTGTCGATGG | 3.324192 GCTCACCACGAATAGATTCAAGC | TGTGGCTGATAGGTTGGTGC | 3.366645 TCTTCTGCGGGAAAGCTCG | TCTTGTCCTGTCGATGGGG | 3.409302 ATCTTCTGCGGGAAAGCTCG | GACATGGAGCAGGATTTTCTCC | | | | | |
| NCU00805T0 | 4 | 5 | NCU00805T0\_input.txt | NCU00805T0\_output.txt | 0.343865 TTTGCGGAGAGGTGTAGACG | TGCAATGATAAGCTTGCGCC | 0.627564 TTTTGGCCAAGACGTTTGCG | TGCAATGATAAGCTTGCGCC | 1.028577 GTTTTGGCCAAGACGTTTGC | TGCAATGATAAGCTTGCGCC | 1.042359 TTTGCGGAGAGGTGTAGACG | ATAACACACGGTCGTAGCGC | 1.165986 TTTGGCCAAGACGTTTGCG | TGCAATGATAAGCTTGCGCC | | | | | |
| NCU00806T0 | 7 | 5 | NCU00806T0\_input.txt | NCU00806T0\_output.txt | 0.141624 TGATGAGAGGGGTAGGGTCG | AGAACAGTTTCAGGCCGAGG | 0.280997 TCAGGCCGAAGAAGGTTACG | AGAACAGTTTCAGGCCGAGG | 0.355831 TCGTAGCCAACAAGGTCAGG | AGAACAGTTTCAGGCCGAGG | 0.494096 TGATGAGAGGGGTAGGGTCG | CCCAGCATCATGGTCAATGC | 0.496592 AACAGTTGTTGCTGCTTGGG | AGAACAGTTTCAGGCCGAGG | | | | | |
| NCU00807T0 | 4 | 5 | NCU00807T0\_input.txt | NCU00807T0\_output.txt | 0.569044 TGGAAAGAACCAGCGTCTCG | AGCTCCGTATCGACAATGCC | 0.639306 TCGTTTCTCACCAGCGATGG | AGCTCCGTATCGACAATGCC | 0.698877 CAGCGATGGAAAGAACCAGC | AGCTCCGTATCGACAATGCC | 1.054478 CACCAGCGATGGAAAGAACC | AGCTCCGTATCGACAATGCC | 1.204751 TGGAAAGAACCAGCGTCTCG | TTGCACCAACGAAATAGGCC | | | | | |
| NCU00808T0 | 6 | 5 | NCU00808T0\_input.txt | NCU00808T0\_output.txt | 0.203880 GGATGAGTGGAGGACTGTGC | CCAAGAGAACGAGCATTGCG | 0.274952 ATGATAAGCGGTTCCCTGGC | CCAAGAGAACGAGCATTGCG | 0.283705 GGATGAGTGGAGGACTGTGC | TTCCCGCTTGAAGGATCTCG | 0.287124 GGATGAGTGGAGGACTGTGC | GCCAAGAGAACGAGCATTGC | 0.358197 ATGATAAGCGGTTCCCTGGC | GCCAAGAGAACGAGCATTGC | | | | | |
| NCU00809T0 | 2 | 5 | NCU00809T0\_input.txt | NCU00809T0\_output.txt | 0.637801 ACACAAAGTACTCGCCCTGG | GAACCAGTCGTTGACAGGGG | 0.711186 GTTCCGCCTCTACCTTGTCC | GAACCAGTCGTTGACAGGGG | 0.715770 GTTCCGCCTCTACCTTGTCC | AGAGCACCTGTGAACATGCC | 0.781672 TCTGCGGATGTCTCAATGGG | GAACCAGTCGTTGACAGGGG | 0.786255 TCTGCGGATGTCTCAATGGG | AGAGCACCTGTGAACATGCC | | | | | |
| NCU00810T0 | 2 | 5 | NCU00810T0\_input.txt | NCU00810T0\_output.txt | 0.426453 TATACGCGGTTCAGGTTGGG | TGTGAGGGAGGATTTGCTCG | 0.857462 TATACGCGGTTCAGGTTGGG | GGGTGTGAGGGAGGATTTGC | 1.328942 ATACGCGGTTCAGGTTGGG | TGTGAGGGAGGATTTGCTCG | 1.385141 AGTATACGCGGTTCAGGTTGG | TGTGAGGGAGGATTTGCTCG | 1.441647 GTACATGCTCCCTCCGTAGC | CCTCTGATTTGAAGGGGAGGC | | | | | |
| NCU00811T0 | 1 | 5 | NCU00811T0\_input.txt | NCU00811T0\_output.txt | 0.138545 TAGCACAAAGTGAGAGGGCG | GGCATCATCAAGAAACCGCC | 0.138545 TAGCACAAAGTGAGAGGGCG | GGGCATCATCAAGAAACCGC | 0.275381 CGGGGTTTCCTGTACCTACG | GGCATCATCAAGAAACCGCC | 0.275381 CGGGGTTTCCTGTACCTACG | GGGCATCATCAAGAAACCGC | 0.280265 GGATCCGATGGAGTGGTTGG | GGCATCATCAAGAAACCGCC | | | | | |
| NCU00812T0 | 2 | 5 | NCU00812T0\_input.txt | NCU00812T0\_output.txt | 0.628674 GCAAAGTGGGAATTCGGAGC | GCCTTGCCATTAGTCTTGCC | 0.710770 AGTGGGAATTCGGAGCTTCC | GCCTTGCCATTAGTCTTGCC | 0.915702 GAGTCGGAGGAGAGAAAGGC | GCCTTGCCATTAGTCTTGCC | 0.990012 ATAAACGGACGTGGTTGGGC | GGGAATCTCTTGGGTTGGGG | 1.200078 GGAGGAGAGAAAGGCTTGCG | GCCTTGCCATTAGTCTTGCC | | | | | |
| NCU00813T0 | 2 | 5 | NCU00813T0\_input.txt | NCU00813T0\_output.txt | 0.062776 AAACAAAAGCGTCGCCTTCC | CTTGAGGTTTTGGCAGTGGC | 0.062776 AAACAAAAGCGTCGCCTTCC | GCTTGAGGTTTTGGCAGTGG | 0.063179 CCAGTTTTGCAGGTTGACGG | CTTGAGGTTTTGGCAGTGGC | 0.063179 CCAGTTTTGCAGGTTGACGG | GCTTGAGGTTTTGGCAGTGG | 0.063179 CCCAGTTTTGCAGGTTGACG | CTTGAGGTTTTGGCAGTGGC | | | | | |
| NCU00814T0 | 4 | 5 | NCU00814T0\_input.txt | NCU00814T0\_output.txt | 0.283174 TGTAGGCATCTTCGTTCGGG | ATCTGACTTACGGCGTTGGG | 0.287319 ACCAGGTGGAAGAAGGTTGC | ATCTGACTTACGGCGTTGGG | 0.346299 TGTAGGCATCTTCGTTCGGG | CCTATCTCCGATGGTAGCGC | 0.354461 GACCCCAGATGTTGACCAGG | AATACTACATACCCGGCGGC | 0.498395 ACCAGGTGGAAGAAGGTTGC | AATACTACATACCCGGCGGC | | | | | |
| NCU00817T0 | 1 | 5 | NCU00817T0\_input.txt | NCU00817T0\_output.txt | 0.068728 ATGCAGAGGGTGGAAATGGG | GTTCCTCTGGTTCCCTCTGC | 0.070116 ATGCAGAGGGTGGAAATGGG | TCCCTCTGCTTTGCTCTTGG | 0.071978 ATGCAGAGGGTGGAAATGGG | CTTCCAAGCGCAAACCTTCC | 0.145584 AGGAACCAAGAAGCCAGAGC | ACATAGTCGCTGTCTTGGCC | 0.212595 CGATGTCAAGACAGGGGAGG | GTTCCTCTGGTTCCCTCTGC | | | | | |
| NCU00818T0 | 1 | 5 | NCU00818T0\_input.txt | NCU00818T0\_output.txt | 0.286220 AAGAAGTTGATGGGGAGGCG | TCACCAAACCCAGCCTAAGC | 0.288795 CTACGGTCTGCAGGGAATGG | TATAATGCGGCCTCCCAACC | 0.289475 GAGGTTGGGGTTGAGGAAGG | TCACCAAACCCAGCCTAAGC | 0.358961 GATCAGCTACGGTCTGCAGG | TATAATGCGGCCTCCCAACC | 0.430237 CTACGGTCTGCAGGGAATGG | ACCCAACCCCATCTTGAACG | | | | | |
| NCU00819T0 | 3 | 5 | NCU00819T0\_input.txt | NCU00819T0\_output.txt | 0.698238 CAGGTTTCGAAGCAGCTTCC | ATGTCTCTCGAACGAAGGCG | 0.840202 GACACTTGATGCTTAGCGGC | ATGCTTGCAAGGAGGATCCG | 0.911561 ACAGGCATCCAAATCCGAGG | TTCCGACGACATGATTCGGC | 0.984174 CAGGTTTCGAAGCAGCTTCC | ATGCTTGCAAGGAGGATCCG | 1.028364 ACAGGCATCCAAATCCGAGG | GTTCCGACGACATGATTCGG | | | | | |
| NCU00821T0 | 4 | 5 | NCU00821T0\_input.txt | NCU00821T0\_output.txt | 0.349997 GAAGTTGTTGAAAGCGGGGG | ACCTCTGGCTTGATGGTTCG | 0.581181 AGTGATGAGACCCTGCATGG | ACCTCTGGCTTGATGGTTCG | 0.871951 AGATGGCAGTGATGAGACCC | ACCTCTGGCTTGATGGTTCG | 1.059832 GAAGTTGTTGAAAGCGGGGG | CCTCTGGCTTGATGGTTCGG | 1.233731 GCATGGTCGAGTTGTCTTTGG | ACCTCTGGCTTGATGGTTCG | | | | | |
| NCU00822T0 | 3 | 5 | NCU00822T0\_input.txt | NCU00822T0\_output.txt | 0.578805 AGAGGGGTTGATGAAAGCGG | AGGTAGAGGTCCTGCATTGC | 0.578805 ATAGGGAAGGTGCTGTGTGC | AGGTAGAGGTCCTGCATTGC | 0.697141 AGAGGGGTTGATGAAAGCGG | CTTCGCGTAGGTAGAGGTCC | 0.697141 ATAGGGAAGGTGCTGTGTGC | CTTCGCGTAGGTAGAGGTCC | 0.865313 TCGGCAGGTTGATGTGAAGG | AGGTAGAGGTCCTGCATTGC | | | | | |
| NCU00823T0 | 3 | 5 | NCU00823T0\_input.txt | NCU00823T0\_output.txt | 0.564452 CAGTGTAGAGGCTGTCGACC | CCTTGACGGACTGAATGGGG | 0.564452 CAGTGTAGAGGCTGTCGACC | CCCTTGACGGACTGAATGGG | 0.772501 CAGTGTAGAGGCTGTCGACC | GAAAGCCAGCAGCCAAAACC | 0.894770 CGATGACTTTACCGTTCGCG | GAAAGCCAGCAGCCAAAACC | 0.989089 CAGTGTAGAGGCTGTCGACC | TCACAACCTTGCCCTTGACG | | | | | |
| NCU00824T0 | 4 | 5 | NCU00824T0\_input.txt | NCU00824T0\_output.txt | 0.352480 ATACGGTCATGACGCGATCC | CGTACGTAGGGGTATGCAGG | 0.360348 GCTTCTTGAAACGCAGGAGC | CCCCGCCAGTTCCTATTAGC | 0.365043 GCTTCTTGAAACGCAGGAGC | AGTTTATCCCCGCCAGTTCC | 0.421084 TTCTTGCCGGAACCGATACC | CGTACGTAGGGGTATGCAGG | 0.482641 AGAACGCGCTTCTTGAAACG | CCCCGCCAGTTCCTATTAGC | | | | | |
| NCU00825T0 | 2 | 5 | NCU00825T0\_input.txt | NCU00825T0\_output.txt | 1.316467 TGCCATAATTGGACCCGTCG | AAGGATATGCCCTTGGGACC | 1.387732 CCATAATTGGACCCGTCGGG | AAGGATATGCCCTTGGGACC | 1.505959 TGCCATAATTGGACCCGTCG | GCAAAGGATATGCCCTTGGG | 1.577224 CCATAATTGGACCCGTCGGG | GCAAAGGATATGCCCTTGGG | 1.680879 TGCCATAATTGGACCCGTCG | AAAGGATATGCCCTTGGGACC | | | | | |
| NCU00826T0 | 2 | 5 | NCU00826T0\_input.txt | NCU00826T0\_output.txt | 0.651896 GCAGGTTACCTCCTTCCTCG | GACATGGAAGGGGAGATGGG | 0.651896 GCAGGTTACCTCCTTCCTCG | GGACATGGAAGGGGAGATGG | 0.654979 ATCCATTCCTTCGTCAGCGG | GACATGGAAGGGGAGATGGG | 0.654979 ATCCATTCCTTCGTCAGCGG | GGACATGGAAGGGGAGATGG | 0.717819 ATCCATTCCTTCGTCAGCGG | AGTTAAAGTGGCGGTTGGGG | | | | | |
| NCU00827T0 | 1 | 5 | NCU00827T0\_input.txt | NCU00827T0\_output.txt | 0.428835 CCGTCTCTCTGATCGTTCCG | AGGGACACGGAAGAAAAGGG | 0.498970 CCGTTCTTCGTCGGTAGAGG | AGGGACACGGAAGAAAAGGG | 0.513439 TCCCCTTTTCTTCCGTGTCC | TTTTCTTGCCCTTCCCTCCC | 0.636550 CCGTCTCTCTGATCGTTCCG | GGGACACGGAAGAAAAGGGG | 0.649296 GCCGTCTCTCTGATCGTTCC | AGGGACACGGAAGAAAAGGG | | | | | |
| NCU00828T0 | 5 | 5 | NCU00828T0\_input.txt | NCU00828T0\_output.txt | 0.143480 AACCCACGCTACCAATCTGG | TGACGCTCGGGATTTTACCC | 0.214654 AACCCACGCTACCAATCTGG | GATTTGCTGGTTGACGCTCG | 0.214689 AACCCACGCTACCAATCTGG | CGACGATTTGCTGGTTGACG | 0.346594 GCCAGTCTCCGTGAAAAAGC | TGACGCTCGGGATTTTACCC | 0.417768 GCCAGTCTCCGTGAAAAAGC | GATTTGCTGGTTGACGCTCG | | | | | |
| NCU00829T0 | 1 | 5 | NCU00829T0\_input.txt | NCU00829T0\_output.txt | 0.631721 TGTGACGACAGTGGTCAAGG | TGTCATCACATACGAGCCGC | 0.837054 GATTGGTGCGCTGAGAAACG | ATTAGAATGGCTTCACGCGC | 1.065887 AAGGTGTTGCTCAAGGCTCC | TGTCATCACATACGAGCCGC | 1.066251 TGATGATGCCGCAAAGAGGG | TGACCACTGTCGTCACATCC | 1.108560 TGTGACGACAGTGGTCAAGG | ACCTTCGAGCCAGAGTTGC | | | | | |
| NCU00830T0 | 2 | 5 | NCU00830T0\_input.txt | NCU00830T0\_output.txt | 0.136943 AATCGGGGACATCATGACGG | TGGAATGTCGCGGAGTATCG | 0.143582 AATCGGGGACATCATGACGG | ACGGGCAAGAAGAGGAAAGG | 0.208303 ATTCAGGGGTCAATCGTCGG | TGGAATGTCGCGGAGTATCG | 0.210645 AATCGGGGACATCATGACGG | GATCTGAAGAGGCTGACGGG | 0.764548 AATCGGGGACATCATGACGG | GAAAGGATGGAATGTCGCGG | | | | | |
| NCU00831T0 | 4 | 5 | NCU00831T0\_input.txt | NCU00831T0\_output.txt | 0.616810 GTGGCTGTATCAGGTTTGCG | GTTGATGTTGAAGGCCTCGC | 0.754641 GTGGCTGTATCAGGTTTGCG | TAGTTGAGGTCGATAGCGCG | 1.042403 GTGGCTGTATCAGGTTTGCG | TGGACGTGTAGTTGAGGTCG | 1.060381 ATGTCACCTCAGTCCAGTCG | GTTGATGTTGAAGGCCTCGC | 1.093099 GATCTTAAGCAGACGTGGCG | GTTGATGTTGAAGGCCTCGC | | | | | |
| NCU00831T1 | 4 | 5 | NCU00831T1\_input.txt | NCU00831T1\_output.txt | 0.616810 GTGGCTGTATCAGGTTTGCG | GTTGATGTTGAAGGCCTCGC | 0.754641 GTGGCTGTATCAGGTTTGCG | TAGTTGAGGTCGATAGCGCG | 1.042403 GTGGCTGTATCAGGTTTGCG | TGGACGTGTAGTTGAGGTCG | 1.060381 ATGTCACCTCAGTCCAGTCG | GTTGATGTTGAAGGCCTCGC | 1.093099 GATCTTAAGCAGACGTGGCG | GTTGATGTTGAAGGCCTCGC | | | | | |
| NCU00832T0 | 3 | 5 | NCU00832T0\_input.txt | NCU00832T0\_output.txt | 0.623565 GGGGCTTCGGCGATATTAGG | CGAGTTTTCTGTGGTCGTGC | 0.837892 AAAGAAGAACGGGGCTTCGG | CGAGTTTTCTGTGGTCGTGC | 0.982552 AAAAGCAACTCCAAACCCCG | CGAGTTTTCTGTGGTCGTGC | 1.029230 GAAAGAAGAACGGGGCTTCG | CGAGTTTTCTGTGGTCGTGC | 1.118865 AACGTCCTTAGGTCAGACCG | CGAGTTTTCTGTGGTCGTGC | | | | | |
| NCU00833T0 | 3 | 5 | NCU00833T0\_input.txt | NCU00833T0\_output.txt | 0.351647 TTTGTCAAATGGGCTCGTGC | TCCGTGGTCTCGTAAAAGGC | 0.411691 CGCAAACTGCCTTACGATCC | TCCGTGGTCTCGTAAAAGGC | 0.413429 TTTATTCACATTCGCCGCGG | TCCGTGGTCTCGTAAAAGGC | 0.483820 CGCAAACTGCCTTACGATCC | GGGAGAATGAGATGGCCAGG | 0.485558 TTTATTCACATTCGCCGCGG | GGGAGAATGAGATGGCCAGG | | | | | |
| NCU00834T0 | 2 | 5 | NCU00834T0\_input.txt | NCU00834T0\_output.txt | 0.770521 TTCTTCTTGCACTTGTGCGC | CTGGTAAGGCGAGCAGAACC | 1.095873 TTCTTCTTGCACTTGTGCGC | ACGCATCTGTTGAGAAGGAGG | 1.161303 CGGCTAAAAAGGTCTTCGCG | ACGCATCTGTTGAGAAGGAGG | 1.245798 GTAGCCGTTTGCATGACACG | ACGCATCTGTTGAGAAGGAGG | 1.324724 TTCTTCTTGCACTTGTGCGC | TGGTAAGGCGAGCAGAACC | | | | | |
| NCU00835T0 | 2 | 5 | NCU00835T0\_input.txt | NCU00835T0\_output.txt | 0.430949 CACAGCTCTCATTGCATGCC | ACATTACTCCCCCAAACCGG | 0.439400 GATGGCTTTGGCGTTTGAGG | ACATTACTCCCCCAAACCGG | 0.496268 CACAGCTCTCATTGCATGCC | TGCATGCACATTACTCCCCC | 0.504718 GATGGCTTTGGCGTTTGAGG | TGCATGCACATTACTCCCCC | 0.509428 GGTCGTCATCGTCTCTGTGG | ACATTACTCCCCCAAACCGG | | | | | |
| NCU00836T0 | 4 | 5 | NCU00836T0\_input.txt | NCU00836T0\_output.txt | 0.359114 ACTCGCAGTGCTTGTAAGGG | CAAAAGTGTGAATGCCCCGG | 0.359114 ACTCGCAGTGCTTGTAAGGG | CCAAAAGTGTGAATGCCCCG | 0.438937 AGTGCTTGTAAGGGTGTCCC | CAAAAGTGTGAATGCCCCGG | 0.438937 AGTGCTTGTAAGGGTGTCCC | CCAAAAGTGTGAATGCCCCG | 0.713903 ACTCGCAGTGCTTGTAAGGG | GTAGGCCTTTCACTCCCAGC | | | | | |
| NCU00837T0 | 1 | 5 | NCU00837T0\_input.txt | NCU00837T0\_output.txt | 0.064622 GACTGTGTATCGCTCGAGGG | ACTCGGCCTCTTGGAATTGG | 0.133961 TGCTCGAGACTGTGTATCGC | ACTCGGCCTCTTGGAATTGG | 0.138323 GACTGTGTATCGCTCGAGGG | GTGGGTAGCTCAGTCGAACC | 0.202370 GACTGTGTATCGCTCGAGGG | CCCGAAGTAGTCCAACTCGG | 0.202370 GACTGTGTATCGCTCGAGGG | CCCCGAAGTAGTCCAACTCG | | | | | |
| NCU00838T0 | 1 | 5 | NCU00838T0\_input.txt | NCU00838T0\_output.txt | 0.224249 GCGTATGACTGGAGATCGGG | ACACCTCAGAAACCAACCCC | 0.224249 GGCGTATGACTGGAGATCGG | ACACCTCAGAAACCAACCCC | 0.288521 TATCCATCCTCTTGCACGCG | CCGATCTCCAGTCATACGCC | 0.288521 TATCCATCCTCTTGCACGCG | CCCGATCTCCAGTCATACGC | 0.428652 TATCCATCCTCTTGCACGCG | CGAAGACGACTACGACACCC | | | | | |
| NCU00839T0 | 2 | 5 | NCU00839T0\_input.txt | NCU00839T0\_output.txt | 0.133660 GCTATTGCTTGAAGCCAGCC | GAGATACGTTACCCCAGCGG | 0.133660 GCTATTGCTTGAAGCCAGCC | GGAGATACGTTACCCCAGCG | 0.133660 GCTATTGCTTGAAGCCAGCC | CGGAGATACGTTACCCCAGC | 0.138798 GCTATTGCTTGAAGCCAGCC | GCTTCTCGATATCCAGGGGC | 0.139376 CTGGCAGTAGCAACTACCCC | GAGATACGTTACCCCAGCGG | | | | | |
| NCU00840T0 | 3 | 5 | NCU00840T0\_input.txt | NCU00840T0\_output.txt | 0.569994 TGGCTTGTAGTCGGTTCTGC | TTATAGGCCGGTTAGCGTGC | 0.648237 TAGGGAGGAGTAAGCCTGGC | TTATAGGCCGGTTAGCGTGC | 0.720644 ATGACCTCGCTGTTATGGGG | TCCGGTTCAGCTTTGAAGGG | 0.921973 TTCGCTTCAAGCTTACCGGG | TTATAGGCCGGTTAGCGTGC | 1.034350 GTCGGTTCTGCTTGAAGACG | TTATAGGCCGGTTAGCGTGC | | | | | |
| NCU00841T0 | 2 | 5 | NCU00841T0\_input.txt | NCU00841T0\_output.txt | 0.414895 GCAATTCACCCGACTTACGC | TCTCAGAGGCAGTCAAACGG | 0.417576 GCAATTCACCCGACTTACGC | AAGCTGCTCAGTAGTGTGGC | 0.613770 GCAATTCACCCGACTTACGC | CAGTCAAACGGTGCCTAAGC | 0.687348 CAATTCACCCGACTTACGCG | TCTCAGAGGCAGTCAAACGG | 0.690029 CAATTCACCCGACTTACGCG | AAGCTGCTCAGTAGTGTGGC | | | | | |
| NCU00842T0 | 4 | 5 | NCU00842T0\_input.txt | NCU00842T0\_output.txt | 0.286389 GAAACGGAGTATCGGGGTCG | CGGATATTCCACCTACGCCC | 0.417274 CACGTACCTACTACCCAGCG | CGGATATTCCACCTACGCCC | 0.495457 TTAGCGACATGACACGAGGC | CGGATATTCCACCTACGCCC | 0.721758 TGGCCACGTACCTACTACCC | CGGATATTCCACCTACGCCC | 0.780701 CCACGTACCTACTACCCAGC | CGGATATTCCACCTACGCCC | | | | | |
| NCU00843T0 | 2 | 5 | NCU00843T0\_input.txt | NCU00843T0\_output.txt | 0.923802 CCCTAGGTCGTTTTCCTCGG | TGTGGGTTCCGTGAAACACC | 1.000082 GGGTCCCTAGGTCGTTTTCC | TGTGGGTTCCGTGAAACACC | 1.130362 AGCCGAAATTTTGCTGCAGC | GATCCCGAGTTCTCCAGTGG | 1.213181 GTGATTGGGTCCCTAGGTCG | TGTGGGTTCCGTGAAACACC | 1.327995 CCCTAGGTCGTTTTCCTCGG | TGGGTTCCGTGAAACACCC | | | | | |
| NCU00844T0 | 8 | 5 | NCU00844T0\_input.txt | NCU00844T0\_output.txt | 1.203179 ACCGAAAGCTTGAGCACAGG | GGTGTCGTTTGAACAAGGCG | 1.247397 ACTACCATTTCTCGGACAGGC | CTTGTCAAAGAACCCAGCGC | 1.247397 ACTACCATTTCTCGGACAGGC | GCTTGTCAAAGAACCCAGCG | 1.287084 ACCGAAAGCTTGAGCACAGG | AACTTTCTCCTTGTCCCCCG | 1.308728 ACTACCATTTCTCGGACAGGC | CGACTGATCTCCTCCAACCG | | | | | |
| NCU00845T0 | 2 | 5 | NCU00845T0\_input.txt | NCU00845T0\_output.txt | 0.141262 ACGCTATCATGGTAGGTGCG | GGCGTTGAATGCAAAGAGGG | 0.141681 ACGCTATCATGGTAGGTGCG | CGGAGGATGACGATGATCCG | 0.211275 ACGCTATCATGGTAGGTGCG | AGTAAGCGATTCGTGACGGG | 0.277124 ACGCTATCATGGTAGGTGCG | GGAGGATGACGATGATCCGG | 0.361726 ACGCTATCATGGTAGGTGCG | AGAGGGTTGAGTGAATGGGC | | | | | |
| NCU00846T0 | 6 | 5 | NCU00846T0\_input.txt | NCU00846T0\_output.txt | 0.071394 TTGCATCCACCAAAAAGCGG | TCAAGTTGATCACCCCAGCC | 0.072643 AAAGCGGTACCCTTCCATGG | TCAAGTTGATCACCCCAGCC | 0.142469 CCAGTATCCGGTACCACTGC | TCAAGTTGATCACCCCAGCC | 0.214845 CTTCCGTGACAGATCCTGGG | TCAAGTTGATCACCCCAGCC | 0.217166 ATGGCTTCCGTGACAGATCC | TCAAGTTGATCACCCCAGCC | | | | | |
| NCU00847T0 | 1 | 5 | NCU00847T0\_input.txt | NCU00847T0\_output.txt | 0.143510 ATGAAGTCAAGGGCGAGACG | ACACCAAGCACCTAACCTCG | 0.220253 ATGAAGTCAAGGGCGAGACG | TTCTTTCCCTTTCCCCACGG | 0.287638 ATGAAGTCAAGGGCGAGACG | ACCTCGATACTCTCCCGTCC | 0.289643 TTTGATAGTTGGGGCGAGGG | ACACCAAGCACCTAACCTCG | 0.433771 TTTGATAGTTGGGGCGAGGG | ACCTCGATACTCTCCCGTCC | | | | | |
| NCU00848T0 | 1 | 5 | NCU00848T0\_input.txt | NCU00848T0\_output.txt | 1.055180 ATTGGATACTAAGCGCGGCG | TTAGGAACCCCAACCAACCG | 1.337616 AATTGGATACTAAGCGCGGC | TTAGGAACCCCAACCAACCG | 1.346431 ATTGGATACTAAGCGCGGCG | AGGCCTGTCTGTTCCTTTCC | 1.628867 AATTGGATACTAAGCGCGGC | AGGCCTGTCTGTTCCTTTCC | 1.701394 TTGGATACTAAGCGCGGCG | TTAGGAACCCCAACCAACCG | | | | | |
| NCU00849T1 | 2 | 5 | NCU00849T1\_input.txt | NCU00849T1\_output.txt | 0.563063 CAAATTTGTCACCTCGGCGG | CACTCAGCAATGCGAAACCG | 0.570240 CAAATTTGTCACCTCGGCGG | GTTACCCTCTTGAGCCTCGC | 0.702909 CAAATTTGTCACCTCGGCGG | ACACTCAGCAATGCGAAACC | 1.005311 CAAATTTGTCACCTCGGCGG | TGTTACCCTCTTGAGCCTCG | 1.044452 ACAAATTTGTCACCTCGGCG | CACTCAGCAATGCGAAACCG | | | | | |
| NCU00849T0 | 3 | 5 | NCU00849T0\_input.txt | NCU00849T0\_output.txt | 0.359608 AAGAGCCTTGCCAACCTACC | AGACCGTCCCACCAATTTCG | 0.498769 AAGAGCCTTGCCAACCTACC | GTTACCCTCTTGAGCCTCGC | 0.706406 AAGAGCCTTGCCAACCTACC | TCCCACCAATTTCGTCGTCG | 0.933840 AAGAGCCTTGCCAACCTACC | TGTTACCCTCTTGAGCCTCG | 1.105026 AAGAGCCTTGCCAACCTACC | TAGACCGTCCCACCAATTTCG | | | | | |
| NCU00850T0 | 3 | 5 | NCU00850T0\_input.txt | NCU00850T0\_output.txt | 0.358459 TTCGAGGTACTTTTGGGGGC | AGACCGTCCCACCAATTTCG | 0.497620 TTCGAGGTACTTTTGGGGGC | GTTACCCTCTTGAGCCTCGC | 0.704648 TTCGAGGTACTTTTGGGGGC | TTCCGTTCTTGAGCTCAGCG | 0.705256 TTCGAGGTACTTTTGGGGGC | TCCCACCAATTTCGTCGTCG | 0.854330 GGACGTGAAGAGGAAGGAGG | AGACCGTCCCACCAATTTCG | | | | | |
| NCU00851T0 | 2 | 5 | NCU00851T0\_input.txt | NCU00851T0\_output.txt | 2.592184 ATCCAACGAGCTGCCTTTGC | CTGAAGATATTTTGGCGGCGG | 2.610918 ATCCAACGAGCTGCCTTTGC | TGAAGATATTTTGGCGGCGG | 2.858974 ATCCAACGAGCTGCCTTTGC | GGCTGAAGATATTTTGGCGGC | 3.187653 ATCCAACGAGCTGCCTTTGC | GCTGAAGATATTTTGGCGGCG | 3.263337 TCCAACGAGCTGCCTTTGC | CTGAAGATATTTTGGCGGCGG | | | | | |
| NCU00852T0 | 1 | 5 | NCU00852T0\_input.txt | NCU00852T0\_output.txt | 0.208904 TCATCAAGGGCTTCGTACGG | CCCCTGGACATATGTCGAGC | 0.213150 GCAAATGGCTCATCAAGGGC | CCCCTGGACATATGTCGAGC | 0.357509 CTCGACATATGTCCAGGGGC | CTAACCACTTTGGCCTCCCC | 0.357509 CTCGACATATGTCCAGGGGC | CCTAACCACTTTGGCCTCCC | 0.357509 CTCGACATATGTCCAGGGGC | CCCTAACCACTTTGGCCTCC | | | | | |
| NCU00853T0 | 1 | 5 | NCU00853T0\_input.txt | NCU00853T0\_output.txt | 0.487224 AGAGTGCCAATCGAAGTCCG | GCTATCTTTCAACCAGCCGC | 0.502423 AGAGTGCCAATCGAAGTCCG | TATCTTTCAACCAGCCGCCC | 0.574075 AGAGTGCCAATCGAAGTCCG | TGCCGAACATATTGAGCCCC | 0.629250 GTCAGGCAGGCTCATATGGG | GCTATCTTTCAACCAGCCGC | 0.629250 GGTCAGGCAGGCTCATATGG | GCTATCTTTCAACCAGCCGC | | | | | |
| NCU00854T0 | 6 | 5 | NCU00854T0\_input.txt | NCU00854T0\_output.txt | 0.140827 GTTGTCCAGTTGGTGCAACC | CGCGGGGTAGTGATGATACC | 0.281424 GTTGTCCAGTTGGTGCAACC | GAGCAAGTAGTCGGCTGAGG | 0.623543 GTTGTCCAGTTGGTGCAACC | CATCTTTGCGTGCTTACGGC | 0.623543 GTTGTCCAGTTGGTGCAACC | GCATCTTTGCGTGCTTACGG | 0.628962 GTTGTCCAGTTGGTGCAACC | CTACGTCAGTCTGGGTGTCC | | | | | |
| NCU00855T0 | 3 | 5 | NCU00855T0\_input.txt | NCU00855T0\_output.txt | 0.070643 TCCAGGTTTACGTCTGCACC | CCGGGGTATGTAAGGATGGC | 0.070643 TCCAGGTTTACGTCTGCACC | GCCGGGGTATGTAAGGATGG | 0.354758 TCCAGGTTTACGTCTGCACC | CGGGGTATGTAAGGATGGCG | 0.354758 TCCAGGTTTACGTCTGCACC | CATCGCCGGGGTATGTAAGG | 0.427568 CCCTCCAGGTTTACGTCTGC | CCGGGGTATGTAAGGATGGC | | | | | |
| NCU00856T0 | 1 | 5 | NCU00856T0\_input.txt | NCU00856T0\_output.txt | 0.347018 ACTAGCGATGCTCAACCAGG | GGCACCATAAAGCAACGACC | 0.348407 TGAATCCTTCCGGCACTAGC | GGCACCATAAAGCAACGACC | 0.349345 GATGCTCAACCAGGATCCCC | GGCACCATAAAGCAACGACC | 0.349483 ACTAGCGATGCTCAACCAGG | AGCAACGACCGAGTACATCC | 0.350871 TGAATCCTTCCGGCACTAGC | AGCAACGACCGAGTACATCC | | | | | |
| NCU00857T0 | 1 | 5 | NCU00857T0\_input.txt | NCU00857T0\_output.txt | 0.280755 TGTAGGGGTAAACGAGCACG | ATCCGACCAACTGACAACCC | 0.358851 GTTGGCCAAGAGTGTAGGGG | ATCCGACCAACTGACAACCC | 0.358851 GGTTGGCCAAGAGTGTAGGG | ATCCGACCAACTGACAACCC | 0.428331 ATGGGTTGTCAGTTGGTCGG | AGCAGCATCCAGACTACAGC | 0.496616 GGAGGGTCAGTACACGATGG | ATCCGACCAACTGACAACCC | | | | | |
| NCU00859T1 | 2 | 5 | NCU00859T1\_input.txt | NCU00859T1\_output.txt | 0.500406 AGGTTTTGATGACGGGGTCG | GGAGATCAAGTCGGGGAACG | 0.558796 AGGTTTTGATGACGGGGTCG | GAACGAAACTTGCGGGAAGG | 0.642862 AGGTTTTGATGACGGGGTCG | TGGAATCTGTGTGAAGCCGG | 0.711115 AGGTTTTGATGACGGGGTCG | TGCTGTAACGCTAGCTGTGG | 0.785852 AGGTTTTGATGACGGGGTCG | GGGAGATGGGTGCACTAAGC | | | | | |
| NCU00859T0 | 2 | 5 | NCU00859T0\_input.txt | NCU00859T0\_output.txt | 0.486975 TGAATCACGATGGAGAGCCG | TTGCGTTTCAGTTGGAAGCC | 0.716921 AGGGGTTGGAAGATGAAGCC | TTGCGTTTCAGTTGGAAGCC | 0.908014 TGAATCACGATGGAGAGCCG | CAGTTGGAAGCCATTCGACC | 0.982629 GAATCACGATGGAGAGCCGG | TTGCGTTTCAGTTGGAAGCC | 1.103300 TGAATCACGATGGAGAGCCG | ATTGCGTTTCAGTTGGAAGCC | | | | | |
| NCU00860T0 | 3 | 5 | NCU00860T0\_input.txt | NCU00860T0\_output.txt | 0.488426 GTCAGTCGGAATCGAGGACC | ATCCAAACAAACACGCCACC | 0.637054 GTCGGAATCGAGGACCATGG | ATCCAAACAAACACGCCACC | 0.714662 ACAAGGTGCTCAACAGGAGG | TGCATCAGACCGGACAAACC | 0.719181 AGCCCTGTGTTGTATTCCCC | ATCCAAACAAACACGCCACC | 0.770080 GAGGTCAGTCGGAATCGAGG | ATCCAAACAAACACGCCACC | | | | | |
| NCU00861T0 | 7 | 5 | NCU00861T0\_input.txt | NCU00861T0\_output.txt | 0.138122 GATAGAGAAGGTGACGGGCG | AACCAGTGTGTGTGAGGTGG | 0.138122 CGATAGAGAAGGTGACGGGC | AACCAGTGTGTGTGAGGTGG | 0.199036 GATAGAGAAGGTGACGGGCG | GGAACAGTCGAATTTGCGGG | 0.199036 CGATAGAGAAGGTGACGGGC | GGAACAGTCGAATTTGCGGG | 0.281289 GATAGAGAAGGTGACGGGCG | GAGGATGATGCAGAGCCAGG | | | | | |
| NCU00862T0 | 2 | 5 | NCU00862T0\_input.txt | NCU00862T0\_output.txt | 1.253059 CATCTCGTGCAGTGTTCTGG | TACTTTGCGGCCGTAGATGG | 1.395973 CATCTCGTGCAGTGTTCTGG | CAAGTTCAGGACCAGAGGGC | 1.395973 CATCTCGTGCAGTGTTCTGG | GCCAAGTTCAGGACCAGAGG | 1.400228 CATCTCGTGCAGTGTTCTGG | CCAAGTTCAGGACCAGAGGG | 1.785575 ACATCTCGTGCAGTGTTCTGG | TACTTTGCGGCCGTAGATGG | | | | | |
| NCU00863T0 | 1 | 5 | NCU00863T0\_input.txt | NCU00863T0\_output.txt | 0.283150 AGGATCTGAGAGTTGCTGCG | TTCTCTCGGGCGGAAAATCC | 0.354491 AGGATCTGAGAGTTGCTGCG | CGCAATCGTCCTCTTTTGGC | 0.412537 AGGATCTGAGAGTTGCTGCG | TGTTCACGCAAATCCGAAGC | 0.430774 GATGATGGTGACGGGGTAGG | TCTCGTCCAACATTGACCCG | 0.500925 GATGATGGTGACGGGGTAGG | TTCTCTCGGGCGGAAAATCC | | | | | |
| NCU00864T0 | 2 | 5 | NCU00864T0\_input.txt | NCU00864T0\_output.txt | 0.500035 AAATTGGGATCGTGGGTGGG | TGCTCACGGGAGATAACAGC | 1.196296 AAATTGGGATCGTGGGTGGG | GCTCACGGGAGATAACAGCG | 1.289715 AAATTGGGATCGTGGGTGGG | TTGAAATCGCCCCAAGTTGG | 1.395746 AAATTGGGATCGTGGGTGGG | AAGTTGGAGTGCTCACGGG | 1.482056 AATTGGGATCGTGGGTGGG | TGCTCACGGGAGATAACAGC | | | | | |
| NCU00865T0 | 1 | 5 | NCU00865T0\_input.txt | NCU00865T0\_output.txt | 0.692703 GGCGCGACAGAAGTTTTTGG | TGCAGAACCACAAATGCAGC | 0.698275 GGCGCGACAGAAGTTTTTGG | CAGAACCACAAATGCAGCCC | 0.719509 GAAGGATGAGGTCACCCAGG | TGCTCACGGGAGATAACAGC | 0.778825 GTTTGTGGGGAGACATTGGC | TGCTCACGGGAGATAACAGC | 0.781453 TCAGTGATGATGAGGAGGCG | TGCAGAACCACAAATGCAGC | | | | | |
| NCU00866T0 | 1 | 5 | NCU00866T0\_input.txt | NCU00866T0\_output.txt | 0.209282 AGGCCAAAAACAACGAACGG | TGTGCCATTCCGTCTCATCC | 0.211992 TTGGTCGTTTGGTGAGGTCC | GTTTTTGGCCTTTCCCGTCC | 0.212025 GGTGAGGTCCACGATGATGG | GTTTTTGGCCTTTCCCGTCC | 0.279901 TTGGTCGTTTGGTGAGGTCC | TCCCGTTCGTTGTTTTTGGC | 0.279934 GGTGAGGTCCACGATGATGG | TCCCGTTCGTTGTTTTTGGC | | | | | |
| NCU00867T0 | 2 | 5 | NCU00867T0\_input.txt | NCU00867T0\_output.txt | 0.216903 TTGGCTACTGCTTGACCTGG | TGGAAGGTACCCATGATGCG | 0.358472 GTTCCCCAAGATCGAGGACC | TGGAAGGTACCCATGATGCG | 0.359332 GTCTCACCATGACGACGAGG | TGGAAGGTACCCATGATGCG | 0.427907 CTTCGAGGAGACCCACAAGG | TGGAAGGTACCCATGATGCG | 0.499501 GAGACCCACAAGGAGCATGG | TGTCTTGAAGGTGGTGACGG | | | | | |
| NCU00868T0 | 2 | 5 | NCU00868T0\_input.txt | NCU00868T0\_output.txt | 0.700288 GTGTCATCCGCGAATGTTGC | ACTGGCATACCGAATCAGGC | 0.772596 GTGTCATCCGCGAATGTTGC | CTGAGGACTGTGGTGATGGG | 0.896708 CATCGTACGTGATGTGTGCC | CTGAGGACTGTGGTGATGGG | 0.896708 CCATCGTACGTGATGTGTGC | CTGAGGACTGTGGTGATGGG | 0.913436 GTGTCATCCGCGAATGTTGC | GCTGAGGACTGTGGTGATGG | | | | | |
| NCU00869T0 | 1 | 5 | NCU00869T0\_input.txt | NCU00869T0\_output.txt | 0.427481 TAGTAAAAGGCCTGCGTGGG | TTCTTCGCATGCTCACTCCC | 0.428013 TAGTAAAAGGCCTGCGTGGG | CACTCCCAGCAGATGTCACC | 0.441406 TAGTAAAAGGCCTGCGTGGG | AGGACACCAACAATCAGGGG | 0.514200 AGGCTGCTGTAGTAGTTGCG | AGGACACCAACAATCAGGGG | 0.556416 AGGCTGCTGTAGTAGTTGCG | CATTGCTACAGAACCCACGC | | | | | |
| NCU00870T0 | 3 | 5 | NCU00870T0\_input.txt | NCU00870T0\_output.txt | 0.267853 CACGGTGATGTGTTTCCACG | GAGGATGTGGATGCTCTCGG | 0.267853 CACGGTGATGTGTTTCCACG | GGAGGATGTGGATGCTCTCG | 0.485966 CACGGTGATGTGTTTCCACG | ATCTAATGGGGCGATTGGGC | 0.709194 TCGACAATACTGACAGCGCC | ATCTAATGGGGCGATTGGGC | 0.787473 CACGGTGATGTGTTTCCACG | AGGTGTGGCATCTAATGGGG | | | | | |
| NCU00871T0 | 1 | 5 | NCU00871T0\_input.txt | NCU00871T0\_output.txt | 0.148090 GTAAGGGCAGTCGTGGTACC | CTCGACCGAGCTCATCATCC | 0.212420 CAGTCGTGGTACCAGTAGGC | CTCGACCGAGCTCATCATCC | 0.212420 GCAGTCGTGGTACCAGTAGG | CTCGACCGAGCTCATCATCC | 0.287518 GTAAGGGCAGTCGTGGTACC | CTCATCATCCCCGACAACCC | 0.351848 CAGTCGTGGTACCAGTAGGC | CTCATCATCCCCGACAACCC | | | | | |
| NCU00872T0 | 3 | 5 | NCU00872T0\_input.txt | NCU00872T0\_output.txt | 0.480884 GCAGGACTGTCGCTAATTGC | GAAGAAGATGGCGAGGAGGG | 0.480884 GCAGGACTGTCGCTAATTGC | GGAAGAAGATGGCGAGGAGG | 0.624344 GCAGGACTGTCGCTAATTGC | CGAGGAAGAAGATGGCGAGG | 1.596945 GCAGGACTGTCGCTAATTGC | TGCCGAGGAAGAAGATGGC | 1.767815 GCAGGACTGTCGCTAATTGC | AGAGGCCGGTGAGGATAACG | | | | | |
| NCU00873T0 | 4 | 5 | NCU00873T0\_input.txt | NCU00873T0\_output.txt | 0.147386 ACCGAAAGCCGACTCTTACG | TTCTACCGTCAAAGTCCGCC | 0.217020 CCCTTGCCTTATCCACTCCG | TTCTACCGTCAAAGTCCGCC | 0.221706 CCCCTTGCCTTATCCACTCC | TTCTACCGTCAAAGTCCGCC | 0.368511 TACTGGGGCCACAATGAAGG | CCTGTCTGAACTTCCGGTCC | 0.368511 TACTGGGGCCACAATGAAGG | GGACCTGTCTGAACTTCCGG | | | | | |
| NCU00874T0 | 6 | 5 | NCU00874T0\_input.txt | NCU00874T0\_output.txt | 0.491034 TCAGCCAAGGATAACGTCGC | AATGCCGGTTTGTTGGTTCG | 0.695017 ACGTCGCACTTACTGTCTGC | AATGCCGGTTTGTTGGTTCG | 0.696034 TCAGCCAAGGATAACGTCGC | CCGGTTTGTTGGTTCGTAGC | 0.742017 CGCACTTACTGTCTGCTTCG | AATGCCGGTTTGTTGGTTCG | 0.780006 TCAGCCAAGGATAACGTCGC | TGTTGGTTCGTAGCTGAGCC | | | | | |
| NCU00874T1 | 6 | 5 | NCU00874T1\_input.txt | NCU00874T1\_output.txt | 0.491034 TCAGCCAAGGATAACGTCGC | AATGCCGGTTTGTTGGTTCG | 0.695017 ACGTCGCACTTACTGTCTGC | AATGCCGGTTTGTTGGTTCG | 0.696034 TCAGCCAAGGATAACGTCGC | CCGGTTTGTTGGTTCGTAGC | 0.742017 CGCACTTACTGTCTGCTTCG | AATGCCGGTTTGTTGGTTCG | 0.780006 TCAGCCAAGGATAACGTCGC | TGTTGGTTCGTAGCTGAGCC | | | | | |
| NCU00875T0 | 6 | 5 | NCU00875T0\_input.txt | NCU00875T0\_output.txt | 0.199835 GTCAGCGGACAGATACTGGG | GCTGTGCTCATTGAGAAGCG | 0.211201 GTCAGCGGACAGATACTGGG | TGCTCATTGAGAAGCGAGGG | 0.275712 ATACGATGCTTGGACGGTGG | GCTGTGCTCATTGAGAAGCG | 0.287077 ATACGATGCTTGGACGGTGG | TGCTCATTGAGAAGCGAGGG | 0.399518 GAAGATGCTCGATGTTGCGG | GCTGTGCTCATTGAGAAGCG | | | | | |
| NCU00876T0 | 1 | 5 | NCU00876T0\_input.txt | NCU00876T0\_output.txt | 0.147385 GTAGAGGATACGGCGGATGC | CGTCCTGTCACTCCACTTCC | 0.217294 GTTCTGGACATCTCGGACCG | CGTCCTGTCACTCCACTTCC | 0.358282 GTTCTGGACATCTCGGACCG | GAATTACGGGCAAAAGGGCG | 0.362553 GTAGAGGATACGGCGGATGC | GTCACTCCACTTCCCATCCC | 0.431222 GTAGAGGATACGGCGGATGC | GTCCTGTCACTCCACTTCCC | | | | | |
| NCU00877T0 | 1 | 5 | NCU00877T0\_input.txt | NCU00877T0\_output.txt | 0.145512 GGAGTTGAAGGGGAGTGAGC | CTGAACAATGCCTTCCACGC | 0.276860 GGAGTTGAAGGGGAGTGAGC | AATGCCTTCCACGCTTTTCG | 0.855442 CAAGGGAGTGAAGGATGCCG | CTGAACAATGCCTTCCACGC | 0.986790 CAAGGGAGTGAAGGATGCCG | AATGCCTTCCACGCTTTTCG | 1.067485 GTTGAAGGGGAGTGAGCAGC | CTGAACAATGCCTTCCACGC | | | | | |
| NCU00878T0 | 2 | 5 | NCU00878T0\_input.txt | NCU00878T0\_output.txt | 0.140451 TGCTACTATATCGCTGCGCC | CAAGCACAAACACTGCTCCC | 0.140451 TGCTACTATATCGCTGCGCC | CCAAGCACAAACACTGCTCC | 0.850009 TGCTACTATATCGCTGCGCC | AGTCCAAGCACAAACACTGC | 1.165708 CTACTATATCGCTGCGCCGC | CAAGCACAAACACTGCTCCC | 1.165708 CTACTATATCGCTGCGCCGC | CCAAGCACAAACACTGCTCC | | | | | |
| NCU00879T1 | 3 | 5 | NCU00879T1\_input.txt | NCU00879T1\_output.txt | 0.137668 ATCACGATCCACGATGACCG | CCCTAGAAAGGTGCCGATCC | 0.281938 ATCACGATCCACGATGACCG | TTGTGCCCTAGAAAGGTGCC | 0.287813 ATCACGATCCACGATGACCG | ATCCAATCACCAGTCTGGCC | 0.425125 ATCACGATCCACGATGACCG | AAGGTGCCGATCCAATCACC | 0.428903 CACCCACTTGAGCTTCTCCC | CCCTAGAAAGGTGCCGATCC | | | | | |
| NCU00879T0 | 2 | 5 | NCU00879T0\_input.txt | NCU00879T0\_output.txt | 0.137668 ATCACGATCCACGATGACCG | CCCTAGAAAGGTGCCGATCC | 0.281938 ATCACGATCCACGATGACCG | TTGTGCCCTAGAAAGGTGCC | 0.287813 ATCACGATCCACGATGACCG | ATCCAATCACCAGTCTGGCC | 0.425125 ATCACGATCCACGATGACCG | AAGGTGCCGATCCAATCACC | 0.428903 CACCCACTTGAGCTTCTCCC | CCCTAGAAAGGTGCCGATCC | | | | | |
| NCU00880T0 | 3 | 5 | NCU00880T0\_input.txt | NCU00880T0\_output.txt | 0.198400 GCTGCTGGAAAGCTAACTGC | TACGTGCTTATCGTCTCGCC | 0.273832 GCTGCTGGAAAGCTAACTGC | CTAGCACCAGTGAGCCTAGC | 0.277626 TGATGTAGCCGTGACCTTGG | TACGTGCTTATCGTCTCGCC | 0.336266 GCCAAAGTCGAACAGTGTGG | TACGTGCTTATCGTCTCGCC | 0.339671 AGTCGAACAGTGTGGTCTCG | TACGTGCTTATCGTCTCGCC | | | | | |
| NCU00881T0 | 3 | 5 | NCU00881T0\_input.txt | NCU00881T0\_output.txt | 0.072490 GGAAAGAACCCGTACCCTGG | TTGATGGTGTACAGGTCGGC | 0.072490 CCTGGAAAGAACCCGTACCC | TTGATGGTGTACAGGTCGGC | 0.626895 GGAAAGAACCCGTACCCTGG | ATTCGTTGACTTGGGCAACG | 0.626895 CCTGGAAAGAACCCGTACCC | ATTCGTTGACTTGGGCAACG | 0.629336 TGAGTTTCCCCATGGTCAGC | ATTCGTTGACTTGGGCAACG | | | | | |
| NCU00881T1 | 4 | 5 | NCU00881T1\_input.txt | NCU00881T1\_output.txt | 1.657708 TACAAGATTCCCTAGCGCGG | GGTCAAGTGGAAGGCTTTTGC | 1.929518 TACAAGATTCCCTAGCGCGG | GATTGTGGTCAAGTGGAAGGC | 2.008752 TACAAGATTCCCTAGCGCGG | ATTGTGGTCAAGTGGAAGGC | 2.210460 CTTGAGGTTGTCTGTGTGGC | GCAACAAACAGTACACTCGGG | 2.280604 GCGTTTATCTGGGAAGCAGG | GCAACAAACAGTACACTCGGG | | | | | |
| NCU00882T0 | 2 | 5 | NCU00882T0\_input.txt | NCU00882T0\_output.txt | 0.216657 CACATGGGTCATTTGCCTGC | ATTTCCCTCTCGCAGTTCCG | 0.284248 CAAAACCTGTGGCACATGGG | ACATCCACATCGCCATAGCC | 0.358987 CACATGGGTCATTTGCCTGC | ACATCCACATCGCCATAGCC | 0.429701 TCTTGCCTGTGATTGGACGG | ATTTCCCTCTCGCAGTTCCG | 0.467273 CAAAACCTGTGGCACATGGG | GACCACAAATGTCGACGTCG | | | | | |
| NCU00883T0 | 3 | 5 | NCU00883T0\_input.txt | NCU00883T0\_output.txt | 0.357132 CTGCTCGATATGGGAAGGGC | AGGAGCAACCGAGTGATTGG | 0.357132 GCTGCTCGATATGGGAAGGG | AGGAGCAACCGAGTGATTGG | 0.357132 GGCTGCTCGATATGGGAAGG | AGGAGCAACCGAGTGATTGG | 0.425810 ACTGGACGACGACTTTGACG | CTGCTCTTGGGGCTATCTGG | 0.430202 TCTTGGCTGCTCGATATGGG | AGGAGCAACCGAGTGATTGG | | | | | |
| NCU00884T0 | 3 | 5 | NCU00884T0\_input.txt | NCU00884T0\_output.txt | 0.215821 ATGTGGTGGGTAGCATGAGC | ACTTCACCTACGGCATCACG | 0.217011 CATGAGCAAGGCTAGGGAGG | ACTTCACCTACGGCATCACG | 0.360438 GAGAGAGGGGTTGCAGAAGG | ACTTCACCTACGGCATCACG | 0.486519 GAGAGAGGGGTTGCAGAAGG | CAAGTTCGCCGAAAGTGTCC | 0.502341 AGGATGCTTTGGAGAAGGCG | ACTTCACCTACGGCATCACG | | | | | |
| NCU00885T0 | 2 | 5 | NCU00885T0\_input.txt | NCU00885T0\_output.txt | 0.286825 CCGGTTGAGAGTGGGTATGG | GTACGTGGAAGGCTCACTCC | 0.570804 GGTTGAGAGTGGGTATGGCG | GTACGTGGAAGGCTCACTCC | 0.570804 CGGTTGAGAGTGGGTATGGC | GTACGTGGAAGGCTCACTCC | 0.784672 CCGGTTGAGAGTGGGTATGG | TCTTGGAGTACGTGGAAGGC | 0.993163 CCGGTTGAGAGTGGGTATGG | CTATTTTCAACACCCGGGCC | | | | | |
| NCU00886T0 | 3 | 5 | NCU00886T0\_input.txt | NCU00886T0\_output.txt | 0.353958 TATGACGAAGCCGACCAAGG | CCAAATTCGCATTTCGGGGG | 0.420351 TCGAGTGCTTCTCATGGTCG | TGTAGTGTTCATCGGCAGGG | 0.423965 TATGACGAAGCCGACCAAGG | TGTAGTGTTCATCGGCAGGG | 0.496580 TGGTCGAGTGCTTCTCATGG | TGTAGTGTTCATCGGCAGGG | 0.853647 AGGATATGACGAAGCCGACC | CCAAATTCGCATTTCGGGGG | | | | | |
| NCU00887T0 | 1 | 5 | NCU00887T0\_input.txt | NCU00887T0\_output.txt | 0.198327 GCTGTACTTTCTGCTGCTGC | ACAACAACATCAACAGCGGC | 0.280872 TCGAAACTGATGGCTCCTGG | ACAACAACATCAACAGCGGC | 0.416555 GCTGTACTTTCTGCTGCTGC | AAAAGAACGCAAAGCACCCG | 0.472171 CTGTACTTTCTGCTGCTGCG | ACAACAACATCAACAGCGGC | 0.500163 TGCACTTCTTCCATGGTGGG | AAAAGAACGCAAAGCACCCG | | | | | |
| NCU00889T0 | 3 | 5 | NCU00889T0\_input.txt | NCU00889T0\_output.txt | 0.422135 TGTTTGGGAGGAGAGAACGC | CAAGCTTGGCGTTTACTGCC | 0.560720 TCACGAAGCGATGAAGGAGG | CAAGCTTGGCGTTTACTGCC | 0.626066 TGTTTGGGAGGAGAGAACGC | TCAAGCTTGGCGTTTACTGC | 0.708538 TGCTTACTTTCCTGACCCGC | CAAGCTTGGCGTTTACTGCC | 0.710868 TACTTTCCTGACCCGCTTGG | CAAGCTTGGCGTTTACTGCC | | | | | |
| NCU00890T0 | 2 | 5 | NCU00890T0\_input.txt | NCU00890T0\_output.txt | 2.217051 GGGCTGTTAGGTAGTAGTCCC | CAAGGAGACGAAGCTCGAGG | 2.600679 GGTACCTTGATGGGCTGTTAGG | CAAGGAGACGAAGCTCGAGG | 2.629973 GAGGTAGCCAAGTTACCCGG | TTGGGGATGTTATGAAGGGC | 2.804237 TGGGCTGTTAGGTAGTAGTCCC | CAAGGAGACGAAGCTCGAGG | 2.922704 TGGGCTGTTAGGTAGTAGTCC | CAAGGAGACGAAGCTCGAGG | | | | | |
| NCU00891T0 | 2 | 5 | NCU00891T0\_input.txt | NCU00891T0\_output.txt | 0.282275 TCACCTCCGTCAACAACACC | CAGTAGTGGACATCGGAGCC | 0.286722 TCACCTCCGTCAACAACACC | GAGTTGATCTTGGGGACGGG | 0.286722 TCACCTCCGTCAACAACACC | GGAGTTGATCTTGGGGACGG | 0.286722 TCACCTCCGTCAACAACACC | GGGAGTTGATCTTGGGGACG | 0.287871 TCACCTCCGTCAACAACACC | AGATGCCGGTGTAGTTGACG | | | | | |
| NCU00892T0 | 3 | 5 | NCU00892T0\_input.txt | NCU00892T0\_output.txt | 0.708552 GGCACGCATTTCCTTTACCG | CTGTGAGGAGGCCATTTTGC | 0.791906 GGCACGCATTTCCTTTACCG | TGAGGAGGCCATTTTGCAGG | 0.855640 GGCACGCATTTCCTTTACCG | TGGTATGGCAAGTGCGAAGG | 0.907116 GCGATCGAGATCTTGGGAGG | GTTTCGCTCTTGTGGTGAGG | 0.907116 GAGGCGATCGAGATCTTGGG | GTTTCGCTCTTGTGGTGAGG | | | | | |
| NCU00892T1 | 4 | 5 | NCU00892T1\_input.txt | NCU00892T1\_output.txt | 0.506000 GCGATCGAGATCTTGGGAGG | AGATCTCTCTCCGGCTCACC | 0.506000 GAGGCGATCGAGATCTTGGG | AGATCTCTCTCCGGCTCACC | 0.576282 TACGTGGACGATGTTGAGGC | AGATCTCTCTCCGGCTCACC | 0.646464 GGCACGCATTTCCTTTACCG | AGATCTCTCTCCGGCTCACC | 0.986042 GGCACGCATTTCCTTTACCG | TACTGCGTTGTAGACCGAGG | | | | | |
| NCU00893T0 | 1 | 5 | NCU00893T0\_input.txt | NCU00893T0\_output.txt | 0.555482 CAAGGAACGAGGAAAAGGCG | TCTGAACCTGTTCCATGGCC | 0.700494 TCTACAACGCAGTAGAGCCG | ACTGTCTTGAGAACGCCACC | 0.700878 TCTACAACGCAGTAGAGCCG | TGAGAACGCCACCAAGAAGG | 0.767227 CAAGGAACGAGGAAAAGGCG | ACTGTCTTGAGAACGCCACC | 0.767611 CAAGGAACGAGGAAAAGGCG | TGAGAACGCCACCAAGAAGG | | | | | |
| NCU00894T0 | 2 | 5 | NCU00894T0\_input.txt | NCU00894T0\_output.txt | 0.430616 TCCCATATCCTCAGTTGCGC | AGAAGTCAGGGTCTCGGAGG | 0.718882 TCCCATATCCTCAGTTGCGC | TGGAGAAGTCAGGGTCTCGG | 0.801703 AAGGCCCTATACCATCCCCC | AGAAGTCAGGGTCTCGGAGG | 0.874821 TACCATCCCCCTCATGTTGC | AGAAGTCAGGGTCTCGGAGG | 0.967218 CATGTTGCGCACATCATTGC | AGAAGTCAGGGTCTCGGAGG | | | | | |
| NCU00895T0 | 4 | 5 | NCU00895T0\_input.txt | NCU00895T0\_output.txt | 0.216917 ACAGACCATTGCTCTCGACG | TTCCTTGACCCACGACTTGG | 0.356945 GCTACAAGTCTCTAGCGCCC | TTCCTTGACCCACGACTTGG | 0.501368 GCAACTGACCTCCATCCAGG | TTCCTTGACCCACGACTTGG | 0.555308 ACAGACCATTGCTCTCGACG | TATTCTCGTTTGCTTGGCGC | 0.695336 GCTACAAGTCTCTAGCGCCC | TATTCTCGTTTGCTTGGCGC | | | | | |
| NCU00896T0 | 3 | 5 | NCU00896T0\_input.txt | NCU00896T0\_output.txt | 3.338415 AGGAAAAGTACATGGGCCCG | AGATGGAGAGTAGTGTGATGGG | 3.413857 TCGTGGTCGTGAATCTGTCG | AGATGGAGAGTAGTGTGATGGG | 3.547845 ACTTGTCCTCATCGTGGTCG | AGATGGAGAGTAGTGTGATGGG | 3.666450 GTCGTGAATCTGTCGTTCGC | AGATGGAGAGTAGTGTGATGGG | 3.776910 AGGAAAAGTACATGGGCCCG | GATGGAGAGTAGTGTGATGGG | | | | | |
| NCU00897T0 | 2 | 5 | NCU00897T0\_input.txt | NCU00897T0\_output.txt | 0.420545 ATCTTACCGTCCCAACCAGC | ATCGAGGACACAGTTCGTCG | 0.425387 ATGTGTCTTCCTCCATGGCC | ATCGAGGACACAGTTCGTCG | 0.627237 TTACCAAAGATCCGTCGCCG | ATCGAGGACACAGTTCGTCG | 0.915271 GCCATCTTACCGTCCCAACC | ATCGAGGACACAGTTCGTCG | 1.172942 GTTACCAAAGATCCGTCGCC | ATCGAGGACACAGTTCGTCG | | | | | |
| NCU00898T0 | 2 | 5 | NCU00898T0\_input.txt | NCU00898T0\_output.txt | 1.187146 TGCATCATCTACGCGGAAGG | TACTCTCAACACGCACACGC | 1.327090 ATCATCTACGCGGAAGGTGG | TACTCTCAACACGCACACGC | 2.116656 CCGTACTTGGATTGCAAGGC | TTTTTCACATGCAGCCCGG | 2.116656 GCCGTACTTGGATTGCAAGG | TTTTTCACATGCAGCCCGG | 2.158229 AATGTGCATCATCTACGCGG | TACTCTCAACACGCACACGC | | | | | |
| NCU00899T0 | 3 | 5 | NCU00899T0\_input.txt | NCU00899T0\_output.txt | 0.208417 CTTGAAGCAGATTGGTGCCG | ACTGGATTGTGAAGCCCAGG | 0.210454 CTTGAAGCAGATTGGTGCCG | ACATTCCAACGCGGTTTTCG | 0.348887 CTTGAAGCAGATTGGTGCCG | GTTCGATGCTTGTCTTGCCG | 0.698573 CTTGAAGCAGATTGGTGCCG | TAATGGTGACGGGAATCGCG | 0.773634 CGCCTGGCAACTTTATCAGG | ACTGGATTGTGAAGCCCAGG | | | | | |
| NCU00900T0 | 3 | 5 | NCU00900T0\_input.txt | NCU00900T0\_output.txt | 0.287821 GAAGGAATGTCGGGGATGGG | ATTCGTCTGTGGCCAGTACG | 0.350553 TTGCTTGCCTTCTCAATGCG | ATTCGTCTGTGGCCAGTACG | 0.356898 TCTCAATGCGCGAGACTACG | ATTCGTCTGTGGCCAGTACG | 0.492690 GAAGGAATGTCGGGGATGGG | TTGCAAATTCGTCTGTGGCC | 0.507468 ATCTAGAGGTGGGGGAACGG | ATTCGTCTGTGGCCAGTACG | | | | | |
| NCU00901T0 | 2 | 5 | NCU00901T0\_input.txt | NCU00901T0\_output.txt | 0.343869 CTACTACTGGCGAGCTTCCG | ACTTGAGGTCTTCGTGACGG | 0.563416 GCTACTACTGGCGAGCTTCC | ACTTGAGGTCTTCGTGACGG | 0.730870 CTACTACTGGCGAGCTTCCG | GCACGTTTCTAGTCACGACG | 0.771468 TCGTCGGTTACAATGGCTCG | ACTTGAGGTCTTCGTGACGG | 0.778676 CACCCCATTGACCCTCATCG | ACTTGAGGTCTTCGTGACGG | | | | | |
| NCU00902T0 | 3 | 5 | NCU00902T0\_input.txt | NCU00902T0\_output.txt | 0.217126 AGCGACATCATGTCCTGTGG | CGCACATGAAAGAAAGGGGC | 0.217126 AGCGACATCATGTCCTGTGG | GCCGCACATGAAAGAAAGGG | 0.217126 AGCGACATCATGTCCTGTGG | GGCCGCACATGAAAGAAAGG | 0.287066 AGCGACATCATGTCCTGTGG | ATCCAGCGATGAACGGAAGG | 0.359366 ACATCATGTCCTGTGGGTCG | CGCACATGAAAGAAAGGGGC | | | | | |
| NCU00903T0 | 4 | 5 | NCU00903T0\_input.txt | NCU00903T0\_output.txt | 1.523034 TCATCGTCCTGTTCGTAGGC | ACAACTCCCCAATGTTTTGCG | 1.628513 TCATCGTCCTGTTCGTAGGC | CGGAATTTGGACAACTCCCC | 1.994428 TCATCGTCCTGTTCGTAGGC | CACGGAATTTGGACAACTCCC | 2.001598 TCATCGTCCTGTTCGTAGGC | ACGGAATTTGGACAACTCCCC | 2.236644 TCATCGTCCTGTTCGTAGGC | CAACTCCCCAATGTTTTGCG | | | | | |
| NCU00904T0 | 3 | 5 | NCU00904T0\_input.txt | NCU00904T0\_output.txt | 0.486288 ACTGTTACTGGTGAGCACGG | GTTTCTGCATGCGAACGACC | 0.486786 ACTGTTACTGGTGAGCACGG | CAGGTTTCTGCATGCGAACG | 0.562381 GAAGCGTGCTGTGGAAATGG | GTTTCTGCATGCGAACGACC | 0.562879 GAAGCGTGCTGTGGAAATGG | CAGGTTTCTGCATGCGAACG | 0.842210 TTCTCCTTTACAGCGACGCC | GTTTCTGCATGCGAACGACC | | | | | |
| NCU00905T0 | 5 | 5 | NCU00905T0\_input.txt | NCU00905T0\_output.txt | 0.277376 TCATCTGGTGCTGGATCTGC | GACGCACATGGATGAGTTGC | 0.284741 TCATCTGGTGCTGGATCTGC | CAGGTCGGATGGGTTAGAGC | 0.570042 TCATCTGGTGCTGGATCTGC | CATGATCCGGTAGGCGATGG | 0.732550 TCATCTGGTGCTGGATCTGC | ATGGGTTAGAGCACATGGGG | 0.762435 CTTGTGCCCATCGCTATTGG | GACGCACATGGATGAGTTGC | | | | | |
| NCU00906T0 | 1 | 5 | NCU00906T0\_input.txt | NCU00906T0\_output.txt | 0.138373 CATCAGCATGCATCAACGGG | AGTGTCATAACAGGCGCTCC | 0.268524 CATCAGCATGCATCAACGGG | CCATAATCGCCGTCCAATGC | 0.279682 CATCAGCATGCATCAACGGG | TCATAACAGGCGCTCCATCG | 0.360335 ATCAACGGGTCTTAGTGCCC | AGTGTCATAACAGGCGCTCC | 0.421366 CATCAGCATGCATCAACGGG | TCCAGTGTCATAACAGGCGC | | | | | |
| NCU00907T0 | 1 | 5 | NCU00907T0\_input.txt | NCU00907T0\_output.txt | 0.076120 CCATGCTCACTTTTTGCCCC | AGTAGCGTCCATGATAGCGC | 0.076120 CCCATGCTCACTTTTTGCCC | AGTAGCGTCCATGATAGCGC | 0.132763 CTCTCGAGTTTCTCCGACCG | TGCTCTTCCAACCGAATCCC | 0.134370 CTCTCGAGTTTCTCCGACCG | GGGCAAAAAGTGAGCATGGG | 0.134370 CTCTCGAGTTTCTCCGACCG | GGGGCAAAAAGTGAGCATGG | | | | | |
| NCU00911T0 | 4 | 5 | NCU00911T0\_input.txt | NCU00911T0\_output.txt | 0.420958 GACAACAAAGGCCAAGGACG | GAGGCATGAGGAGATTGGGG | 0.420958 GTCGACAACAAAGGCCAAGG | GAGGCATGAGGAGATTGGGG | 0.432380 ACCAGCATGAACAGAGCAGG | GAGGCATGAGGAGATTGGGG | 0.501764 AGAGCGTAAGTGCACCATGG | GAGGCATGAGGAGATTGGGG | 0.502833 AGCATGAACAGAGCAGGAGC | GAGGCATGAGGAGATTGGGG | | | | | |
| NCU00912T0 | 2 | 5 | NCU00912T0\_input.txt | NCU00912T0\_output.txt | 0.213068 CAGATCACCCAAGTCCGAGG | TCGGCAGTTCCAAGATGTCC | 0.427394 AGGCAGAACTGAGAAAGGCC | ACAAGCGGGAAGAAGTAGCG | 0.428849 TGGAGCGAAATGGCACTACC | TCGGCAGTTCCAAGATGTCC | 0.490989 CAGGCATGCAACAGATCACC | TCGGCAGTTCCAAGATGTCC | 0.566627 CAGATCACCCAAGTCCGAGG | ACAAGCGGGAAGAAGTAGCG | | | | | |
| NCU00913T1 | 2 | 5 | NCU00913T1\_input.txt | NCU00913T1\_output.txt | 0.133834 AAGTTTCCCAGCTGGTCTCG | CGGAAGAAGCAGCTGAATGC | 0.207973 CCTTCCAGTTTGTCATGCGC | CGGAAGAAGCAGCTGAATGC | 0.207973 GCCTTCCAGTTTGTCATGCG | CGGAAGAAGCAGCTGAATGC | 0.343098 GGCCTTCCAGTTTGTCATGC | CGGAAGAAGCAGCTGAATGC | 0.421936 AAGTTTCCCAGCTGGTCTCG | CTGCAAACGGAAGAAGCAGC | | | | | |
| NCU00913T0 | 2 | 5 | NCU00913T0\_input.txt | NCU00913T0\_output.txt | 0.070513 AAGTTTCCCAGCTGGTCTCG | AATGGCTTTGGAGGAGACGG | 0.074804 AAGTTTCCCAGCTGGTCTCG | GCTTCCTATCGGGTAGACGC | 0.144653 CCTTCCAGTTTGTCATGCGC | AATGGCTTTGGAGGAGACGG | 0.144653 GCCTTCCAGTTTGTCATGCG | AATGGCTTTGGAGGAGACGG | 0.148943 CCTTCCAGTTTGTCATGCGC | GCTTCCTATCGGGTAGACGC | | | | | |
| NCU00914T0 | 3 | 5 | NCU00914T0\_input.txt | NCU00914T0\_output.txt | 0.354676 TAGTCAAACAGCTCGCCTCC | GTTTGGCATGCATGAAGGGG | 0.495919 CTCCCGATGCATACTCGAGC | GTTTGGCATGCATGAAGGGG | 0.499172 TTCAGAATCGCAACCTCCCG | GTTTGGCATGCATGAAGGGG | 0.837731 GCTTCAGAATCGCAACCTCC | GTTTGGCATGCATGAAGGGG | 0.857496 ACCTCCCGCATGATCTTTGC | GTTTGGCATGCATGAAGGGG | | | | | |
| NCU00914T1 | 4 | 5 | NCU00914T1\_input.txt | NCU00914T1\_output.txt | 0.215803 AAGGGAGATAGCCAGCAAGC | TGAAGTCGTGGAAGTGAGGC | 0.424767 ATTCGGGAAACGTGAGAGGC | TGAAGTCGTGGAAGTGAGGC | 0.430675 AAGGGAGATAGCCAGCAAGC | GAAGTTGAGCGATGGCATGC | 0.494667 TGTGGCGTGACATATTCGGG | TGAAGTCGTGGAAGTGAGGC | 0.504307 AAGGGAGATAGCCAGCAAGC | GTGGAAGTGAGGCTCTTGGG | | | | | |
| NCU00915T0 | 5 | 5 | NCU00915T0\_input.txt | NCU00915T0\_output.txt | 0.705533 AAGATGTGAAGCAGGACGCC | TTGCTTTGCCGACTTCTTGC | 0.754620 GAAGATGTGAAGCAGGACGC | TTGCTTTGCCGACTTCTTGC | 0.783557 AAGATGTGAAGCAGGACGCC | GCCAAACTGCTACATTGGGC | 0.832643 GAAGATGTGAAGCAGGACGC | GCCAAACTGCTACATTGGGC | 0.846798 AAGATGTGAAGCAGGACGCC | CTGCTACATTGGGCTTGTGC | | | | | |
| NCU00916T2 | 6 | 5 | NCU00916T2\_input.txt | NCU00916T2\_output.txt | 0.914857 TCAGAGTCTTCTTGTCGGCG | AACGAATTGACCTGGGAGCG | 0.923422 TCAGAGTCTTCTTGTCGGCG | AATGATGGCTGCTGGACACC | 0.972907 CGTTGGAGGTTTGTTGTCGG | AGCGAAAACGTTGAAAGGGG | 0.980178 CGTTGGAGGTTTGTTGTCGG | AACGAATTGACCTGGGAGCG | 0.983063 GCGTTGGAGGTTTGTTGTCG | AGCGAAAACGTTGAAAGGGG | | | | | |
| NCU00916T1 | 6 | 5 | NCU00916T1\_input.txt | NCU00916T1\_output.txt | 0.914857 TCAGAGTCTTCTTGTCGGCG | AACGAATTGACCTGGGAGCG | 0.923422 TCAGAGTCTTCTTGTCGGCG | AATGATGGCTGCTGGACACC | 0.972907 CGTTGGAGGTTTGTTGTCGG | AGCGAAAACGTTGAAAGGGG | 0.980178 CGTTGGAGGTTTGTTGTCGG | AACGAATTGACCTGGGAGCG | 0.983063 GCGTTGGAGGTTTGTTGTCG | AGCGAAAACGTTGAAAGGGG | | | | | |
| NCU00916T0 | 6 | 5 | NCU00916T0\_input.txt | NCU00916T0\_output.txt | 0.914857 TCAGAGTCTTCTTGTCGGCG | AACGAATTGACCTGGGAGCG | 0.923422 TCAGAGTCTTCTTGTCGGCG | AATGATGGCTGCTGGACACC | 0.972907 CGTTGGAGGTTTGTTGTCGG | AGCGAAAACGTTGAAAGGGG | 0.980178 CGTTGGAGGTTTGTTGTCGG | AACGAATTGACCTGGGAGCG | 0.983063 GCGTTGGAGGTTTGTTGTCG | AGCGAAAACGTTGAAAGGGG | | | | | |
| NCU00917T0 | 2 | 5 | NCU00917T0\_input.txt | NCU00917T0\_output.txt | 0.210119 TACAGTCGTGGTTGTGGACG | TGAGTGCCAGGCATACATCG | 0.279664 TACAGTCGTGGTTGTGGACG | CATTTGCCGATTTCTCGCCC | 0.281203 GTTGGCGACGATTTGCTACG | GGCAGGGGTGATGTATCTCG | 0.495765 GTTGGCGACGATTTGCTACG | CATTTGCCGATTTCTCGCCC | 0.634891 GTCATGGTGCGAAATTCCCC | TGAGTGCCAGGCATACATCG | | | | | |
| NCU00917T1 | 3 | 5 | NCU00917T1\_input.txt | NCU00917T1\_output.txt | 0.559309 GCGTGCGATCGTTTATGTCG | TGAGTGCCAGGCATACATCG | 0.781980 AACTCGGTGTTGCTGTAGGC | TGAGTGCCAGGCATACATCG | 0.851525 AACTCGGTGTTGCTGTAGGC | CATTTGCCGATTTCTCGCCC | 1.139439 TTTGGCGGATCCTGTTGAGC | TGAGTGCCAGGCATACATCG | 1.183994 ACTCGGTGTTGCTGTAGGC | TGAGTGCCAGGCATACATCG | | | | | |
| NCU00918T0 | 1 | 5 | NCU00918T0\_input.txt | NCU00918T0\_output.txt | 0.215803 TAGTCGAGGTTGCCGATTGG | GAGTGCTTGTCACCAAAGCG | 0.429557 AGTGCGTGATGGATGACTGG | TGACCAACTGGCCTGATTCG | 0.486947 TAGTCGAGGTTGCCGATTGG | GGAGTGCTTGTCACCAAAGC | 0.496409 TAGTCGAGGTTGCCGATTGG | TGACCAACTGGCCTGATTCG | 0.578797 GACCTTTCCTGAAGCCTGGG | AAGACATCCCACCCAATCGG | | | | | |
| NCU00919T0 | 1 | 5 | NCU00919T0\_input.txt | NCU00919T0\_output.txt | 0.145205 GGAAGTTGAAGGTCAGGGGG | CCGCCTGTTATTCTCCCTCC | 0.145205 GGAAGTTGAAGGTCAGGGGG | CTCCCGCCTGTTATTCTCCC | 0.145205 GGAAGTTGAAGGTCAGGGGG | CCTCCCGCCTGTTATTCTCC | 0.287799 GGAAGTTGAAGGTCAGGGGG | CGCCTGTTATTCTCCCTCCG | 0.361612 GGAAGTTGAAGGTCAGGGGG | GCTAGCCTCAATAGCCTCCC | | | | | |
| NCU00920T0 | 3 | 5 | NCU00920T0\_input.txt | NCU00920T0\_output.txt | 0.213177 CCCTGCTGACGATACCTTCC | CGGAAGTCCATGACAGTCCC | 0.287100 CCCTGCTGACGATACCTTCC | CCATGACAGTCCCATCCTGG | 0.355273 CCCTGCTGACGATACCTTCC | TCATGGTCCTGCCTTCTTCG | 0.561261 CCCTGCTGACGATACCTTCC | TTGAGCTCGATCGGAAGTCC | 0.777214 CGATACCTTCCTCCTCCACG | CGGAAGTCCATGACAGTCCC | | | | | |
| NCU00921T0 | 2 | 5 | NCU00921T0\_input.txt | NCU00921T0\_output.txt | 1.066438 AGGTAACGTCTTATCGGCGC | GGGGAAAGAGGAAAATGCGG | 1.066438 AGGTAACGTCTTATCGGCGC | GGGGGAAAGAGGAAAATGCG | 1.818371 CTGTTTTGTTAAGTGGCGGGG | GGGGAAAGAGGAAAATGCGG | 1.818371 CTGTTTTGTTAAGTGGCGGGG | GGGGGAAAGAGGAAAATGCG | 1.851228 TGTTTTGTTAAGTGGCGGGG | GGGGAAAGAGGAAAATGCGG | | | | | |
| NCU00922T0 | 3 | 5 | NCU00922T0\_input.txt | NCU00922T0\_output.txt | 0.697323 CGCAGGACGCCAAAAATACG | GAGTGAGGTCTTGGCTAGGC | 0.788786 TTTTGGCTGTTGTTGTCGCC | ATGGCCACCTCATGTTTTGC | 0.900842 TTTTGGCTGTTGTTGTCGCC | CAACCGCATCTTCGTTCTCC | 0.985226 TTTTGGCTGTTGTTGTCGCC | TCGTTCTCCAGCGCAATAGC | 1.181202 GGACGCCAAAAATACGATCGG | GAGTGAGGTCTTGGCTAGGC | | | | | |
| NCU00923T0 | 3 | 5 | NCU00923T0\_input.txt | NCU00923T0\_output.txt | 0.285569 ATTTGCTTCGGAGGGTCAGG | CACCCCAAGAGTCACTAGGC | 0.578090 AGGGTCAGGTAGGGATTGGG | CACCCCAAGAGTCACTAGGC | 0.645549 TGGGGTATGTCAAATGCCGG | CACCCCAAGAGTCACTAGGC | 0.846596 GGATGGGTTGTTGTCGTTGG | CACCCCAAGAGTCACTAGGC | 0.911533 GTTGTCGTTGGACCTTGTGG | CACCCCAAGAGTCACTAGGC | | | | | |
| NCU00924T0 | 2 | 5 | NCU00924T0\_input.txt | NCU00924T0\_output.txt | 0.143712 GGTAACGTTGAGGAGGACGG | TGAAGATGGGCAGCAAGAGG | 0.217890 GGTAACGTTGAGGAGGACGG | GCAAGTTGAAGATGGGCAGC | 0.281952 AACAATGAATGGCGGCAAGG | TGAAGATGGGCAGCAAGAGG | 0.353551 GAGGACGGCTGGCTTATACG | TGAAGATGGGCAGCAAGAGG | 0.426048 GGTAACGTTGAGGAGGACGG | CGCCATCGTAAGGAGAGACG | | | | | |
| NCU00925T0 | 1 | 5 | NCU00925T0\_input.txt | NCU00925T0\_output.txt | 0.215496 TCCGAAACATCACCTCTGCC | CTTAGCCTTCCCGACTTCCG | 0.284426 TTTGAAGAAGGGGCTCGTGG | ATCAAGAAGAAGGGCCAGGC | 0.287159 TCCGAAACATCACCTCTGCC | AGAAGGTTTCCAAGTCGGCC | 0.429835 TTTGAAGAAGGGGCTCGTGG | CTTAGCCTTCCCGACTTCCG | 0.498613 TCCGAAACATCACCTCTGCC | GCTTAGCCTTCCCGACTTCC | | | | | |
| NCU00926T0 | 2 | 5 | NCU00926T0\_input.txt | NCU00926T0\_output.txt | 1.591910 CTGACGTACGAAAGCATCCC | TGGAAAACTGTTGCGGATGC | 1.717452 ACTGACGTACGAAAGCATCCC | TGGAAAACTGTTGCGGATGC | 1.724761 CGCACTGACGTACGAAAGC | TGGAAAACTGTTGCGGATGC | 1.967777 CACTGACGTACGAAAGCATCC | TGGAAAACTGTTGCGGATGC | 1.998814 AAGAACCGCACTGACGTACG | CACTACCATCTCTCTGAGGCG | | | | | |
| NCU00927T0 | 3 | 5 | NCU00927T0\_input.txt | NCU00927T0\_output.txt | 0.138240 CCTCATCAATTCGCAAGCGG | TAGAGTTGCTGTGCTCGTCG | 0.138240 CCCTCATCAATTCGCAAGCG | TAGAGTTGCTGTGCTCGTCG | 0.152183 GCGGTTATGGATTCGAACGC | TAGAGTTGCTGTGCTCGTCG | 0.214590 TCGGCTCCCTCATCAATTCG | TAGAGTTGCTGTGCTCGTCG | 0.221619 GCGGTTATGGATTCGAACGC | AGCATCGACTACACGAACCG | | | | | |
| NCU00928T0 | 1 | 5 | NCU00928T0\_input.txt | NCU00928T0\_output.txt | 0.435795 TTCGTTGGTCGTCTGATGGG | TACCTACCTAGTGCTGGCCC | 0.498684 TTCGTTGGTCGTCTGATGGG | ACGTAATCTGGAGCAGCTGC | 0.789149 TTCGTTGGTCGTCTGATGGG | GTACCTACCTAGTGCTGGCC | 0.789149 TTCGTTGGTCGTCTGATGGG | GGTACCTACCTAGTGCTGGC | 0.970794 TTCGTTGGTCGTCTGATGGG | CGCCTTCATGTGATTCGAGG | | | | | |
| NCU00929T0 | 1 | 5 | NCU00929T0\_input.txt | NCU00929T0\_output.txt | 0.216316 GTCTCGGTCTTGGTCTTGGG | TAGCTGCATCGGTACCTTGC | 0.216316 GGTCTCGGTCTTGGTCTTGG | TAGCTGCATCGGTACCTTGC | 0.288147 TTCTATTCGTCGTCGGGTGC | TGACCTTCCCGTCGATTTCG | 0.577254 AGTGTGACAGTAACAGGCCG | CTGGACCTCCATGACCTTCC | 0.579681 TTCTATTCGTCGTCGGGTGC | TCCCATCATCTGTCCCTCCC | | | | | |
| NCU00930T0 | 3 | 5 | NCU00930T0\_input.txt | NCU00930T0\_output.txt | 0.272065 CCAAAATGTTCGGACTCGGC | GCTGGTCCGAGATCTTCTGG | 0.278771 CCAAAATGTTCGGACTCGGC | GCGTCTCAAAGAACTTGGCG | 0.347225 CCAAAATGTTCGGACTCGGC | GGTCCGAGATCTTCTGGTGC | 0.406681 CCAAAATGTTCGGACTCGGC | GTCTCAAAGAACTTGGCGGC | 0.549373 CAAGGTTGTCGCTGAGATGC | GCTGGTCCGAGATCTTCTGG | | | | | |
| NCU00930T1 | 2 | 5 | NCU00930T1\_input.txt | NCU00930T1\_output.txt | 0.272065 CCAAAATGTTCGGACTCGGC | GCTGGTCCGAGATCTTCTGG | 0.278771 CCAAAATGTTCGGACTCGGC | GCGTCTCAAAGAACTTGGCG | 0.347225 CCAAAATGTTCGGACTCGGC | GGTCCGAGATCTTCTGGTGC | 0.406681 CCAAAATGTTCGGACTCGGC | GTCTCAAAGAACTTGGCGGC | 0.549373 CAAGGTTGTCGCTGAGATGC | GCTGGTCCGAGATCTTCTGG | | | | | |
| NCU00931T1 | 3 | 5 | NCU00931T1\_input.txt | NCU00931T1\_output.txt | 1.050627 CTTCCACTTCCAGGCGTACG | GATATGGGTGTCTCCGACGG | 1.050627 CTTCCACTTCCAGGCGTACG | GGATATGGGTGTCTCCGACG | 1.115369 GTGGTAGAAGCGCAGGTACG | GATATGGGTGTCTCCGACGG | 1.605258 TTCCACTTCCAGGCGTACG | GATATGGGTGTCTCCGACGG | 1.605258 TTCCACTTCCAGGCGTACG | GGATATGGGTGTCTCCGACG | | | | | |
| NCU00931T0 | 2 | 5 | NCU00931T0\_input.txt | NCU00931T0\_output.txt | 1.802692 ACAATCATGATGGTTGCCCC | GATATGGGTGTCTCCGACGG | 1.802692 ACAATCATGATGGTTGCCCC | GGATATGGGTGTCTCCGACG | 2.026042 ACAATCATGATGGTTGCCCC | GAGAGGAAACGGGAGAAGGG | 2.026042 ACAATCATGATGGTTGCCCC | GGAGAGGAAACGGGAGAAGG | 2.027907 ACAATCATGATGGTTGCCCC | GAGGTGGAGAAGAAGGGAGC | | | | | |
| NCU00932T0 | 2 | 5 | NCU00932T0\_input.txt | NCU00932T0\_output.txt | 0.193530 CCGTTACAAAAGCCTGCTCG | GGAATTGCCGGACGAAATCG | 0.221898 ACCTCTGTCAACACCCAAGC | TTTGCGCGTGTAGTTGAACG | 0.221901 TCTGTCAACACCCAAGCTGG | TTTGCGCGTGTAGTTGAACG | 0.259888 GCTCGACTTGACAAACACCG | GGAATTGCCGGACGAAATCG | 0.288150 ACCTCTGTCAACACCCAAGC | CCAAAGAGACCCATTTGCGC | | | | | |
| NCU00934T0 | 2 | 5 | NCU00934T0\_input.txt | NCU00934T0\_output.txt | 0.215750 TGCAATTGCCATTTTCCCCG | GTAAACATGGCGCGGAAAGG | 0.426394 TGCAATTGCCATTTTCCCCG | GGAAAGGCGACAATTTGGCC | 0.639642 TGCAATTGCCATTTTCCCCG | AGGAAAGGCGACAATTTGGC | 0.846607 TGCAATTGCCATTTTCCCCG | AGCTAGTAAACATGGCGCGG | 0.893057 CCGTTTCAAGATGTCGTCCG | GTAAACATGGCGCGGAAAGG | | | | | |
| NCU00935T0 | 3 | 5 | NCU00935T0\_input.txt | NCU00935T0\_output.txt | 0.282283 ATGGACTTCAGATCGACGGC | TGTCGCTGCATGACCATAGG | 0.353692 CGCTGAAGAAGAGGAGACCC | TGTCGCTGCATGACCATAGG | 0.859356 AGCCTTGAAGCTGCTGATGG | TGTCGCTGCATGACCATAGG | 0.913328 CGGCCTTGTCAGACTTCTCG | TGTCGCTGCATGACCATAGG | 1.167138 AAGAAGAGGAGACCCTTGCC | TGTCGCTGCATGACCATAGG | | | | | |
| NCU00936T2 | 4 | 5 | NCU00936T2\_input.txt | NCU00936T2\_output.txt | 0.483778 GCTACGTCAATGGCGAATGG | GGGTGGTGAGATCATCGTGG | 0.682962 GCTACGTCAATGGCGAATGG | CTTGTGCATCGAATTCGGGG | 0.694200 TGTTTGCTACGTCAATGGCG | GGGTGGTGAGATCATCGTGG | 0.893384 TGTTTGCTACGTCAATGGCG | CTTGTGCATCGAATTCGGGG | 0.934400 ATGGCGAATGGGTAAAGGCC | GGGTGGTGAGATCATCGTGG | | | | | |
| NCU00936T1 | 4 | 5 | NCU00936T1\_input.txt | NCU00936T1\_output.txt | 0.483778 GCTACGTCAATGGCGAATGG | GGGTGGTGAGATCATCGTGG | 0.682962 GCTACGTCAATGGCGAATGG | CTTGTGCATCGAATTCGGGG | 0.694200 TGTTTGCTACGTCAATGGCG | GGGTGGTGAGATCATCGTGG | 0.893384 TGTTTGCTACGTCAATGGCG | CTTGTGCATCGAATTCGGGG | 0.934400 ATGGCGAATGGGTAAAGGCC | GGGTGGTGAGATCATCGTGG | | | | | |
| NCU00936T0 | 3 | 5 | NCU00936T0\_input.txt | NCU00936T0\_output.txt | 0.483778 GCTACGTCAATGGCGAATGG | GGGTGGTGAGATCATCGTGG | 0.682962 GCTACGTCAATGGCGAATGG | CTTGTGCATCGAATTCGGGG | 0.694200 TGTTTGCTACGTCAATGGCG | GGGTGGTGAGATCATCGTGG | 0.893384 TGTTTGCTACGTCAATGGCG | CTTGTGCATCGAATTCGGGG | 0.934400 ATGGCGAATGGGTAAAGGCC | GGGTGGTGAGATCATCGTGG | | | | | |
| NCU00937T0 | 4 | 5 | NCU00937T0\_input.txt | NCU00937T0\_output.txt | 1.005237 GTAACCCCTGGAGAGGTTGC | AGTTCCAGTCCTTCTCGTGG | 1.140901 CAACAATGTGGGCATCGACC | AGTTCCAGTCCTTCTCGTGG | 1.277420 GTAACCCCTGGAGAGGTTGC | AGCATTCATGGCGAATAGGC | 1.293087 GTAACCCCTGGAGAGGTTGC | TCCAGTTCCAGTCCTTCTCG | 1.372603 GTAACCCCTGGAGAGGTTGC | CAGTTCCAGTCCTTCTCGTGG | | | | | |
| NCU00938T0 | 3 | 5 | NCU00938T0\_input.txt | NCU00938T0\_output.txt | 0.271733 AGAAGAACTGCCAGATCGCC | CAGTGACGTTGACGATTGGC | 0.271733 AGAAGAACTGCCAGATCGCC | GCAGTGACGTTGACGATTGG | 0.277447 CGGTCTGATCGTCAGTCTCG | AGAGGGCGAAGTTTGCTAGG | 0.418641 GGTACATTGCCAAGTCGTGC | AGAGGGCGAAGTTTGCTAGG | 0.498809 GTCTGATCGTCAGTCTCGGC | AGAGGGCGAAGTTTGCTAGG | | | | | |
| NCU00939T0 | 2 | 5 | NCU00939T0\_input.txt | NCU00939T0\_output.txt | 0.504400 TAGCGGATAGGTGGGAGAGG | GGAAAGCAGACGGAAGGAGG | 0.715511 TAGCGGATAGGTGGGAGAGG | AACGGAAAGCAGACGGAAGG | 0.719210 TAGCGGATAGGTGGGAGAGG | ATACCATACCATCGGTCGGC | 0.937915 TTGGGAGGACGATGGATTGG | GGAAAGCAGACGGAAGGAGG | 0.999736 GGGCCGAAAGAGAAAGAGGG | ATACCATACCATCGGTCGGC | | | | | |
| NCU00940T0 | 5 | 5 | NCU00940T0\_input.txt | NCU00940T0\_output.txt | 0.138457 TCGAGAACCTCAAAACCCGG | TTCCCGTATCTGCACATCGG | 0.500892 TCGAGAACCTCAAAACCCGG | TTGTGGTTGGTGGTGGTAGC | 0.505300 AAGTGCCAGGTGGATCAAGG | TTGTGGTTGGTGGTGGTAGC | 0.573077 GATCAAGGAGCCTCTGAGCC | TTGTGGTTGGTGGTGGTAGC | 0.573077 GGATCAAGGAGCCTCTGAGC | TTGTGGTTGGTGGTGGTAGC | | | | | |
| NCU00941T0 | 2 | 5 | NCU00941T0\_input.txt | NCU00941T0\_output.txt | 0.552748 CACAGCAGGGAGAGATTGGG | CCATTTCTCGCTGACTGTGC | 0.753196 CGAAAGAAGTGTGTGTGGGC | CCATTTCTCGCTGACTGTGC | 0.962752 GTGTTGATTGTCCTGTCGGC | CCATTTCTCGCTGACTGTGC | 1.113971 GAAAGAAGTGTGTGTGGGCC | CCATTTCTCGCTGACTGTGC | 1.141937 TGGTGGAGCCTAAAAGGTCC | CCATTTCTCGCTGACTGTGC | | | | | |
| NCU00943T0 | 3 | 5 | NCU00943T0\_input.txt | NCU00943T0\_output.txt | 0.275602 CTACTACCTTGACCGCTCGC | GTTGGTTGTTGTCGACGTGG | 0.553275 GGATTTGTTCCCAACGGAGC | GTTGGTTGTTGTCGACGTGG | 1.022088 AGAAGGAATGGGAGTGGTGG | GTTGGTTGTTGTCGACGTGG | 1.358201 CTCTACTACCTTGACCGCTCG | GTTGGTTGTTGTCGACGTGG | 1.382783 AAGAAGGAATGGGAGTGGTGG | GTTGGTTGTTGTCGACGTGG | | | | | |
| NCU00944T0 | 3 | 5 | NCU00944T0\_input.txt | NCU00944T0\_output.txt | 0.145674 TGCAAGAAGATCCCACCACC | TAGGCATGACAGGCTTGAGC | 0.146764 CGTTTTGTCAGGCACCATGG | TAGGCATGACAGGCTTGAGC | 0.425628 GTTTTGTCAGGCACCATGGG | TAGGCATGACAGGCTTGAGC | 0.437104 AAGAAGATCCCACCACCAGC | TAGGCATGACAGGCTTGAGC | 0.574430 ACCATGGGCAATCAGCTAGC | TAGGCATGACAGGCTTGAGC | | | | | |
| NCU00945T0 | 5 | 5 | NCU00945T0\_input.txt | NCU00945T0\_output.txt | 0.207624 GGAACTGCCATTCATGCAGC | CAATGGCCGAGAAAGCATCG | 0.348578 TTTCTGGTACAAGTCGGCCG | CAATGGCCGAGAAAGCATCG | 0.349457 GGAACTGCCATTCATGCAGC | GTGGTTAACGAGCAATGGCC | 0.351480 TCCAGCAGCTCTTCTTGACC | CAATGGCCGAGAAAGCATCG | 0.490412 TTTCTGGTACAAGTCGGCCG | GTGGTTAACGAGCAATGGCC | | | | | |
| NCU00946T0 | 3 | 5 | NCU00946T0\_input.txt | NCU00946T0\_output.txt | 1.065599 AGGCAGGATGTCATTGTCCG | TGGTGAGAAACATTTGCGGG | 1.146528 GTGGTGGTGAATTTGGCAGC | TCCTTCGCTTCATCTCTGGG | 1.238635 AGGCAGGATGTCATTGTCCG | CCCAGATGTTGACGTTGATGC | 1.269413 GAAGAACTACTTTGGCGCCG | TCCTTCGCTTCATCTCTGGG | 1.277905 GTGGTGGTGAATTTGGCAGC | TGGTGAGAAACATTTGCGGG | | | | | |
| NCU00947T0 | 2 | 5 | NCU00947T0\_input.txt | NCU00947T0\_output.txt | 0.487306 AATTTGCCCGCTACTTTGGC | AGGATTTCCAAAGCGTTGCG | 0.562244 AAGAACCACCCAATCGTCGG | AGGATTTCCAAAGCGTTGCG | 0.699750 AATTTGCCCGCTACTTTGGC | CTGGCGAGGATTTCCAAAGC | 0.774688 AAGAACCACCCAATCGTCGG | CTGGCGAGGATTTCCAAAGC | 0.842340 CACTCTCGAGAACTGGCAGC | TATCCTTCTCCGCCATGTGC | | | | | |
| NCU00948T0 | 4 | 5 | NCU00948T0\_input.txt | NCU00948T0\_output.txt | 1.130302 TACCGATACATACAGCGGCC | TCGGTGTCGAAATGAGGAGG | 1.267350 TACCGATACATACAGCGGCC | GGACAGTTGTCTGGACTCGC | 1.268265 TACCGATACATACAGCGGCC | CAGTTGTCTGGACTCGCTGG | 1.273543 TACCGATACATACAGCGGCC | TGGACAGTTGTCTGGACTCG | 1.415797 ACATTTCGGGCCCTTGATCG | GGACAGTTGTCTGGACTCGC | | | | | |
| NCU00949T0 | 3 | 5 | NCU00949T0\_input.txt | NCU00949T0\_output.txt | 0.287163 TCATTGGACAGTGGTGCAGG | AACAAGTGGGCTAAGGTCGG | 0.423124 TCTCGGGAGCCAATGTTTCG | CCAACGGCTGGTTTCTTTGG | 0.427436 TCTCGGGAGCCAATGTTTCG | AACAAGTGGGCTAAGGTCGG | 0.427999 TCATTGGACAGTGGTGCAGG | TGCACCGTAAGCATAGGTCC | 0.488356 TCATTGGACAGTGGTGCAGG | GTCCTTGTTGCACCGTAAGC | | | | | |
| NCU00950T0 | 2 | 5 | NCU00950T0\_input.txt | NCU00950T0\_output.txt | 0.428051 AATCGTTCTGGCTCCCATGG | GACGTTGTTGGTAGGGACCC | 0.492265 GCAAAATCGTTCTGGCTCCC | GACGTTGTTGGTAGGGACCC | 0.492265 GGCAAAATCGTTCTGGCTCC | GACGTTGTTGGTAGGGACCC | 0.630856 ATGGTGCCGATCTCGTATGG | TTTGGAAAATGTGACGGCCG | 0.639474 ATGGTGCCGATCTCGTATGG | GACGTTGTTGGTAGGGACCC | | | | | |
| NCU00951T0 | 3 | 5 | NCU00951T0\_input.txt | NCU00951T0\_output.txt | 0.283291 TTGGTGTCCTGCTTGATGGG | ACCCTCATGCGTTGTTTTGC | 0.353778 TTGGTGTCCTGCTTGATGGG | CGTGGGATAGGGACTTGTCG | 0.430359 TTGGTGTCCTGCTTGATGGG | CTGAGGCTGTAGTCCATGCC | 0.430394 TTGGTGTCCTGCTTGATGGG | GGGACTTGTCGAGGAGATGC | 0.500744 TTGGTGTCCTGCTTGATGGG | TATGCGCGGTCAGAGATTGG | | | | | |
| NCU00952T0 | 2 | 5 | NCU00952T0\_input.txt | NCU00952T0\_output.txt | 0.144677 TTCCACCGAGACTCCTTTGC | CCGCAAAGTTCGATTGGTGG | 0.213896 TTCCACCGAGACTCCTTTGC | ATCCACCTCCTCGACAATGC | 0.275454 GTATCAACGCGCTTCAACCC | ATCCACCTCCTCGACAATGC | 0.426144 CGTATCAACGCGCTTCAACC | ATCCACCTCCTCGACAATGC | 0.519208 TTCCTCTCCTTCCTCCCTCC | ATCCACCTCCTCGACAATGC | | | | | |
| NCU00953T0 | 2 | 5 | NCU00953T0\_input.txt | NCU00953T0\_output.txt | 2.269642 CATGGATCGTAGTCGGATCCC | TCCTTGTATCTGTCATCCGCG | 2.269642 CCATGGATCGTAGTCGGATCC | TCCTTGTATCTGTCATCCGCG | 2.346331 AGGTACCTGGTGTTGATCAGC | TCCTTGTATCTGTCATCCGCG | 2.614021 ATGGATCGTAGTCGGATCCC | TCCTTGTATCTGTCATCCGCG | 2.674688 CGTAGTCGGATCCCACTTAGG | TCCTTGTATCTGTCATCCGCG | | | | | |
| NCU00954T0 | 3 | 5 | NCU00954T0\_input.txt | NCU00954T0\_output.txt | 0.622695 AAGATGTTGTTGCTTGCGGG | CGGTGAAAGTCCACAAGTGC | 0.637909 AAGATGTTGTTGCTTGCGGG | CCCCTGAGCCAATAGATCCG | 0.769387 AAGATGTTGTTGCTTGCGGG | GTGCAATCGCCGTTTTCTCC | 0.779541 AAGTTTTTGGAGGCCAGGGG | CGGTGAAAGTCCACAAGTGC | 0.794755 AAGTTTTTGGAGGCCAGGGG | CCCCTGAGCCAATAGATCCG | | | | | |
| NCU00955T0 | 5 | 5 | NCU00955T0\_input.txt | NCU00955T0\_output.txt | 0.347516 AACACATCTTTTTGCCCGCG | GTGAAAACACTGCCGTGACC | 0.418719 TGGCGAAGTGATCAAGGTCG | GTGAAAACACTGCCGTGACC | 0.425433 AACACATCTTTTTGCCCGCG | AGGGCAGATGGTCTTGATGC | 0.565016 TTGCGCCATTGCTAACATGC | AGGGCAGATGGTCTTGATGC | 0.628536 TTGCGCCATTGCTAACATGC | GTTGTACGGGGCATGAAAGC | | | | | |
| NCU00956T0 | 2 | 5 | NCU00956T0\_input.txt | NCU00956T0\_output.txt | 0.070069 CTGTCCGGACCATGATAGCC | ATCATGGTAGGGCTATGCGC | 0.070069 CCTGTCCGGACCATGATAGC | ATCATGGTAGGGCTATGCGC | 0.072033 CTGTCCGGACCATGATAGCC | CACAAGAAGCCACCCATTGC | 0.072033 CCTGTCCGGACCATGATAGC | CACAAGAAGCCACCCATTGC | 0.213076 CTGTCCGGACCATGATAGCC | AGTGACACAAGAAGCCACCC | | | | | |
| NCU00957T0 | 4 | 5 | NCU00957T0\_input.txt | NCU00957T0\_output.txt | 0.434963 ACCATCGGCGATCTTGAAGG | TGGGCTCCCTTCTTACATGC | 0.496302 ACACTGTAGTTCTCGTCCGC | TGGGCTCCCTTCTTACATGC | 0.498985 GTCCAGGTGTACGAGTCACC | TGGGCTCCCTTCTTACATGC | 0.505006 ACCATCGGCGATCTTGAAGG | TTCTGCCAGTGATCCTGTGG | 0.569028 GTCCAGGTGTACGAGTCACC | TTCTGCCAGTGATCCTGTGG | | | | | |
| NCU00958T0 | 2 | 5 | NCU00958T0\_input.txt | NCU00958T0\_output.txt | 0.820963 CTTCAGTTTCTACAGCGCGG | TCATGATAGTGTCTGCGGGC | 0.954397 CTTCAGTTTCTACAGCGCGG | CAACACACCACAGGAATCGC | 1.031043 CTTCAGTTTCTACAGCGCGG | GGCAATCAACACACCACAGG | 1.139385 CTGTTCACATGTGCCAGAGC | GTACAGGAAGAGGGTTGGGG | 1.139385 CTGTTCACATGTGCCAGAGC | GGTACAGGAAGAGGGTTGGG | | | | | |
| NCU00959T0 | 3 | 5 | NCU00959T0\_input.txt | NCU00959T0\_output.txt | 0.357191 CGACGTAGGTGTGAGGAAGG | ACTTAACAACCCACCCACCC | 0.417399 GTTAGCATCGATCTGTGCGC | ACTTAACAACCCACCCACCC | 0.434764 GGACGAGATCCTTGACGACG | ACTTAACAACCCACCCACCC | 0.658247 CTGGAGGTAGGGCTTGATGG | ACTTAACAACCCACCCACCC | 0.692297 CGACGTAGGTGTGAGGAAGG | GATCAACCGGCACAAAGTCC | | | | | |
| NCU00959T1 | 2 | 5 | NCU00959T1\_input.txt | NCU00959T1\_output.txt | 0.644230 AAAAGCAAGCAAGGGAAGCC | TCCGATAGCCAGAGCAATGC | 0.991259 AGACGAGGATGCGGAAAAGC | TCCGATAGCCAGAGCAATGC | 1.281610 TTGGCGGATGAGGTGAATGC | TCCGATAGCCAGAGCAATGC | 1.454742 TGAGGTGAATGCCAGACTACG | TCCGATAGCCAGAGCAATGC | 1.613482 TGCGGAAAAGCAAGCAAGG | TCCGATAGCCAGAGCAATGC | | | | | |
| NCU00960T0 | 3 | 5 | NCU00960T0\_input.txt | NCU00960T0\_output.txt | 0.284808 GCTTTACGCTTGACCAAGGC | ACGAGGTTCATTACTCCGCC | 0.341018 CTTTACGCTTGACCAAGGCG | ACGAGGTTCATTACTCCGCC | 0.352215 TACTGTGAGTTATGCGGGGC | ACGAGGTTCATTACTCCGCC | 0.503951 GCTTTACGCTTGACCAAGGC | TCCGCAATGGTGAAGATGGG | 0.560160 CTTTACGCTTGACCAAGGCG | TCCGCAATGGTGAAGATGGG | | | | | |
| NCU00961T0 | 4 | 5 | NCU00961T0\_input.txt | NCU00961T0\_output.txt | 0.143787 TCGAAGCTTCCATCGTTCCC | ACCAAAGAGTGAAGGAGCCG | 0.351661 AAACTGTACAGACCGACGGG | ACCAAAGAGTGAAGGAGCCG | 0.705985 TGCTTACTTACCTGCCTGCC | TTGAGCGCTACAGTTCTGCC | 0.710334 TGCTTACTTACCTGCCTGCC | CCTTCCAAGAACAGGCTCGG | 0.710334 TGCTTACTTACCTGCCTGCC | CCCTTCCAAGAACAGGCTCG | | | | | |
| NCU00962T0 | 3 | 5 | NCU00962T0\_input.txt | NCU00962T0\_output.txt | 0.286087 ACATGGGCAGTTGAGAGTGG | AGTAAACGTATCGAGCGGGC | 0.356292 AATGCTCACGGTCCATGAGG | AGTAAACGTATCGAGCGGGC | 0.365561 ACATGGGCAGTTGAGAGTGG | ACCAAGATCGGAAACTGGGG | 0.367907 ACATGGGCAGTTGAGAGTGG | CTGGGGCTATGATGGATGGG | 0.427881 GAGAGTGGTGGCTCGATTCC | AGTAAACGTATCGAGCGGGC | | | | | |
| NCU00962T1 | 4 | 5 | NCU00962T1\_input.txt | NCU00962T1\_output.txt | 0.286087 ACATGGGCAGTTGAGAGTGG | AGTAAACGTATCGAGCGGGC | 0.356292 AATGCTCACGGTCCATGAGG | AGTAAACGTATCGAGCGGGC | 0.365561 ACATGGGCAGTTGAGAGTGG | ACCAAGATCGGAAACTGGGG | 0.367907 ACATGGGCAGTTGAGAGTGG | CTGGGGCTATGATGGATGGG | 0.427881 GAGAGTGGTGGCTCGATTCC | AGTAAACGTATCGAGCGGGC | | | | | |
| NCU00963T0 | 4 | 5 | NCU00963T0\_input.txt | NCU00963T0\_output.txt | 0.364842 TCTATGTCCCCAGCATTGGC | ATCAACCCTCGATGAAGGCC | 0.431990 CCAGCATCTATGTCCCCAGC | ATCAACCCTCGATGAAGGCC | 0.657256 AAGGCCAGCATCTATGTCCC | ATCAACCCTCGATGAAGGCC | 0.926121 CGGTTAAGGAGAAGGCCAGC | ATCAACCCTCGATGAAGGCC | 1.047119 CAAGAACTTTGACGGTCCGG | ATCAACCCTCGATGAAGGCC | | | | | |
| NCU00963T1 | 4 | 5 | NCU00963T1\_input.txt | NCU00963T1\_output.txt | 0.364842 TCTATGTCCCCAGCATTGGC | ATCAACCCTCGATGAAGGCC | 0.431990 CCAGCATCTATGTCCCCAGC | ATCAACCCTCGATGAAGGCC | 0.657256 AAGGCCAGCATCTATGTCCC | ATCAACCCTCGATGAAGGCC | 0.926121 CGGTTAAGGAGAAGGCCAGC | ATCAACCCTCGATGAAGGCC | 1.047119 CAAGAACTTTGACGGTCCGG | ATCAACCCTCGATGAAGGCC | | | | | |
| NCU00964T0 | 3 | 5 | NCU00964T0\_input.txt | NCU00964T0\_output.txt | 0.919808 TGCTGGCTCTTGTGTTCTCG | GGATGATCGACTGGGTAGGC | 1.099875 GAGAGTATCGGCCAGGAAGC | TGACAAGGTGACAGAGTGACG | 1.260473 TGCTGGCTCTTGTGTTCTCG | CGTCAAGGCATGCATAGTGG | 1.272438 ATTGAGGCAGCCCAACTTCC | CGTCAAGGCATGCATAGTGG | 1.518379 TGCTGGCTCTTGTGTTCTCG | TGGGTAGGCAGGGGTAAAGG | | | | | |
| NCU00965T0 | 2 | 5 | NCU00965T0\_input.txt | NCU00965T0\_output.txt | 0.637242 CTTTGGCTTCACGTTGTGGG | TTGAGGTAGTTGACGAGGGC | 0.785616 CACTTTTCTCGCACATCGCC | AAGTGTGGGATTGCACGAGG | 0.785616 CCACTTTTCTCGCACATCGC | AAGTGTGGGATTGCACGAGG | 0.785818 CACTTTTCTCGCACATCGCC | TTGAGGTAGTTGACGAGGGC | 0.785818 CCACTTTTCTCGCACATCGC | TTGAGGTAGTTGACGAGGGC | | | | | |
| NCU00966T0 | 5 | 5 | NCU00966T0\_input.txt | NCU00966T0\_output.txt | 0.076799 TCCTTGGTCATGACACCTGC | CACAAGCAGACGAAAAGGCC | 0.076799 TCCTTGGTCATGACACCTGC | CCACAAGCAGACGAAAAGGC | 0.357415 TCCTTGGTCATGACACCTGC | TGACATCCCACAAGCAGACG | 0.361987 TCATGACACCTGCCTGAAGC | CACAAGCAGACGAAAAGGCC | 0.361987 TCATGACACCTGCCTGAAGC | CCACAAGCAGACGAAAAGGC | | | | | |
| NCU00967T0 | 3 | 5 | NCU00967T0\_input.txt | NCU00967T0\_output.txt | 0.353474 CAACTTGGGCTTTGCTTCCC | TATATGAGCGCGGCTTGTCC | 0.356600 GCTTTGCTTCCCCAATGTGG | TATATGAGCGCGGCTTGTCC | 0.571470 TGGTAGGATTCAACTGGGCG | TATATGAGCGCGGCTTGTCC | 0.832019 TGGTAGGATTCAACTGGGCG | AAAGGTCGTTTTCGTGCTCG | 0.920601 GCAACTTGGGCTTTGCTTCC | TATATGAGCGCGGCTTGTCC | | | | | |
| NCU00968T0 | 2 | 5 | NCU00968T0\_input.txt | NCU00968T0\_output.txt | 2.604653 AGGCGAATGGTCTTGTTTCC | CTGACGCTGAAGCTGAGACG | 2.615904 AGGCGAATGGTCTTGTTTCC | TGACGCTGAAGCTGAGACG | 2.762933 AAGACGGGAGGCAAAAATCG | GGTTGTCCAAAGTGAGTGACG | 2.956466 AAGGCGAATGGTCTTGTTTCC | CTGACGCTGAAGCTGAGACG | 2.957785 AGGCGAATGGTCTTGTTTCC | GTGATGCTGACGCTGAAGC | | | | | |
| NCU00968T1 | 1 | 5 | NCU00968T1\_input.txt | NCU00968T1\_output.txt | 1.056485 TTTCAAATCGGGACAAGCCG | CAAGGACAGATGGGGGTACG | 1.192719 TTTCAAATCGGGACAAGCCG | GACAGATGGGGGTACGATCG | 1.266346 TTTCAAATCGGGACAAGCCG | CTATTTGCATGCTGGCTGGG | 1.310711 GGGCTACGAGTGATATAGGCG | CAAGGACAGATGGGGGTACG | 1.313757 CTTTCAAATCGGGACAAGCCG | CAAGGACAGATGGGGGTACG | | | | | |
| NCU00968T2 | 2 | 5 | NCU00968T2\_input.txt | NCU00968T2\_output.txt | 1.943287 AGGTGTTCAAAGACGGGAGG | TGTGTGTTGTTTGGGTTGTCC | 1.994474 AGGTGTTCAAAGACGGGAGG | GGTTGTCCAAAGTGAGTGACG | 2.304601 AGGCGAATGGTCTTGTTTCC | ATGATGAGGGTGAGGATGGC | 2.370005 AGGCGAATGGTCTTGTTTCC | TGCTGATGATGAGGGTGAGG | 2.564298 AGGTGTTCAAAGACGGGAGG | ATGTGTGTTGTTTGGGTTGTCC | | | | | |
| NCU00969T0 | 3 | 5 | NCU00969T0\_input.txt | NCU00969T0\_output.txt | 1.168837 TCACCAGAGCGTTCTTGTCC | CGTTAGACTGCTCCTCCTTCC | 1.457341 CTCCTCCCACTTCTGCAACC | CGTTAGACTGCTCCTCCTTCC | 1.499298 TCACCAGAGCGTTCTTGTCC | AGACGTTAGACTGCTCCTCC | 1.516508 TCACCAGAGCGTTCTTGTCC | GTTAGACTGCTCCTCCTTCCC | 1.723585 CCACTTCTGCAACCACTTCG | CGTTAGACTGCTCCTCCTTCC | | | | | |
| NCU00970T0 | 2 | 5 | NCU00970T0\_input.txt | NCU00970T0\_output.txt | 1.091433 TGATGATGATGGAGGGGACG | GGGTTCGTGTGTGAATGTGC | 1.230107 TGATGATGATGGAGGGGACG | CAGCTGACGTGGGACTATGG | 1.286481 TGGAGGGGACGACTACAACG | GGGTTCGTGTGTGAATGTGC | 1.287162 AGGGGACGACTACAACGACC | GGGTTCGTGTGTGAATGTGC | 1.425156 TGGAGGGGACGACTACAACG | CAGCTGACGTGGGACTATGG | | | | | |
| NCU00971T0 | 4 | 5 | NCU00971T0\_input.txt | NCU00971T0\_output.txt | 0.280922 TTGTCCCCGAGATGATTGGC | GAGAAGAGTGGGTAGCACCG | 0.280922 TTGTCCCCGAGATGATTGGC | CGAGAAGAGTGGGTAGCACC | 0.282084 TGACATGATCGTTGTCCCCG | GAGAAGAGTGGGTAGCACCG | 0.282084 TGACATGATCGTTGTCCCCG | CGAGAAGAGTGGGTAGCACC | 0.407814 GATGATTGGCAGCGTCATCG | GAGAAGAGTGGGTAGCACCG | | | | | |
| NCU00972T0 | 2 | 5 | NCU00972T0\_input.txt | NCU00972T0\_output.txt | 0.823827 GTATAACATCGCGCGCTTGC | AGGGATAGAACGAGACCCCC | 1.036808 TCGTGGTATAACATCGCGCG | AGGGATAGAACGAGACCCCC | 1.074000 AGAGACTCGAGGCTGAACCC | AGGGATAGAACGAGACCCCC | 1.295053 CTTCGTGGTATAACATCGCGC | AGGGATAGAACGAGACCCCC | 1.306446 GTGGTATAACATCGCGCGC | AGGGATAGAACGAGACCCCC | | | | | |
| NCU00973T0 | 2 | 5 | NCU00973T0\_input.txt | NCU00973T0\_output.txt | 0.056826 ATGAGTCGCCAGAGTATGCG | CATTCGTAGCACGCTCAAGC | 0.056962 GCTACTGAGGATGAGTCGCC | CATTCGTAGCACGCTCAAGC | 0.139928 ATGAGTCGCCAGAGTATGCG | ACGATCCTCTGCTTGTTCGG | 0.140064 GCTACTGAGGATGAGTCGCC | ACGATCCTCTGCTTGTTCGG | 0.336367 GTCACCAAGCTTTTGACGGG | CATTCGTAGCACGCTCAAGC | | | | | |
| NCU00974T0 | 3 | 5 | NCU00974T0\_input.txt | NCU00974T0\_output.txt | 0.348665 TCGCTTTCTTCGAGTACGGG | TTGTTGTGCCTCCCAGAAGG | 0.435541 CTCCTATGCTTATCCCGCCG | TCAAGGTCCCCAATTCGTCC | 0.491488 CTCCTATGCTTATCCCGCCG | CCATGTGCCAATCGTCATGG | 0.577440 CCTCCTATGCTTATCCCGCC | TCAAGGTCCCCAATTCGTCC | 0.633386 CCTCCTATGCTTATCCCGCC | CCATGTGCCAATCGTCATGG | | | | | |
| NCU00974T1 | 2 | 5 | NCU00974T1\_input.txt | NCU00974T1\_output.txt | 0.072368 TGAATGTCGTCGACAAGGGG | AGCAGAAGGTTGGATGGAGC | 0.501351 TGAATGTCGTCGACAAGGGG | TGGATGGAGCGGGAAAATCG | 0.764524 TGAATGTCGTCGACAAGGGG | ATTAAGTCATGGCGTGCACG | 0.774483 GAATGTCGTCGACAAGGGGG | AGCAGAAGGTTGGATGGAGC | 0.783542 TGAATGTCGTCGACAAGGGG | CAGAAGGTTGGATGGAGCGG | | | | | |
| NCU00975T0 | 1 | 5 | NCU00975T0\_input.txt | NCU00975T0\_output.txt | 0.215298 AGTGCTTTGGACCGTTAGGG | TTGTTGTGCCTCCCAGAAGG | 0.282930 GGAGGTGAAGATGAGCGAGG | TTGTTGTGCCTCCCAGAAGG | 0.287130 CGAGGAAGTAGTTGCAGGGG | TTGTTGTGCCTCCCAGAAGG | 0.288031 GTCTGCTCTGAAGGTCGTCC | TTGTTGTGCCTCCCAGAAGG | 0.358970 GTCCAAGGTGGGGTGTATCC | CCTCGCTCATCTTCACCTCC | | | | | |
| NCU00976T0 | 4 | 5 | NCU00976T0\_input.txt | NCU00976T0\_output.txt | 3.682299 GCTGAAGTTGAAGCTAGGGC | ACTACCCGACGATAAAAGGC | 3.871992 AGGCTGAAGTTGAAGCTAGGG | ACTACCCGACGATAAAAGGC | 4.158048 GCTGAAGTTGAAGCTAGGGC | AGTACTACCCGACGATAAAAGGC | 4.335567 GGCTGAAGTTGAAGCTAGGG | ACTACCCGACGATAAAAGGC | 4.347742 AGGCTGAAGTTGAAGCTAGGG | AGTACTACCCGACGATAAAAGGC | | | | | |
| NCU00977T0 | 1 | 5 | NCU00977T0\_input.txt | NCU00977T0\_output.txt | 0.071069 TGGTAGGCTCAACTTGCTGG | TCGACAACTTCCCCCTTTCG | 0.141304 GAGGAAAGATGGTGGGAGGC | TCGACAACTTCCCCCTTTCG | 0.350719 TGGTAGGCTCAACTTGCTGG | CAACTTCCCCCTTTCGTTGC | 0.420954 GAGGAAAGATGGTGGGAGGC | CAACTTCCCCCTTTCGTTGC | 0.429711 TGACTTGCTGGCTTATCGGG | AGCAAGTTGAGCCTACCAGC | | | | | |
| NCU00978T0 | 2 | 5 | NCU00978T0\_input.txt | NCU00978T0\_output.txt | 0.421444 AGGGTGTCTCTCGAACATGC | AAGGTTGGATCGAGACGAGC | 0.483319 AACATGCCGACGAAAACACC | AAGGTTGGATCGAGACGAGC | 0.758584 GACGAAAACACCGTGAAGGG | AAGGTTGGATCGAGACGAGC | 0.914928 GGGTGTCTCTCGAACATGCC | AAGGTTGGATCGAGACGAGC | 1.162375 GTTTGAATGGTGTGGAACGGG | GATCACTGTGCACAACGTCG | | | | | |
| NCU00979T0 | 4 | 5 | NCU00979T0\_input.txt | NCU00979T0\_output.txt | 0.288672 AGTTGATCGCTGAGGGTTCC | GAAGCCCATATCCTCGTCGG | 0.288672 AGTTGATCGCTGAGGGTTCC | GACCGAAGCCCATATCCTCG | 0.352995 AGTTGATCGCTGAGGGTTCC | CCGGACAATGGTAGGAGACG | 0.358721 AGTTGATCGCTGAGGGTTCC | TATCCTCGTCGGACTCCTCC | 0.427326 GAGCAGACTTGAGTGTCCGG | GAAGCCCATATCCTCGTCGG | | | | | |
| NCU00980T0 | 2 | 5 | NCU00980T0\_input.txt | NCU00980T0\_output.txt | 0.144419 TGATCGGGGACTTGGTTTCG | CCTTCATCCGAACCCACTCC | 0.215754 TGATCGGGGACTTGGTTTCG | AAGTTGTCCACTGGCCTTCC | 0.277710 TGATCGGGGACTTGGTTTCG | CCACGTTTTCCTGGACATGC | 0.286727 AAGAATCCTGCGTGATCGGG | CCTTCATCCGAACCCACTCC | 0.420018 AAGAATCCTGCGTGATCGGG | CCACGTTTTCCTGGACATGC | | | | | |
| NCU00981T0 | 5 | 5 | NCU00981T0\_input.txt | NCU00981T0\_output.txt | 0.144519 GCTCCTCTTCGCTGATACCC | CGCGCAAAATATCTCGTCCG | 0.265118 GCTCCTCTTCGCTGATACCC | GCGCAAAATATCTCGTCCGG | 0.265118 GCTCCTCTTCGCTGATACCC | CCGCGCAAAATATCTCGTCC | 0.503898 ACGCTTGCTCTTGATCTGGG | CGCGCAAAATATCTCGTCCG | 0.580352 GACCTTGGGAATAGGGACCG | CGCGCAAAATATCTCGTCCG | | | | | |
| NCU00983T0 | 3 | 5 | NCU00983T0\_input.txt | NCU00983T0\_output.txt | 0.068391 TTCGGTTCGTCCACTTGAGG | AAGTATTCACGCCCACCAGG | 0.143576 GTCCACTTGAGGATGGTCGG | AAGTATTCACGCCCACCAGG | 0.207566 GGAGTAGGTTCGGTTCGTCC | AAGTATTCACGCCCACCAGG | 0.209601 AATTTGAAGGGCATGCTCGC | AAGTATTCACGCCCACCAGG | 0.284587 TTCGGTTCGTCCACTTGAGG | CTGGCTCCTGAGACTTTCCC | | | | | |
| NCU00984T1 | 3 | 5 | NCU00984T1\_input.txt | NCU00984T1\_output.txt | 0.444495 CTGAGCTGTTGGAGACTGGG | TCCCTCCCAAACACATTCCC | 0.444495 CCTGAGCTGTTGGAGACTGG | TCCCTCCCAAACACATTCCC | 0.662666 TTCTACGGGAAATGGCTGGG | TCCCTCCCAAACACATTCCC | 0.729927 CTGTTGGAGACTGGGAAGCC | TCCCTCCCAAACACATTCCC | 1.074260 ATTGAGCCCATCCTTGCTGC | AGGCAAGGACAATGATGGGG | | | | | |
| NCU00984T0 | 2 | 5 | NCU00984T0\_input.txt | NCU00984T0\_output.txt | 0.069671 GAGGGAAGGATGATAGCGGC | AGGCAAGGACAATGATGGGG | 0.073164 GAGGGAAGGATGATAGCGGC | AGGCGTGTTTGTAAGGAGGG | 0.355299 GAGGGAAGGATGATAGCGGC | TTCTGTTGAGGAGCTCGTGG | 0.364091 TGGGGTACTTGCTGATTGGG | AGGCAAGGACAATGATGGGG | 0.367584 TGGGGTACTTGCTGATTGGG | AGGCGTGTTTGTAAGGAGGG | | | | | |
| NCU00985T0 | 4 | 5 | NCU00985T0\_input.txt | NCU00985T0\_output.txt | 0.288950 CGCAGGCGTAGTAGTAGAGC | AGTAACGTATGACCGTCGCG | 0.361208 CGCAGGCGTAGTAGTAGAGC | TGTGGAAGGCGAGGTAAACG | 0.499678 CGCAGGCGTAGTAGTAGAGC | CAGACGACGGATGTGGAAGG | 0.846124 CGCAGGCGTAGTAGTAGAGC | AAGCTTTGTACGATGCTGCC | 1.046167 CGCAGGCGTAGTAGTAGAGC | TAGTAACGTATGACCGTCGCG | | | | | |
| NCU00986T0 | 4 | 5 | NCU00986T0\_input.txt | NCU00986T0\_output.txt | 0.076867 CTGCATTCCGGGCATATTGC | CCAGATTTTGTGCAGGTGCC | 0.289793 CTGCATTCCGGGCATATTGC | TTGCTGCTCAAGTTCCCTCC | 0.426514 CTGCATTCCGGGCATATTGC | CAGATTTTGTGCAGGTGCCG | 0.640228 TGATGGAGCGCTCAATGAGG | TCACGGGTATGGTCATTGCG | 0.714525 CTGCATTCCGGGCATATTGC | ACCAGATTTTGTGCAGGTGC | | | | | |
| NCU00987T0 | 5 | 5 | NCU00987T0\_input.txt | NCU00987T0\_output.txt | 0.134747 TCTGTGCGAGATCGAATGGG | CCATACTAGGGGACGAACGC | 0.148249 TCTCGAGCTCACGGTTTTCC | TAACCTGGCGCCATAATCCC | 0.212748 TCTGTGCGAGATCGAATGGG | TAACCTGGCGCCATAATCCC | 0.348275 GAGCTCACGGTTTTCCTTGC | TAACCTGGCGCCATAATCCC | 0.358947 TCTCGAGCTCACGGTTTTCC | AACGGTCGGTGTCTTGATGG | | | | | |
| NCU00988T0 | 3 | 5 | NCU00988T0\_input.txt | NCU00988T0\_output.txt | 0.850330 TGCGGGTTATGTAGCGTTGG | GTAGGACACGTTGAGGGAGC | 0.970594 CTTTATGTCCGCATCTCGCC | GTAGGACACGTTGAGGGAGC | 0.970594 CCTTTATGTCCGCATCTCGC | GTAGGACACGTTGAGGGAGC | 0.970594 GCCTTTATGTCCGCATCTCG | GTAGGACACGTTGAGGGAGC | 0.982931 TTTATGTCCGCATCTCGCCG | GTAGGACACGTTGAGGGAGC | | | | | |
| NCU00989T0 | 2 | 5 | NCU00989T0\_input.txt | NCU00989T0\_output.txt | 1.498465 GCCTCGATATCCTCCAGTCC | TTTGAAGAGCCAAGCCGAGG | 1.895418 GCCTCGATATCCTCCAGTCC | TTGAAGAGCCAAGCCGAGG | 2.306172 TTTCACTCTGGCCATTCCCC | GAGCCAGAATCAGAACAGACG | 2.416426 TTTCACTCTGGCCATTCCCC | AGCCAGAATCAGAACAGACG | 2.435563 GATTTCCACACTGCTTCGGC | GAGCCAGAATCAGAACAGACG | | | | | |
| NCU00990T0 | 3 | 5 | NCU00990T0\_input.txt | NCU00990T0\_output.txt | 0.426890 AGAACATGTTCTCGCCGAGG | CGGAGAGTCAAGCTATCGCC | 0.490772 AGAACATGTTCTCGCCGAGG | TGCGGAGAGTCAAGCTATCG | 0.558002 AGAACATGTTCTCGCCGAGG | CGCCATCCTTGACTTTGAGC | 0.568186 GCGGCTTGTGGGTAGATAGG | CGGAGAGTCAAGCTATCGCC | 0.611888 TACGTCCCGCTTTGAGTAGC | CAATGTGCTGTCGGTCTTCG | | | | | |
| NCU00992T1 | 4 | 5 | NCU00992T1\_input.txt | NCU00992T1\_output.txt | 0.285572 GACTGGAGGGAGAAGTTGGC | TTGAACCGGTTGCCAAATGC | 0.286172 ATGGATGAAAGACTGGGCGG | TAATGAGACGACGAACCGGC | 0.358651 ATGGATGAAAGACTGGGCGG | GGGAGGAGAAGAGTTGCTGG | 0.429359 GACTAGGGCCCGGTTAAAGG | TTGAACCGGTTGCCAAATGC | 0.488210 GAGAAGTTGGCGGAAGTTGC | TTGAACCGGTTGCCAAATGC | | | | | |
| NCU00992T0 | 3 | 5 | NCU00992T0\_input.txt | NCU00992T0\_output.txt | 0.285572 GACTGGAGGGAGAAGTTGGC | TTGAACCGGTTGCCAAATGC | 0.286172 ATGGATGAAAGACTGGGCGG | TAATGAGACGACGAACCGGC | 0.358651 ATGGATGAAAGACTGGGCGG | GGGAGGAGAAGAGTTGCTGG | 0.429359 GACTAGGGCCCGGTTAAAGG | TTGAACCGGTTGCCAAATGC | 0.488210 GAGAAGTTGGCGGAAGTTGC | TTGAACCGGTTGCCAAATGC | | | | | |
| NCU00993T0 | 3 | 5 | NCU00993T0\_input.txt | NCU00993T0\_output.txt | 0.707349 AGATCAGAATACCGCGGTCC | GTAGGCGAGGATCTGGAAGC | 0.777689 TCCGGCTCTGAATAGTTCGG | GTAGGCGAGGATCTGGAAGC | 0.924768 AGATCAGAATACCGCGGTCC | TTCCATGAGCGTCTGGAAGG | 0.995108 TCCGGCTCTGAATAGTTCGG | TTCCATGAGCGTCTGGAAGG | 0.995385 TCCGGCTCTGAATAGTTCGG | GGCGGAGGATGGATTCTTCC | | | | | |
| NCU00994T0 | 2 | 5 | NCU00994T0\_input.txt | NCU00994T0\_output.txt | 0.553327 TTCATCCGAGTTCACCGACG | GTTGCCGATATATGCTGCGG | 0.656022 GATGGCTTGTTGGGTTTGGG | TGAAACCCCAATAGCCCTGG | 0.656365 GATGGCTTGTTGGGTTTGGG | AGGTGAAACCCCAATAGCCC | 0.759304 CGCTCCTTTGTTTACCGTCG | ACTTGGGCAGAAGCAGTAGC | 0.765215 GATGGCTTGTTGGGTTTGGG | GTTGCCGATATATGCTGCGG | | | | | |
| NCU00995T0 | 2 | 5 | NCU00995T0\_input.txt | NCU00995T0\_output.txt | 1.082575 ACAGGTTCCGATTGACTCCG | ATCTAGAGTGGCCTGCTTGG | 1.099412 ACAGGTTCCGATTGACTCCG | CTGTCGTTGTCTCAACACCG | 1.138538 ACAGGTTCCGATTGACTCCG | AAAACCCCGGAACATGGTCG | 1.169466 CTTGTTTGCAACGGACGACC | CTGTCGTTGTCTCAACACCG | 1.219172 GGGTCGTGGTAGGTATGTCG | ATCTAGAGTGGCCTGCTTGG | | | | | |
| NCU00998T0 | 6 | 5 | NCU00998T0\_input.txt | NCU00998T0\_output.txt | 0.782551 TCATGGTCTCCTTGTTGCGG | AGCGAGCAGGAATTCGAAGG | 0.816008 TTGTTGCGGCAAAGTGTACC | CGTTTGTAGACGCAGGTTCG | 0.843502 TTGTTGCGGCAAAGTGTACC | AGCGAGCAGGAATTCGAAGG | 1.146444 AGGATGATGGTTTGCTGGCC | AGCGAGCAGGAATTCGAAGG | 1.259762 GTCATGGTCTCCTTGTTGCG | AGCGAGCAGGAATTCGAAGG | | | | | |
| NCU00999T0 | 2 | 5 | NCU00999T0\_input.txt | NCU00999T0\_output.txt | 1.314906 TTGTCGTCTCGTCTTGGTGG | AGGACATTTCCCAGCTCTTCC | 1.392078 GTCTCGTCTTGGTGGTAGGC | AGGACATTTCCCAGCTCTTCC | 1.427626 GAGGGTCGTCTTGGTAGAGG | TTTTACGGGCCATGTCTGGG | 1.453882 CGTCTCGTCTTGGTGGTAGG | AGGACATTTCCCAGCTCTTCC | 1.788015 TTGTCGTCTCGTCTTGGTGG | GGACATTTCCCAGCTCTTCC | | | | | |
| NCU01000T0 | 3 | 5 | NCU01000T0\_input.txt | NCU01000T0\_output.txt | 0.280626 GAAGGCTGGAGAGGTGTACG | TGTGTTCCGGGATTATGGGC | 0.357213 TTGCTCGAGGTTTCCAGTGG | TGTGTTCCGGGATTATGGGC | 0.429374 TGGTGGGATTTTGCTCGAGG | TGTGTTCCGGGATTATGGGC | 0.716049 AGGTGGTGGGATTTTGCTCG | TGTGTTCCGGGATTATGGGC | 0.850549 TTGCTCGAGGTTTCCAGTGG | AAAACTGCCTGAGGTCGACG | | | | | |
| NCU01000T1 | 2 | 5 | NCU01000T1\_input.txt | NCU01000T1\_output.txt | 0.143093 GGAGTGGTCGAATGGGAAGG | TTGTTGGAGAGGATTCCGCC | 0.370115 TGGGAAGGTGGGCTTATTGG | TTGTTGGAGAGGATTCCGCC | 0.432553 GAAGGTGGGCTTATTGGGGG | TTGTTGGAGAGGATTCCGCC | 0.432553 GGAAGGTGGGCTTATTGGGG | TTGTTGGAGAGGATTCCGCC | 0.432553 GGGAAGGTGGGCTTATTGGG | TTGTTGGAGAGGATTCCGCC | | | | | |
| NCU01001T0 | 1 | 5 | NCU01001T0\_input.txt | NCU01001T0\_output.txt | 0.353610 ACCATTTCCGCGAGATACCC | TGGTCACTTGGAGCTGATCG | 0.354674 TCTCCATCCACCATTTCCGC | TGGTCACTTGGAGCTGATCG | 0.357817 ATCAGCTCCAAGTGACCAGC | CCAACCCTGGACTAAACCGG | 0.357817 ATCAGCTCCAAGTGACCAGC | CCCAACCCTGGACTAAACCG | 0.360714 AGTGGACAGTCGTTTAGCCG | CCAACCCTGGACTAAACCGG | | | | | |
| NCU01002T0 | 3 | 5 | NCU01002T0\_input.txt | NCU01002T0\_output.txt | 0.284626 TACTGGACAAGATTCGGCCG | CGCCTCACCCAAAACATTCG | 0.412080 TACTGGACAAGATTCGGCCG | TTCGTCATTCGCCTTGTTGC | 0.563365 TACTGGACAAGATTCGGCCG | GCCTCACCCAAAACATTCGC | 0.570092 AGATTCGGCCGAGTACAAGG | CGCCTCACCCAAAACATTCG | 0.627653 CCGTACGGAGTCTCGTTTCG | GATCGGTCAAGAAGGGGTGG | | | | | |
| NCU01003T0 | 4 | 5 | NCU01003T0\_input.txt | NCU01003T0\_output.txt | 0.279372 TCTCAGCTTCCACGACTTCG | AAGAACTGAAGAGGGTCGCG | 0.488949 TCTCAGCTTCCACGACTTCG | CTCCAACCTGACGGAAGAGG | 0.649856 AGTTCCAAGACTCCAAGCCC | CTCCAACCTGACGGAAGAGG | 0.695999 CACTCGGGAATTTCCATCGC | AAGAACTGAAGAGGGTCGCG | 0.857142 GTTCCAAGACTCCAAGCCCC | CTCCAACCTGACGGAAGAGG | | | | | |
| NCU01004T0 | 4 | 5 | NCU01004T0\_input.txt | NCU01004T0\_output.txt | 0.572111 GTGGGATGAGGATCTGGTGC | ATATCGCCCTCGTTCTTGGG | 0.575138 CAGGATGGTGTGGGATGAGG | ATATCGCCCTCGTTCTTGGG | 0.695757 GAACTCGCTTCTGCCTTTGG | CGGAGACCACTTCCTCATGG | 0.724205 GTGGGATGAGGATCTGGTGC | ATCCACTGTGGCTCATCAGG | 0.727232 CAGGATGGTGTGGGATGAGG | ATCCACTGTGGCTCATCAGG | | | | | |
| NCU01005T0 | 1 | 5 | NCU01005T0\_input.txt | NCU01005T0\_output.txt | 0.358385 TTGGGTGTTGAGGAAGTCGG | TCCACCACAATCGACCAACC | 0.421340 TGGTTGAGTTTCTGGAGGGC | CTTCCTCAACACCCAAACGC | 0.421784 GAGCGGTTTTTGAGGGATGC | TCCACCACAATCGACCAACC | 0.435689 TGGTTGAGTTTCTGGAGGGC | ACAACCCATAATGCCACCCC | 0.482085 GAGCGGTTTTTGAGGGATGC | CTTCCTCAACACCCAAACGC | | | | | |
| NCU01006T0 | 3 | 5 | NCU01006T0\_input.txt | NCU01006T0\_output.txt | 0.140744 GTGGGGGAGAAAGATGGACG | GTCGGGAAGGGCGTTATAGG | 0.143146 GTGTGGCAGAAGATGTTGCG | GTCGGGAAGGGCGTTATAGG | 0.275107 GGTGTGGCAGAAGATGTTGC | GTCGGGAAGGGCGTTATAGG | 0.360981 GTGGGGGAGAAAGATGGACG | GTACTCCTCCTTTGCCCAGG | 0.363382 GTGTGGCAGAAGATGTTGCG | GTACTCCTCCTTTGCCCAGG | | | | | |
| NCU01007T0 | 3 | 5 | NCU01007T0\_input.txt | NCU01007T0\_output.txt | 0.222819 TTTGGAAGGAAGTGGTGGGG | GGAGGAATGCGGATAGTGGG | 0.363811 TTTGGAAGGAAGTGGTGGGG | GTTTGGGCTTGGATGATGGC | 0.404024 GGCGTTGTTGTTGTTGTTGC | GGAGGAATGCGGATAGTGGG | 0.410912 TTGTTGTTGTTGCTCGACGG | GGAGGAATGCGGATAGTGGG | 0.477792 GGCGTTGTTGTTGTTGTTGC | GAGACCCTGACGGAGAAACG | | | | | |
| NCU01008T0 | 5 | 5 | NCU01008T0\_input.txt | NCU01008T0\_output.txt | 0.277965 GATATGCCACAGTCCCTCCG | AAGTCTGGTCATGACGACGG | 0.346393 CTCCAGACAACGAGGTGAGG | CAGGATCACGGACCTCTTCG | 0.488900 CTCCAGACAACGAGGTGAGG | AAGTCTGGTCATGACGACGG | 0.634378 GCTTCCTCCAGACAACGAGG | AAGTCTGGTCATGACGACGG | 0.985098 GATATGCCACAGTCCCTCCG | CAAAGGCCGAGACTGTGACC | | | | | |
| NCU01009T0 | 4 | 5 | NCU01009T0\_input.txt | NCU01009T0\_output.txt | 0.215067 TACTGGGTCCATTGGCATCG | AGGGTAAGGAATATGGCGCG | 0.285019 TGGACGTGGTGCTTTACTGG | AGGGTAAGGAATATGGCGCG | 0.287083 ATCTGTGGCGGAACTCAAGG | AACAGGTACCGGTATGTGGC | 0.486993 ATCTGTGGCGGAACTCAAGG | GGAGTAGGTCGGAACAGTCG | 0.639717 ATCTGTGGCGGAACTCAAGG | CCGTGTATGCCATGCCTAGG | | | | | |
| NCU01010T0 | 1 | 5 | NCU01010T0\_input.txt | NCU01010T0\_output.txt | 0.500616 TTGGAGGGCTTTTGTGAGGG | CTTCACCTGCACCCTACACC | 0.566102 GTGTAGGGTGCAGGTGAAGG | TCATGGGTCACGTAACGTCC | 0.640352 GTGTAGGGTGCAGGTGAAGG | TTGAGGAGTGTCGCTTGTCG | 0.644559 GAGGGCTTTTGTGAGGGAGG | CTTCACCTGCACCCTACACC | 0.657884 ATTGGGAGGGTTTTGAGGGG | TCATGGGTCACGTAACGTCC | | | | | |
| NCU01011T0 | 1 | 5 | NCU01011T0\_input.txt | NCU01011T0\_output.txt | 0.346603 CACGGACAAGGAATGGTTGC | TAATGTCTCCTCCTCCCCCG | 0.560845 CACGGACAAGGAATGGTTGC | CCCCGCTGATCTTATCACCG | 0.705319 CACGGACAAGGAATGGTTGC | TTGATCTTCATCAGCGCCCC | 0.709633 GGTGATAAGATCAGCGGGGC | TAATGTCTCCTCCTCCCCCG | 0.844920 CACGGACAAGGAATGGTTGC | GCCCCGCTGATCTTATCACC | | | | | |
| NCU01012T0 | 2 | 5 | NCU01012T0\_input.txt | NCU01012T0\_output.txt | 1.727706 GAAGGCATTGTGGTGTCAGG | GCGTCGTGGTTTCAAAAAGC | 1.895920 GAAGGCATTGTGGTGTCAGG | AGCACATAGATTGCACTGCG | 1.951502 GAAGGCATTGTGGTGTCAGG | TGCACTGCGATCTACAGATCC | 2.146383 ATTGTGGTGTCAGGACGGC | GCGTCGTGGTTTCAAAAAGC | 2.283273 GAAGGCATTGTGGTGTCAGG | AAGCACATAGATTGCACTGCG | | | | | |
| NCU01013T0 | 3 | 5 | NCU01013T0\_input.txt | NCU01013T0\_output.txt | 0.547745 GGCCATATCAAAGCCACACG | ATGCGTTGTTTCGTAGCTGC | 1.269342 AGCACGGGCCATATCAAAGC | ATGCGTTGTTTCGTAGCTGC | 1.346159 GGCCATATCAAAGCCACACG | ATTCAGTTCCTGCTGGCAGC | 1.356637 GGCCATATCAAAGCCACACG | GCAATGCGTTGTTTCGTAGC | 1.447451 TACTGTGTGCCCCTTCAACC | TGGTAGCAACAGTCATCCACG | | | | | |
| NCU01014T0 | 1 | 5 | NCU01014T0\_input.txt | NCU01014T0\_output.txt | 0.132774 CCGTCCCCATCTTCTCTTCG | GTACTGCTCATCCGTCTCCG | 0.132774 CCGTCCCCATCTTCTCTTCG | CGTACTGCTCATCCGTCTCC | 0.145081 GAAGTTCACCTGGACTCGGG | CCCAGATCACCAACGGTACC | 0.211056 CCGTCCCCATCTTCTCTTCG | CCCAGATCACCAACGGTACC | 0.213998 ACGGTCCTGTTCTTCACACC | CCCAGATCACCAACGGTACC | | | | | |
| NCU01015T0 | 1 | 5 | NCU01015T0\_input.txt | NCU01015T0\_output.txt | 0.141428 TTTCCATCTGACTCGGTGGC | CTCTATCTTGGTGTCGGCCC | 0.144219 TTTCCATCTGACTCGGTGGC | TCCAACCTCCGACAACAAGG | 0.215434 TTTCCATCTGACTCGGTGGC | ACCAGGCTCCGTTCATATGC | 0.285461 TTTCCATCTGACTCGGTGGC | CGGCTCTATCTTGGTGTCGG | 0.480733 ATATGAACGGAGCCTGGTGC | CAGACGATGCCAACATACGC | | | | | |
| NCU01016T0 | 2 | 5 | NCU01016T0\_input.txt | NCU01016T0\_output.txt | 0.359131 GAACGAGGACGAGAACGAGG | GAGAATCACAGGGCAGGAGG | 0.359131 GAACGAGGACGAGAACGAGG | GGAGAGAATCACAGGGCAGG | 0.770935 GAACGAGGACGAGAACGAGG | GATACAAGAGTCCGTCGCCG | 0.868939 GAACGAGGACGAGAACGAGG | TACCGGGACAGGGAAGATGG | 0.918756 GAACGAGGACGAGAACGAGG | AGATACAAGAGTCCGTCGCC | | | | | |
| NCU01017T0 | 2 | 5 | NCU01017T0\_input.txt | NCU01017T0\_output.txt | 0.567400 GGTAGTGCCTGGGTAAGTCG | GGAGTCTCAGCCCTTTCAGC | 0.649257 GGTAGTGCCTGGGTAAGTCG | AGATAGGATTTGCGGGGTGG | 0.712444 GGTAGTGCCTGGGTAAGTCG | GATAGGATTTGCGGGGTGGG | 0.780976 CCAGTTCTGTCCGTTCGAGG | GGAGTCTCAGCCCTTTCAGC | 0.862833 CCAGTTCTGTCCGTTCGAGG | AGATAGGATTTGCGGGGTGG | | | | | |
| NCU01019T0 | 5 | 5 | NCU01019T0\_input.txt | NCU01019T0\_output.txt | 0.145684 ACAAGCATATGTCCCCCAGC | GCTGTTGCACTGGTAAAGCC | 0.148234 CCTCAATATGGCGCAATGGC | GGCATGGGAGGAGTGTATGG | 0.148234 CCTCAATATGGCGCAATGGC | GATACACTGGGCATGGGAGG | 0.215861 ACAAGCATATGTCCCCCAGC | GGCATGGGAGGAGTGTATGG | 0.215861 ACAAGCATATGTCCCCCAGC | GATACACTGGGCATGGGAGG | | | | | |
| NCU01021T0 | 2 | 5 | NCU01021T0\_input.txt | NCU01021T0\_output.txt | 0.574707 AGTGTGAGGGTAGGTCTCGG | ATGGGACGTCTTTGGTTGGG | 0.716003 AGTGTGAGGGTAGGTCTCGG | TGGGTCTCAAGCAATACGGC | 1.063079 CAGTGTGAGGGTAGGTCTCG | ATGGGACGTCTTTGGTTGGG | 1.149484 CACTCCAGTGTGAGGGTAGG | ATGGGACGTCTTTGGTTGGG | 1.204376 CAGTGTGAGGGTAGGTCTCG | TGGGTCTCAAGCAATACGGC | | | | | |
| NCU01022T0 | 3 | 5 | NCU01022T0\_input.txt | NCU01022T0\_output.txt | 0.357141 TTACTGCAACCGGTCAGTGG | ATCATCAAGACCTTGGGGCG | 0.418912 TTACTGCAACCGGTCAGTGG | ATTTTGACGCATGACTGGCG | 0.493369 TAAGCACACCGAGTTCGACG | ATCATCAAGACCTTGGGGCG | 0.495666 TTCGACGAAGCTGAGAAGGC | ATCATCAAGACCTTGGGGCG | 0.849652 GCTGAGAAGGCAGTAGCACC | ATCATCAAGACCTTGGGGCG | | | | | |
| NCU01023T0 | 2 | 5 | NCU01023T0\_input.txt | NCU01023T0\_output.txt | 0.629573 GACCCGAAATTTTCTCGGCC | ACTTGGGAGACTGGCAAACC | 0.629573 GGACCCGAAATTTTCTCGGC | ACTTGGGAGACTGGCAAACC | 0.630773 CGAAATTTTCTCGGCCCTGG | ACTTGGGAGACTGGCAAACC | 0.648134 TGCTTTTGGGGTCCTTGTCC | ACTTGGGAGACTGGCAAACC | 0.996644 GAAATTTTCTCGGCCCTGGG | ACTTGGGAGACTGGCAAACC | | | | | |
| NCU01024T0 | 2 | 5 | NCU01024T0\_input.txt | NCU01024T0\_output.txt | 0.277457 CCCTTCGTTCGGTAGAGTCG | GTAGGAGGTCGGATTGGTGC | 0.287683 GCAGCAATGGGTTTCGTAGC | TTCATCGTTGACGGGGTAGC | 0.358244 GCAGCAATGGGTTTCGTAGC | GTAGGAGGTCGGATTGGTGC | 0.425798 GCAGCAATGGGTTTCGTAGC | AGGTCTTCATCGTTGACGGG | 0.552105 CGTTCGGTAGAGTCGAGACC | GTAGGAGGTCGGATTGGTGC | | | | | |
| NCU01025T0 | 1 | 5 | NCU01025T0\_input.txt | NCU01025T0\_output.txt | 0.075291 TACCCACTGTTCATCACCGC | TGAAGAACGGAGACTTCGCC | 0.075830 AGACTGATGTTGGCAGTGGG | TGAAGAACGGAGACTTCGCC | 0.141335 TTCCAGGTTGAACTCCACGG | TTCTCCCTTCGATATGGCGC | 0.212522 GGAATCCCATCACCCCTTCG | TTCTCCCTTCGATATGGCGC | 0.215782 TACCCACTGTTCATCACCGC | CGAAGGGGTGATGGGATTCC | | | | | |
| NCU01026T0 | 2 | 5 | NCU01026T0\_input.txt | NCU01026T0\_output.txt | 0.130893 TTGAGTGGAATCCGTGGTGG | GTTGCACGATAAGACACGGC | 0.142872 TTGAGTGGAATCCGTGGTGG | TCGATTGCTGGGCTATGACC | 0.265737 GTGATGTGGTCAATTGCGGG | GTTGCACGATAAGACACGGC | 0.273922 CGTGATGTGGTCAATTGCGG | GTTGCACGATAAGACACGGC | 0.273922 CCGTGATGTGGTCAATTGCG | GTTGCACGATAAGACACGGC | | | | | |
| NCU01027T0 | 1 | 5 | NCU01027T0\_input.txt | NCU01027T0\_output.txt | 0.212777 GAGTTCCAGGACGATCACCG | GCGTAGAGGTATTCCCCTGC | 0.212777 CGAGTTCCAGGACGATCACC | GCGTAGAGGTATTCCCCTGC | 0.283754 GAGTTCCAGGACGATCACCG | TAGAGGTATTCCCCTGCCCC | 0.283754 CGAGTTCCAGGACGATCACC | TAGAGGTATTCCCCTGCCCC | 0.283779 GCCTCCCATAAACCCTACGG | TTCTTTCATCCTCGAGGCCG | | | | | |
| NCU01032T0 | 1 | 5 | NCU01032T0\_input.txt | NCU01032T0\_output.txt | 0.212717 GCAGGACGAACATGAAAGCG | AAATGACCGTCCCAATCCCC | 0.477372 CAGGACGAACATGAAAGCGG | AAATGACCGTCCCAATCCCC | 0.707147 AGGACGAACATGAAAGCGGG | AAATGACCGTCCCAATCCCC | 0.907892 TGCAGGACGAACATGAAAGC | AAATGACCGTCCCAATCCCC | 0.987969 GCAGGACGAACATGAAAGCG | CGCATACCCTTGACCAAACC | | | | | |
| NCU01033T0 | 1 | 5 | NCU01033T0\_input.txt | NCU01033T0\_output.txt | 0.073929 CGAAAACCAAAGCACGGAGG | CCCATTCCCATACCTCGTCG | 0.215909 CAACTTCACTCCCCTGGACC | CTAACATCTCCCCCAGCAGC | 0.219459 CGAAAACCAAAGCACGGAGG | CTAACATCTCCCCCAGCAGC | 0.293353 ACCTCCTTTCCCTCCTCTGG | CCTCCGTGCTTTGGTTTTCG | 0.293752 AATCCAAACCTCGCTCCTCC | CCTCCGTGCTTTGGTTTTCG | | | | | |
| NCU01034T1 | 2 | 5 | NCU01034T1\_input.txt | NCU01034T1\_output.txt | 0.139060 GAAAAACCAGGCAGTGCAGG | CATGACCTCCATCCCCAACC | 0.143752 GGCGAAGAGGATAATCCCCG | CATGACCTCCATCCCCAACC | 0.623552 GGCGAAGAGGATAATCCCCG | ATTTGCACGATATTCCCGCG | 0.648752 AGAGGATAATCCCCGACCCG | CATGACCTCCATCCCCAACC | 0.871409 TCACAACTGAGACCCCTTGG | CATGACCTCCATCCCCAACC | | | | | |
| NCU01034T0 | 2 | 5 | NCU01034T0\_input.txt | NCU01034T0\_output.txt | 0.139060 GAAAAACCAGGCAGTGCAGG | CATGACCTCCATCCCCAACC | 0.143752 GGCGAAGAGGATAATCCCCG | CATGACCTCCATCCCCAACC | 0.623552 GGCGAAGAGGATAATCCCCG | ATTTGCACGATATTCCCGCG | 0.648752 AGAGGATAATCCCCGACCCG | CATGACCTCCATCCCCAACC | 0.871409 TCACAACTGAGACCCCTTGG | CATGACCTCCATCCCCAACC | | | | | |
| NCU01036T0 | 2 | 5 | NCU01036T0\_input.txt | NCU01036T0\_output.txt | 0.429368 CCAAGGTCCTCAGTATGGCG | TAATGCTCATAGCCGGCACC | 0.431645 CCCAAGGTCCTCAGTATGGC | TAATGCTCATAGCCGGCACC | 0.501636 ACACTTCACCAATGCTCCCC | TAATGCTCATAGCCGGCACC | 0.583267 AATGCTCCCCCAAAGTCTCC | TAATGCTCATAGCCGGCACC | 0.631290 CTCACGCCACAGTATAGGGC | ATCGCCGTCATGAAAATGGC | | | | | |
| NCU01038T0 | 4 | 5 | NCU01038T0\_input.txt | NCU01038T0\_output.txt | 0.569191 AACAGCTGACTTTGGGTCCG | GTGAAGGGGGTGGAGAATGG | 0.763205 CCGATGTGAACACAGATGGC | GTGAAGGGGGTGGAGAATGG | 0.785336 AACAGCTGACTTTGGGTCCG | TGGAGAATGGAGCGTTGAGG | 0.852128 GTTGTCGGATACCCCATGGC | GTGAAGGGGGTGGAGAATGG | 0.871361 AGTTGTCGGATACCCCATGG | GTGAAGGGGGTGGAGAATGG | | | | | |
| NCU01039T0 | 7 | 5 | NCU01039T0\_input.txt | NCU01039T0\_output.txt | 1.168718 CCACGCAAAGGACATTCAGC | CGTCGTCAATTGATGTCACCG | 1.241962 CCACGCAAAGGACATTCAGC | ACCTCTATCCTCCATTCCCCC | 1.299000 CCACGCAAAGGACATTCAGC | GTCACCGATACTCGAAAGCG | 1.323769 GACTCCGGAAAGATCGCTGG | GTCGCAGCATGGATATCTGC | 1.458954 CCACGCAAAGGACATTCAGC | GCACCTCTATCCTCCATTCCC | | | | | |
| NCU01041T0 | 3 | 5 | NCU01041T0\_input.txt | NCU01041T0\_output.txt | 0.642888 TCCAGGAAGCAAAGCAATGC | AAAGGAAGGGAAGGAAGCCG | 0.770993 GCTTCCCCTTTCATGCTTCG | AGTCGTGGTTTGAGACGTCG | 0.847742 GCTTCCCCTTTCATGCTTCG | ACGGGGGAGAATGTTGATGC | 0.861091 AACACCTTTGTCCGGTTCCC | ACGGGGGAGAATGTTGATGC | 0.922181 TCCAGGAAGCAAAGCAATGC | AGTCGTGGTTTGAGACGTCG | | | | | |
| NCU01042T0 | 2 | 5 | NCU01042T0\_input.txt | NCU01042T0\_output.txt | 0.214207 CATCCTCAGTCCAGGCAAGG | GGGAGGCGGTGAGAAATAGG | 0.439866 ATTCTTCCGAGGGCGAATCC | AGATTGAAAGGGGTGGGAGC | 0.440284 CATCCTCAGTCCAGGCAAGG | AGATTGAAAGGGGTGGGAGC | 0.483026 GACGATGTCGAATTGTGCGG | GCGACGAAGAGTAAGGTGGG | 0.483026 GACGATGTCGAATTGTGCGG | GGCGACGAAGAGTAAGGTGG | | | | | |
| NCU01043T0 | 3 | 5 | NCU01043T0\_input.txt | NCU01043T0\_output.txt | 0.926606 CACGTTCAATGGTCCAAGGC | AACCGGAGAGACCAAAGACC | 0.937402 TTCAATGGTCCAAGGCTGGG | AACCGGAGAGACCAAAGACC | 1.312802 CACGTTCAATGGTCCAAGGC | AAACCGGAGAGACCAAAGACC | 1.321767 TGGGAGTCGGCATTTTCCC | TGCTCCGTTAGGTGTCAAGG | 1.323599 TTCAATGGTCCAAGGCTGGG | AAACCGGAGAGACCAAAGACC | | | | | |
| NCU01044T0 | 1 | 5 | NCU01044T0\_input.txt | NCU01044T0\_output.txt | 0.291498 CACCATTGCGAGTGTGTTGG | AAGTCTTGCCCACATGGTCC | 0.296047 CACCATTGCGAGTGTGTTGG | TCCCAGCCCATAAGTCTTGC | 0.498347 TTGACGGGGTATAGGAGGGG | ACCCAAGACGCATCATCACC | 0.635276 TTTGGTGTTTGGGCTTTGGC | GTACTTCGGCCTCGTGATGG | 0.716357 TGTGGGCAAGACTTATGGGC | ACCCAAGACGCATCATCACC | | | | | |
| NCU01045T0 | 2 | 5 | NCU01045T0\_input.txt | NCU01045T0\_output.txt | 0.142952 TCTTCTGCCATCTCTGCTGC | TGTGGGTGATGAGCATGTCC | 0.213685 AGCTCTTCTGCCATCTCTGC | TGTGGGTGATGAGCATGTCC | 0.360154 ATATCTGCGGCTCAGTCTGC | AGTTGTTGTATCGGTGGGGG | 0.417896 ATATCTGCGGCTCAGTCTGC | GATCGGGAACTCGTACCAGG | 0.417896 ATATCTGCGGCTCAGTCTGC | CAGGATCGGGAACTCGTACC | | | | | |
| NCU01045T1 | 2 | 5 | NCU01045T1\_input.txt | NCU01045T1\_output.txt | 0.142952 TCTTCTGCCATCTCTGCTGC | TGTGGGTGATGAGCATGTCC | 0.212292 ATATCTGCGGCTCAGTCTGC | GTTGTATCGGTGGGGGTAGC | 0.213685 AGCTCTTCTGCCATCTCTGC | TGTGGGTGATGAGCATGTCC | 0.345879 GAGGCTGTCGTTGATGTTGC | GTTGTATCGGTGGGGGTAGC | 0.360154 ATATCTGCGGCTCAGTCTGC | AGTTGTTGTATCGGTGGGGG | | | | | |
| NCU01047T0 | 1 | 5 | NCU01047T0\_input.txt | NCU01047T0\_output.txt | 0.067459 GGAGAGCTGGTATGTCGAGC | AGAAGCCATCAGTGTTCCCG | 0.268948 GGAGAGCTGGTATGTCGAGC | AAACGCAAGGCTTAACTCGC | 0.275149 CAAGCGAGTTAAGCCTTGCG | CGATCACAGCCAAGCTTTCG | 0.286477 TGCTGAGGTGTTTGGAGAGC | AGAAGCCATCAGTGTTCCCG | 0.359188 AGTGCAGAAGGGCATGAAGG | AGAAGCCATCAGTGTTCCCG | | | | | |
| NCU01048T0 | 1 | 5 | NCU01048T0\_input.txt | NCU01048T0\_output.txt | 0.137557 GAGTCAGCGGAAGAGATCGG | CATCATCACCCTCCCCTTCG | 0.137557 GGAGTCAGCGGAAGAGATCG | CATCATCACCCTCCCCTTCG | 0.141514 GGCAGGTATGGATGAGACCG | CATCATCACCCTCCCCTTCG | 0.209163 GAGTCAGCGGAAGAGATCGG | CTGTAGCACAGCCATCTCCC | 0.209163 GGAGTCAGCGGAAGAGATCG | CTGTAGCACAGCCATCTCCC | | | | | |
| NCU01049T0 | 1 | 5 | NCU01049T0\_input.txt | NCU01049T0\_output.txt | 0.213804 GGAGGCGGTAGGTAATGTGG | ATGCTTAGTTGCCCGACTCC | 0.213872 GGGGGTGGAATATGGTGTGG | ATGCTTAGTTGCCCGACTCC | 0.216617 TGTGGGAAGGGTACGTTTGG | ATGCTTAGTTGCCCGACTCC | 0.285284 GGAGGCGGTAGGTAATGTGG | CTTAGTTGCCCGACTCCTCG | 0.285352 GGGGGTGGAATATGGTGTGG | CTTAGTTGCCCGACTCCTCG | | | | | |
| NCU01050T0 | 2 | 5 | NCU01050T0\_input.txt | NCU01050T0\_output.txt | 0.736713 CTTTCCTGGGGCTTACTCGG | ACCATGTTCCCACCATACCC | 0.737531 GTGGTACGGGTGCTAAGACC | ACCATGTTCCCACCATACCC | 0.947499 TCGACTGTCAGCTTTCCTGG | ACCATGTTCCCACCATACCC | 0.950005 AATGTCGTTGGTGGTACGGG | ACCATGTTCCCACCATACCC | 0.951067 GACTGTCAGCTTTCCTGGGG | ACCATGTTCCCACCATACCC | | | | | |
| NCU01051T0 | 3 | 5 | NCU01051T0\_input.txt | NCU01051T0\_output.txt | 0.579567 GGAAAGGAGTCTCACGACCG | GAGTATTGGAGGGAGGCACC | 0.583531 AGTGAAAGGGCTGGAAAGGG | GAGTATTGGAGGGAGGCACC | 0.585493 AAGGGCTGGAAAGGGAAAGG | GAGTATTGGAGGGAGGCACC | 0.717251 GGGAAAGGAGTCTCACGACC | GAGTATTGGAGGGAGGCACC | 0.719830 CACGACCGAGGATGGATTCC | GAGTATTGGAGGGAGGCACC | | | | | |
| NCU01052T0 | 2 | 5 | NCU01052T0\_input.txt | NCU01052T0\_output.txt | 0.145548 AAGACTGAGGATGGTGCTGC | GCCGCCAAAGTAAACGAAGG | 0.218688 GTACCTAGCTGAGCCTTGCC | TGGCCATGACCCTTTCTTCC | 0.218688 GGTACCTAGCTGAGCCTTGC | TGGCCATGACCCTTTCTTCC | 0.288989 GTACCTAGCTGAGCCTTGCC | GCCGCCAAAGTAAACGAAGG | 0.288989 GGTACCTAGCTGAGCCTTGC | GCCGCCAAAGTAAACGAAGG | | | | | |
| NCU01053T0 | 2 | 5 | NCU01053T0\_input.txt | NCU01053T0\_output.txt | 0.359036 ATGGCGTGTAAGTGGTGAGG | TCACTCAGCACCTCACATGG | 0.654209 ATGGCGTGTAAGTGGTGAGG | TGGAGTAAGAGGACCTGGGC | 0.712225 ATGGCGTGTAAGTGGTGAGG | CACTCAGCACCTCACATGGG | 0.774770 GTCAAAACGTTCGGCATCGG | TCACTCAGCACCTCACATGG | 0.858654 GAGGATGAAGGGGAATGGCG | TCACTCAGCACCTCACATGG | | | | | |
| NCU01054T0 | 1 | 5 | NCU01054T0\_input.txt | NCU01054T0\_output.txt | 0.358230 GTGGTCTTACGTCTCTCGGC | ATGGACGAGCTCATCCTTGC | 0.358230 GTGGTCTTACGTCTCTCGGC | AGCAATGGACGAGCTCATCC | 0.429037 GTGGTCTTACGTCTCTCGGC | TCAACCTGACAACTGAGCCG | 0.493504 GTTTTTGGGTCAGGGATGCG | TCAACCTGACAACTGAGCCG | 0.637885 GTGGTCTTACGTCTCTCGGC | GAGCCGACTGGTAGTGAAGC | | | | | |
| NCU01055T0 | 3 | 5 | NCU01055T0\_input.txt | NCU01055T0\_output.txt | 0.483725 CAAGACGGATTCCTTGCTGC | TTAGCCGATCCGTAATGGCC | 0.712385 AAGACGGATTCCTTGCTGCC | TTAGCCGATCCGTAATGGCC | 0.909699 CAAGACGGATTCCTTGCTGC | ACCAGGATTCTCGTTAGCCG | 1.112480 AGACGGATTCCTTGCTGCC | TTAGCCGATCCGTAATGGCC | 1.121205 TGAGCAGGTGTACCAAGAGC | CGACAGTACCGGCGTTAAGG | | | | | |
| NCU01056T0 | 5 | 5 | NCU01056T0\_input.txt | NCU01056T0\_output.txt | 0.142308 TTCGTCCCGTTCTCATCAGC | TGGTCTCGCTTTGACAGTCC | 0.142823 CATGGCTTTTCCGGGTTTCG | TGGTCTCGCTTTGACAGTCC | 0.353145 AGCTCGAACCGAACTGTTCC | TGGTCTCGCTTTGACAGTCC | 0.362765 TCCTCTTTCACTGGCCATGG | TGGTCTCGCTTTGACAGTCC | 0.560028 GTCCCGTTCTCATCAGCTCG | TGGTCTCGCTTTGACAGTCC | | | | | |
| NCU01057T0 | 2 | 5 | NCU01057T0\_input.txt | NCU01057T0\_output.txt | 1.042676 GTGATCATTAGCCGGCGAGG | GGGCATTGAGTTACGAACGC | 1.118422 GGACCGCCATCATGTTTACC | GGGCATTGAGTTACGAACGC | 1.229708 CTTGCTGGTGTCGATTGCG | GGGCATTGAGTTACGAACGC | 1.232983 CGATTGCGTGATCATTAGCCG | GGGCATTGAGTTACGAACGC | 1.250604 GCTTGCTGGTGTCGATTGC | GGGCATTGAGTTACGAACGC | | | | | |
| NCU01057T1 | 1 | 5 | NCU01057T1\_input.txt | NCU01057T1\_output.txt | 0.280643 CGTAGGGCTTAAGGACGTCG | GGCTCCCGGAATTCTAGTCG | 0.427376 CTTCCTCATCGGTGAAGGGG | CGACGTCCTTAAGCCCTACG | 0.496479 CTTCCTCATCGGTGAAGGGG | CATCTTCCCCGACGGTATCC | 0.498391 GATCTCCTTGGGGAACTCCG | GGCTCCCGGAATTCTAGTCG | 0.498391 CGATCTCCTTGGGGAACTCC | GGCTCCCGGAATTCTAGTCG | | | | | |
| NCU01058T0 | 1 | 5 | NCU01058T0\_input.txt | NCU01058T0\_output.txt | 0.422656 AGGTCTGACGGAGAGAGAGG | TTGGTCAAATCACGCATCGC | 0.560636 ATTGAGAAGCGACAGCCTCG | ATGTCTAACGCTCTGCTCCG | 0.627145 AGCAGAAGATCCTTGTCGCG | TTGGTCAAATCACGCATCGC | 0.627145 ATTGAGAAGCGACAGCCTCG | TTGGTCAAATCACGCATCGC | 0.636548 AGGTCTGACGGAGAGAGAGG | AAATCACGCATCGCCATTCC | | | | | |
| NCU01059T0 | 1 | 5 | NCU01059T0\_input.txt | NCU01059T0\_output.txt | 0.076124 GGTACTGGGGAGGTTTCTGC | TGCTTTTTACTTCGCACCGC | 0.134425 CTTGTCTCTCGAGGCGTACC | GCAGAAACCTCCCCAGTACC | 0.134711 CTTGTCTCTCGAGGCGTACC | ACCAAGATGAGACAGCTGCC | 0.143481 GGTCATTGTCAGACTCCGGG | GCAGAAACCTCCCCAGTACC | 0.148919 GTCTCATCTTGGTGACGGGG | TGCTTTTTACTTCGCACCGC | | | | | |
| NCU01060T0 | 1 | 5 | NCU01060T0\_input.txt | NCU01060T0\_output.txt | 0.079402 AGTGAACACAGAGAGCGAGC | CATGCGGGCTTGATGATTCG | 0.147709 ATGGCTAGGTGGTGTGATGC | CATGCGGGCTTGATGATTCG | 0.555045 GACCCGACGAGTTTAGAGCG | TCTCTGTGTTCACTGCGTCC | 0.565404 CAAATCACCATCCGCATCGC | CTCCCGCTCTAAACTCGTCG | 0.568046 AGTGAACACAGAGAGCGAGC | CTCTACCACTGAGCCATGCG | | | | | |
| NCU01061T0 | 1 | 5 | NCU01061T0\_input.txt | NCU01061T0\_output.txt | 0.288551 GAGCATGAGGAGGGTTCTGG | ATGCGTCTTCAACTCCTCCG | 0.352250 GTGGGCGATTTTCAGGTTGG | ATGCGTCTTCAACTCCTCCG | 0.357047 GGTTTCAAAGAACGTGCCGG | GAAACAACCGTGATGCACCC | 0.357047 GGGTTTCAAAGAACGTGCCG | GAAACAACCGTGATGCACCC | 0.428020 ATTTTCAGGTTGGCTGCTGC | ATGCGTCTTCAACTCCTCCG | | | | | |
| NCU01063T0 | 2 | 5 | NCU01063T0\_input.txt | NCU01063T0\_output.txt | 0.621448 CGACTTCTTCTACGGGACCG | CCTCGGTGCAGTTCAAATGG | 0.629026 CGACTTCTTCTACGGGACCG | AGCCGCATATCCAGTGAACG | 0.696441 CGACTTCTTCTACGGGACCG | AAATGGAGTTTGACAGCCGC | 0.774151 AGTTTGATTTCAACCGGCCG | TCGAGTAAATCTGGCCCTGC | 0.908563 GGACCGTACAGTACACGTGC | TCGAGTAAATCTGGCCCTGC | | | | | |
| NCU01064T0 | 2 | 5 | NCU01064T0\_input.txt | NCU01064T0\_output.txt | 1.123077 GTGGAGTGCAGATGTGATGC | CAGACCCTTGTGGATGGACG | 1.133588 TGGAGTGCAGATGTGATGCG | CAGACCCTTGTGGATGGACG | 1.501160 GGTGGGTTCTGCCATTTTGG | ACTCGATACCGATCTGGGGC | 1.826283 GGAGTGCAGATGTGATGCGG | CAGACCCTTGTGGATGGACG | 1.911231 GTGGAGTGCAGATGTGATGC | ACTCGATACCGATCTGGGGC | | | | | |
| NCU01065T0 | 4 | 5 | NCU01065T0\_input.txt | NCU01065T0\_output.txt | 0.548962 AACATGAAGACCAGAGCGGG | CTTCTCACCAGCCGAAAACG | 0.710591 AACATGAAGACCAGAGCGGG | TGCAATCTTCTCACCAGCCG | 0.757728 AACATGAAGACCAGAGCGGG | GCTTGGAAAGACGTCATCCG | 0.918937 AACATGAAGACCAGAGCGGG | TTCTCACCAGCCGAAAACGG | 0.985407 AACATGAAGACCAGAGCGGG | AAAACGGGAGGACGAAAAGC | | | | | |
| NCU01066T0 | 1 | 5 | NCU01066T0\_input.txt | NCU01066T0\_output.txt | 0.068554 GCTCCATACCGAGAGAAGGC | TCAAGGTTGAGGGTAACGCC | 0.068825 GCTCCATACCGAGAGAAGGC | ATGGACTGCAAGGGTTCTCG | 0.071655 GTTCCTCCTGGTGAAGCTCC | TCAAGGTTGAGGGTAACGCC | 0.071925 GTTCCTCCTGGTGAAGCTCC | ATGGACTGCAAGGGTTCTCG | 0.073251 CAGTCAGTCAGTCAGCCAGG | TCAAGGTTGAGGGTAACGCC | | | | | |
| NCU01067T0 | 3 | 5 | NCU01067T0\_input.txt | NCU01067T0\_output.txt | 0.342436 GCGAACATGATCTGCGATGG | TTGCTCCCTAACTACTGCGC | 0.356303 GTGCTGCGAACATGATCTGC | TTGCTCCCTAACTACTGCGC | 0.413339 GCGAACATGATCTGCGATGG | GCGAAAATCGGTCGACAACC | 0.427207 GTGCTGCGAACATGATCTGC | GCGAAAATCGGTCGACAACC | 0.487433 GCGAACATGATCTGCGATGG | AGAATCTTCCCGGTTGCTCC | | | | | |
| NCU01068T0 | 3 | 5 | NCU01068T0\_input.txt | NCU01068T0\_output.txt | 0.148853 CTTTTGCAGTCGGTCCATGC | TCGAACGAGTAAACGTGGGG | 0.927231 CTTTTGCAGTCGGTCCATGC | TTTTGTGGACAGGAGCCACG | 1.120015 CTTTTGCAGTCGGTCCATGC | CGAACGAGTAAACGTGGGGG | 1.166251 GTCTTCGTACGAGTCGCAGC | TCGAACGAGTAAACGTGGGG | 1.173331 TCTTCGTACGAGTCGCAGC | TCGAACGAGTAAACGTGGGG | | | | | |
| NCU01069T0 | 5 | 5 | NCU01069T0\_input.txt | NCU01069T0\_output.txt | 0.285609 ACTCCGTCTTTAGCACCAGC | GATACGGAGGACACGGTTCG | 0.286974 ACTCCGTCTTTAGCACCAGC | AGGTGTTGAAACGAGGTCCG | 0.486223 ACTCCGTCTTTAGCACCAGC | TTGTCGATACGGAGGACACG | 0.577218 ACTCCGTCTTTAGCACCAGC | TATCCCAGTGGAAAGGACGC | 0.843471 CCAAGTCTTCCACTGCTTGC | GATACGGAGGACACGGTTCG | | | | | |
| NCU01070T0 | 2 | 5 | NCU01070T0\_input.txt | NCU01070T0\_output.txt | 0.287712 CGGCGTAGGTTCAGTGTAGG | GTCCGAGGTCTTCAGGAAGC | 0.343782 AGAAGGGACAGTGCTGAAGC | GTGTTTGAGGGTTGCTGTCG | 0.415946 TGCTGAAGCTGTGATACCGG | GTGTTTGAGGGTTGCTGTCG | 0.558400 AGGTTTCTCACAGCAGCTGG | GTGTTTGAGGGTTGCTGTCG | 0.646529 CGGCGTAGGTTCAGTGTAGG | AGGATTCGAGCACCAGATGG | | | | | |
| NCU01071T0 | 2 | 5 | NCU01071T0\_input.txt | NCU01071T0\_output.txt | 0.424365 AAGCCGAAGTCTTGGTCAGG | TTTCTTTACGAGACGGCCGG | 0.572646 AAGCCGAAGTCTTGGTCAGG | TTGGATGAGACGAAGGAGGC | 0.778448 AAGCCGAAGTCTTGGTCAGG | TTCGTTTAGGCCGCATGAGG | 0.977874 CGGCGACAAAAGTCTTCACC | TTCGTTTAGGCCGCATGAGG | 0.984080 AAGCCGAAGTCTTGGTCAGG | TGTTGCCGTTGGATGAGACG | | | | | |
| NCU01072T0 | 2 | 5 | NCU01072T0\_input.txt | NCU01072T0\_output.txt | 0.287968 GTCCAAGGTAACCACTGGGG | TCGAAAGCTGGATGTCTGGG | 0.428205 GTCCAAGGTAACCACTGGGG | GGACTCTGCAGGTTCCTTCG | 0.567254 GAAGCTGGTTTGTGACACGC | TCGAAAGCTGGATGTCTGGG | 0.660398 GTCCAAGGTAACCACTGGGG | AAAGCTGGATGTCTGGGAGG | 0.707492 GAAGCTGGTTTGTGACACGC | GGACTCTGCAGGTTCCTTCG | | | | | |
| NCU01073T0 | 1 | 5 | NCU01073T0\_input.txt | NCU01073T0\_output.txt | 0.069614 AAAGGCTGAGGCATCTTCCC | GTGGGAAACTGAGAGAGGCC | 0.071545 CCCAGACAGGCTTCTCAAGG | GTGGGAAACTGAGAGAGGCC | 0.071925 TTCTCAAGGCTGGCACTAGC | GTGGGAAACTGAGAGAGGCC | 0.136849 CCCAGACAGGCTTCTCAAGG | GGATGCTGCATTTCGGTTCC | 0.137229 TTCTCAAGGCTGGCACTAGC | GGATGCTGCATTTCGGTTCC | | | | | |
| NCU01074T0 | 4 | 5 | NCU01074T0\_input.txt | NCU01074T0\_output.txt | 0.072768 CAGGATGTCGGAGGGTATGC | ACTGCTCTGTTCTCTGTCGC | 0.141917 ACCGCTGTTCGATGAGATCC | ACTGCTCTGTTCTCTGTCGC | 0.286445 GGGTATGCTGATAGGCGAGG | ACTGCTCTGTTCTCTGTCGC | 0.286445 GGAGGGTATGCTGATAGGCG | ACTGCTCTGTTCTCTGTCGC | 0.286445 CGGAGGGTATGCTGATAGGC | ACTGCTCTGTTCTCTGTCGC | | | | | |
| NCU01074T1 | 4 | 5 | NCU01074T1\_input.txt | NCU01074T1\_output.txt | 0.072768 CAGGATGTCGGAGGGTATGC | ACTGCTCTGTTCTCTGTCGC | 0.141917 ACCGCTGTTCGATGAGATCC | ACTGCTCTGTTCTCTGTCGC | 0.286445 GGGTATGCTGATAGGCGAGG | ACTGCTCTGTTCTCTGTCGC | 0.286445 GGAGGGTATGCTGATAGGCG | ACTGCTCTGTTCTCTGTCGC | 0.286445 CGGAGGGTATGCTGATAGGC | ACTGCTCTGTTCTCTGTCGC | | | | | |
| NCU01075T0 | 3 | 5 | NCU01075T0\_input.txt | NCU01075T0\_output.txt | 0.429151 GGTAGAAGGCTGCCTGTAGC | GCCTGGAAGGATAGCGAAGG | 0.429151 GGTAGAAGGCTGCCTGTAGC | CCGAAGCCTGGAAGGATAGC | 0.493717 CGTGGGTGAAAATCAAGGCC | GCCTGGAAGGATAGCGAAGG | 0.493717 CGTGGGTGAAAATCAAGGCC | CCGAAGCCTGGAAGGATAGC | 0.559448 GGTAGAAGGCTGCCTGTAGC | TTCACCAAGCGTTCTGTTGC | | | | | |
| NCU01076T0 | 2 | 5 | NCU01076T0\_input.txt | NCU01076T0\_output.txt | 0.288925 GGTGCTAGAGTGGGAAAGGG | TATGTGTGGTTGACGTGGGG | 0.288925 GGGTGCTAGAGTGGGAAAGG | TATGTGTGGTTGACGTGGGG | 0.428731 GTGCTAGAGTGGGAAAGGGC | TATGTGTGGTTGACGTGGGG | 0.580767 TGGAGAGGGTGAGGACAAGG | GGTAGATCTGGCCATTCCCG | 0.642000 TGCCGGAGAAGCTCTTTTGG | TATGTGTGGTTGACGTGGGG | | | | | |
| NCU01077T0 | 2 | 5 | NCU01077T0\_input.txt | NCU01077T0\_output.txt | 0.284442 AGAACTCGTGATGGGTACGC | TTTTTGTTCGGGGGAGGAGG | 0.351767 AGAACTCGTGATGGGTACGC | ATGAGTTTGTGGTGGTGGGG | 1.175168 AGAACTCGTGATGGGTACGC | GACGTATGAGCAGCAGTTGG | 1.194193 AGAACTCGTGATGGGTACGC | ACGTATGAGCAGCAGTTGGC | 1.254944 CAGCAGAGAGTGAACAACGG | TTTTTGTTCGGGGGAGGAGG | | | | | |
| NCU01078T0 | 3 | 5 | NCU01078T0\_input.txt | NCU01078T0\_output.txt | 1.549765 ACGTCGAGATGACCATTGCC | ATTGCAGCGTACCCATCACG | 1.562438 ACGTCGAGATGACCATTGCC | TTTGGGGATACATTGCAGCG | 1.620131 AATATGGGAGTGTCGCAGCG | ATTGCAGCGTACCCATCACG | 1.632805 AATATGGGAGTGTCGCAGCG | TTTGGGGATACATTGCAGCG | 1.687031 ACGTCGAGATGACCATTGCC | GGATACATTGCAGCGTACCC | | | | | |
| NCU01079T0 | 2 | 5 | NCU01079T0\_input.txt | NCU01079T0\_output.txt | 0.219946 TTGGAATGGGTCTGGGTTGG | GAGGCAGAGGACTATGGTGC | 0.993711 CGTTTTGGAATGGGTCTGGG | GAGGCAGAGGACTATGGTGC | 1.249045 TTGGAATGGGTCTGGGTTGG | GGACTTACCGCAGTTACTCCC | 1.447293 TCGTTGCCGTTTTGGAATGG | GGACTTACCGCAGTTACTCCC | 1.512080 CGTAATCGCTTTTCCTGGCC | GGACTTACCGCAGTTACTCCC | | | | | |
| NCU01080T0 | 1 | 5 | NCU01080T0\_input.txt | NCU01080T0\_output.txt | 0.483960 TCAGACTGGATGAAGACGCG | CGGCAACCAAAGGTTACACC | 0.636686 AGGAAAGTAGGTGGTTCGGC | CGGCAACCAAAGGTTACACC | 0.649121 AAGGGCTGGAGGATTTTCCC | CGGCAACCAAAGGTTACACC | 0.709007 GGCTGGAGGATTTTCCCAGG | CGGCAACCAAAGGTTACACC | 0.734333 AAGGGCTGGAGGATTTTCCC | ATCCATGCCTACATCACGGG | | | | | |
| NCU01081T0 | 3 | 5 | NCU01081T0\_input.txt | NCU01081T0\_output.txt | 0.492329 GATCTCCAGCAGAGTCACCG | TTTTAGACGCAGGTACCGGC | 0.559155 GATCTCCAGCAGAGTCACCG | TCGAAGATACCCGAAACCCG | 0.690372 GATCTCCAGCAGAGTCACCG | CGCTGAGAAACTGTGTTGGG | 0.690372 GATCTCCAGCAGAGTCACCG | CCGCTGAGAAACTGTGTTGG | 0.700539 GATCTCCAGCAGAGTCACCG | GTACCGGCAGCCGTTATACC | | | | | |
| NCU01082T0 | 2 | 5 | NCU01082T0\_input.txt | NCU01082T0\_output.txt | 0.139253 GTGGTGGTGTTGGAAGTTGC | ACAGAACAGTGGGATGAGCG | 0.279442 GTGGTGGTGTTGGAAGTTGC | TTTCGGGATGGGAGTTAGCG | 0.285983 GTGGTGTTGGAAGTTGCTGC | ACAGAACAGTGGGATGAGCG | 0.353972 AAGTTGCTGCTTTTCGACGC | ACAGAACAGTGGGATGAGCG | 0.407005 GTGGTGGTGTTGGAAGTTGC | CGAGAATGTATTGGTGCGCC | | | | | |
| NCU01083T0 | 1 | 5 | NCU01083T0\_input.txt | NCU01083T0\_output.txt | 0.423996 TGAGAGAGTAGGCCCACAGG | TAGGTATCGGTTGTGTCGCG | 0.431357 TGAGAGAGTAGGCCCACAGG | TCTCTCGGCCTTCCTCTACC | 0.555747 GGGGAGAAGGTGTAGTTGGC | TCGTGAGACGTTCAGCTACC | 0.569459 TGAGAGAGTAGGCCCACAGG | GACAAAGCCCACCATTCTGC | 0.630126 TTTGAGTCGGGTTTGAGGGG | TCGTGAGACGTTCAGCTACC | | | | | |
| NCU01084T0 | 2 | 5 | NCU01084T0\_input.txt | NCU01084T0\_output.txt | 0.767430 GCTGCTGATGAATCTTCGCC | GATGCACTGTACCGTCCTGG | 0.986495 GAATCGGTTTGGGGTTGAGC | GATGCACTGTACCGTCCTGG | 1.464123 TGTCCAAGACCCGTGATGC | GATGCACTGTACCGTCCTGG | 1.539043 CGTGATGCGTCCTCTCAACC | GATGCACTGTACCGTCCTGG | 1.647163 GCTGCTGATGAATCTTCGCC | TGGGATAAGCTGGTCACAAGC | | | | | |
| NCU01085T0 | 1 | 5 | NCU01085T0\_input.txt | NCU01085T0\_output.txt | 0.151172 GACGACAAAAGCCAACGACC | GACCCAGCAAAAGCATGTCG | 0.291913 GACGACAAAAGCCAACGACC | GTAACATCATCTCCCGCCCC | 0.291913 GACGACAAAAGCCAACGACC | GGTAACATCATCTCCCGCCC | 0.351020 GGGCGGGAGATGATGTTACC | AGACGACATTCATCCCGACG | 0.362698 GACGACAAAAGCCAACGACC | CTTGACTGACCCTGCTGACC | | | | | |
| NCU01086T0 | 2 | 5 | NCU01086T0\_input.txt | NCU01086T0\_output.txt | 0.214999 TGTTCGTATCCAGCCCAACC | GGAGACAATCTTGCGCTTGC | 0.287699 TCCCGTTGTTCGTATCCAGC | GGAGACAATCTTGCGCTTGC | 0.289665 TTCACAAGAAGCTGGGTCCC | GGAGACAATCTTGCGCTTGC | 0.555819 TGTTCGTATCCAGCCCAACC | GAGACAATCTTGCGCTTGCG | 0.628518 TCCCGTTGTTCGTATCCAGC | GAGACAATCTTGCGCTTGCG | | | | | |
| NCU01087T0 | 1 | 5 | NCU01087T0\_input.txt | NCU01087T0\_output.txt | 0.206548 TGAGCCGGGATGACATATGC | TCCGTCACATTCTCTGCTCG | 0.216751 ACGATCTTGGGGGTTGTTCC | ACAAGGTCTGCCACTGAACC | 0.217561 CTAGGCCCTGCACTGAATCC | TCAACTTCCTCGAACGCTCC | 0.279895 CTAGGCCCTGCACTGAATCC | CTTCCTCGAACGCTCCTACC | 0.360279 ACGATCTTGGGGGTTGTTCC | AAGGGCGACATCAGAAACCC | | | | | |
| NCU01088T0 | 3 | 5 | NCU01088T0\_input.txt | NCU01088T0\_output.txt | 0.575135 ACGGGTGGTTTGGCTAAAGG | TGTCCAATCTTGCCGACTCC | 0.576058 GTGGGAGGTGTTGGGAAAGG | TGTCCAATCTTGCCGACTCC | 0.639293 GTGAAGGGTTGGGACAGACG | TGTCCAATCTTGCCGACTCC | 1.067865 ACGGGTGGTTTGGCTAAAGG | CCAGTAATACCCGTGACGGC | 1.068788 GTGGGAGGTGTTGGGAAAGG | CCAGTAATACCCGTGACGGC | | | | | |
[truncated: 3,458,231 more chars]
